# A meta-analysis of multiple matched aCGH/expression cancer datasets reveals regulatory relationships and pathway enrichment of potential oncogenes

**DOI:** 10.1371/journal.pone.0213221

**Published:** 2019-07-23

**Authors:** Richard Newton, Lorenz Wernisch

**Affiliations:** MRC Biostatistics Unit, Cambridge University, Cambridge, United Kingdom; Instituto Nacional de Medicina Genomica, MEXICO

## Abstract

The copy numbers of genes in cancer samples are often highly disrupted and form a natural amplification/deletion experiment encompassing multiple genes. Matched array comparative genomics and transcriptomics datasets from such samples can be used to predict inter-chromosomal gene regulatory relationships. Previously we published the database METAMATCHED, comprising the results from such an analysis of a large number of publically available cancer datasets. Here we investigate genes in the database which are unusual in that their copy number exhibits consistent heterogeneous disruption in a high proportion of the cancer datasets. We assess the potential relevance of these genes to the pathology of the cancer samples, in light of their predicted regulatory relationships and enriched biological pathways. A network-based method was used to identify enriched pathways from the genes’ inferred targets. The analysis predicts both known and new regulator-target interactions and pathway memberships. We examine examples in detail, in particular the gene *POGZ*, which is disrupted in many of the cancer datasets and has an unusually large number of predicted targets, from which the network analysis predicts membership of cancer related pathways. The results suggest close involvement in known cancer pathways of genes exhibiting consistent heterogeneous copy number disruption. Further experimental work would clarify their relevance to tumor biology. The results of the analysis presented in the database METAMATCHED, and included here as an R archive file, constitute a large number of predicted regulatory relationships and pathway memberships which we anticipate will be useful in informing such experiments.

## Introduction

Previously we have demonstrated that an analysis of matched array comparative genomics and transcriptomics human cancer datasets can reveal inter-chromosomal acting gene regulatory relationships [1–3]. By regulatory relationship we are refering to either a direct relationship, of a transcription factor on its target gene, or a very indirect one, through a pathway containing intermediate regulatory steps. We published the database METAMATCHED [4], comprising the results from such an analysis of a large number of publically available cancer datasets. Careful data randomisation ensures statistically significant predictions. Each dataset originated from samples of a particular type of cancer, and the datasets covered a wide range of cancer types.

We noticed that there are genes in the database which have a highly variable copy number amongst samples *within* a dataset and this occurs consistently for these same genes *across many of the datasets* and different cancer types. In this paper we investigate these unusual genes. We investigate their target genes, predicted by the meta-analysis of publically available cancer datasets, the biological pathways enriched in their lists of target genes, and their relevance to the cancer pathology of the samples. Why genes which have a highly variable, inconsistent copy number disruption amongst samples *within* a cancer dataset may, perhaps counter-intuitively, be of relevance to the cancer pathology is examined later in this introduction. Firstly we discuss the background to the meta-analysis and the pathway enrichment analysis.

Array comparative genomics (aCGH) microarrays detect gene deletions or gene amplifications (extra copies) by comparing gene copy numbers in the DNA extracted from test sample cells to the copy numbers in normal control cells. Transcriptomics experiments use microarrays that measure the abundance of mRNA. In matched experiments the two different types of measurement are performed on the same samples. Reviews of matched aCGH and transcriptomics experiments, their analysis and uses can be found in references [5] and [6].

In cancer samples the copy numbers of genes are often greatly altered and are in effect of a natural gene amplification/deletion experiment encompassing many genes. In matched experiments, transcriptomics data is also available for the same samples, and information can be extracted on how changes in a gene’s copy number affects that gene’s expression. The analysis can however be extended further, using the measurements to investigate whether a change in a given gene’s copy number, with an associated change in expression, affects the expression of any of the other genes in the dataset, hence inferring regulatory relationships.

Inference of regulatory relationships from these experiments is not without difficulties. Relationships are masked by the noise in the data and further confounded by the biological complexity of the system being studied. In particular, unlike conventional gene knockdown experiments, the copy number of many genes are being perturbed simultaneously. Coamplification or codeletion of genes situated in the same region of the genome can produce many spurious relationships and it is for this reason that we concentrate on inter-chromosomal acting regulatory relationships.

We validated the hypothesis that useful regulatory information can be extracted from matched datasets, experimentally in Goh et al. [1] and computationally in Newton and Wernisch [2]. We then performed a meta-analysis of 31 publically available datasets [3]. These comprised matched experiments from a variety of different cancer types. Genes that have altered copy number in one cancer type may have altered copy number in other cancer types [7], so combining datasets should help reinforce any information within the data on regulator-target relationships. The results were made available in the METAMATCHED database [4]. For this paper we have extended the meta-analysis to 45 datasets. The 14 extra datasets contain 824 samples, taking the total number of samples in the meta-analysis to 3398 samples.

The meta-analysis predicts target lists for regulatory genes. In this paper, to augment this information, we investigate the enrichment of biological pathways in the target lists. To this end, we used a network-based pathway enrichment approach. An enrichment analysis based on the network of a pathway, rather than simply the gene set of the pathway, takes into consideration the interactions between the genes in the pathway. We use PathwayCommons network databases [8] and the local enrichment analysis (LEAN) method of Gwinner et al. [9] for network analysis of target lists, and then assess the resulting output for biological pathway enrichment using a hypergeometric test. The results of the analysis are presented in the Metamatched database and are also included in this paper’s supplementary material as an R [10] archive file (S3 File).

The aim of the pathway enrichment analysis is three-fold. Firstly, statistically significance enrichments can help to validate the predictions of regulatory relationships generated by the meta-analysis of the matched datasets. Secondly, the enrichment of a regulatory gene’s predicted target gene list in a particular pathway can be used to augment current knowledge of that pathway. Thirdly, the enrichment analysis can be used to investigate the participation of the regulators in pathways implicated in cancers, which is the main focus of this paper.

We are using cancer samples to predict regulatory relationships because they can be seen as a natural gene amplification/deletion experiment. That the disruptions are outside our control does mean however that inference about regulatory relationships will only be possible for certain genes. Those genes with high variation in their copy number amongst the samples *within* a dataset will show the highest self aCGH/expression correlation, provided of course that there is a concomitant change in expression. Conversely, if a gene is not amplified or deleted in any sample in a dataset, or if it is amplified or deleted by a similar amount in all the samples, then little or no self aCGH/expression correlation will be detected and the analysis will be unable to reveal any regulatory relationships for the gene. This means that any oncogenes which are consistently amplified or deleted in cancer samples are unlikely to feature in the results.

We find that there are a number of genes which have a highly variable copy number amongst samples *within* a dataset and this occurs consistently *across many of the datasets* and a very wide variety of different cancer types. There are 47 genes which have significant self aCGH/expression correlation (and predicted targets) in twenty or more datasets. In this paper we assess the potential relevance to the pathology of the cancer samples of genes exhibiting this unusual, consistent heterogeneous copy number disruption in light of their predicted regulatory relationships and enriched biological pathways. A prominent example of such a gene, that also has an unusually large number of target genes predicted by the meta-analysis, is *POGZ* (Pogo Transposable Element Derived With ZNF Domain) and we examine in detail some of the novel pathway involvements predicted for this interesting gene.

It may appear counter-intuitive that genes whose copy number disruption varies greatly between the different cancer samples within a dataset could be relevant to tumor biology. It would suggest that these genes are located in genomic regions which are prone to disruption in cancer cells, but this disruption occurs only sporadically amongst samples. And this inconsistency would suggest that the disruption of these regulators would have little relevance to the actual cancer pathology. It is known however that few genes are altered in more than 10% of tumors for any type of cancer [11] and there is a complex interplay of alterations within pathways in cancers [12]. So it is possible that disruption of several different gene members of a particular pathway, in an inconsistent manner across the various cancer samples in a dataset could result in that pathway being consistently disrupted in all the samples of that cancer type.

Alternatively, these genes may occur in genomic regions which are disrupted, not sporadically, but consistently in the later stages of tumor development. In general the samples within a dataset will come from tumors at different stages of the disease, so the disruption to these particular genes may reflect changes that take place during the course of tumor development. These points are examined further in the Discussion section.

The aim of the work described in this paper was two-fold. Firstly, to augment the information held in the Metamatched database with a comprehensive pathway enrichment analysis. This has added predictions of pathway memberships, as well as predictions of regulatory relationships to the database, and we give examples of the usefulness of these predictions in the following. Secondly, the aim was to explore the relevance of the information in the database to cancer, concentrating on particular prominent regulators highlighted by the meta-analysis. We present arguments as to why these genes could play a role in tumor biology, which appears to be supported by the pathway enrichment results.

## Materials and methods

**Fig 1:**
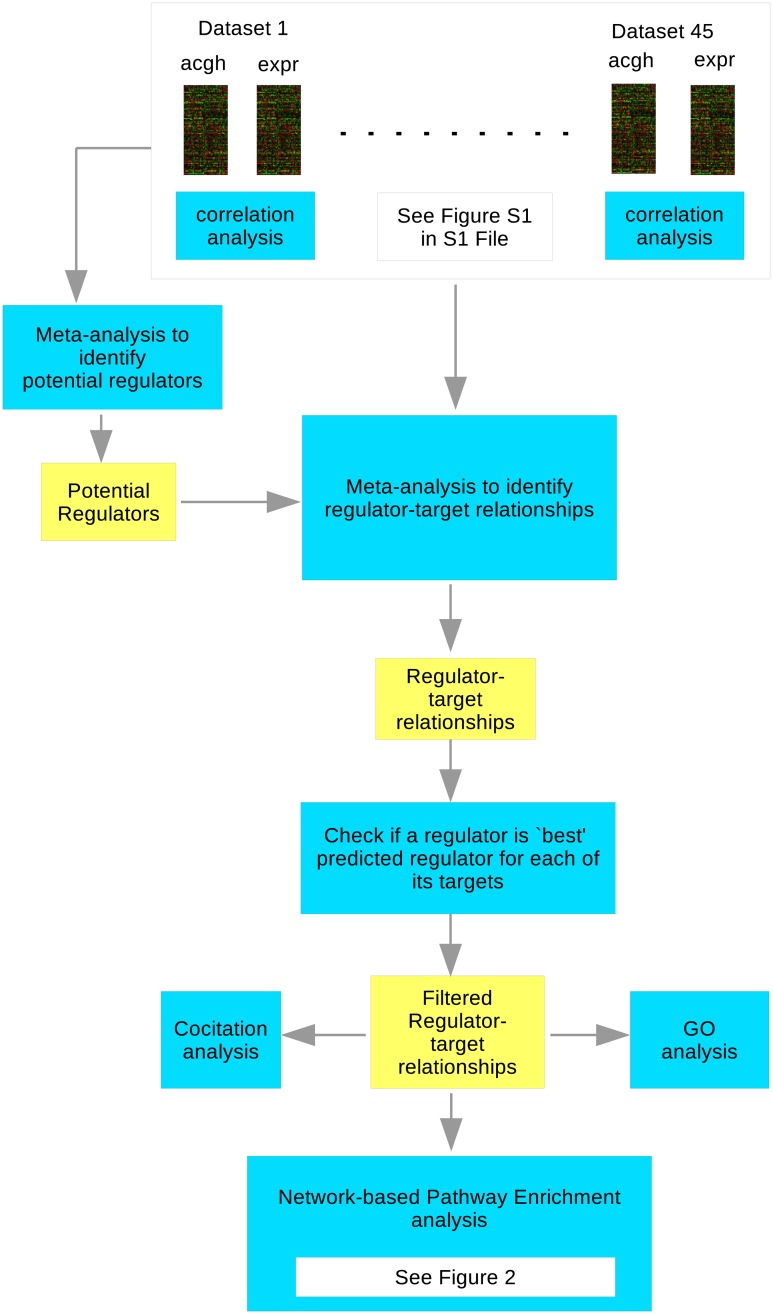
Schematic diagram showing the steps involved in the analysis.

In this section we first give a brief summary of the previously published meta-analysis method we use to predict gene regulatory relationships. We then describe the network-based pathway enrichment approach we use in this paper to analyse the results from the meta-analysis. Fig 1 is a flow chart illustrating all the steps involved in the analysis from array data through to enriched pathways.

### Prediction of regulatory relationships

We infer inter-chromosomal acting gene regulatory relationships from a meta-analysis of 45 matched aCGH/transcriptomics datasets, which are listed in Table 1. We use a method based on correlations, a robust approach for analysing relationships amongst large amounts of data of unknown complexities. More sophisticated network inference methods are generally more likely to be susceptible to noise and heterogeneity between datasets. A major advantage of our method is that it avoids the risk of confounding that can occur when expression data alone is used in the analysis. Careful data randomisation to generate a null distribution is used to ensure statistically significant predictions. Full details of the algorithm can be found in references [3], [2] and in [1], where the code, written in the R statistical environment [10], can also be found. The details are also provided in supplementary information S1 File.

**Table 1:** Details of the 45 datasets used in the meta-analysis, the original 31 followed by the 14 new datasets.

Code	GEO	Publication	N	P	Pathology
parr	GSE20486	[13]	97	18616	Breast Cancer (Diploid)
crow	GSE15134	[14]	31	16153	Breast Cancer (ER+)
sirc	GSE17907	[15]	51	14689	Breast Cancer (ERBB2 amplified)
myll	*	[16]	46	17050	Gastric Cancer
junn	*	[17]	10	16844	Gastric Cancer
ch.w	†	[18]	91	10285	Lung adenocarcinoma
ch.s	†	[18]	94	10285	Lung adenocarcinoma
hoac	GSE20154	[19]	54	14388	Oesophageal adenocarcinoma
zho	GSE29023	[20]	115	13697	Multiple Myeloma
shai	GSE26089	[21]	68	14201	Pancreatic Cancer
vain	GSE28403	[22]	13	10107	Prostate Cancer
bott	GSE29211	[23]	53	10321	Pleural Mesothelioma
bekh	GSE23720	[24]	173	13682	Breast Cancer (Inflammatory)
chap	GSE26863	[25]	245	13667	Multiple Myeloma
ooi	GSE22785	[26]	14	10091	Neuroblastoma
brag	GSE12668	[27]	11	10310	Waldenström’s Macroglobulinemia
jons	GSE22133	[28]	356	4183	Breast Cancer
mura	GSE24707	[29]	47	4472	Breast Cancer
lin1	GSE19915	[30]	72	4965	Urothelial Carcinoma
beck	GSE17555	[31]	18	12174	Leiomyosarcoma
toed	GSE18166	[32]	74	4289	Astrocytic Gliomas
ell	GSE35191	[33]	124	13569	Breast Cancer
gra.1	GSE35988	[34]	85	12849	Prostate Cancer
gra.2	GSE35988	[34]	34	12813	Prostate Cancer
lenz	GSE11318	[35]	203	15212	Lymphoma
lin2	GSE32549	[36]	131	8450	Urothelial Carcinoma
micc	GSE38230	[37]	12	16657	Vulva Squamous Cell Carcinoma
tayl	GSE21032	[38]	155	14572	Prostate Cancer
coco	GSE25711 ‡	[39]	36	4394	Neuroblastoma
med	GSE14079	[40]	8	6376	Lung Cancer
przy	GSE54188	[41]	53	17032	Synovial Sarcoma
huang	GSE30311	[42]	98	14927	Ovarian Cancer
lira	GSE34211	[43]	89	14907	Cancer Cell lines
chpy.1	GSE34171	[44]	87	14907	Diffuse Large B-cell Lymphoma
chpy.2	GSE34171	[44]	78	10437	Diffuse Large B-cell Lymphoma
ross	GSE70770	[45]	78	15150	Prostate Cancer
ochs	GSE33232		69	14489	Head and Neck Squamous Cell Carcinoma
rama	GSE19539	[46]	67	14972	Ovarian Cancer
guar	GSE66399	[47]	65	14833	Breast Cancer
wilk	GSE36471	[48]	47	13681	Lung Adenocarcinoma
dona	GSE32688	[49]	32	14833	Pancreatic Cancer
zhu	GSE12805		31	11639	Osteosarcoma
kuij	GSE33383	[50]	29	14339	Osteosarcoma
pau	GSE26576	[51]	29	14883	Diffuse Intrinsic Pontine Glioma
weig	GSE57549	[52]	25	15150	Breast Cancer

GEO = Gene Expression Omnibus dataset reference (http://www.ncbi.nlm.nih.gov/geo/), N = Number of samples, P = Number of matched probes,

* http://www.cangem.org/,

^†^ http://cbio.mskcc.org/Public/lung_array_data/,

^‡^ Expression data in ArrayExpress (http://www.ebi.ac.uk/arrayexpress/): E-TABM-38, E-MTAB-161. Expts. ch, gra & chpy use 2 expr. platforms, so samples from each platform treated as separate dataset, to avoid spurious correlations which may be caused by systematic shifts between the 2 sets of expr. data. Each contributes 2 datasets to study, resulting in 45 d’sets from 42 expts.

We refer to a ‘regulating gene’ or ‘regulator’ as one whose up or down expression change has a direct or indirect effect on the up or down regulation of a ‘target gene’. Any gene whose mRNA expression levels are significantly correlated with changes in its own copy number is considered worth investigating as a potential regulating gene. A potential target gene of a regulating gene has expression levels which are significantly correlated with the copy number alterations of the regulating gene.

A gene appearing in a regulator’s list of predicted targets, does not mean that regulator is the most probable regulator for that target. Therefore, for each potential regulator, all predicted targets were assessed to see if the data indicated a more probable gene as its regulator. There are two possible criteria for assigning the best regulator for a target gene: either by minimum *p*-value from the meta-analysis or by the maximum number of datasets where there is significant correlation between the target’s expression and the regulator’s aCGH.

In the following we use the short-hand ‘significant best target’ to refer to a predicted target of a regulator which is first of all significant (*p*-value < 0.05). Moreover this regulator is predicted to be the best regulator for the target out of all the regulators in the Metamatched analysis by way of *both* minimum *p*-value *and* having the maximum number of datasets where there is significant correlation between the target’s expression and the regulator’s aCGH. We use the short-hand ‘less stringent condition for best regulator’ to refer to assigning a regulator as the best regulator of a target by way of *either* minimum *p*-value *or* having the maximum number of datasets where there is significant correlation between the target’s expression and the regulator’s aCGH.

### Network-based pathway enrichment analysis

#### Introduction

For each regulator, pathway enrichment was investigated separately using a network based analysis of the list of its target genes. The networks used were four databases of gene interactions in PathwayCommons [8], namely Humancyc (v20; 2016) [53], Panther (v3.6.1; 25-Jan-2018) [54], Pid (NCI Curated Human Pathways; 27-Jul-2015) [55] and Reactome (v64; 26-Mar-2018) [56], in the SIF (Simple interaction file) format. In the four databases there are a total of 9849 pathways, whose membership ranged from 2 to 2405 genes.

The SIF format is a flat-file compendium of pairwise gene interactions documenting on each line two genes, the nature of their interaction (comprising one of eight different interaction types), and a biological pathway annotation for the interaction and/or a Pubmed ID (PMID) [57]. Not all interactions are annotated with a pathway or PMID and the pathway annotations may be composite pathways, each member in the list being separated by a semi-colon. We use the term node of the network to refer to a gene and the term edge of the network to refer to the undirected interaction between two genes. In the following pathway analysis we will make use of this annotation of edges by pathways. We refer to a gene as being annotated with a pathway if at least one of the edges connected to the gene is annotated with the pathway.

**Fig 2:**
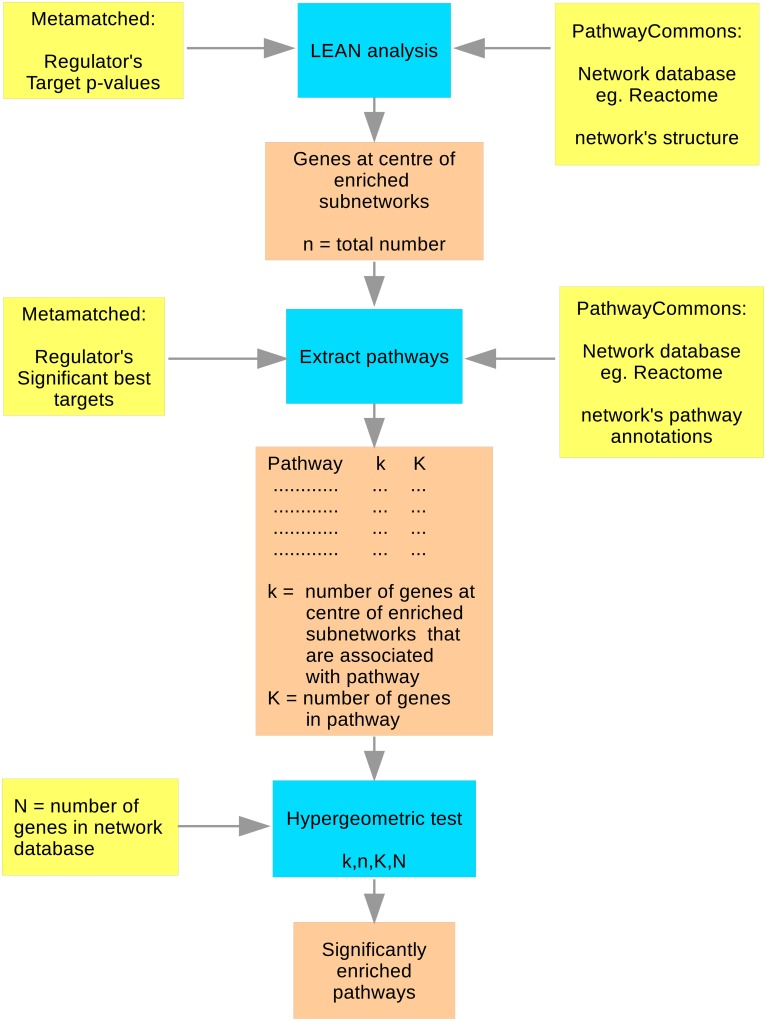
Schematic diagram showing the steps involved in the network-based pathway enrichment analysis.

The analysis consisted of two steps. Firstly the target gene list of the regulator was analysed in order to find enrichment of local subnetworks within the given PathwayCommons network database. This step uses the network structure encapsulated in the PathwayCommons database. Then the set of enriched local subnetworks discovered in the first step were investigated for the presence of enriched biological pathways according to the pathway annotations in the PathwayCommons database. Fig 2 shows a flow diagram of the different steps in the analysis, which are described in the following two sections.

#### Network analysis

The local enrichment analysis (LEAN) method of Gwinner et al. [9] was used for the network analysis. The purpose of this analysis is to find subnetworks of genes which together have unusually low *p*-values. The LEAN method examines *local* subnetworks, each of which comprises a single centre gene and its direct neighbours in a network database. All possible local subnetworks (centre genes) are evaluated. Evaluation of a local subnetwork takes as input the *p*-values of the centre gene and its direct neighbours, calculates an enrichment score and uses a null distribution derived from random sampling to return an enrichment *p*-value. The parameter free and exhaustive aspect of the LEAN method makes it particularly suitable for a comprehensive analysis of large numbers of disparate gene lists.

For a regulator, the input to the LEAN algorithm was the complete list of target gene *p*-values from the meta-analysis for this regulator. That is, the *p*-values for the correlation between the regulator’s aCGH and the target genes’ expression. For each target gene the minimum of the *p*-value for activation by the regulator and the *p*-value for repression by the regulator was selected.

A gene was considered to be an enriched centre gene, at the centre of a significantly enriched subnetwork, if the LEAN *p*-value for the local subnetwork was less than 0.05.

#### Pathway enrichment analysis

The LEAN analysis of a regulator’s target gene list generates a number of significantly enriched local subnetworks for a given PathwayCommons network database. In this step the results of the network analysis are summarised and condensed in order to extract significantly enriched biological pathways.

The following procedure was carried out for each regulator in each PathwayCommons network database in which the regulator shows significant enrichment of its target list. For each significantly enriched local subnetwork found for the regulator, biological pathways associated with the subnetwork were recorded by noting the pathways assigned to the edges of the subnetwork in the PathwayCommons network database. Not all edges were included; only the edges attached to a gene for which the regulator is predicted to be the best regulator and where this regulator-target relationship is significant (*p*-value < 0.05). The stringent rule was applied as to whether a regulator is predicted to be the best regulator for a target gene.

The results of applying this procedure for all the significantly enriched local subnetworks of the regulator were collated to give a list of pathways and the number of enriched centre genes associated with each of the pathways. The hypergeometric distribution was then used to test whether the number of occurences for a particular pathway was more than expected by chance.

The hypergeometric distribution provides the distribution of finding *k* objects with a certain feature in a random sample of size *n* out of a population of *N* objects where a total of *K* objects has this feature. Here *k* is the number of enriched centre genes associated with a pathway, *n* is the total number of enriched centre genes in the LEAN analysis for this regulator, *K* is the number of genes recorded as being members of this pathway in the PathwayCommons database and *N* is the total number of genes in the PathwayCommons database. Significantly enriched pathways were selected as those with adjusted *p*-value from the hypergeometric test less than 0.05.

The pathway with the minimum hypergeometric *p*-value was recorded as the main pathway for the regulator. As well as using the pathways in the PathwayCommons databases, the analysis used the Pubmed Ids if an interaction was not annotated with an actual pathway name.

If a regulator itself was not annotated on any of its edges with one of its significantly enriched pathways then the R package igraph [58] was used to discover whether the regulator had any path in the network database to any of the genes whose edges were annotated with the pathway. The package igraph was also used to create plots of the significantly enriched pathways for a regulator. The biological pathways derived from the combined local subnetworks could be too large for plotting and required some pruning. S1 File contains details of how this was performed.

## Results

In this section we first summarize the results of the meta-analysis. We then describe the primary results of the paper, namely the outcome of the network-based pathway enrichment anaysis. We first provide an overview of these results and then concentrate on genes exhibiting consistent heterogeneous copy number disruption, examining some of their enriched pathways in detail and their relevance to cancer biology.

### Meta-analysis

From the meta-analysis of 45 matched aCGH/transcriptomics datasets we infer inter-chromosomal acting gene regulatory relationships. The complete results of the analysis can be found in the METAMATCHED database [4]. A description of the composition of the database can be found in S1 File. The analysis found 15496 genes considered worth investigating as potential regulators; that is, having significant correlation (adjusted *p*-value < 0.05) between their copy number profile and gene expression profile in at least one of the datasets. Of the 15496 potential regulators, 1176 were found to have at least one significant predicted target (adjusted *p*-value < 0.05). S2 File is a spreadsheet that summarises the results for the regulatory genes. The complete results, in R archive format are available for download from the Metamatched website, and are also included here as supplementary file S3 File.

### Network analysis

The target gene list of each regulator was analysed for enrichment of local subnetworks within four PathwayCommons network databases using the LEAN method [9]. The set of enriched local subnetworks were then investigated for the presence of enriched biological pathways using a hypergeometric test. After the results from the LEAN analysis have been summarised and condensed in this way a total of 250 regulators show significant pathway enrichment of their target gene lists. Each regulator that demonstrates some pathway enrichment is assigned a main pathway (or Pubmed ID) as described in the Methods section. The 250 regulators have a total of 152 unique main pathways (see Table A in S4 File). The number of genes assigned to the 152 main pathways in the PathwayCommons database ranged from 5 to 262 genes, with a mean of 65.

Each regulator will, in general, have a number of significantly enriched pathways besides a main pathway; the number ranges from 1 to 89 pathways, with a mean of 5. The 250 regulators between them have a total of 663 unique enriched pathways (see Table B in S4 File).

Summary pathway enrichment information is displayed for each of the regulators on its webpage in the Metamatched database. More detailed information can be downloaded from the web page; a spreadsheet summarises the pathway enrichment results, an R archive file contains comprehensive information on the enrichment of each of the pathways and plots on the web page illustrate these pathways.

We classified the enriched pathways into twenty major pathway types based on the top-level pathway classifications in Reactome. We assessed the frequency of occurrence of each pathway type by counting the number of regulators with target lists which are enriched with at least one pathway of the pathway type. In the sum of the number of regulators enriched with a particular pathway type the values were weighted by the number of datasets in which each regulator showed significant self aCGH/expression correlation. However, the number of genes annotated with a top-level pathway type in the PathwayCommons databases vary greatly, so the frequencies of occurrence of a pathway type were corrected for the number of annotated genes. Frequencies of occurrence were normalised so that the highest value was one.

**Fig 3:**
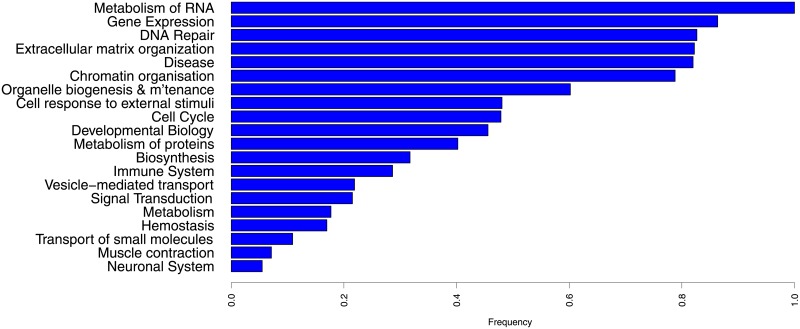
Frequency of occurrence of the major types of pathway amongst the enriched pathways of the 250 regulators.

A pathway type will feature high in the ranking if it is enriched in the target lists of many regulators and these regulators have self aCGH/expression correlation in many datasets, and hence many types of cancer. The results are shown in Fig 3. The most frequently occurring pathway types were Metabolism of RNA, Gene Expression (primarily RNA Polymerase II Transcription) and DNA Repair (primarily DNA Double-Strand Break Repair).

### Genes exhibiting consistent heterogeneous copy number disruption

We focus on those regulatory genes which have significant correlation between their aCGH profiles and their own expression profiles in the highest number of datasets. The top genes in this respect are listed in Table 2, which gives the number of datasets in which significant self aCGH/expression correlation is found, the number of significant targets for which the gene is the best regulator from the meta-analysis and the main pathway for the gene from the network analysis.

**Table 2:** Regulatory genes with self aCGH/expression correlation in the greatest number of datasets.

Regulator	Targs	D’sets	Main pathway
AZIN1	2	22	Regulation of ornithine decarboxylase (ODC)
AGO2	7	21	Transcriptional regulation by small RNAs
DERL1	3	21	E3 ubiquitin ligases ubiquitinate target proteins
PTK2	3	21	PMID: 9187108 [59] *; 9256433 [60] **
BCL9	1	21	Formation of the beta-catenin:TCF transactivating complex
POGZ	33	20	RNA polymerase II transcribes snRNA genes
MRPS28	4	19	Mitochondrial translation termination
YWHAZ	4	19	ATR signaling pathway
CHD1	2	18	Estrogen-dependent gene expression
ZC3H3	1	18	Cleavage of Growing Transcript in the Termination Region;Transport of Mature mRNA derived from an Intron-Containing Transcript;mRNA 3’-end processing
HSBP1	5	17	CXCR4-mediated signaling events;IL12 signaling mediated by STAT4;IL12-mediated signaling events;TCR signaling in naïve CD4+ T cells
ANKRD46	4	17	RUNX1 regulates genes involved in megakaryocyte differentiation and platelet function
RABGAP1	1	17	Regulation of gene expression in beta cells
TRAK1	1	17	Signaling by BRAF and RAF fusions
TERF2IP	8	16	Acetylcholine regulates insulin secretion;Activation of … ***
RBBP5	2	16	Activation of anterior HOX genes in hindbrain development during early embryogenesis;RUNX1 regulates genes involved in megakaryocyte differentiation and platelet function
ATG7	1	16	Antigen processing: Ubiquitination and Proteasome degradation;Interconversion of nucleotide di- and triphosphates
NCOA6	1	16	Activation of anterior HOX genes in hindbrain development during early embryogenesis
SLC30A5	1	16	NEP/NS2 Interacts with the Cellular Export Machinery;NS1 Mediated … ****
VCPIP1	1	16	Ovarian tumor domain proteases
WWOX	1	16	Formation of the beta-catenin:TCF transactivating complex
PTS	6	15	tetrahydrobiopterin biosynthesis I;tetrahydrobiopterin biosynthesis II
DOCK1	1	15	Integrin signalling pathway

Targs = Number of significant targets for which regulator is the best regulator *p*-value < 0.05, D’sets = Number of datasets in which the regulator has significant correlation between its aCGH profile and its own expression profile

* TEP1, encoded by a candidate tumor suppressor locus, is a novel protein tyrosine phosphatase regulated by transforming growth factor beta.

** P-TEN, the tumor suppressor from human chromosome 10q23, is a dual-specificity phosphatase.

*** Acetylcholine regulates insulin secretion;Activation of NF-kappaB in B cells;Activation of RAS in B cells;Antigen activates B Cell Receptor (BCR) leading to generation of second messengers;Arachidonate production from DAG;Ca2+ pathway;EGFR Transactivation by Gastrin;Effects of PIP2 hydrolysis;Elevation of cytosolic Ca2+ levels;Fatty Acids bound to GPR40 (FFAR1) regulate insulin secretion;G alpha (q) signalling events;G beta:gamma signalling through PLC beta;GPVI-mediated activation cascade;Rap1 signalling;Response to elevated platelet cytosolic Ca2+;Syndecan interactions;Synthesis of IP3 and IP4 in the cytosol.

****NEP/NS2 Interacts with the Cellular Export Machinery;NS1 Mediated Effects on Host Pathways;Nuclear Pore Complex (NPC) Disassembly;Nuclear import of Rev protein;Regulation of Glucokinase by Glucokinase Regulatory Protein;Regulation of HSF1-mediated heat shock response;Rev-mediated nuclear export of HIV RNA;SUMOylation of DNA damage response and repair proteins;SUMOylation of DNA replication proteins;SUMOylation of RNA binding proteins;SUMOylation of chromatin organization proteins;Transcriptional regulation by small RNAs;Transport of Mature mRNA Derived from an Intronless Transcript;Transport of Mature mRNA derived from an Intron-Containing Transcript;Transport of Ribonucleoproteins into the Host Nucleus;Transport of the SLBP Dependant Mature mRNA;Transport of the SLBP independent Mature mRNA;Viral Messenger RNA Synthesis;Vpr-mediated nuclear import of PICs;snRNP Assembly;tRNA processing in the nucleus.

**Fig 4:**
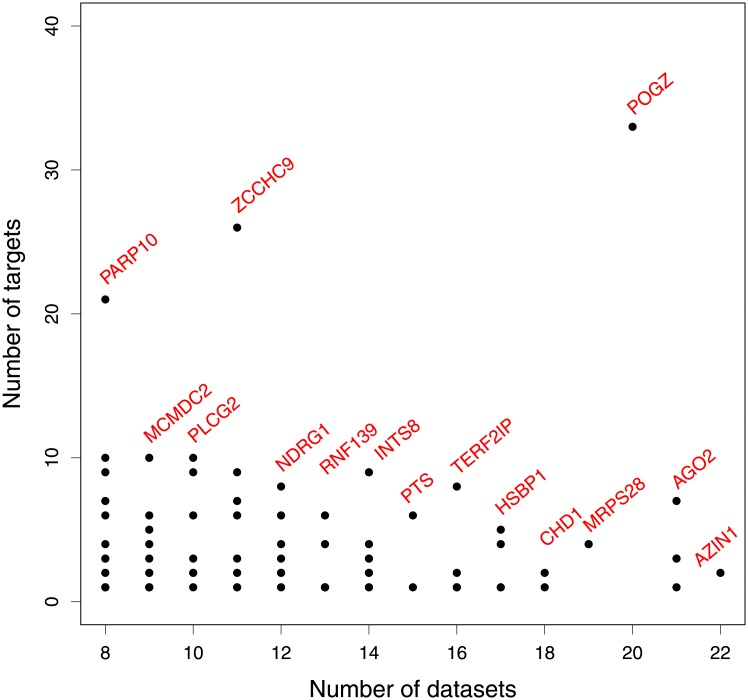
For regulators, number of significant best targets against number of datasets. For each regulator, plotting the number of their predicted significant best targets against the number of datasets in which they show significant self aCGH/expression correlation. Showing only regulators with self-correlation in 8 or more datasets and regulators with at least one enriched pathway. The regulator with the maximum number of targets at each number of datasets is annotated.

The full list can be found in Table D in S4 File. This information is also displayed graphically in Fig 4 which plots for each regulator the number of significant best targets against the number of datasets in which it shows significant self aCGH/expression correlation. There are genes in the Metamatched analysis which show significant self correlation in a higher number of datasets (up to 28 datasets, see S2 File), but which do not have significantly enriched pathways so are not included in Fig 4.

In the following we examine some of the enriched pathways of these regulatory genes in detail in order to assess their potential relevance to tumor biology. Enrichment of a pathway in a gene’s target list can also provide further evidence for previously known interactions amongst genes or validate the predictions made by the Metamatched analysis. In addition it can be used to predict novel participants in pathways in two ways.

Firstly, if a regulator is not currently known to participate in a pathway, but the regulator’s target list is enriched in that pathway, the enrichment suggests that the regulator has a connection with the pathway and some effect on the pathway’s function. We investigated whether each regulator was already known to be part of its enriched pathways or had any network path to any known member of the pathways in the relevant PathwayCommons database. We found that 118 of the regulators are known to be connected to all their enriched pathways, 100 to none, and the remaining 32 to some of their enriched pathways. In Table B in S4 File, which tabulates each of the 663 enriched pathways, the regulators associated with a pathway are divided into those that are known to be connected to the pathway and those that are not known to be connected to the pathway.

Secondly, even if a regulator is already known to be part of a pathway for which its target gene list is enriched, in general there will be genes in its target gene list which are not known to be associated with the pathway. The prediction of these genes as targets of the regulator suggests they may be participants in the pathway. In general, the majority of a regulator’s significant best targets are not known to be involved in its enriched pathways (see Methods section for definition of ‘significant best target’). The details of the network analysis for each regulator that can be downloaded from the Metamatched website includes, for each enriched pathway, those significant best targets which are known to be connected to the pathway and those which are not known to be connected.

In the examples we concentrate on some of the pathways found to be the most disrupted pathways in the 45 datasets used in the analysis. As a measure of the disruption of a pathway we used the number of datasets in which at least one regulator associated by enrichment with this pathway has significant copy number disruption, with a concomitant change in expression. The top disrupted pathways are listed in Table 3 and the full list is given in Table C in S4 File. Note that the pathway entries for gene pairs in PathwayCommons may be composite pathways, separated by a semi-colon. In the LEAN analysis these composite pathways have been used, but in compiling Table 3 composite entries have first been split into their component pathways. For brevity in the table, if two pathways are found to have exactly the same complement of regulators associated with them by enrichment then the pathways have been concatenated by a vertical-bar symbol. This generates 353 unique pathways, or pathway combinations, from the 663 composite pathways.

**Table 3:** Most disrupted pathways in the 45 datasets used in the analysis.

Pathway	Regulators	N
Downstream TCR signaling	ABCC12 ARHGEF4 ATXN7L2 HSBP1 IGSF9 MEF2C MNS1 PBXIP1 PIK3R1 PIP5K1A PLCG2 POGZ PSMB10 PTK2 RASGRF2 TERF2IP VHL	33
Generation of second messenger molecules	ARHGEF4 ATXN7L2 HSBP1 PIP5K1A PLCG2 POGZ PTK2 TERF2IP VHL	33
Antigen activates B Cell Receptor (BCR) leading to generation of second messengers	ABCC12 ARHGEF4 ATXN7L2 ETV7 GALE IGSF9 INTS8 MCMDC2 MEF2C MNS1 PBXIP1 PEBP4 PIK3R1 PIP5K1A PLCG2 PTK2 RASGRF2 TERF2IP	31
Formation of the beta-catenin:TCF transactivating complex	BCL9 CPEB4 HIST2H2BE HOXD9 LYL1 MYC NIPAL4 POGZ PYGO2 RAB3A RBBP5 WWOX ZNF446	31
RUNX1 regulates genes involved in megakaryocyte differentiation and platelet function	ANKRD46 DAAM2 HIST2H2BE HOXD9 KMT2E LYL1 NIPAL4 POGZ RAB3A RBBP5 ZNF446	31
PD-1 signaling	ARHGEF4 ATXN7L2 HSBP1 MNS1 POGZ VHL	31
Phosphorylation of CD3 and TCR zeta chains ∣ Translocation of ZAP-70 to Immunological synapse	ARHGEF4 ATXN7L2 HSBP1 POGZ VHL	31
FCERI mediated Ca+2 mobilization ∣ Role of phospholipids in phagocytosis	ABCC12 ETV7 IGSF9 MCMDC2 MEF2C MNS1 PBXIP1 PEBP4 PIK3R1 PIP5K1A PLCG2 PTK2 RASGRF2 TERF2IP	30
SUMOylation of DNA damage response and repair proteins	ASNSD1 GUCA2B HOXD9 LINC01587 NR3C2 NSMCE2 PHC3 SLC30A5 SSB TNNT2 TSPAN7 XRCC4	30
Ca2+ pathway	ETV7 GALE INTS8 MCMDC2 PEBP4 PIP5K1A PLCG2 PTK2 TERF2IP	30
DAG and IP3 signaling ∣ PLC beta mediated events ∣ VEGFR2 mediated cell proliferation	ETV7 MCMDC2 PEBP4 PIP5K1A PLCG2 PTK2 TERF2IP	30
Major pathway of rRNA processing in the nucleolus and cytosol	AGO2 BCAN CDX1 CFHR4 DENND1C DUOX1 FAM212B FCRL5 GKN1 IDH1 KCNK4 KDELR1 KLK6 MYC NIP7 PDE4C PRKACA PROCR RIPK3 RPL13A RPS23 RPS4X SEC24D SF3B4 STARD4 TLE2 ZCCHC9	29
Transcriptional regulation by small RNAs	AGO2 ASNSD1 BCAN GIMAP2 GUCA2B HIST2H2BE HOXD9 LINC01587 LYL1 NIPAL4 NR3C2 RAB3A SLC30A5 SSB TNNT2 TSPAN7 ZNF446	29
snRNP Assembly	ASNSD1 CFHR4 FIGF GCG GEMIN7 GEMIN8 GUCA2B HOXD9 LINC01587 NR3C2 POGZ SLC30A5 SSB TNNT2 TSPAN7	29
GPVI-mediated activation cascade	ABCC12 GALE IGSF9 INTS8 MEF2C MNS1 PBXIP1 PIK3R1 PIP5K1A PLCG2 PTK2 RASGRF2 TERF2IP	29
Estrogen-dependent gene expression	AGO2 CHD1 HIST2H2BE IQGAP2 LYL1 MYC NIPAL4 PIEZO1 POGZ RAB3A ZNF446	29
FGF signaling pathway	ARHGEF4 COTL1 GAB2 PIK3R1 PLCG2 POGZ PTK2 SELPLG TNFSF10	29
G alpha (q) signalling events	INTS8 MNS1 NPY4R PIP5K1A PLCG2 PTK2 TERF2IP	29
Activation of anterior HOX genes in hindbrain development during early embryogenesis	DAAM2 HIST2H2BE LYL1 NCOA6 NIPAL4 PARP10 PAX6 PLCG2 POGZ RAB3A RBBP5 VHL ZNF446	28
PKMTs methylate histone lysines	ASH1L DAAM2 DQX1 KMT2E LYL1 MECOM PARP10 POGZ RBBP5	28
G beta:gamma signalling through PLC beta	GALE INTS8 PIP5K1A PLCG2 PTK2 TERF2IP	28
MHC class II antigen presentation	DCTN4 GIMAP2 HSBP1 POGZ RASGRF2 RILP	28

N = Total number of datasets in which pathway potentially disrupted

We now look at three examples of enriched pathways in detail (a further two are given in supplementary information S5 File):

#### Pathway example 1: Formation of the beta-catenin:TCF transactivating complex

Beta-catenin plays an important part in the Wnt-signaling pathway and is itself controlled by binding partners such as the TCF family of transcription factors that affect its stability and localization. It participates in various processes such as gene expression and cell adhesion. Mutations in beta-catenin and the partners regulating its stability can contribute to tumorgenesis [61].

Thirteen regulators have this pathway as one of their enriched pathways, namely *BCL9* (21), *POGZ* (20), *RBBP5* (16), *WWOX* (16), *CPEB4* (9), *MYC* (8), *PYGO2* (7), *LYL1* (3), *ZNF446* (3), *HOXD9* (2), *NIPAL4* (2), *RAB3A* (2) and *HIST2H2BE* (2). The numbers in brackets indicate the number of datasets in which the regulator has significant self aCGH/expression correlation.

*BCL9 (B Cell CLL/Lymphoma 9)* has significant self-correlation in 21 datasets. It is known to be part of this pathway, being annotated with the pathway in Reactome.

*POGZ (Pogo Transposable Element Derived With ZNF Domain)*, a protein coding gene, has significant self-correlation in 20 of the datasets in Metamatched (Table 2). It also has an unusually high number of significant targets for which it is predicted to be the best regulator; 33 in total (see Fig 4). It is not annotated with this pathway, and there is no path in Reactome from *POGZ* to known members of this pathway, so the pathway enrichment analysis is suggesting a novel involvement of *POGZ* in the pathway ‘Formation of the beta-catenin:TCF transactivating complex’, disruption of which is of relevance to tumor biology.

The pathway enrichment analysis of the predicted targets for *POGZ* infers a total of 17 enriched pathways (Table 4), fifteen of them in Reactome and two in Panther. The functional role of *POGZ* is, as yet, not well characterised in the literature and it is not annotated with these pathways, neither is there any path in Reactome or Panther from *POGZ* to known members of the pathways, so the enrichment analysis in conjunction with the meta-analysis is suggesting a novel involvement of *POGZ* in all of these pathways.

**Table 4:** Enriched pathways of POGZ with associated significant best targets.

Pathway	Targets
TGF-beta signaling pathway	ACVR2B EP400
EGF receptor signaling pathway;FGF signaling pathway	RASA1
RNA polymerase II transcribes snRNA genes	SP1 YY1 TRRAP EP400
RUNX1 regulates genes involved in megakaryocyte differentiation and platelet function;RUNX1 regulates transcription of genes involved in differentiation of HSCs	YY1 TRRAP KMT2A SMC4 EP400
Activation of anterior HOX genes in hindbrain development during early embryogenesis;Estrogen-dependent gene expression	YY1 TRRAP KMT2A SMC4 EP400
Condensation of Prophase Chromosomes	YY1 TRRAP KMT2A SMC4 EP400
snRNP Assembly	GEMIN5
MHC class II antigen presentation	SP1 HLA-DPA1
Formation of the beta-catenin:TCF transactivating complex;Ub-specific processing proteases	YY1 TRRAP KMT2A SMC4 EP400
PKMTs methylate histone lysines;RUNX1 regulates genes involved in megakaryocyte differentiation and platelet function;RUNX1 regulates transcription of genes involved in differentiation of HSCs	YY1 TRRAP KMT2A SMC4 EP400
Formation of the beta-catenin:TCF transactivating complex;HATs acetylate histones	YY1 TRRAP KMT2A SMC4 EP400 NAT8L
HATs acetylate histones	SP1 YY1 TRRAP KMT2A SMC4 EP400 NAT8L
DNA Damage Recognition in GG-NER	YY1
HATs acetylate histones;Ub-specific processing proteases	TRRAP EP400
Formation of the beta-catenin:TCF transactivating complex;HATs acetylate histones;Ub-specific processing proteases	YY1 TRRAP KMT2A SMC4 EP400
Downstream TCR signaling;Generation of second messenger molecules;Interferon gamma signaling;MHC class II antigen presentation;PD-1 signaling;Phosphorylation of CD3 and TCR zeta chains;Translocation of ZAP-70 to Immunological synapse	HLA-DPA1
Downstream TCR signaling;Generation of second messenger molecules;PD-1 signaling;Phosphorylation of CD3 and TCR zeta chains;Translocation of ZAP-70 to Immunological synapse	HLA-DPA1

*POGZ* is known to encode a protein containing a zinc-finger cluster, an HP1-binding motif, a centromere protein-B-like DNA-binding (CENPB-DB) domain, and a transposase-derived DDE domain [62]. The HP1-binding domain of POGZ is required for mitotic progression and dissociation of HP1*α* from mitotic chromosome arms and for activation and dissociation of Aurora B kinase from chromosome arms at M phase [63]. POGZ has also been found to interact with glucocorticoid receptors, which regulate various metabolic, homeostatic and differentiation processes [64], and with the C terminus of LEDGF/p75 via its DDE domain [65, 66], and with trimethyl-lysine modifications on histones that control chromatin-mediated regulation of gene expression, and with the mitotic spindle checkpoint protein MAD2L2 [67].

*POGZ* has six significant best targets associated with the pathway ‘Formation of the beta-catenin:TCF transactivating complex’ in the enrichment analysis. *TTRAP* is annotated with this pathway in Reactome, *EP400* and *YY1* are centres of enriched local subnetworks, and *SMC4*, *KMT2A* and *NAT8L* are part of enriched local subnetworks.

**Fig 5:**
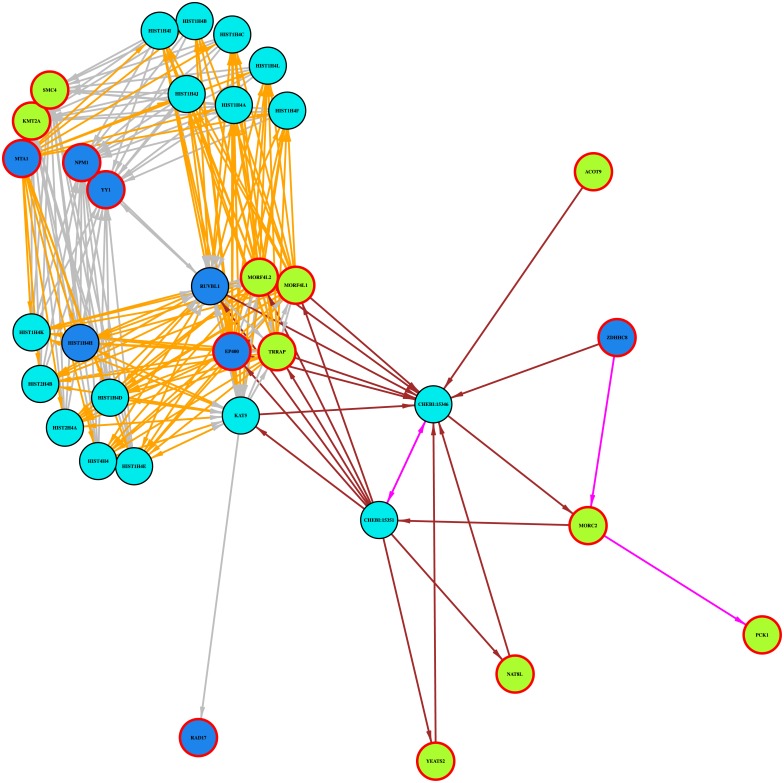
POGZ: Formation of the beta-catenin:TCF transactivating complex. Simplified diagram of the pathway ‘Formation of the beta-catenin:TCF transactivating complex’ as enriched in the target list of the gene POGZ. Applying the less stringent criterion for a regulator to be the best regulator of targets. Nodes: Green = significant target, Red border = regulator is best regulator for target Dark Blue = enriched centre also significant target, Light Blue = enriched centre but not significant target. Edges: grey = ‘in-complex-with’, purple = ‘catalysis-precedes’, ‘used-to-produce’ orange = ‘controls-state-change-of’, ‘controls-phosphorylation-of’ brown = ‘controls-expression-of’, ‘cntrls-production-of’, consumption-cntrlled-by’.

If the stringency of the best regulator condition is relaxed (see Methods section for details), then more significant targets of *POGZ* appear in the pathway. Fig 5 plots the enriched network when the less stringent condition is applied. The enrichment analysis now associates sixteen significant targets for which *POGZ* is the best predicted regulator with the pathway.

Loss of function mutations in *POGZ* are known to be associated with severe neurodevelopmental delay, resulting in a majority of cells being unable to form metaphase plates, exiting mitosis prematurely and causing the formation of polyploidy cells, which can lead to cell death or genome instability in subsequent division cycles [68]. *POGZ* mutations that disrupt the DNA-binding activity of POGZ are also associated with Autism spectrum disorder (ASD) [62, 69, 70]. Loss of *POGZ* has also been found to affect the proliferation of mouse neural progenitor cells [71].

Another better characterised gene associated with ASD, *CHD8*, interacts with *POGZ* [72] and is known to be involved with beta-catenin pathways [73], and mutations in pathways regulating beta-catenin have been shown to occur in ASD [74]. *POGZ* expression is also correlated with *CTNND2* (Catenin Delta 2) [75], providing circumstantial support for *POGZ*’s involvement in the beta-catenin pathways predicted here by the pathway enrichment analysis of the regulatory relationships inferred from the meta-analysis of matched datasets.

Disruption of *CHD8* is also implicated in cancer [76], as is the disruption of the destruction complex of beta-catenin [77]. Thus the genomic disruption of *POGZ* in many of the cancer datasets used in our study and the concomitant changes in its expression, and the significant correlation of the expression changes of an unusually high number of targets, may indicate that *POGZ* has a functional role in cancer biology. The datasets in which *POGZ* showed significant aCGH/expression correlation corresponded to the following tissues and cancer pathologies: Breast (ER+, ERBB2 amplified, Inflammatory), Head and Neck Squamous Cell, Lung Adenocarcinoma, Lymphoma, Multiple Myeloma, Osteosarcoma, Ovarian, Pancreatic, Prostate and Urothelial.

*RBBP5 (RB Binding Protein 5)* has significant self-correlation in 16 datasets. It is known to be involved in signaling by Wnt, and in Reactome it is annotated with the pathway ‘Formation of the beta-catenin:TCF transactivating complex’, as is one of its two significant best targets, *SETDB2*. Its other significant best target is *SLTM*. If the less stringent condition for *RBBP5* to be the best regulator is applied then a further significant best target, *CDCA5*, is included in the pathway.

*WWOX (WW Domain Containing Oxidoreductase)* has significant self-correlation in 16 datasets. ‘Formation of the beta-catenin:TCF transactivating complex’, is an enriched pathway for *WWOX* due to its predicted significant best target *LEF1*. This prediction agrees with prior knowledge of *WWOX* in that it has a known regulatory relationship with *LEF1* involving the beta-catenin pathway. That is, *WWOX* interacts with *DVL2*, inhibiting the function of *DVL2* in controlling the transcriptional activity of *LEF1*, and also by interacting with a cofactor of the Wnt/beta-catenin pathway, BCL9-2, to enhance the activity of the beta-catenin-TCF/LEF transcription factor complexes [78, 79]. *WWOX* is also known to act as a tumor suppressor [80].

If the less stringent condition for *WWOX* to be the best regulator is applied then five further significant best targets are included in the pathway, in addition to *LEF1*, namely *SUV39H1*, *TLE1*, *SIN3A*, *KDM4B* and *RCL1*. None are annotated with this pathway but they are associated with the pathway by the enrichment analysis as either enriched centres or connected to enriched centres. Overexpression of *SUV39H1* is known to be associated with cell proliferation in cancer [81]. *SIN3A* is downregulated in a variety of cancers and is thought to influence a specific step of tumorgenesis in part via the beta-catenin pathway [82]. *KDM4B* overexpression contributes to the genesis of colorectal tumors via its role in beta-catenin mediated gene transcription [83].

*CPEB4 (Cytoplasmic Polyadenylation Element Binding Protein 4)* has significant self-correlation in 9 of the datasets and has ‘Formation of the beta-catenin:TCF transactivating complex’ as an enriched pathway through its significant best target *TCF7L2*. In Reactome *CPEB4* is not connected to members of the pathway. Besides *TCF7L2* it has four other significant best targets, namely *NQO2*, *CES4A*, *TET1* and *ZNF26*. *CES4A* has no path to the pathway in Reactome, indicating that it may be a novel member of this pathway. If the less stringent condition is applied then the gene *CROT* is also a significant target of *CPEB4* in this pathway.

*CPEB4* has been shown to have a role in oncogenesis through translational activation of mRNAs that are normally silenced [84]. Silencing of *CPEB4* prevents cell invasion and migration in non-small cell lung cancer [85]. High *CPEB4* expression can serve as a prognostic factor in invasive ductal breast carcinoma [86]. The related gene *CPEB1* regulates beta-catenin mRNA translation [87].

*MYC (MYC Proto-Oncogene)* has significant self-correlation in 8 of the datasets and is known to be part of the pathway ‘Formation of the beta-catenin:TCF transactivating complex’. Similarly *PYGO2 (Pygopus Family PHD Finger 2)*, with significant self-correlation in 7 datasets, is also annotated with this pathway in Reactome.

#### Pathway example 2: RUNX1 regulates genes involved in megakaryocyte differentiation and platelet function

RUNX1 is a transcription factor, known to be essential for the maturation of megakaryocytes [88]. It is known as a tumor suppressor in leukemia but has recently been implicated to have a role in other cancer types [89, 90]. A total of eleven regulators have their target lists enriched with this pathway in Metamatched, namely *POGZ* (20), *ANKRD46* (17), *RBBP5* (16), *KMT2E* (9), *DAAM2* (4), *LYL1* (3), *ZNF446* (3), *HOXD9* (2), *NIPAL4* (2), *HIST2H2BE* (2) and *RAB3A* (2).

*POGZ (Pogo Transposable Element Derived With ZNF Domain)*: There is no path in Reactome from *POGZ* to members of the pathway ‘RUNX1 regulates genes involved in megakaryocyte differentiation’ however it has been shown that *POGZ* is highly expressed in mouse megakaryocyte erythroid progenitors [71].

Five of the thirty three significant best targets of *POGZ* are associated with the pathway in the enrichment analysis. Of the five, *KMT2A* is annotated with this pathway in Reactome, *EP400* and *YY1* are at the centres of enriched local subnetworks, and *SMC4* and *TRRAP* are part of enriched local subnetworks. Of the remaining 28, 11 do not currently appear in Reactome, one occurs but has no path to any members of the pathway, and 14 do have paths to members of this pathway. If the less stringent condition for a regulator to be the best regulator is applied then a further four significant best targets are associated with the pathway, namely *MORF4L1*, *MORF4L2*, *MTA1* and *YEATS2*.

*ANKRD46 (Ankyrin Repeat Domain 46)* is also not known to be part of this pathway, but is associated in this analysis by gene *KAT2B* being a significant best target. Whereas, *RBBP5 (RB Binding Protein 5, Histone Lysine Methyltransferase Complex Subunit)* and *KMT2E (Lysine Methyltransferase 2E)* are both annotated with this pathway in Reactome.

#### Pathway example 3: HDMs demethylate histones

By removing methyl groups from histone proteins Histone demethylases (HDMs) contribute to epigenetic regulation by reversing histone methylation [91] and given their epigenetic role they are of interest as therapeutic targets in cancer [92].

**Fig 6:**
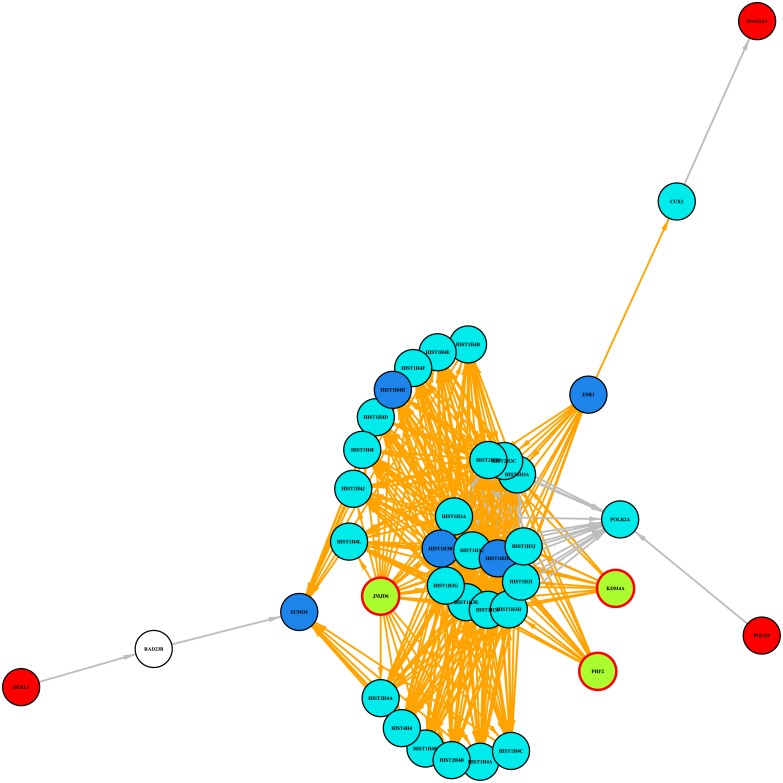
DERL1, MAN2A1 and POLD3: HDMs demethylate histones. Simplified diagram of the pathway ‘HDMs demethylate histones’ fusing the results of the enrichment analysis for regulators DERL1, MAN2A1 and POLD3. Key to nodes and edges as in Fig 5 with the addition of: Nodes: Red = regulator, White = target, not significant.

In Metamatched three regulators have this pathway enriched in their target lists: *DERL1* (21), *MAN2A1* (13) and *POLD3* (11). Fig 6 shows a simplified pathway diagram derived by amalgamating the results of the enrichment analysis for these three regulators.

*DERL1 (Derlin1)* is a component of the endoplasmic reticulum-associated mechanism for the degradation of misfolded proteins. It demonstrates significant self correlation in 21 of the datasets and has 3 significant best targets, *JMJD6*, *ZKSCAN7* and *CCPG1*. Its main pathway from the enrichment analysis is ‘HDMs demethylate histones’. DERL1 is not known to be part of this pathway, but in Reactome it has a path to members of the pathway by way of forming a complex with RAD23B, which in turn is connected to SUMO1. Significant best target *JMJD6* is associated with the pathway. Of the two other significant best targets *CCPG1* does not currently appear in Reactome whilst *ZKSCAN7* does have a path to members of this pathway. If the stringency of the condition for a regulator to be a target’s best regulator is relaxed then *POLD3* is also included as a significant best target. *POLD3* (see below) is itself a potential regulator in the Metamatched analysis and interestingly its own target list is also enriched with the pathway ‘HDMs demethylate histones’.

*POLD3 (DNA Polymerase Delta 3, Accessory Subunit)* plays an important role in DNA replication and repair. Depletion of *POLD3* results in an increase in genome instability, by way of breaks, S-phase progression impairment and chromosome abnormalities [93]. It has self correlation in 11 datasets and has 7 significant best targets, one of which, *PHF2*, is associated with this pathway. Four of the others have some path to the pathway and two do not appear in Reactome. *POLD3* has a path to *PHF2* via *POLR2A*. If the less stringent condition is applied then five further significant best targets are associated with the pathway, *DOT1L*, *RARG*, *SUPT5H*, *TAF1L* and *PDCD7*.

*MAN2A1 (Mannosidase Alpha Class 2A Member 1)* has self correlation in 13 datasets and one significant best target, *KDM4A*. *MAN2A1* is not annotated with this pathway, but can be connected to members of the pathway in Reactome.

## Discussion

Previously we have used matched aCGH/expression datasets to predict statistically significant gene regulatory relationships [3], validated the method experimentally [1] and computationally [2]. In this context regulatory relationship refers to more than just direct casual relationships of transcription factors on targets, encompassing indirect casual relationships as well, through pathways containing intermediate regulatory steps.

The data used in the analysis consists of more than three thousand cancer samples so in this paper we explore whether the results can provide insights into tumor biology. To this end we have carried out an exhaustive network-based pathway enrichment analysis. We concentrate on those genes that are unusual in manifesting copy number heterogeneity, and a concomitant change in expression, in many of the 45 datasets and a very wide variety of different cancer types.

This consistent heterogeneity could indicate genes located in genomic regions which are prone to disruption in cancer cells, but this disruption occurs only sporadically amongst samples, suggesting little relevance to the actual cancer pathology. However it is known that genes are rarely altered in more than 10% of tumors for any type of cancer [11] and there is a complex interplay of alterations within pathways in cancer [12]. So disruption of more than one member of a pathway in an inconsistent manner across samples could result in that pathway being consistently disrupted in all of the samples. We plan to explore this aspect of the data further in future work since it has been recognised that phenotypic heterogeneity between tumors, in terms of the pathways disrupted, is much less than the observed genetic heterogeneity [94].

An alternative possibility is that these genes occur in genomic regions which are disrupted consistently in the later stages of tumor development. In general the samples within an experiment will come from tumors at different stages of the disease, so the disruption to these particular genes may reflect changes that take place during the course of tumor development. In this case the disruption to regulators and their pathways might have a role in later phases of the disease, for example in the development of metastases. Along with tumor heterogeneity, metastases are a major challenge facing cancer therapy [95].

In some cases the pathway enrichment analysis predicts known down-stream target genes of a regulator, and some examples of this feature in the Results section. In other cases however known down-stream targets are not predicted. Such missing predictions may be due to lack of predictive power of the analysis. Alternatively they may result from cellular mechanisms able to compensate for a regulator’s copy number disruption and concomitant change in expression. For example, they may represent regulator-target relationships that are highly non-linear so that large changes in a regulator’s expression has little effect on down-stream components of the pathway. Activation of an alternative pathway, or pathways, that can compensate for the disruption to a pathway is another possible method for nullifying the effects of changes in a regulator’s expression.

If cellular mechanisms are responsible, then the analysis presented here may be highlighting those regulator-target relationships that lack redundancy or any buffering process in the cell. They are relationships where disruption to the regulator’s expression, due to changes in its copy number, cannot be compensated for by the cell and so may have a real functional effect. The genetic diversity of tumors is a major problem in cancer therapy and the aim of personalized cancer therapy is to identify mutations that are clinically relevant. This involves distinguishing ‘driver’ mutations from neutral ‘passenger’ mutations that tend to accumulate during tumorgenesis [11, 96]. If this study is highlighting functionally important regulators, then some of these may be ‘driver’ genes whose disruption is relevant to tumor biology.

So despite the inconsistent disruption of the copy number across samples of the genes in this study, it is possible to hypothesize that these genes could be regulators that have a role in tumor biology. The pathway memberships predicted by the network analysis of the meta-analysis results do suggest a close involvement of some of these genes in known cancer pathways. This includes genes for which there is currently little or no information on their relevance to cancer, especially multiple types of cancer, but which have cancer related pathways enriched in the target lists predicted for them by the meta-analysis of matched experiments.

The pathway enrichment analysis shows some interesting examples of direct validation, for example the relationship between *WWOX* and *LEF1* in the beta-catenin pathway, and circumstantial validation, for example the enrichment of the target list of *POGZ* with the megakaryocytes and beta-catenin pathways.

In order to reduce false positives we use high confidence levels and a stringent condition as to whether a regulator is the best regulator for a target in our analysis. Inevitably the stringency may remove some true positives. An example can be seen in the results for the regulator *WWOX* where applying the less stringent condition reveals further significant best targets known to be involved with beta-catenin pathways. Consequently the Metamatched database also provides the significant targets of a regulator found using the less stringent condition for a regulator to be the best regulator of a target.

The work highlights those genes disrupted in many of the datasets. One of the most interesting of these regulators is *POGZ*. It stands out in having self aCGH/expression correlation in a large number of datasets and cancer types, and also an unusually high number of predicted significant targets (Fig 3) and some novel enriched pathways. Whilst not connected in Reactome to these pathways, and not yet well characterised in the literature, prior knowledge of the role of the gene *POGZ* does provide some support for the enriched pathways predicted by the network analysis presented here.

A major advantage of the analytical approach used, that is correlation of aCGH with expression, is that it avoids the risk of confounding that can occur when using the correlation of expression profiles alone. One problem of the analysis is the potential difficulty of distinguishing the targets of two regulators which are neighbours in the genome and are often co-disrupted. The more datasets in which a regulator demonstrates copy number disruption, the less likely this will occur. The relationships that can be investigated are also necessarily constrained by the probes that are available on the arrays used in the experiments.

The aim of the predictions in the Metamatched database is to inform experiments and provide information for generating gene regulatory networks. Since our analysis builds on the causal influence of the regulator gene on its target, the predictions of pathway membership may help to infer the downstream effects of drugs on their targets.

As discussed in more detail in our previous publication [3] it is possible that some of the predicted relationships in this study have arisen through a confounding factor as part of some as yet poorly understood or unknown genetic mechanisms. For example histone modification regulated by microRNAs promoting copy number variation [97, 98] or hypoxia-dependent copy gain [99] are possible mechanisms by which confounding could occur. This means that as well as detecting direct interactions, and indirect ones through pathways, the analysis may be detecting subtler genomic effects, which cannot be isolated with current knowledge.

In future work we plan to explore whether the inconsistent genomic disruption of multiple genes within a dataset leads to a more consistent phenotypic disruption of the samples in terms of pathways altered. It would be interesting to identify which subsets of regulators are required to be altered to consistently perturb a particular pathway within a dataset, where these genes are positioned in the pathway and what role they perform there. We also plan to update the METAMATCHED database in line with updates to PathwayCommons, GO and cocitation information, and to integrate the results with other oncogenomics databases. In addition there are alternative analysis methods which could be employed, for example, Pearson correlation, or maximal information-based nonparametric exploration statistics [100], a technique designed to cope with non-linearities in the data being analysed.

## Conclusion

We have added information from an exhaustive network-based pathway enrichment analysis to METAMATCHED, a database of statistically significant regulator-target predictions. In this paper we explore the relevance of these results to tumor biology. We have concentrated on genes exhibiting consistent heterogeneous copy number disruption and presented arguments why these genes could be of relevance to cancer pathways, which appear to be supported by the pathway enrichment results. The wealth of predicted regulatory relationships and pathway memberships contained in the Metamatched database provide pointers as to possible experiments that could clarify their role in cancer. We demonstrate how the predictions contained in the database can be useful in informing experiments and extending networks of regulatory relationships. We provide some interesting examples of this process, in particular for the gene *POGZ*.

## Supporting information

S1 File Supplementary information for methods and results sections. (PDF)

S2 File Summary results for 2172 regulators. A spreadsheet that summarises the Metamatched results for 2172 regulators that have at least one significant predicted target (adjusted *p*-value < 0.1).
(CSV)

S3 File Complete results for 2172 regulators. Results for 2172 genes, in R archive format. Unpacks as an R list object named ‘regulators.details’ with a format described in S1 File.
(RDATA)

S4 File Supplementary tables. (PDF)

S5 File Supplementary results—Network analysis. (PDF)
