# Supplementary material for: A meta-analysis of multiple matched aCGH/expression cancer datasets reveals regulatory relationships and pathway enrichment of potential oncogenes
Source: PLoS One. 2019 Jul 23;14(7):e0213221. doi: 10.1371/journal.pone.0213221 (PMC6650054; doi:10.1371/journal.pone.0213221)
Supplement: S1 File — (PDF) [file pone.0213221.s001.pdf]

S1 File: Supplementary information
...
"A meta-analysis of multiple matched aCGH/expression cancer datasets reveals regulatory relationships and pathway enrichment of potential oncogenes"
...

Richard Newton & Lorenz Wernisch

February 11, 2019

1 Supplementary Methods

1.1 Data

Table S1 lists the 42 experiments used in the current meta-analysis. Some of the experiments utilized two different expression platforms and the samples from each expression platform were treated as a separate data set. This is done in order to avoid the possibility of spurious correlations which may be caused by systematic distortions or shifts between the two sets of expression data. This situation pertains to 3 of the experiments, so each of these 3 experiments contribute 2 data sets to the study, resulting in a total of 45 data sets from the 42 experiments.

Pre-processing was as described in Newton & Wernisch [41], namely the aCGH data was location and scale normalized using the median and mad, as was the expression data. The aCGH and expression probes were mapped by the gene names of probes to give the maximum number of probes with corresponding aCGH and expression profiles. If necessary probe gene names were converted from synonyms to standard gene names using the database of the HUGO Gene Nomenclature Committee (HGNC) [42]. If there was more than one probe for any gene name then the median value of the probes was taken to represent that gene name. The aCGH data was not thresholded so that, in general, fractional rather than integer aCGH values were used in the analysis. Fractional variations in copy number occur because of the heterogeneity of the cancer samples being studied. By using matched aCGH and expression profiles we eliminated the effects of a sample's heterogeneity considering that both sets of data were affected equally.

1.2 Analysis

1.2.1 Introduction

In order to infer regulatory relationships we use a method based on correlations, a robust approach for analysing relationships amongst large amounts of data of unknown complexities. More sophisticated network inference methods are generally much more susceptible to noise and heterogeneity between data sets. The great strength of our approach is that it avoids the confounding that can occur when expression data alone is used in the analysis. Full details of the algorithm can be found in Newton & Wernisch [41], Newton & Wernisch [43] and in Goh et al. [44], where the code, written in the R statistical environment [45], can also be found. Here we provide only a brief description of the method used.

We refer to a 'regulating gene' as one whose up or down expression change has a direct or indirect effect on the up or down regulation of a 'target gene'. A gene which demonstrates changes in mRNA expression levels which are significantly correlated with its own copy number changes is considered worth investigating as a potential regulating gene. Potential target genes of a regulating gene are those genes with significant correlation between the expression changes of the target gene and the copy number alterations of the regulating gene. Figure S1 illustrates the steps involved in the analysis.

1.2.2 Identifying potential regulators

For each gene 45 Spearman rank correlations (from the 45 data sets), and their p-values for being greater than zero, were calculated (R function cor.test). The 45 correlation p-values for a gene were combined into a single p-value statistic using the adaptive rank truncated

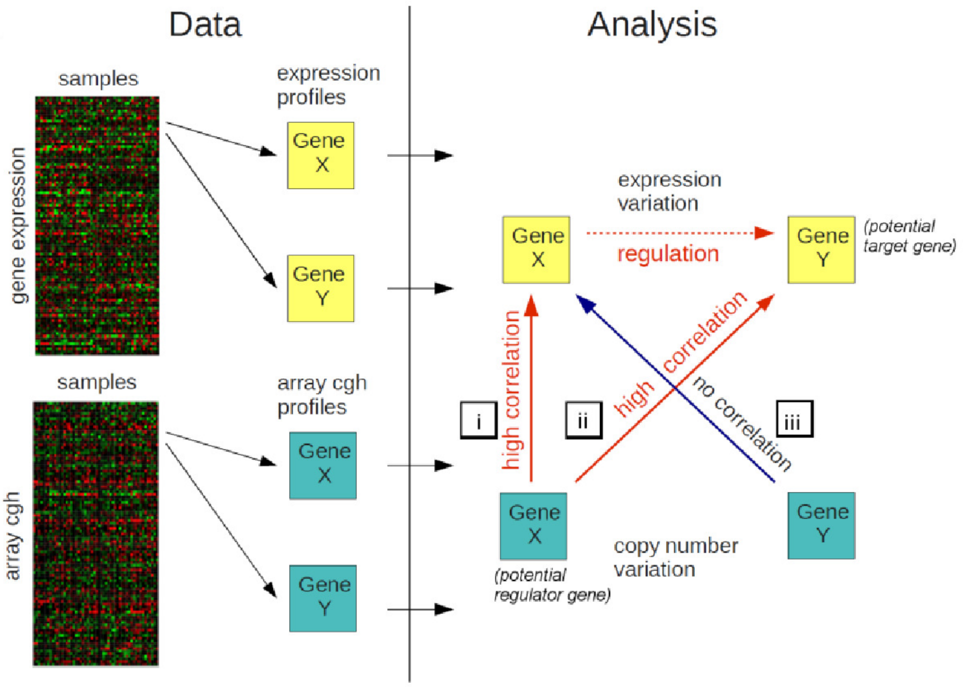

Figure S1: **Schematic diagram illustrating the key analysis steps.**

product method [46]. In order not to rely on any statistical assumptions we obtained a null distribution of combined p-value statistics through permutation of gene identifiers ($5 \cdot 10^6$ permutations). A final p-value for a gene was calculated using its combined p-value and the null distribution of combined p-values. The final p-values were then corrected for multiple testing by the Benjamini-Hochberg (B-H) method, to give false discovery rates (fdr) for genes based on their aCGH/expression correlations in the 45 data sets. In the following the Benjamini-Hochberg adjusted p-values are referred to as adjusted p-values and are now fdr values rather than p-values in the sense of a type I error.

1.2.3 Predicting regulator-target relationships

Expression changes of a potential *target* gene must correlate highly with its regulating gene's aCGH profile. Only inter-chromosomal acting relationships were investigated. The correlation tests were similar to those in the previous section to find potential regulators but we tested separately the two alternative hypotheses: that the correlation of a regulator-target pair is greater than zero and that the correlation is less than zero. For each potential regulator only those data sets were included in the analysis for which that regulator had a significant self aCGH/expression correlation.

Just because a gene appears in a regulator's list of predicted targets, does not mean that regulator is the most probable regulator for that target. Therefore, for each potential regulator, any predicted inter-chromosomal acted targets were assessed to see if the data indicated an alternative, more probable gene as its regulator. There are two possible criteria for assigning the best regulator for a target gene, that is, best by way of minimum p-value from the meta-analysis or best by having the maximum number of datasets where there is significant correlation between the target's expression and the regulator's aCGH.

We use the short-hand ‘significant best target’ to refer to a predicted target of a regulator which is first of all significant (p-value < 0.05), and this regulator is predicted to be the best regulator for the target out of all the regulators in the Metmatched analysis by way of *both* minimum p-value *and* having the maximum number of datasets where there is significant correlation between the target’s expression and the regulator’s aCGH. We also use the short-hand ‘less stringent condition for best regulator’ to refer to assigning a regulator as the best regulator of a target by way of *either* minimum p-value *or* having the maximum number of datasets where there is significant correlation between the target’s expression and the regulator’s aCGH.

1.2.4 Co-citations

Co-citation analysis was performed using the Bioconductor [47] package org.Hs.eg.db [48] (version 3.6.0) and functions from the package CoCiteStats [49]. We restricted the analysis to papers which contain fewer than 100 genes, in order to exclude papers that cite very many genes. We performed a resampling to see whether the proportion of cocitations found could occur by chance in a random list of genes of the same length, and used this to assign a p-value to the proportion found.

1.2.5 Gene Ontology annotations

Gene Ontology (GO) [50] annotations for gene lists comprising the regulator and its predicted target were found using the Bioconductor packages GOstats [51] and GO.db [52] (version 3.6.0).

Table S1: **Details of the 45 data sets used in the meta-analysis, the original 31 followed by the 14 new data sets.**

Code	GEO	Publication	N	P	Pathology
parr	GSE20486	Parris et al. 2010 [1]	97	18616	Breast Cancer (Diploid)
crow	GSE15134	Crowder et al. 2009 [2]	31	16153	Breast Cancer (ER+)
sirc	GSE17907	Sircoulomb et al. 2010 [3]	51	14689	Breast Cancer (ERBB2 amplified)
myll	∗	Myllykangas et al. 2008 [4]	46	17050	Gastric Cancer
junn	∗	Junnila et al. 2010[5]	10	16844	Gastric Cancer
ch.w	†	Chitale et al. 2009 [6]	91	10285	Lung adenocarcinoma
ch.s	†	Chitale et al. 2009 [6]	94	10285	Lung adenocarcinoma
hoac	GSE20154	Goh et al. 2011 [7]	54	14388	Oesophageal adenocarcinoma
zho	GSE29023	Zhou et al. 2012 [8]	115	13697	Multiple Myeloma
shai	GSE26089	Shain et al. 2012 [9]	68	14201	Pancreatic Cancer
vain	GSE28403	Vainio et al. 2012 [10]	13	10107	Prostate Cancer
bott	GSE29211	Bott et al. 2011 [11]	53	10321	Pleural Mesothelioma
bekh	GSE23720	Bekhouche et al. 2011 [12]	173	13682	Breast Cancer (Inflammatory)
chap	GSE26863	Chapman et al. 2011 [13]	245	13667	Multiple Myeloma
ooi	GSE22785	Ooi et al. 2012 [14]	14	10091	Neuroblastoma
brag	GSE12668	Braggio et al. 2009 [15]	11	10310	Waldenströms Macroglobulinemia
jons	GSE22133	Jönsson et al. 2010 [16]	356	4183	Breast Cancer
mura	GSE24707	Muranen et al. 2011 [17]	47	4472	Breast Cancer
lin1	GSE19915	Lindgren et al. 2010 [18]	72	4965	Urothelial Carcinoma
beck	GSE17555	Beck et al. 2010 [19]	18	12174	Leiomyosarcoma
toed	GSE18166	Toedt et al. 2011 [20]	74	4289	Astrocytic Gliomas
ell	GSE35191	Ellis et al. 2012 [21]	124	13569	Breast Cancer
gra.1	GSE35988	Grasso et al. 2012 [22]	85	12849	Prostate Cancer
gra.2	GSE35988	Grasso et al. 2012 [22]	34	12813	Prostate Cancer
lenz	GSE11318	Lenz et al. 2009 [23]	203	15212	Lymphoma
lin2	GSE32549	Lindgren et al. 2012 [24]	131	8450	Urothelial Carcinoma
micc	GSE38230	Micci et al. 2013 [25]	12	16657	Vulva Squamous Cell Carcinoma
tayl	GSE21032	Taylor et al. 2010 [26]	155	14572	Prostate Cancer
coco	GSE25711 ‡	Coco et al. 2012 [27]	36	4394	Neuroblastoma
med	GSE14079	Medina et al. 2009 [28]	8	6376	Lung Cancer
przy	GSE54188	Przybyl et al. 2014 [29]	53	17032	Synovial Sarcoma
huang	GSE30311	Huang et al. 2012 [30]	98	14927	Ovarian Cancer
lira	GSE34211	Hook et al. 2012 [31]	89	14907	Cancer Cell lines
chpy.1	GSE34171	Monti et al. 2012 [32]	87	14907	Diffuse Large B-cell Lymphoma
chpy.2	GSE34171	Monti et al. 2012 [32]	78	10437	Diffuse Large B-cell Lymphoma
ross	GSE70770	Ross-Adams et al. 2015 [33]	78	15150	Prostate Cancer
ochs	GSE33232		69	14489	Head and Neck Squamous Cell Carci
rama	GSE19539	Ramakrishna et al. 2010 [34]	67	14972	Ovarian Cancer
guar	GSE66399	Guarneri et al. [35]	65	14833	Breast Cancer
wilk	GSE36471	Wilkerson et al. 2012 [36]	47	13681	Lung Adenocarcinoma
dona	GSE32688	Donahue et al. 2012 [37]	32	14833	Pancreatic Cancer
zhu	GSE12805		31	11639	Osteosarcoma
kuij	GSE33383	Kuijjer et al. 2012 [38]	29	14339	Osteosarcoma
pau	GSE26576	Paugh et al. 2011 [39]	29	14883	Diffuse Intrinsic Pontine Glioma
weig	GSE57549	Weigelt et al. 2015 [40]	25	15150	Breast Cancer

GEO = Gene Expression Omnibus data set reference (http://www.ncbi.nlm.nih.gov/geo/), N = Number of samples, P = Number of matched probes, ∗ http://www.cangem.org/, † http://cbio.mskcc.org/Public/lung_array_data/, ‡ Expression data in ArrayExpress (http://www.ebi.ac.uk/arrayexpress/): E-TABM-38, E-MTAB-161

1.2.6 Pathway Enrichment

The R package igraph [53] was used to create network plots of the enriched pathways for a regulator. The derived pathway networks of combined local subnetworks could in general include many nodes and so required pruning prior to plotting. The following steps were used to achieve this:

1. For each regulator:
2. For each of the regulator's significant pathways:
3. Take all local subnetworks that have the pathway annotated on at least one edge.
4. List all genes in these local subnetworks (centre genes and all genes directly connected to centre genes) and also the regulator.
5. Filter the pathway SIF file to include all node pair entries that include at least one gene from this gene list.
6. Form a graph from the filtered SIF. This graph will contain the genes in the gene list and any genes which are connected to them in the pathway database
7. Remove nodes from the graph which are not in the gene list and are not significant targets of the regulator.
8. Except, if the regulator is in the filtered SIF do not remove any genes which connect the regulator to members of the pathway even if they are not significant targets.
9. If the graph has more than 100 nodes perform further pruning, namely:
10. Include only the local subnetwork centre genes, the regulator's target genes if they are significant *and/or* the regulator is the best regulator for the target, and also the regulator and connecting genes (if present).
11. If the graph still has more than 100 nodes perform further pruning, namely:
12. Include only the local subnetwork centre genes, the regulator's target genes if they are significant *and* the regulator is the best regulator for the target, and also the regulator and connecting genes (if present).

2 Supplementary Results

2.1 METAMATCHED Database

2.1.1 Summary

The complete results of the analysis can be found in the METAMATCHED database of inferred regulatory relationships, available at:

http://sysbio.mrc-bsu.cam.ac.uk/METAMATCHED

The analysis found 15496 genes considered worth investigating as potential regulators; that is, having significant correlation (adjusted p-value < 0.05) between their copy number profile and gene expression profile in at least one of the data sets. This was out of 19498 genes which occur in at least one of the 45 datasets.

Of the 15496 potential regulators, 1176 were found to have at least one significant predicted target (adjusted p-value < 0.05). 250 of these regulators were found to have target lists enriched in at least one of the four Pathway Commons network databases. In the database a significance level of adjusted p-value < 0.1 is used giving 2172 regulators with significant predicted targets. Additional file 2 is a spreadsheet that summarises the results for those regulators that have at least one significant target and/or pathway enrichment. The complete results for these 2172 regulators in R archive format are available for download from the Metamatched website, and are also included in this publication as Additional file 3.

There are a total of 137035 predicted regulator-target pairs in the database. The number of predicted targets for a regulator ranged from 1 to 888, the mean value being 9. Of the 2172 regulators, 379 are known to be involved in transcription regulation in humans (list from AMIGO version: 2.4.26 [54]). A total of 559 of the 2172 regulators have cocitations with at least one of their predicted targets; 24 predicted targets being the maximum number of targets cocited with any regulator. Examining cocitations between genes within the target list of a regulator, 1597 of the 2172 regulators feature cocitations between some of the genes in their target list and 315 of these were significantly more cocitations than expected to occur by chance (adjusted p-value < 0.1).

In the METAMATCHED database a web page for a gene as a regulator gives summary statistics for the gene, namely the number of predicted target genes activated and repressed by the regulator, the number of regulator-target pairs which have cocitations, the total number of papers which cocite at least two genes from a list containing the predicted targets and the regulator, and the statistical significance of finding this number of cocites. Pathway enrichment information is displayed, if any has been detected by the LEAN analysis, namely the PathwayCommons databases in which local subnetwork enrichment occurs, the total number of centre genes which are enriched and the main pathway (or PMID) found.

The predicted target genes are listed on the web page and there are links to spreadsheets giving more detailed information on each target's relationship with the regulator, for example, the number of datasets, and in which datasets, the relationship is significant.

Consistent coamplification or codeletion of potential regulators in the same region of the genome, coupled with the inherent noise in the data, can lead to ambiguity as to which of the regulators is regulating a particular target gene. So the spreadsheet results files contain two columns giving for each target the best predicted regulator in the database based on the criterion of minimum p-value and the best regulator in the database based on

the criterion of significance in the most number of data sets. Further information on the possible regulators for any target gene can be found in the web page containing details in the database on the gene *as a target*. This page has spreadsheets giving all the significant regulators of the target gene in question.

In the new version of METAMATCHED the spreadsheets has an additional column. If a target is at the centre of an enriched local subnetwork the column will contain the name of the pathway (or PMID) associated with the local subnetwork that has the lowest p-value from the hypergeometric test.

The publications which cite both the regulator and a target together in the same paper are listed on the web page with their Pubmed links. The papers which cocite genes in the target list are given in a spreadsheet linked to from the web page. If some of the target list are cocited, then a network graph will be displayed showing how cocitations link the genes in the target list (the regulator is also included in the graph if it has cocitations with any of the targets). There is also a link to a page with GO annotations of the target list.

The results of the LEAN analysis is available for download from the web page of each of the regulators that demonstrate pathway enrichment of their target list - a spreadsheet summarises the pathways (or PMIDs) found and an R archive file contains more detailed information on the enrichment of each of the pathways. Plots of the pathways are included on the web page, provided the graph contains less than 100 nodes.

2.1.2 Details

The database contains entries for 15496 potential regulatory genes that have at least one significant predicted target. The entry for a gene can be found through a search box on the main page. Radio buttons are used to select information in the database for a gene as a regulator, or a gene as a target. Section 2.1.4 gives details of the web page for a gene as a regulator and section 2.15 details of the web page for a gene as a target.

There are links on the main page to download a summary of the information held for each potential regulator. To appear in the summary a regulator has to have at least one significant target or at least one enriched pathway. The summary is available as a spreadsheet, `Regulators Summary spreadsheet`. There is also as an R archive file `Regulators_Details.Rdata` containing much more detailed information about these regulators. This is an R list object with slots described in the following section 2.1.3.

2.1.3 Regulators_Details.Rdata

This Rdata object is a list called regulators.details with an entry for each regulator. The name of each entry being the regulator's name. Each entry is a list with 7 slots:

1. location - regulator's genomic location
   (a) chr - chromosome
   (b) pos - chromsome position
2. self_correl - information on regulator's self aCGH/expression correlation
   (a) p-value_self_acgh_expr - fdr adjusted p-value ofthe correlation
   (b) num_datasets_self_acgh_expr - number of datasets with significant (<0.05) correlation

    (c) names_datasets_self_acgh_expr - names of those datasets

    (d) pathologies_datasets_self_acgh_expr - pathologies of samples in those datasets

3. signif_targets - information on significant targets i.e. genes with expression profiles that correlate with regulator's aCGH profiles.

    (a) num_signif_act - number of significant targets (<0.1) activated

    (b) num_signif_rep - number of significant targets (<0.1) repressed

    (c) targ_act - table of the activated targets

    (d) targ_rep - table of the repressed targets

4. reg_targ_cocites - information on regulator-target cocitations in publications

    (a) num_reg_targ_cocites - the number of targets cocited with the regulator

    (b) reg_targ_cocite_papers - the cociting papers (PUBMED ids)

5. targ_cocites - information on targets cocited together

    (a) num_targ_cocites - number of papers cociting two or more targets

    (b) percent - percentage of target list cocited with at least one other target

    (c) fdr - fdr p-value adjusted p-value of finding this percentage of the list cocited together.

6. pathway_enrich - information on any pathway enrichment ofthe target list

    (a) num_databases - number of Pathway commons databases showing any enrichment

    (b) num_centre_genes - the number of enriched 'centre' genes from the LEAN method

    (c) main_pathway - the main enriched pathway

    (d) pathway_enrich_summary - table giving summary information of the LEAN results (see entry GENENAME_lean_summary.csv below)

    (e) pathway_enrich_details - detailed information on the LEAN results (see entry GENENAME_lean_details.Rdata below)

7. go_annotations - information on any Gene Ontology (GO) enrichment

    (a) go_annotations - table of GO enrichment

2.1.4 Web page for gene as a Regulator

Selecting information on a gene as a regulator gives summary statistics for the gene, namely:

1. The number of significant (fdr<0.1) target genes activated by the regulator (positive correlation of aCGH profile and expression).

    - Significant best (stringent) - the target is significant and the regulator is the best regulator for the target using the stringent condition, that is, the regulator is the best regulator by way of lowest p-value *and* the best regulator by way of highest number of datasets in which the relationship is significant.

- Significant best (non-stringent) - the target is significant and the regulator is the best regulator for the target using the less stringent condition, that is, the regulator is the best regulator by way of lowest p-value *or* the best regulator by way of highest number of datasets in which the relationship is significant.
- Significant - the target is significant

2. The number of significant (fdr$<$0.1) targets that are repressed by the regulator (negative correlation).
   - Significant best (stringent) - the target is significant and the regulator is the best regulator for the target using the stringent condition, that is, the regulator is the best regulator by way of lowest p-value *and* the best regulator by way of highest number of datasets in which the relationship is significant.
   - Significant best (non-stringent) - the target is significant and the regulator is the best regulator for the target using the less stringent condition, that is, the regulator is the best regulator by way of lowest p-value *or* the best regulator by way of highest number of datasets in which the relationship is significant.
   - Significant - the target is significant
3. The number of regulator-target pairs which have cocitations.
4. The total number of papers which cocite at least two genes from the list of significant targets and the regulator, and the statistical significance of finding this number of cocites.
5. Pathway enrichment details, namely the databases that contain subnetworks exhibiting pathway enrichment, the total number of genes at the centre of enriched subnetworks and the main enriched pathway (or Pubmed id).
6. List of significant target genes activated by the regulator (each entry linking to the webpage giving information from the database on that gene as a target)
7. List of significant target genes repressed by the regulator (each entry linking to the webpage giving information from the database on that gene as a target)
8. Links to results files (see below)
9. Link to a zip file by which all the results files can be downloaded
10. List of papers cociting regulator-target pairs, each entry linking to the papers Pubmed page.
11. Link to a page of GO annotations of the target list.
12. A network graph showing how cocitations (if they exist) link the genes in the target list (activation and repression combined). The regulator is also included in the graph if it has cocitations with any of the targets.
13. Network graphs showing the enriched subnetworks, grouped as described in the Methods section above.

The results files

- `GENENAME_activation_sig.csv` - for activation of targets by the regulator, a spreadsheet that contains the significant predicted targets for the regulating gene (significance level Benjamini-Hochberg (BH) adjusted p-value < 0.1), for which the regulator is the 'best' regulator in the database by way of either minimum p-value and/or

significant in most datasets. This spreadsheet will not be present if the algorithm has predicted no such significant targets. The column names of the spreadsheet are:

`gene` = predicted target gene's HGNC symbol.

`chr` = predicted gene's chromosome.

`pos` = predicted gene's chromosome position.

`pv` = p-value for the correlation between the regulator and target genes based on a meta-analysis of the datasets. The correlation is between the regulating gene's aCGH and the target gene's expression. (Note: only those datasets for which the regulator gene has significant self aCGH/expression correlation are included in the meta-analysis).

`fdr` = as above but BH adjusted.

`fdr.rev` = BH adjusted p-value for the correlation between the regulator and target genes based on a meta-analysis of the datasets. Unlike `fdr` this is the 'reverse' correlation i.e. the correlation is between the target gene's aCGH and regulator gene's expression. If the regulator gene's aCGH is significantly correlated with a target gene's expression (so significant `fdr`), but the target gene's aCGH is also significantly correlated with the regulator's expression (so significant `fdr.rev`), this would suggest that a predicted relationship between the two genes is spurious, either due to coincidentally similar aCGH profiles or some form of coamplification/codeletion. (Since only inter-chromosomal relationships are being considered the latter explanation is less likely.)

`num` = number of datasets which show significant correlation (BH adjusted p-values $<$ 0.05) between regulator gene's aCGH and target gene's expression.

`best-pv` = The 'best' regulator in the database for this target based on the criterion of minimum p-value. If the regulator associated with this page is the best regulator then this entry will be blank.

`best-num` = The 'best' regulator in the database for this target based on the criterion of significance in the most number of datasets. If the regulator associated with this page is the best regulator then this entry will be blank.

`pubmed` = If the regulator associated with this page and the target are cocited then the pubmed id(s) for the paper(s) will appear here.

`pathway` = If a target is at the centre of an enriched local subnetwork the column will contain the name of the pathway (or PMID) associated with the local subnetwork that has the lowest p-value from the hypergeometric test.

These columns dealing with the results from a meta-analysis of the datasets are followed by columns giving the results for each individual dataset. The values are the BH adjusted p-values for the correlation between the regulator gene's aCGH and target gene's expression (Note: only those datasets for which the regulator gene has significant self aCGH/expression correlation are included in the analysis and shown on the spreadsheet).

- `GENENAME_activation.csv` - a spreadsheet that contains the significance of all possible targets in the database for the regulating gene; for activation of the target by the regulator (ranked by Benjamini-Hochberg (BH) adjusted p-values). So in this spreadsheet no criterion for most probable regulator and no threshold on p-value have been applied. The column names of the spreadsheet are as above.

Then there are similar files to the above for *repression* of the target by the regulator (that is, *negative* correlation between the regulator gene's aCGH and target genes' expression), the files are called `GENENAME_repression_sig.csv`, `GENENAME_repression.csv`.

- `GENENAME_target_cocitations.csv` - a spreadsheet with cocitation information. If file `GENENAME_activation_sig.csv` is present then a cocitation analysis is performed on a gene list comprising the regulator gene and all of its predicted targets. All papers that co-cite at least two of the genes in the list are recorded. The column names are:

  `CITED GENES` = the genes from the gene list cocited in this paper.

  `Number of Genes` = the number of genes cocited in this paper.

  `PUBMED ID` = the Pubmed (http://www.ncbi.nlm.nih.gov/pubmed) ID of the paper. Full information about the paper can be found by entering the Pubmed Id in the 'search' box at Pubmed.

  `PAPER TITLE` = the paper title.

  `LINK to PAPER` = the URL of the paper.

- `GENENAME_GO.html` = Gene Ontology annotations of a gene list comprising the regulator gene and all of its predicted targets. This file will be blank if no significant GO annotations were found.

- `GENENAME_lean_summary.csv` = Spreadsheet with summary information of the results of the LEAN pathway enrichment analysis. The column names are:

  `Database` = the database in which enrichment is found

  `Pathway` = the enriched pathway

  `Reg. connected?` = whether the regulator is already known to be connected to the enriched subnetwork(s)

  `p-value` = p-value from the hypergeometric test

  `Num. Centre genes` = number of enriched centres associated with pathway in the analysis

  `Num. Signif. Best Targets` = number of significant targets for which the regulator is the best regulator associated with the pathway by the analysis

- `GENENAME_lean_details.Rdata` = An R archive file with detailed information of the results of the LEAN pathway enrichment analysis. A slot for each PathwayCommons Database used in the study. For each of these slots, one slot for each enriched pathway. For each pathway there are eight slots:

  `details_list` = database, pathway, regulator connected?, p-value, number of enriched centre genes, number of nodes in graph, number of significant best targets in graph

  `enrich_cens` = the enriched centre genes

  `enrich_subnet_genes`= the genes connected to the centre genes in the enriched local subnetworks

  `graph_nodes` = all gene nodes in the graph

  `sig_best` = the significant targets of the regulator for which the regulator is the best target

sig_best_in_pway = the significant targets of the regulator for which the regulator is the best target which are associated with the pathway by the analysis

sig_best_less_stringent = the significant targets of the regulator for which the regulator is the best target using the less stringent condition which are associated with the pathway by the analysis

(Note: some spreadsheets may have too many characters in a column to load completely in some spreadsheet programs - a text editor could be used instead)

2.1.5 Web page for gene as a Target

Selecting information on a gene as a target gives summary statistics for the gene, namely:

1. Predicted regulator, for activation of the target, based on the criterion of lowest p-value, followed in brackets by the fdr (Benjamini-Hochberg (BH) adjusted p-value).
2. Predicted regulator, for activation of the target, based on the criterion of significance in the most number of datasets, followed in brackets by the fdr (Benjamini-Hochberg (BH) adjusted p-value).

   NB. the criterion of lowest p-value in 1. above is the *unadjusted* p-value, not the fdr value. The fdr value for the predicted regulator based on the criterion of lowest p-value and the fdr value for the predicted regulator based on the criterion of significance in the most number of datasets can, in some cases, be the same.
3. Number of significant predicted regulators (activation) - due to coamplification/codeletion of genes in the genomic region of the actual regulator this may be a relatively long list.
4. Predicted regulator, for repression of the target, based on the criterion of lowest p-value, followed in brackets by the fdr (Benjamini-Hochberg (BH) adjusted p-value).
5. Predicted regulator, for repression of the target, based on the criterion of significance in the most number of datasets, followed in brackets by the fdr (Benjamini-Hochberg (BH) adjusted p-value).
6. Number of significant predicted regulators (repression) - due to coamplification/codeletion of genes in the genomic region of the actual regulator this may be a relatively long list.
7. The number of the significant predicted regulators cocited in at least one paper with this target.
8. A list of these papers if any.
9. Results files - see below.

The results files

- GENENAME_activation_sig.csv - for activation of the target by regulators, a spreadsheet that contains the significant predicted regulators (significance level Benjamini-Hochberg (BH) adjusted p-value < 0.1), for which the regulator is the 'best' regulator in the database by way of either minimum p-value and/or significant in most

datasets. This spreadsheet will not be present if the algorithm has predicted no such significant targets. The column names of the spreadsheet are:

`gene` = predicted regulator gene's HGNC symbol.

`chr` = predicted gene's chromosome.

`pos` = predicted gene's chromosome position.

`pv` = p-value for the correlation between the regulator and target gene based on a meta-analysis of the datasets. The correlation is between the regulating gene's aCGH and the target gene's expression. (Note: only those datasets for which the regulator gene has significant self aCGH/expression correlation are included in the meta-analysis).

`fdr` = as above but BH adjusted.

`fdr.rev` = BH adjusted p-value for the correlation between the regulator and target genes based on a meta-analysis of the datasets. Unlike `fdr` this is the 'reverse' correlation i.e. the correlation is between the target gene's aCGH and regulator gene's expression. If the regulator gene's aCGH is significantly correlated with a target gene's expression (so significant `fdr`), but the target gene's aCGH is also significantly correlated with the regulator's expression (so significant `fdr.rev`), this would suggest that a predicted relationship between the two genes is spurious, either due to coincidentally similar aCGH profiles or some form of coamplification/codeletion. (Since only inter-chromosomal relationships are being considered the latter explanation is less likely.)

`num` = number of datasets which show significant correlation (BH adjusted p-values $<$ 0.05) between regulator gene's aCGH and target gene's expression.

`pubmed` = If the target associated with this page and the regulator are cocited then the pubmed id(s) for the paper(s) will appear here.

These columns dealing with the results from a meta-analysis of the datasets are followed by columns giving the results for each individual dataset. The values are the BH adjusted p-values for the correlation between the regulator gene's aCGH and target gene's expression (Note: only those datasets for which the regulator gene has significant self aCGH/expression correlation are included in the analysis and shown on the spreadsheet).

- `GENENAME_activation.csv` - a spreadsheet that contains the significance of all possible regulators in the database for the target gene; for activation of the target by the regulator (ranked by Benjamini-Hochberg (BH) adjusted p-values). The ranked list is thresholded at a p-value < 0.33 for practical purposes. If there are no entries with a p-value < 0.33 then the top 100 entries are given. The column names of the spreadsheet are as above.

  Then there are similar files to the above for *repression* of the target by the regulator (that is, *negative* correlation between the regulator gene's aCGH and target genes' expression), the files are called `GENENAME_repression_sig.csv`, `GENENAME_repression.csv`.

2.1.6 Interpreting the results files

Consistent coamplification or codeletion of neighbouring potential regulators coupled with the inherent noise in the data can lead to ambiguity in the database as to which of the regulators is actually regulating a particular target gene. So the results files contain columns

giving the 'best' regulator in the database for each target based on the criterion of minimum p-value and the 'best' regulator in the database based on the criterion of significance in the most number of datasets. In addition each gene has a webpage containing information in the database on the gene *as a target*. This page contains spreadsheets giving all the significant regulators of the target gene in question.

NB. A note on cross-referencing. For any regulator and a predicted target, details of the relationship will be found on two spreadsheets. Firstly the regulator will have a spreadsheet of predicted targets in which the target will appear. Secondly the target will have a spreadsheet of predicted regulators in which the regulator will appear. The *unadjusted* p-value of the relationship between the regulator and target in the two spreadsheets will of course be the same. However the two fdr values (the Benjamini-Hochberg adjusted p-value) will in general be different since the multiple correction has been performed on two different lists of p-values.

References

[1] Parris TZ, Danielsson A, Nemes S, Kovcs A, Delle U, Fallenius G, et al. Clinical Implications of Gene Dosage and Gene Expression Patterns in Diploid Breast Carcinoma. Clinical Cancer Research. 2010;16:3860–3874.

[2] Crowder RJ, Phommaly C, Tao Y, Hoog J, Luo J, et al. PIK3CA and PIK3CB Inhibition Produce Synthetic Lethality when Combined with Estrogen Deprivation in Estrogen ReceptorPositive Breast Cancer. Cancer Research. 2009;69:3955–3962.

[3] Sircoulomb F, Bekhouche I, Finetti P, Adélaïde J, Hamida AB, et al. Genome profiling of ERBB2-amplified breast cancers. BMC Cancer. 2010;10:539.

[4] Myllykangas S, Junnila S, Kokkola A, Autio R, Scheinin I, Kiviluoto T, et al. Integrated gene copy number and expression microarray analysis of gastric cancer highlights potential target genes. Int J Cancer. 2008;123:817–825.

[5] Junnila S, Kokkola A, Karjalainen-Lindsberg ML, Puolakkainen P, Monni O. Genome-wide gene copy number and expression analysis of primary gastric tumors and gastric cancer cell lines. BMC Cancer. 2010;10:73.

[6] Chitale D, Gong Y, Taylor BS, Broderick S, Brennan C, et al. An integrated genomic analysis of lung cancer reveals loss of DUSP4 in EGFR-mutant tumors. Oncogene. 2009;6:2773–2783.

[7] Goh XY, Rees JR, Paterson AL, Chin SF, Marioni JC, et al. Integrative analysis of array-comparative genomic hybridisation and matched gene expression profiling data reveals novel genes with prognostic significance in oesophageal adenocarcinoma. Gut. 2011;60:1317–1326.

[8] Zhou Y, Zhang Q, Stephens O, Heuck CJ, Tian E, et al. Prediction of cytogenetic abnormalities with gene expression profiles. Blood. 2012;119:e148–e150.

[9] Shain AH, Giacomini CP, Matsukuma K, Karikari CA, Bashyam MD, Hidalgo M, et al. Convergent structural alterations define SWItch/Sucrose NonFermentable (SWI/SNF) chromatin remodeler as a central tumor suppressive complex in pancreatic cancer. Proc Nat Acad Sci. 2012;109:252–259.

[10] Vainio P, Wolf M, Edgren H, He T, Kohonen P, Mpindi JP, et al. Integrative Genomic, Transcriptomic, and RNAi Analysis Indicates a Potential Oncogenic Role for FAM110B in Castration-Resistant Prostate Cancer. The Prostate. 2012;72:789–802.

[11] Bott M, Brevet M, Taylor BS, Shimizu S, Ito1and T, et al. The nuclear deubiquitinase BAP1 is commonly inactivated by somatic mutations and 3p21.1 losses in malignant pleural mesothelioma. Nature Genetics. 2011;43:668–674.

[12] Bekhouche I, Finetti P, Adelaide J, Ferrari A, Tarpin C, Charafe-Jauffret E, et al. High-Resolution Comparative Genomic Hybridization of Inflammatory Breast Cancer and Identification of Candidate Genes. PlosOne. 2011;6:e16950.

[13] Chapman MA, Lawrence MS, Keats JJ, Cibulskis K, Sougnez C, et al. Initial genome sequencing and analysis of multiple myeloma. Nature. 2011;471:467–472.

[14] Ooi WF, Re A, Sidarovich V, Canella V, Arseni N, Adami V, et al. Segmental Chromosome Aberrations Converge on Overexpression of Mitotic Spindle Regulatory Genes in High-Risk Neuroblastoma. Genes, Chromosomes and Cancer. 2012;51:545–556.

[15] Braggio E, Keats JJ, Leleu X, Van Wier S, Jimenez-Zepeda VH, et al. Identification of Copy Number Abnormalities and Inactivating Mutations in Two Negative Regulators of Nuclear Factor-κB Signaling Pathways in Waldenströms Macroglobulinemia. Cancer Research. 2009;69:3579–3588.

[16] Jönsson G, Staaf J, Vallon-Christersson J, Ringnér M, Holm K, et al. Genomic subtypes of breast cancer identified by array-comparative genomic hybridization display distinct molecular and clinical characteristics. Breast Cancer Research. 2010;12:R42.

[17] Muranen TA, Greco D, Fagerholm R, Kilpivaara O, Kämpjärvi K, Aittomäki K, et al. Breast tumors from CHEK2 1100delC-mutation carriers: genomic landscape and clinical implications. Breast Cancer Research. 2011;13:R90.

[18] Lindgren D, Frigyesi A, Gudjonsson S, Sjödahl G, Hallden C, et al. Combined Gene Expression and Genomic Profiling Define Two Intrinsic Molecular Subtypes of Urothelial Carcinoma and Gene Signatures for Molecular Grading and Outcome. Cancer Research. 2010;70:3463–3472.

[19] Beck AH, Lee CH, Witten DM, Gleason BC, Edris B, et al. Discovery of molecular subtypes in leiomyosarcoma through integrative molecular profiling. Oncogene. 2010;29:845–862.

[20] Toedt G, Barbus S, Wolter M, Felsberg J, Tews B, et al. Molecular signatures classify astrocytic gliomas by IDH1 mutation status. International Journal of Cancer. 2011;128:1095–1103.

[21] Ellis MJ, Ding L, Shen D, Luo J, Suman VJ, et al. Whole-genome analysis informs breast cancer response to aromatase inhibition. Nature. 2012;486:353–360.

[22] Grasso CS, Wu YM, Robinson DR, Cao X, Dhanasekaran SM, et al. The mutational landscape of lethal castration-resistant prostate cancer. Nature. 2012;487:239–243.

[23] Lenz G, Wright GW, Emre NC, Kohlhammer H, Dave SS, Davis RE, et al. Molecular subtypes of diffuse large B-cell lymphoma arise by distinct genetic pathways. Proc Natl Acad Sci U S A. 2008;105:13520–13525.

[24] Lindgren D, Sjödahl G, Lauss M, Staaf J, Chebil G, et al. Integrated Genomic and Gene Expression Profiling Identifies Two Major Genomic Circuits in Urothelial Carcinoma. PlosOne. 2012;7:e38863.

[25] Micci F, Panagopoulos I, Haugom L, Dahlback HS, Pretorius ME, Davidson B, et al. Genomic aberration patterns and expression profiles of squamous cell carcinomas of the vulva. Genes Chromosomes Cancer. 2013;52:551–563.

[26] Taylor BS, Schultz N, Hieronymus H, Gopalan A, Xiao Y, et al. Integrative Genomic Profiling of Human Prostate Cancer. Cancer Cell. 2010;18:11–22.

[27] Coco S, Theissen J, Scaruffi P, Stigliani S, Moretti S, et al. Age-dependent accumulation of genomic aberrations and deregulation of cell cycle and telomerase genes in metastatic neuroblastoma. International Journal of Cancer. 2012;131:1591–1600.

[28] Medina PP, Castillo SD, Blanco S, Sanz-Garcia M, Largo C, et al. The SRY-HMG box gene, SOX4, is a target of gene amplification at chromosome 6p in lung cancer. Human Molecular Genetics. 2009;18:1343–1352.

[29] Przybyl J, Sciot R, Wozniak A, Schffski P, Vanspauwen V, Samson I, et al. Metastatic potential is determined early in synovial sarcoma development and reflected by tumor molecular features. The International Journal of Biochemistry & Cell Biology. 2014;53(0):505 – 513.

[30] Huang RY, Chen GB, Matsumura N, Lai HC, Mori S, Li J, et al. Histotype-specific copy-number alterations in ovarian cancer. BMC Medical Genomics. 2012;5(1):47. doi:10.1186/1755-8794-5-47.

[31] Hook KE, Garza SJ, Lira ME, Ching KA, Lee NV, Cao J, et al. An Integrated Genomic Approach to Identify Predictive Biomarkers of Response to the Aurora Kinase Inhibitor PF-03814735. Molecular Cancer Therapeutics. 2012;11(3):710–719. doi:10.1158/1535-7163.MCT-11-0184.

[32] Monti S, Chapuy B, Takeyama K, et al. Integrative Analysis Reveals an Outcome-associated and Targetable Pattern of p53 and Cell Cycle Deregulation in Diffuse Large B-cell Lymphoma. Cancer cell. 2012;22:359–372.

[33] Ross-Adams H, Lamb A, Dunning M, et al. Integration of copy number and transcriptomics provides risk stratification in prostate cancer: A discovery and validation cohort study. EBioMedicine. 2015;2:1133 – 1144.

[34] Ramakrishna M, Williams LH, Boyle SE, Bearfoot JL, Sridhar A, Speed TP, et al. Identification of Candidate Growth Promoting Genes in Ovarian Cancer through Integrated Copy Number and Expression Analysis. PLOS ONE. 2010;5(4):1–12. doi:10.1371/journal.pone.0009983.

[35] Guarneri V, Dieci MV, Frassoldati A, Maiorana A, Ficarra G, Bettelli S, et al. Prospective Biomarker Analysis of the Randomized CHER-LOB Study Evaluating the Dual Anti-HER2 Treatment With Trastuzumab and Lapatinib Plus Chemotherapy as Neoadjuvant Therapy for HER2-Positive Breast Cancer. The Oncologist. 2015;20(9):1001–1010. doi:10.1634/theoncologist.2015-0138.

[36] Wilkerson MD, Yin X, Walter V, Zhao N, Cabanski CR, Hayward MC, et al. Differential Pathogenesis of Lung Adenocarcinoma Subtypes Involving Sequence Mutations, Copy Number, Chromosomal Instability, and Methylation. PLOS ONE. 2012;7(5):1–13. doi:10.1371/journal.pone.0036530.

[37] Donahue TR, Tran LM, Hill R, Li Y, Kovochich A, Calvopina JH, et al. Integrative Survival-Based Molecular Profiling of Human Pancreatic Cancer. Clinical Cancer Research. 2012;18(5):1352–1363. doi:10.1158/1078-0432.CCR-11-1539.

[38] Kuijjer ML, Rydbeck H, Kresse SH, Buddingh EP, Lid AB, Roelofs H, et al. Identification of osteosarcoma driver genes by integrative analysis of copy number and gene expression data. Genes, Chromosomes and Cancer. 2012;51(7):696–706. doi:10.1002/gcc.21956.

[39] Paugh BS, Broniscer A, Qu C, Miller CP, Zhang J, Tatevossian RG, et al. Genome-Wide Analyses Identify Recurrent Amplifications of Receptor Tyrosine Kinases and Cell-Cycle Regulatory Genes in Diffuse Intrinsic Pontine Glioma. Journal of Clinical Oncology. 2011;29(30):3999–4006. doi:10.1200/JCO.2011.35.5677.

[40] Weigelt B, Ng C, Shen R, et al. Metastatic breast carcinomas display genomic and transcriptomic heterogeneity. Modern pathology. 2015;28(3):340–351.

[41] Newton R, Wernisch L. Investigating inter-chromosomal regulatory relationships through a comprehensive meta-analysis of matched copy number and transcriptomics data sets. BMC Genomics. 2015;16(1):967.

[42] HUGO Gene Nomenclature Committe (HGNC);. Available from: `http://www.genenames.org/cgi-bin/hgnc_downloads`.

[43] Newton R, Wernisch L. A Meta-Analysis of Multiple Matched Copy Number and Transcriptomics Data Sets for Inferring Gene Regulatory Relationships. PLoS ONE. 2014;9(8):e105522.

[44] Goh XY, Newton R, Wernisch L, Fitzgerald R. Testing the Utility of an Integrated Analysis of Copy Number and Transcriptomics Datasets for Inferring Gene Regulatory Relationships. PLoSONE. 2013;8:e63780.

[45] R Core Team. R: A Language and Environment for Statistical Computing; 2018. Available from: `http://www.R-project.org/`.

[46] Dudbridge F, Koeleman BPC. Rank truncated product of P-values, with application to genomewide association scans. Genetic Epidemiology. 2003;25(4):360–366. doi:http://dx.doi.org/10.1002/gepi.10264.

[47] Gentleman RC, Carey VJ, Bates DM, and others. Bioconductor: Open software development for computational biology and bioinformatics. Genome Biology. 2004;5:R80.

[48] Carlson M. org.Hs.eg.db: Genome wide annotation for Human;.

[49] Ding B, Gentleman R. CoCiteStats: Different test statistics based on co-citation.;.

[50] Ashburner M, Ball C, Blake J, Botstein D, Butler H, et al. Gene Ontology: tool for the unification of biology. Nature Genetics. 2000;25:25–29.

[51] Falcon S, Gentleman R. Using GOstats to test gene lists for GO term association. Bioinformatics. 2007;23(2):257–8.

[52] Carlson M. GO.db: A set of annotation maps describing the entire Gene Ontology;.

[53] Csardi G, Nepusz T. The igraph software package for complex network research. InterJournal. 2006;Complex Systems:1695.

[54] Carbon S, Ireland A, Mungall C, et al. AmiGO: online access to ontology and annotation data. Bioinformatics. 2009;25:2588–2589.
