# Supplementary material for: A meta-analysis of multiple matched aCGH/expression cancer datasets reveals regulatory relationships and pathway enrichment of potential oncogenes
Source: PLoS One. 2019 Jul 23;14(7):e0213221. doi: 10.1371/journal.pone.0213221 (PMC6650054; doi:10.1371/journal.pone.0213221)
Supplement: S4 File — (PDF) [file pone.0213221.s004.pdf]

S4 File: Supplementary tables

...

"A meta-analysis of multiple matched aCGH/expression cancer datasets reveals regulatory relationships and pathway enrichment of potential oncogenes"

...

Richard Newton & Lorenz Wernisch

July 10, 2019

List of Tables

A Main enriched pathways and associated regulators 3
B Enriched pathways and associated regulators 15
C Most disrupted pathways. 77
D Regulators, their number of targets, number of datasets with self aCGH/expression correlation and main pathway. 101

Table A. Main enriched pathways and associated regulators

Main Pathway	Regulators
pre-mRNA splicing	YTHDC1, PURA, TMEM259, ASNSD1, SMOC2, PCDHB9, ZNF446, EMILIN1, NIPAL4, TCERG1, SF3B4, NPIPB15, PROCR
Amplification of signal from unattached kinetochores via a MAD2 inhibitory signal;Mitotic Prometaphase;RHO GTPases Activate Formins;Resolution of Sister Chromatid Cohesion;Separation of Sister Chromatids	SLC27A3, NAALADL2, USP13, GUCA2B, LINC01587, AURKC, TBCK, LSM11, SOX14, SGCZ, KCNMB3, TSPAN7
RNA polymerase II transcribes snRNA genes	PITX1, POGZ, ELL, ELL2, ELL3, SNAPC2

Table A (continued)

Main Pathway	Regulators
Activated NTRK2 signals through PI3K;Activation of PKB;Antigen activates B Cell Receptor (BCR) leading to generation of second messengers;CD28 dependent PI3K/Akt signaling;CD28 dependent Vav1 pathway;Constitutive Signaling by Aberrant PI3K in Cancer;Constitutive Signaling by EGFRvIII;Constitutive Signaling by Ligand-Responsive EGFR Cancer Variants;DAP12 signaling;Downstream TCR signaling;Downstream signal transduction;FCERI mediated Ca+2 mobilization;FCERI mediated MAPK activation;G beta:gamma signalling through PI3Kgamma;GAB1 signalosome;GPVI-mediated activation cascade;Inhibition of TSC complex formation by PKB;MET activates PI3K/AKT signaling;Negative regulation of the PI3K/AKT network;PDE3B signalling;PI Metabolism;PI-3K cascade:FGFR1;PI-3K cascade:FGFR2;PI-3K cascade:FGFR3;PI-3K cascade:FGFR4;PI3K Cascade;PI3K events in ERBB2 signaling;PI3K events in ERBB4 signaling;PI3K/AKT Signaling;PI3K/AKT activation;PI5P, PP2A and IER3 Regulate PI3K/AKT Signaling;PTEN Loss of Function in Cancer;Phospholipase C-mediated cascade: FGFR1;Phospholipase C-mediated cascade; FGFR2;Phospholipase C-mediated cascade; FGFR3;Phospholipase C-mediated cascade; FGFR4;RHO GTPases Activate WASPs and WAVEs;RHO GTPases activate PKNs;RIPK1-mediated regulated necrosis;Regulation of TP53 Degradation;Regulation of actin dynamics for phagocytic cup formation;Role of LAT2/NTAL/LAB on calcium mobilization;Role of phospholipids in phagocytosis;Signaling by FGFR1 in disease;Signaling by FGFR2 in disease;Signaling by FGFR3 fusions in cancer;Signaling by FGFR3 point mutants in cancer;Signaling by FGFR4 in disease;Signaling by SCF-KIT;Signaling by cytosolic FGFR1 fusion mutants;Synthesis of PIPs at the plasma membrane;VEGFA-VEGFR2 Pathway;VEGFR2 mediated vascular permeability	ABCC12, PIK3R1, IGSF9, MEF2C, PBXIP1
Formation of the beta-catenin:TCF transactivating complex	CPEB4, MYC, PYGO2, BCL9, WWOX
Integrin signalling pathway	DOCK1, PTPRE, ZEB1, CX3CL1, FPR2
Major pathway of rRNA processing in the nucleolus and cytosol;rRNA modification in the nucleus and cytosol	GKN1, CDX1, CFHR4, SEC24D, KCNK4
SRP-dependent cotranslational protein targeting to membrane	SSB, RPL13A, RPRM, CLDND2, KCNN3
Eukaryotic Translation Termination;Formation of a pool of free 40S subunits;GTP hydrolysis and joining of the 60S ribosomal subunit;Nonsense Mediated Decay (NMD) enhanced by the Exon Junction Complex (EJC);Nonsense Mediated Decay (NMD) independent of the Exon Junction Complex (EJC);Peptide chain elongation;Regulation of expression of SLITs and ROBOs;SRP-dependent cotranslational protein targeting to membrane;Selenocysteine synthesis;Viral mRNA Translation	FCRL5, DENND1C, PRKACA, KDELR1
Major pathway of rRNA processing in the nucleolus and cytosol	FAM212B, NIP7, PDE4C, RIPK3

Table A (continued)

Main Pathway	Regulators
Activation of AMPA receptors;Activation of Ca-permeable Kainate Receptor;Activation of CaMK IV;Activation of NF-kappaB in B cells;Activation of the phototransduction cascade;Amyloid fiber formation;Antigen activates B Cell Receptor (BCR) leading to generation of second messengers;CLEC7A (Dectin-1) induces NFAT activation;CREB phosphorylation through the activation of Adenylate Cyclase;CREB phosphorylation through the activation of CaMKII;Ca2+ pathway;CaM pathway;CaMK IV-mediated phosphorylation of CREB;Calcineurin activates NFAT;Calmodulin induced events;Cam-PDE 1 activation;Chromatin modifying enzymes;DAG and IP3 signaling;DARPP-32 events;Defective SLC24A1 causes congenital stationary night blindness 1D (CSNB1D);Defective SLC24A4 causes hypomineralized amelogenesis imperfecta (AI);Degradation of the extracellular matrix;Deregulated CDK5 triggers multiple neurodegenerative pathways in Alzheimer's disease models;Effects of PIP2 hydrolysis;Elevation of cytosolic Ca2+ levels;FCERI mediated Ca+2 mobilization;FCERI mediated NF-kB activation;Glutamate Neurotransmitter Release Cycle;Glycerophospholipid biosynthesis;Glycogen breakdown (glycogenolysis);Glycosphingolipid metabolism;Highly calcium permeable nicotinic acetylcholine receptors;Highly calcium permeable postsynaptic nicotinic acetylcholine receptors;Inactivation, recovery and regulation of the phototransduction cascade;Ion homeostasis;Ion transport by P-type ATPases;Mitochondrial calcium ion transport;Neurotransmitter receptors and postsynaptic signal transmission;PKA activation;PLC beta mediated events;Phase 0 - rapid depolarisation;Phase 2 - plateau phase;Presynaptic depolarization and calcium channel opening;RHO GTPases activate PAKs;Rap1 signalling;Ras activation upon Ca2+ influx through NMDA receptor;Reduction of cytosolic Ca++ levels;Regulation of insulin secretion;Response to elevated platelet cytosolic Ca2+;Role of phospholipids in phagocytosis;Smooth Muscle Contraction;Sodium/Calcium exchangers;Sperm Motility And Taxes;Stimuli-sensing channels;Striated Muscle Contraction;Synthesis of Leukotrienes (LT) and Eoxins (EX);TRP channels;Tetrahydrobiopterin (BH4) synthesis, recycling, salvage and regulation;Translocation of GLUT4 to the plasma membrane;Unblocking of NMDA receptor, glutamate binding and activation;Uptake and function of anthrax toxins;VEGFR2 mediated cell proliferation;VEGFR2 mediated vascular permeability;eNOS activation;phospho-PLA2 pathway	MCMDC2, PEBP4, ETV7
Activation of anterior HOX genes in hindbrain development during early embryogenesis	RAB3A, NCOA6, VHL
Anchoring of the basal body to the plasma membrane	TMEM37, CERK, PDE8B
Antigen processing: Ubiquitination and Proteasome degradation	ZCCHC9, LRSAM1, MRPL54
AURKA Activation by TPX2;Anchoring of the basal body to the plasma membrane;Loss of Nlp from mitotic centrosomes;Loss of proteins required for interphase microtubule organizationfrom the centrosome;Recruitment of NuMA to mitotic centrosomes;Recruitment of mitotic centrosome proteins and complexes;Regulation of PLK1 Activity at G2/M Transition	KRTAP15-1, ADH1C, ATG10
Estrogen-dependent gene expression	PIEZO1, CHD1, IQGAP2

Table A (continued)

Main Pathway	Regulators
Eukaryotic Translation Termination;Formation of a pool of free 40S subunits;GTP hydrolysis and joining of the 60S ribosomal subunit;L13a-mediated translational silencing of Ceruloplasmin expression;Major pathway of rRNA processing in the nucleolus and cytosol;Nonsense Mediated Decay (NMD) enhanced by the Exon Junction Complex (EJC);Nonsense Mediated Decay (NMD) independent of the Exon Junction Complex (EJC);Peptide chain elongation;Regulation of expression of SLITs and ROBOs;SRP-dependent cotranslational protein targeting to membrane;Selenocysteine synthesis;Viral mRNA Translation	STARD4, KLK6, DUOX1
HDACs deacetylate histones	TBL1XR1, HDAC10, WFIKKN1
Mitochondrial translation elongation;Mitochondrial translation initiation;Mitochondrial translation termination	ADAMTSL4, SLC39A13, FIGF
Nonsense Mediated Decay (NMD) enhanced by the Exon Junction Complex (EJC)	RPS23, RPS4X, PAX6
PKMTs methylate histone lysines	DQX1, MECOM, ASH1L
snRNP Assembly	GEMIN7, GEMIN8, GCG
Transcriptional regulation by small RNAs	GIMAP2, AGO2, HIST2H2BE
Acetylcholine regulates insulin secretion;Activation of NF-kappaB in B cells;Activation of RAS in B cells;Antigen activates B Cell Receptor (BCR) leading to generation of second messengers;Arachidonate production from DAG;Ca2+ pathway;EGFR Transactivation by Gastrin;Effects of PIP2 hydrolysis;Elevation of cytosolic Ca2+ levels;Fatty Acids bound to GPR40 (FFAR1) regulate insulin secretion;G alpha (q) signalling events;G beta:gamma signalling through PLC beta;GPVI-mediated activation cascade;Rap1 signalling;Response to elevated platelet cytosolic Ca2+;Syndecan interactions;Synthesis of IP3 and IP4 in the cytosol	INTS8, TERF2IP
Antigen activates B Cell Receptor (BCR) leading to generation of second messengers;CD22 mediated BCR regulation	ARHGEF4, ATXN7L2
APC-Cdc20 mediated degradation of Nek2A;APC/C:Cdc20 mediated degradation of Cyclin B;APC/C:Cdc20 mediated degradation of Securin;APC/C:Cdc20 mediated degradation of mitotic proteins;APC/C:Cdh1 mediated degradation of Cdc20 and other APC/C:Cdh1 targeted proteins in late mitosis/early G1;Antigen processing: Ubiquitination and Proteasome degradation;Autodegradation of Cdh1 by Cdh1:APC/C;CDK-mediated phosphorylation and removal of Cdc6;Cdc20:Phospho-APC/C mediated degradation of Cyclin A;Conversion from APC/C:Cdc20 to APC/C:Cdh1 in late anaphase;Inactivation of APC/C via direct inhibition of the APC/C complex;Phosphorylation of the APC/C;Regulation of APC/C activators between G1/S and early anaphase;Senescence-Associated Secretory Phenotype (SASP);Separation of Sister Chromatids	SLC25A2, NQO2
COPI-dependent Golgi-to-ER retrograde traffic	WDR49, KDELR2
Deposition of new CENPA-containing nucleosomes at the centromere	SRPX, S100A14
E2F transcription factor network	CXCL14, CES2

Table A (continued)

Main Pathway	Regulators
Fanconi Anemia Pathway	KLK11, UBE2T
Formation of a pool of free 40S subunits;Formation of the ternary complex, and subsequently, the 43S complex;GTP hydrolysis and joining of the 60S ribosomal subunit;L13a-mediated translational silencing of Ceruloplasmin expression;Ribosomal scanning and start codon recognition;Translation initiation complex formation	MUC5B, VPS8
Gap-filling DNA repair synthesis and ligation in TC-NER;HDR through MMEJ (alt-NHEJ);Processing of DNA double-strand break ends	PAM, RNF139
Gap-filling DNA repair synthesis and ligation in TC-NER;Processing of DNA double-strand break ends	HOXD9, IL6R
HDR through Homologous Recombination (HRR)	RAD51D, POLK
HIV Transcription Initiation;RNA Polymerase II HIV Promoter Escape;RNA Polymerase II Pre-transcription Events;RNA Polymerase II Promoter Escape;RNA Polymerase II Transcription Initiation;RNA Polymerase II Transcription Initiation And Promoter Clearance;RNA Polymerase II Transcription Pre-Initiation And Promoter Opening;Transcription of the HIV genome	SLC25A37, FBLN1
Neddylation	KLHL3, BCAN
NEP/NS2 Interacts with the Cellular Export Machinery;NS1 Mediated Effects on Host Pathways;Nuclear Pore Complex (NPC) Disassembly;Nuclear import of Rev protein;Regulation of Glucokinase by Glucokinase Regulatory Protein;Regulation of HSF1-mediated heat shock response;Rev-mediated nuclear export of HIV RNA;SUMOylation of DNA damage response and repair proteins;SUMOylation of DNA replication proteins;SUMOylation of RNA binding proteins;SUMOylation of chromatin organization proteins;Transcriptional regulation by small RNAs;Transport of Mature mRNA Derived from an Intronless Transcript;Transport of Mature mRNA derived from an Intron-Containing Transcript;Transport of Ribonucleoproteins into the Host Nucleus;Transport of the SLBP Dependant Mature mRNA;Transport of the SLBP independent Mature mRNA;Viral Messenger RNA Synthesis;Vpr-mediated nuclear import of PICs;snRNP Assembly;tRNA processing in the nucleus	SLC30A5, NR3C2
Rho GTPase cycle	ARHGAP30, LRRIQ4
RUNX1 regulates genes involved in megakaryocyte differentiation and platelet function	KMT2E, ANKRD46
TNFR2 non-canonical NF-kB pathway	POLD3, TNNT2
Ub-specific processing proteases	AR, ADRB2
2-oxobutanoate degradation I	KCNK7

Table A (continued)

Main Pathway	Regulators
ABC-family proteins mediated transport;ATP sensitive Potassium channels;Activation of G protein gated Potassium channels;Ca activated K+ channels;Classical Kir channels;Defective ABCC8 can cause hypoglycemias and hyperglycemias;HCN channels;Inhibition of voltage gated Ca2+ channels via Gbeta/gamma subunits;Ion homeostasis;Ion transport by P-type ATPases;Phase 1 - inactivation of fast Na+ channels;Phase 2 - plateau phase;Phase 3 - rapid repolarisation;Phase 4 - resting membrane potential;Potassium transport channels;Sperm Motility And Taxes;TWIK related potassium channel (TREK);TWIK-related alkaline pH activated K+ channel (TALK);TWIK-related spinal cord K+ channel (TRESK);TWIK-releated acid-sensitive K+ channel (TASK);Tandem of pore domain in a weak inwardly rectifying K+ channels (TWIK);Tandem pore domain halothane-inhibited K+ channel (THIK);The NLRP3 inflammasome;Voltage gated Potassium channels	PGK2
Abortive elongation of HIV-1 transcript in the absence of Tat;Formation of HIV elongation complex in the absence of HIV Tat;Formation of HIV-1 elongation complex containing HIV-1 Tat;Formation of RNA Pol II elongation complex;Formation of the Early Elongation Complex;Formation of the HIV-1 Early Elongation Complex;HIV elongation arrest and recovery;Pausing and recovery of HIV elongation;Pausing and recovery of Tat-mediated HIV elongation;RNA Polymerase II Pre-transcription Events;RNA Polymerase II Transcription Elongation;TP53 Regulates Transcription of DNA Repair Genes;Tat-mediated HIV elongation arrest and recovery;Tat-mediated elongation of the HIV-1 transcript	NTRK1

Table A (continued)

Main Pathway	Regulators
Acetylcholine regulates insulin secretion;Activated NTRK2 signals through PI3K;Antigen activates B Cell Receptor (BCR) leading to generation of second messengers;CD28 dependent PI3K/Akt signaling;CLEC7A (Dectin-1) signaling;Ca2+ pathway;Cargo recognition for clathrin-mediated endocytosis;Clathrin-mediated endocytosis;Constitutive Signaling by AKT1 E17K in Cancer;Constitutive Signaling by Aberrant PI3K in Cancer;Constitutive Signaling by EGFRvIII;Constitutive Signaling by Ligand-Responsive EGFR Cancer Variants;DAG and IP3 signaling;DAP12 signaling;DCC mediated attractive signaling;Downstream TCR signaling;Downstream signal transduction;EGFR downregulation;EPHB-mediated forward signaling;ER-Phagosome pathway;FCERI mediated Ca+2 mobilization;Fatty Acids bound to GPR40 (FFAR1) regulate insulin secretion;G alpha (q) signalling events;G beta:gamma signalling through PI3Kgamma;G beta:gamma signalling through PLC beta;GAB1 signalosome;GPVI-mediated activation cascade;GRB2:SOS provides linkage to MAPK signaling for Integrins;Generation of second messenger molecules;IRAK4 deficiency (TLR2/4);Integrin alphaIIb beta3 signaling;MET activates PI3K/AKT signaling;MyD88 deficiency (TLR2/4);MyD88:Mal cascade initiated on plasma membrane;Negative regulation of the PI3K/AKT network;Netrin-1 signaling;PI Metabolism;PI-3K cascade:FGFR1;PI-3K cascade:FGFR2;PI-3K cascade:FGFR3;PI-3K cascade:FGFR4;PI3K Cascade;PI3K events in ERBB2 signaling;PI3K events in ERBB4 signaling;PI3K/AKT Signaling;PI3K/AKT activation;PI5P, PP2A and IER3 Regulate PI3K/AKT Signaling;PLC beta mediated events;RHO GTPases Activate WASPs and WAVEs;RIPK1-mediated regulated necrosis;Recycling pathway of L1;Regulation of actin dynamics for phagocytic cup formation;Role of LAT2/NTAL/LAB on calcium mobilization;Role of phospholipids in phagocytosis;Role of second messengers in netrin-1 signaling;Signaling by FGFR1 in disease;Signaling by FGFR2 in disease;Signaling by FGFR3 fusions in cancer;Signaling by FGFR3 point mutants in cancer;Signaling by FGFR4 in disease;Signaling by SCF-KIT;Signaling by cytosolic FGFR1 fusion mutants;Syndecan interactions;Synthesis of IP3 and IP4 in the cytosol;Synthesis of PIPs at the plasma membrane;VEGFA-VEGFR2 Pathway;VEGFR2 mediated cell proliferation;WNT mediated activation of DVL;p130Cas linkage to MAPK signaling for integrins	PIP5K1A
Activated NOTCH1 Transmits Signal to the Nucleus	FAM83E
Activation of anterior HOX genes in hindbrain development during early embryogenesis;Deposition of new CENPA-containing nucleosomes at the centromere;PRC2 methylates histones and DNA	LYL1
Activation of anterior HOX genes in hindbrain development during early embryogenesis;RUNX1 regulates genes involved in megakaryocyte differentiation and platelet function	RBBP5
Activation of E2F1 target genes at G1/S	DHFR
Activation of E2F1 target genes at G1/S;Cyclin E associated events during G1/S transition;G0 and Early G1;Polo-like kinase mediated events;Transcription of E2F targets under negative control by DREAM complex	SEC62
Activation of RAC1	GRHL2

Table A (continued)

Main Pathway	Regulators
Activation of rRNA Expression by ERCC6 (CSB) and EHMT2 (G9a);HDACs deacetylate histones;SIRT1 negatively regulates rRNA expression	PARP10
adenine and adenosine salvage III;adenosine nucleotides degradation II;guanine and guanosine salvage I;guanosine nucleotides degradation III;purine nucleotides degradation II (aerobic);purine ribonucleosides degradation to ribose-1-phosphate;urate biosynthesis/inosine 5'-phosphate degradation;xanthine and xanthosine salvage	PCK1
ALK1 signaling events;Angiopoietin receptor Tie2-mediated signaling;Arf6 downstream pathway;CDC42 signaling events;CXCR3-mediated signaling events;Cellular roles of Anthrax toxin;EPHB forward signaling;Endothelins;ErbB1 downstream signaling;ErbB4 signaling events;FGF signaling pathway;GMCSF-mediated signaling events;IFN-gamma pathway;Integrins in angiogenesis;Netrin-mediated signaling events;Neurotrophic factor-mediated Trk receptor signaling;Nongenotropic Androgen signaling;Osteopontin-mediated events;PDGFR-beta signaling pathway;Regulation of Telomerase;S1P1 pathway;S1P2 pathway;S1P3 pathway;S1P4 pathway;Signaling events mediated by PRL;Signaling events mediated by VEGFR1 and VEGFR2;Signaling events regulated by Ret tyrosine kinase;Syndecan-1-mediated signaling events;Syndecan-2-mediated signaling events;TRAIL signaling pathway;Trk receptor signaling mediated by the MAPK pathway;VEGFR1 specific signals;VEGFR3 signaling in lymphatic endothelium;mTOR signaling pathway	TNFSF10
Alpha4 beta1 integrin signaling events	JAML
Antigen activates B Cell Receptor (BCR) leading to generation of second messengers;Ca2+ pathway;G beta:gamma signalling through PLC beta;GPVI-mediated activation cascade;Synthesis of IP2, IP, and Ins in the cytosol	GALE
Antigen processing: Ubiquitination and Proteasome degradation;Interconversion of nucleotide di- and triphosphates	ATG7
APC/C:Cdc20 mediated degradation of Securin;APC/C:Cdh1 mediated degradation of Cdc20 and other APC/C:Cdh1 targeted proteins in late mitosis/early G1;Autodegradation of Cdh1 by Cdh1:APC/C;Cdc20:Phospho-APC/C mediated degradation of Cyclin A;Separation of Sister Chromatids	FCRL2
Arf1 pathway;BCR signaling pathway;CXCR4-mediated signaling events;Class I PI3K signaling events;E-cadherin signaling in keratinocytes;EPO signaling pathway;Endothelins;ErbB1 downstream signaling;FGF signaling pathway;Fc-epsilon receptor I signaling in mast cells;IL2 signaling events mediated by PI3K;IL8- and CXCR1-mediated signaling events;IL8- and CXCR2-mediated signaling events;LPA receptor mediated events;LPA4-mediated signaling events;Nephrin/Neph1 signaling in the kidney podocyte;Netrin-mediated signaling events;Nongenotropic Androgen signaling;PAR1-mediated thrombin signaling events;PDGFR-alpha signaling pathway;PDGFR-beta signaling pathway;Plasma membrane estrogen receptor signaling;Regulation of Ras family activation;Role of Calcineurin-dependent NFAT signaling in lymphocytes;Signaling events mediated by Hepatocyte Growth Factor Receptor (c-Met);Signaling events mediated by VEGFR1 and VEGFR2;TCR signaling in nave CD4+ T cells;TCR signaling in nave CD8+ T cells;Thromboxane A2 receptor signaling;Trk receptor signaling mediated by PI3K and PLC-gamma;VEGFR1 specific signals	SELPLG

Table A (continued)

Main Pathway	Regulators
Arf6 signaling events;BCR signaling pathway;CXCR3-mediated signaling events;CXCR4-mediated signaling events;Class I PI3K signaling events;Class I PI3K signaling events mediated by Akt;E-cadherin signaling in the nascent adherens junction;EPHA2 forward signaling;ErbB1 downstream signaling;ErbB2/ErbB3 signaling events;FAS (CD95) signaling pathway;FGF signaling pathway;GMCSF-mediated signaling events;IFN-gamma pathway;IL2 signaling events mediated by PI3K;IL4-mediated signaling events;IL8- and CXCR1-mediated signaling events;IL8- and CXCR2-mediated signaling events;Insulin Pathway;Nephrin/Neph1 signaling in the kidney podocyte;Osteopontin-mediated events;PDGFR-beta signaling pathway;Plasma membrane estrogen receptor signaling;RAC1 signaling pathway;Regulation of Ras family activation;Signaling events mediated by Hepatocyte Growth Factor Receptor (c-Met);Signaling events mediated by VEGFR1 and VEGFR2;TCR signaling in nave CD4+ T cells;TCR signaling in nave CD8+ T cells;Trk receptor signaling mediated by PI3K and PLC-gamma;VEGFR1 specific signals	GAB2
ATR signaling pathway	YWHAZ
Beta1 integrin cell surface interactions	ZNF488
Beta-catenin phosphorylation cascade	NDFIP1
Budding and maturation of HIV virion;Endosomal Sorting Complex Required For Transport (ESCRT)	ARRDC2
Budding and maturation of HIV virion;Membrane binding and targetting of GAG proteins	RASSF5
B-WICH complex positively regulates rRNA expression	SYT11
Carboxyterminal post-translational modifications of tubulin;Post-chaperonin tubulin folding pathway	NUP210L
Cation-coupled Chloride cotransporters;Neurotransmitter receptors and postsynaptic signal transmission	LINC00051
Cation-coupled Chloride cotransporters;Phase 0 - rapid depolarisation;Stimuli-sensing channels;TRP channels	HTN1
Cdc20:Phospho-APC/C mediated degradation of Cyclin A	PSMB10
Cellular hexose transport;Phase 0 - rapid depolarisation;Stimuli-sensing channels;TRP channels	PART1
Clathrin-mediated endocytosis	RASGRF2
Cleavage of Growing Transcript in the Termination Region;Processing of Intronless Pre-mRNAs;Transport of Mature mRNA Derived from an Intronless Transcript;mRNA 3'-end processing;pre-mRNA splicing	ICAM3
Cleavage of Growing Transcript in the Termination Region;Transport of Mature mRNA derived from an Intron-Containing Transcript;mRNA 3'-end processing	ZC3H3
CMP-N-acetylneuraminate biosynthesis I (eukaryotes)	ST3GAL2
coenzyme A biosynthesis	ANP32B
Complex I biogenesis;Respiratory electron transport	ALDH3A1
Condensation of Prophase Chromosomes	WDR41
Constitutive Signaling by NOTCH1 HD Domain Mutants	ZBTB43
COPI-dependent Golgi-to-ER retrograde traffic;COPI-mediated anterograde transport	ADGRB1
COPI-mediated anterograde transport	DCTN4
Cristae formation;Formation of ATP by chemiosmotic coupling	ATP5G3

Table A (continued)

Main Pathway	Regulators
CTLA4 inhibitory signaling	KLHL24
CXCR4-mediated signaling events;IL12 signaling mediated by STAT4;IL12-mediated signaling events;TCR signaling in nave CD4+ T cells	HSBP1
Cyclin A/B1/B2 associated events during G2/M transition	HAMP
Degradation of DVL	DVL3
Dual incision in TC-NER;Formation of TC-NER Pre-Incision Complex;Gap-filling DNA repair synthesis and ligation in TC-NER;Transcription-Coupled Nucleotide Excision Repair (TC-NER)	C14orf1
Dual incision in TC-NER;Gap-filling DNA repair synthesis and ligation in TC-NER	MCTP1
E3 ubiquitin ligases ubiquitinate target proteins	DERL1
Eukaryotic Translation Termination;Formation of a pool of free 40S subunits;Formation of the ternary complex, and subsequently, the 43S complex;GTP hydrolysis and joining of the 60S ribosomal subunit;L13a-mediated translational silencing of Ceruloplasmin expression;Major pathway of rRNA processing in the nucleolus and cytosol;Nonsense Mediated Decay (NMD) enhanced by the Exon Junction Complex (EJC);Nonsense Mediated Decay (NMD) independent of the Exon Junction Complex (EJC);Peptide chain elongation;Regulation of expression of SLITs and ROBOs;Ribosomal scanning and start codon recognition;SRP-dependent cotranslational protein targeting to membrane;Selenocysteine synthesis;Translation initiation complex formation;Viral mRNA Translation	TLE2
Fanconi anemia pathway	COL4A5
FBXL7 down-regulates AURKA during mitotic entry and in early mitosis	PMP22
FCERI mediated Ca+2 mobilization	PLCG2
Formation of the Early Elongation Complex;Formation of the HIV-1 Early Elongation Complex	DAPK3
G2/M DNA damage checkpoint;Recruitment and ATM-mediated phosphorylation of repair and signaling proteins at DNA double strand breaks	TMOD4
G alpha (q) signalling events;G alpha (s) signalling events;Vasopressin regulates renal water homeostasis via Aquaporins	NPY4R
G alpha (s) signalling events;Vasopressin regulates renal water homeostasis via Aquaporins	MNS1
gamma-glutamyl cycle	APOA1BP
GDP-mannose biosynthesis;colanic acid building blocks biosynthesis	MAN2A1
gluconeogenesis I;glycolysis I	PFKFB1
Glucuronidation	SH2D3C
glutaryl-CoA degradation;ketogenesis;ketolysis;mevalonate pathway I;superpathway of cholesterol biosynthesis;superpathway of geranylgeranyldiphosphate biosynthesis I (via mevalonate);tryptophan degradation III (eukaryotic)	TSPAN15
GTP hydrolysis and joining of the 60S ribosomal subunit;L13a-mediated translational silencing of Ceruloplasmin expression;Ribosomal scanning and start codon recognition;Translation initiation complex formation	IDH1
HDACs deacetylate histones;NoRC negatively regulates rRNA expression	TAF7

Table A (continued)

Main Pathway	Regulators
HDR through Homologous Recombination (HRR);Homologous DNA Pairing and Strand Exchange;Presynaptic phase of homologous DNA pairing and strand exchange;Resolution of D-loop Structures through Holliday Junction Intermediates;Resolution of D-loop Structures through Synthesis-Dependent Strand Annealing (SDSA)	SULT4A1
Heterotrimeric G-protein signaling pathway-Gi alpha and Gs alpha mediated pathway	HHLA1
Heterotrimeric G-protein signaling pathway-Gi alpha and Gs alpha mediated pathway;Muscarinic acetylcholine receptor 2 and 4 signaling pathway	EDN3
Inflammation mediated by chemokine and cytokine signaling pathway;Interleukin signaling pathway;PDGF signaling pathway;VEGF signaling pathway	DCP2
IRF3-mediated induction of type I IFN	IFI16
MASTL Facilitates Mitotic Progression	ENSA
MHC class II antigen presentation	RILP
Mitochondrial translation elongation	DHX16
Mitochondrial translation termination	MRPS28
Mitotic Prometaphase;RHO GTPases Activate Formins	PAM16
Negative regulation of MAPK pathway	RPGR
Nonhomologous End-Joining (NHEJ)	XRCC4
NoRC negatively regulates rRNA expression	SAP30L
Ovarian tumor domain proteases	VCPIP1
Oxidative Stress Induced Senescence	PHC3
p53 pathway	SPINK4
PKMTs methylate histone lysines;RUNX1 regulates genes involved in megakaryocyte differentiation and platelet function	DAAM2
PLK1 signaling events	ECT2
PMID: 9122178;9822713	GNPDA1
PMID: 9187108;9256433	PTK2
Pre-NOTCH Transcription and Translation	PRKCI
Processing of Capped Intron-Containing Pre-mRNA	FEV
Regulation of expression of SLITs and ROBOs	ISL1
Regulation of gene expression in beta cells	RABGAP1
Regulation of insulin secretion;Zinc efflux and compartmentalization by the SLC30 family;Zinc influx into cells by the SLC39 gene family	CACNB3
Regulation of ornithine decarboxylase (ODC)	AZIN1
Regulation of RAC1 activity	ARHGAP1
Respiratory electron transport	CAMK2A
Retinoid cycle disease events;The canonical retinoid cycle in rods (twilight vision)	LST1
Retrograde transport at the Trans-Golgi-Network	RHOBTB3
RHO GTPases Activate WASPs and WAVEs;Regulation of actin dynamics for phagocytic cup formation	RFTN1
RMTs methylate histone arginines	RASA1
RNA Polymerase III Abortive And Retractive Initiation;RNA Polymerase III Transcription Initiation From Type 3 Promoter	BDP1
RUNX1 regulates transcription of genes involved in differentiation of HSCs	CCNH
Signaling by BRAF and RAF fusions	TRAK1
Signaling events regulated by Ret tyrosine kinase	COTL1

Table A (continued)

Main Pathway	Regulators
SMAD2/SMAD3:SMAD4 heterotrimer regulates transcription	CDK8
sulfate activation for sulfonation	CHST12
SUMOylation of DNA damage response and repair proteins	NSMCE2
Synthesis of PIPs at the Golgi membrane	VAC14
TCR signaling in nave CD4+ T cells	KCNA2
Termination of translesion DNA synthesis	MYO1D
tetrahydrobiopterin biosynthesis I;tetrahydrobiopterin biosynthesis II	PTS
TFAP2A acts as a transcriptional repressor during retinoic acid induced cell differentiation;Transcription of E2F targets under negative control by p107 (RBL1) and p130 (RBL2) in complex with HDAC1	NPM1
TFAP2 (AP-2) family regulates transcription of growth factors and their receptors	NDRG1
tRNA processing in the nucleus	POP1
UDP-N-acetyl-D-galactosamine biosynthesis II;UDP-N-acetyl-D-glucosamine biosynthesis II	MLYCD
Validated targets of C-MYC transcriptional activation	GSTP1

Table B. Enriched pathways and associated regulators, known to be connected (Regs Conn) and not known to be connected to the pathway (Regs Nconn)

Pathway	Regs Conn	Regs NConn
pre-mRNA splicing	DHX16<br>ICAM3<br>IDH1<br>MRPL54<br>NIPAL4<br>PROCR<br>SF3B4<br>TNNT2	ASNSD1<br>EMILIN1<br>FIGF<br>GUCA2B<br>KLHL24<br>NPIPB15<br>PCDHB9<br>PURA<br>SMOC2<br>TCERG1<br>TMEM259<br>YTHDC1<br>ZNF446
Major pathway of rRNA processing in the nucleolus and cytosol	DENND1C<br>IDH1<br>KCNK4<br>KDELR1<br>MYC NIP7<br>PDE4C<br>PRKACA<br>PROCR<br>RIPK3<br>RPS23<br>RPS4X<br>SEC24D<br>SF3B4	CDX1<br>FAM212B<br>FCRL5<br>GKN1
SRP-dependent cotranslational protein targeting to membrane	DENND1C<br>IDH1<br>KCNN3<br>KDELR1<br>PRKACA<br>RPL13A<br>RPS23<br>RPS4X<br>SSB TLE2	CLDND2<br>DUOX1<br>FCRL5<br>KLK6<br>RPRM<br>STARD4
Amplification of signal from unattached kinetochores via a MAD2 inhibitory signal;Mitotic Prometaphase;RHO GTPases Activate Formins;Resolution of Sister Chromatid Cohesion;Separation of Sister Chromatids	KCNMB3<br>LSM11<br>MRPL54<br>SLC27A3<br>SSB<br>TSPAN7<br>USP13	ASNSD1<br>AURKC<br>GUCA2B<br>LINC01587<br>NAAL-<br>ADL2<br>SGCZ<br>SOX14<br>TBCK

Table B (continued)

Pathway	Regs Conn	Regs NConn
snRNP Assembly	GCG GEMIN7 GEMIN8 NR3C2 SSB TNNT2	ASNSD1 CFHR4 FIGF GUCA2B HOXD9 LINC01587 POGZ
Eukaryotic Translation Termination;Formation of a pool of free 40S subunits;GTP hydrolysis and joining of the 60S ribosomal subunit;Nonsense Mediated Decay (NMD) enhanced by the Exon Junction Complex (EJC);Nonsense Mediated Decay (NMD) independent of the Exon Junction Complex (EJC);Peptide chain elongation;Regulation of expression of SLITs and ROBOs;SRP-dependent cotranslational protein targeting to membrane;Selenocysteine synthesis;Viral mRNA Translation	DENND1C DUOX1 IDH1 KDELR1 PRKACA RPS23 RPS4X STARD4 TLE2	FCRL5 KLK6 ZCCHC9
Formation of a pool of free 40S subunits;Formation of the ternary complex, and subsequently, the 43S complex;GTP hydrolysis and joining of the 60S ribosomal subunit;L13a-mediated translational silencing of Ceruloplasmin expression;Ribosomal scanning and start codon recognition;Translation initiation complex formation	DENND1C IDH1 KDELR1 MRPL54 MUC5B PRKACA RPS23 RPS4X	GKN1 TLE2 VPS8
Activation of anterior HOX genes in hindbrain development during early embryogenesis	HIST2H2BE NCOA6 NIPAL4 PARP10 PAX6 PLCG2 RAB3A RBBP5 VHL	
Deposition of new CENPA-containing nucleosomes at the centromere	HIST2H2BE KCNMB3 NIPAL4 USP13	LYL1 S100A14 SRPX TBCK ZNF446
Formation of the beta-catenin:TCF transactivating complex	BCL9 HIST2H2BE MYC PYGO2 RAB3A RBBP5 WWOX	CPEB4 HOXD9

Table B (continued)

Pathway	Regs Conn	Regs NConn
Gap-filling DNA repair synthesis and ligation in TC-NER;Processing of DNA double-strand break ends	CCNH IL6R TMOD4 TNNT2	C14orf1 DAPK3 HOXD9 LYL1 MCTP1
NEP/NS2 Interacts with the Cellular Export Machinery;NS1 Mediated Effects on Host Pathways;Nuclear Pore Complex (NPC) Disassembly;Nuclear import of Rev protein;Regulation of Glucokinase by Glucokinase Regulatory Protein;Regulation of HSF1-mediated heat shock response;Rev-mediated nuclear export of HIV RNA;SUMOylation of DNA damage response and repair proteins;SUMOylation of DNA replication proteins;SUMOylation of RNA binding proteins;SUMOylation of chromatin organization proteins;Transcriptional regulation by small RNAs;Transport of Mature mRNA Derived from an Intronless Transcript;Transport of Mature mRNA derived from an Intron-Containing Transcript;Transport of Ribonucleoproteins into the Host Nucleus;Transport of the SLBP Dependant Mature mRNA;Transport of the SLBP independent Mature mRNA;Viral Messenger RNA Synthesis;Vpr-mediated nuclear import of PICs;snRNP Assembly;tRNA processing in the nucleus	NR3C2 SLC30A5 SSB TNNT2	ASNSD1 GUCA2B HOXD9 LINC01587 TSPAN7

Table B (continued)

Pathway	Regs Conn	Regs NConn
Activated NTRK2 signals through PI3K;Activation of PKB;Antigen activates B Cell Receptor (BCR) leading to generation of second messengers;CD28 dependent PI3K/Akt signaling;CD28 dependent Vav1 pathway;Constitutive Signaling by Aberrant PI3K in Cancer;Constitutive Signaling by EGFRvIII;Constitutive Signaling by Ligand-Responsive EGFR Cancer Variants;DAP12 signaling;Downstream TCR signaling;Downstream signal transduction;FCERI mediated Ca+2 mobilization;FCERI mediated MAPK activation;G beta:gamma signalling through PI3Kgamma;GAB1 signalosome;GPVI-mediated activation cascade;Inhibition of TSC complex formation by PKB;MET activates PI3K/AKT signaling;Negative regulation of the PI3K/AKT network;PDE3B signalling;PI Metabolism;PI-3K cascade:FGFR1;PI-3K cascade:FGFR2;PI-3K cascade:FGFR3;PI-3K cascade:FGFR4;PI3K Cascade;PI3K events in ERBB2 signaling;PI3K events in ERBB4 signaling;PI3K/AKT Signaling;PI3K/AKT activation;PI5P, PP2A and IER3 Regulate PI3K/AKT Signaling;PTEN Loss of Function in Cancer;Phospholipase C-mediated cascade: FGFR1;Phospholipase C-mediated cascade; FGFR2;Phospholipase C-mediated cascade; FGFR3;Phospholipase C-mediated cascade; FGFR4;RHO GTPases Activate WASPs and WAVEs;RHO GTPases activate PKNs;RIPK1-mediated regulated necrosis;Regulation of TP53 Degradation;Regulation of actin dynamics for phagocytic cup formation;Role of LAT2/NTAL/LAB on calcium mobilization;Role of phospholipids in phagocytosis;Signaling by FGFR1 in disease;Signaling by FGFR2 in disease;Signaling by FGFR3 fusions in cancer;Signaling by FGFR3 point mutants in cancer;Signaling by FGFR4 in disease;Signaling by SCF-KIT;Signaling by cytosolic FGFR1 fusion mutants;Synthesis of PIPs at the plasma membrane;VEGFA-VEGFR2 Pathway;VEGFR2 mediated vascular permeability	MEF2C<br>PIK3R1<br>PLCG2<br>RASGRF2	ABCC12<br>IGSF9<br>MNS1<br>PBXIP1
HDACs deacetylate histones	HDAC10<br>HIST2H2BE<br>RAB3A<br>SAP30L<br>TBL1XR1	HOXD9<br>SOX14<br>WFIKKN1
HDR through Homologous Recombination (HRR)	IL6R<br>POLK<br>RAD51D<br>RNF139<br>SULT4A1<br>TMOD4	HOXD9<br>PAM
Major pathway of rRNA processing in the nucleolus and cytosol;rRNA modification in the nucleus and cytosol	AGO2<br>BCAN<br>SEC24D<br>SF3B4	CDX1<br>CFHR4<br>GKN1<br>KCNK4
Dual incision in TC-NER;Gap-filling DNA repair synthesis and ligation in TC-NER	BCAN<br>CCNH<br>POLK	C14orf1<br>DAPK3<br>LYL1<br>MCTP1

Table B (continued)

Pathway	Regs Conn	Regs NConn
Eukaryotic Translation Termination;Formation of a pool of free 40S subunits;Formation of the ternary complex, and subsequently, the 43S complex;GTP hydrolysis and joining of the 60S ribosomal subunit;L13a-mediated translational silencing of Ceruloplasmin expression;Major pathway of rRNA processing in the nucleolus and cytosol;Nonsense Mediated Decay (NMD) enhanced by the Exon Junction Complex (EJC);Nonsense Mediated Decay (NMD) independent of the Exon Junction Complex (EJC);Peptide chain elongation;Regulation of expression of SLITs and ROBOs;Ribosomal scanning and start codon recognition;SRP-dependent cotranslational protein targeting to membrane;Selenocysteine synthesis;Translation initiation complex formation;Viral mRNA Translation	DENND1C IDH1 KDELR1 PRKACA RPS23 RPS4X TLE2	
HIV Transcription Initiation;RNA Polymerase II HIV Promoter Escape;RNA Polymerase II Pre-transcription Events;RNA Polymerase II Promoter Escape;RNA Polymerase II Transcription Initiation;RNA Polymerase II Transcription Initiation And Promoter Clearance;RNA Polymerase II Transcription Pre-Initiation And Promoter Opening;Transcription of the HIV genome	ATG10 CCNH SLC25A37 TAF7	DAPK3 FBLN1 PARP10
Integrin signalling pathway	CX3CL1 DOCK1 PIK3R1 PTK2	FPR2 PTPRE ZEB1
Nonsense Mediated Decay (NMD) enhanced by the Exon Junction Complex (EJC)	PAX6 PHC3 RPL13A RPS23 RPS4X	KLK6 ZCCHC9
Cleavage of Growing Transcript in the Termination Region;mRNA 3'-end processing;pre-mRNA splicing	MRPL54	ICAM3 NPIPB15 PROCR SMOC2 ZNF446
Condensation of Prophase Chromosomes	HIST2H2BE RAB3A	LYL1 POGZ WDR41 ZNF446
Dual incision in TC-NER;Formation of TC-NER Pre-Incision Complex;Gap-filling DNA repair synthesis and ligation in TC-NER;Transcription-Coupled Nucleotide Excision Repair (TC-NER)	BCAN CCNH TNNT2	C14orf1 DAPK3 LYL1
Estrogen-dependent gene expression	AGO2 CHD1 HIST2H2BE IQGAP2 MYC	PIEZO1

Table B (continued)

Pathway	Regs Conn	Regs NConn
Eukaryotic Translation Termination;Formation of a pool of free 40S subunits;GTP hydrolysis and joining of the 60S ribosomal subunit;L13a-mediated translational silencing of Ceruloplasmin expression;Major pathway of rRNA processing in the nucleolus and cytosol;Nonsense Mediated Decay (NMD) enhanced by the Exon Junction Complex (EJC);Nonsense Mediated Decay (NMD) independent of the Exon Junction Complex (EJC);Peptide chain elongation;Regulation of expression of SLITs and ROBOs;SRP-dependent cotranslational protein targeting to membrane;Selenocysteine synthesis;Viral mRNA Translation	DUOX1 PRKACA RPL13A STARD4	KLK6 ZC-CHC9
G2/M DNA damage checkpoint;Nonhomologous End-Joining (NHEJ);Processing of DNA double-strand break ends;Recruitment and ATM-mediated phosphorylation of repair and signaling proteins at DNA double strand breaks	GCG HIST2H2BE MRPL54 NIPAL4 RNF139	PAM
HDR through Single Strand Annealing (SSA);Presynaptic phase of homologous DNA pairing and strand exchange;Processing of DNA double-strand break ends;Regulation of TP53 Activity through Phosphorylation	IL6R RNF139 TMOD4 UBE2T	HOXD9 PAM
PKMTs methylate histone lysines	ASH1L KMT2E MECOM PARP10 RBBP5	DQX1
Resolution of D-loop Structures through Holliday Junction Intermediates	IL6R RAD51D	CDX1 HOXD9 SULT4A1 TMOD4
Rho GTPase cycle	ARHGAP30 ARHGEF4 NSMCE2 RASGRF2	LRRIQ4 MYO1D
RNA polymerase II transcribes snRNA genes	ELL ELL2 ELL3 SNAPC2	PITX1 POGZ
RUNX1 regulates genes involved in megakaryocyte differentiation and platelet function	HIST2H2BE KMT2E RAB3A RBBP5	ANKRD46 HOXD9
Ub-specific processing proteases	ADRB2 AR HIST2H2BE MYC PSMB10 USP13	

Table B (continued)

Pathway	Regs Conn	Regs NConn
Amplification of signal from unattached kinetochores via a MAD2 inhibitory signal;Mitotic Prometaphase;NEP/NS2 Interacts with the Cellular Export Machinery;NS1 Mediated Effects on Host Pathways;Nuclear Pore Complex (NPC) Disassembly;Nuclear import of Rev protein;RHO GTPases Activate Formins;Regulation of Glucokinase by Glucokinase Regulatory Protein;Regulation of HSF1-mediated heat shock response;Resolution of Sister Chromatid Cohesion;Rev-mediated nuclear export of HIV RNA;SUMOylation of DNA damage response and repair proteins;SUMOylation of DNA replication proteins;SUMOylation of RNA binding proteins;SUMOylation of chromatin organization proteins;Separation of Sister Chromatids;Transcriptional regulation by small RNAs;Transport of Mature mRNA Derived from an Intronless Transcript;Transport of Mature mRNA derived from an Intron-Containing Transcript;Transport of Ribonucleoproteins into the Host Nucleus;Transport of the SLBP Dependant Mature mRNA;Transport of the SLBP independent Mature mRNA;Viral Messenger RNA Synthesis;Vpr-mediated nuclear import of PICs;snRNP Assembly;tRNA processing in the nucleus	SSB	ASNSD1 GUCA2B LINC01587 TSPAN7
Antigen processing: Ubiquitination and Proteasome degradation	ATG7 LRSAM1 MRPL54	NPIPB15 ZCCHC9
B-WICH complex positively regulates rRNA expression	HIST2H2BE NIPAL4 SYT11	ANKRD46 LYL1
Cleavage of Growing Transcript in the Termination Region;Transport of Mature mRNA derived from an Intron-Containing Transcript;mRNA 3'-end processing	BCAN MRPL54 PROCR	SMOC2 ZC3H3
Downstream TCR signaling;Generation of second messenger molecules;PD-1 signaling;Phosphorylation of CD3 and TCR zeta chains;Translocation of ZAP-70 to Immunological synapse	ARHGEF4 HSBP1 VHL	ATXN7L2 POGZ
Fanconi Anemia Pathway	DCTN4 RNF139 UBE2T	GALE KLK11
HDR through Homologous Recombination (HRR);HDR through Single Strand Annealing (SSA);Homologous DNA Pairing and Strand Exchange;Presynaptic phase of homologous DNA pairing and strand exchange;Processing of DNA double-strand break ends;Regulation of TP53 Activity through Phosphorylation;Resolution of D-loop Structures through Holliday Junction Intermediates;Resolution of D-loop Structures through Synthesis-Dependent Strand Annealing (SDSA)	IL6R RNF139 TMOD4	HOXD9 PAM
HDR through MMEJ (alt-NHEJ);Processing of DNA double-strand break ends	IL6R RNF139 TMOD4	HOXD9 PAM
MHC class II antigen presentation	DCTN4 HSBP1 RASGRF2 RILP	POGZ

Table B (continued)

Pathway	Regs Conn	Regs NConn
Mitochondrial translation elongation;Mitochondrial translation initiation;Mitochondrial translation termination	MRPL54 MRPS28	ADAMTSL4 FIGF SLC39A13
Nonsense Mediated Decay (NMD) enhanced by the Exon Junction Complex (EJC);Regulation of expression of SLITs and ROBOs	PROCR RPL13A RPS23 RPS4X	KLK6
Recruitment and ATM-mediated phosphorylation of repair and signaling proteins at DNA double strand breaks	HIST2H2BE MAN2A1 RNF139 TMOD4	PAM
TP53 Regulates Transcription of DNA Repair Genes	CCNH ELL PROCR RAD51D	S100A14
U12 Dependent Splicing;pre-mRNA splicing	SF3B4	FIGF NPIPB15 SMOC2 TCERG1
Activated PKN1 stimulates transcription of AR (androgen receptor) regulated genes KLK2 and KLK3;Estrogen-dependent gene expression;PRC2 methylates histones and DNA;RUNX1 regulates genes involved in megakaryocyte differentiation and platelet function;RUNX1 regulates transcription of genes involved in differentiation of HSCs	HIST2H2BE RAB3A	LYL1 ZNF446
Anchoring of the basal body to the plasma membrane	PDE8B	ATG10 CERK TMEM37
Arf6 signaling events;BCR signaling pathway;CXCR3-mediated signaling events;CXCR4-mediated signaling events;Class I PI3K signaling events;Class I PI3K signaling events mediated by Akt;E-cadherin signaling in the nascent adherens junction;EPHA2 forward signaling;ErbB1 downstream signaling;ErbB2/ErbB3 signaling events;FAS (CD95) signaling pathway;FGF signaling pathway;GMCSF-mediated signaling events;IFN-gamma pathway;IL2 signaling events mediated by PI3K;IL4-mediated signaling events;IL8- and CXCR1-mediated signaling events;IL8- and CXCR2-mediated signaling events;Insulin Pathway;Nephrin/Neph1 signaling in the kidney podocyte;Osteopontin-mediated events;PDGFR-beta signaling pathway;Plasma membrane estrogen receptor signaling;RAC1 signaling pathway;Regulation of Ras family activation;Signaling events mediated by Hepatocyte Growth Factor Receptor (c-Met);Signaling events mediated by VEGFR1 and VEGFR2;TCR signaling in nave CD4+ T cells;TCR signaling in nave CD8+ T cells;Trk receptor signaling mediated by PI3K and PLC-gamma;VEGFR1 specific signals	ARHGEF4 GAB2 PIK3R1	COTL1

Table B (continued)

Pathway	Regs Conn	Regs NConn
AURKA Activation by TPX2;Anchoring of the basal body to the plasma membrane;Loss of Nlp from mitotic centrosomes;Loss of proteins required for interphase microtubule organizationfrom the centrosome;Recruitment of NuMA to mitotic centrosomes;Recruitment of mitotic centrosome proteins and complexes;Regulation of PLK1 Activity at G2/M Transition	ADH1C ATG10	KRTAP15-1 SOX14
Clathrin-mediated endocytosis	PFKFB1 RASGRF2	ATXN7L2 MYO1D
Cleavage of Growing Transcript in the Termination Region;Transport of Mature mRNA derived from an Intron-Containing Transcript;mRNA 3'-end processing;pre-mRNA splicing	MRPL54 PROCR	NPIPB15 SMOC2
COPI-mediated anterograde transport	DCTN4 KDELR2 MEF2C	ADGRB1
E2F transcription factor network	CES2 DHFR MYC	CXCL14
Fanconi anemia pathway	COL4A5 UBE2T	HOXD9 RNF139
Fanconi Anemia Pathway;Processing of DNA double-strand break ends	DCTN4 IL6R	GALE KLK11
Gap-filling DNA repair synthesis and ligation in TC-NER;HDR through MMEJ (alt-NHEJ);Processing of DNA double-strand break ends	CCNH RNF139	MCTP1 PAM
GTP hydrolysis and joining of the 60S ribosomal subunit;L13a-mediated translational silencing of Ceruloplasmin expression;Ribosomal scanning and start codon recognition;Translation initiation complex formation	IDH1 RPS23 RPS4X	MUC5B
Neddylation	BCAN KLHL3 PROCR PSMB10	
NoRC negatively regulates rRNA expression	HIST2H2BE SAP30L TAF7	HOXD9
Oxidative Stress Induced Senescence	HIST2H2BE PHC3 RAB3A	ASNSD1
TCR signaling in nave CD4+ T cells	ARHGEF4	ATXN7L2 HSBP1 KCNA2
Transport of Mature mRNA derived from an Intron-Containing Transcript	PROCR SSB	GUCA2B LINC01587

Table B (continued)

Pathway	Regs Conn	Regs NConn
Acetylcholine regulates insulin secretion;Activation of NF-kappaB in B cells;Activation of RAS in B cells;Antigen activates B Cell Receptor (BCR) leading to generation of second messengers;Arachidonate production from DAG;Ca2+ pathway;EGFR Transactivation by Gastrin;Effects of PIP2 hydrolysis;Elevation of cytosolic Ca2+ levels;Fatty Acids bound to GPR40 (FFAR1) regulate insulin secretion;G alpha (q) signalling events;G beta:gamma signalling through PLC beta;GPVI-mediated activation cascade;Rap1 signalling;Response to elevated platelet cytosolic Ca2+;Syndecan interactions;Synthesis of IP3 and IP4 in the cytosol	INTS8<br>PLCG2<br>TERF2IP	
Activated PKN1 stimulates transcription of AR (androgen receptor) regulated genes KLK2 and KLK3;Activation of anterior HOX genes in hindbrain development during early embryogenesis;Activation of rRNA Expression by ERCC6 (CSB) and EHMT2 (G9a);B-WICH complex positively regulates rRNA expression;Condensation of Prophase Chromosomes;DNA methylation;Estrogen-dependent gene expression;Formation of the beta-catenin:TCF transactivating complex;HDACs deacetylate histones;Meiotic recombination;NoRC negatively regulates rRNA expression;Oxidative Stress Induced Senescence;PRC2 methylates histones and DNA;RNA Polymerase I Chain Elongation;RNA Polymerase I Promoter Opening;RUNX1 regulates genes involved in megakaryocyte differentiation and platelet function;RUNX1 regulates transcription of genes involved in differentiation of HSCs;SIRT1 negatively regulates rRNA expression;Senescence-Associated Secretory Phenotype (SASP);Transcriptional regulation by small RNAs	HIST2H2BE<br>RAB3A	LYL1

Table B (continued)

Pathway	Regs Conn	Regs NConn
Activation of AMPA receptors;Activation of Ca-permeable Kainate Receptor;Activation of CaMK IV;Activation of NF-kappaB in B cells;Activation of the phototransduction cascade;Amyloid fiber formation;Antigen activates B Cell Receptor (BCR) leading to generation of second messengers;CLEC7A (Dectin-1) induces NFAT activation;CREB phosphorylation through the activation of Adenylate Cyclase;CREB phosphorylation through the activation of CaMKII;Ca2+ pathway;CaM pathway;CaMK IV-mediated phosphorylation of CREB;Calcineurin activates NFAT;Calmodulin induced events;Cam-PDE 1 activation;Chromatin modifying enzymes;DAG and IP3 signaling;DARPP-32 events;Defective SLC24A1 causes congenital stationary night blindness 1D (CSNB1D);Defective SLC24A4 causes hypomineralized amelogenesis imperfecta (AI);Degradation of the extracellular matrix;Deregulated CDK5 triggers multiple neurodegenerative pathways in Alzheimer's disease models;Effects of PIP2 hydrolysis;Elevation of cytosolic Ca2+ levels;FCERI mediated Ca+2 mobilization;FCERI mediated NF-kB activation;Glutamate Neurotransmitter Release Cycle;Glycerophospholipid biosynthesis;Glycogen breakdown (glycogenolysis);Glycosphingolipid metabolism;Highly calcium permeable nicotinic acetylcholine receptors;Highly calcium permeable postsynaptic nicotinic acetylcholine receptors;Inactivation, recovery and regulation of the phototransduction cascade;Ion homeostasis;Ion transport by P-type ATPases;Mitochondrial calcium ion transport;Neurotransmitter receptors and postsynaptic signal transmission;PKA activation;PLC beta mediated events;Phase 0 - rapid depolarisation;Phase 2 - plateau phase;Presynaptic depolarization and calcium channel opening;RHO GTPases activate PAKs;Rap1 signalling;Ras activation upon Ca2+ influx through NMDA receptor;Reduction of cytosolic Ca++ levels;Regulation of insulin secretion;Response to elevated platelet cytosolic Ca2+;Role of phospholipids in phagocytosis;Smooth Muscle Contraction;Sodium/Calcium exchangers;Sperm Motility And Taxes;Stimuli-sensing channels;Striated Muscle Contraction;Synthesis of Leukotrienes (LT) and Eoxins (EX);TRP channels;Tetrahydrobiopterin (BH4) synthesis, recycling, salvage and regulation;Translocation of GLUT4 to the plasma membrane;Unblocking of NMDA receptor, glutamate binding and activation;Uptake and function of anthrax toxins;VEGFR2 mediated cell proliferation;VEGFR2 mediated vascular permeability;eNOS activation;phospho-PLA2 pathway		ETV7<br>MCMDC2<br>PEBP4
Activation of anterior HOX genes in hindbrain development during early embryogenesis;RUNX1 regulates genes involved in megakaryocyte differentiation and platelet function	HIST2H2BE<br>RBBP5	DAAM2
Alpha4 beta1 integrin signaling events	CX3CL1<br>JAML<br>PTK2	

Table B (continued)

Pathway	Regs Conn	Regs NConn
Amplification of signal from unattached kinetochores via a MAD2 inhibitory signal;Deposition of new CENPA-containing nucleosomes at the centromere;Mitotic Prometaphase;RHO GTPases Activate Formins;Resolution of Sister Chromatid Cohesion;Separation of Sister Chromatids	USP13	KCNMB3 TBCK
Antigen activates B Cell Receptor (BCR) leading to generation of second messengers	ARHGEF4 PIK3R1 PLCG2	
APC/C:Cdc20 mediated degradation of Securin;APC/C:Cdh1 mediated degradation of Cdc20 and other APC/C:Cdh1 targeted proteins in late mitosis/early G1;Autodegradation of Cdh1 by Cdh1:APC/C;Cdc20:Phospho-APC/C mediated degradation of Cyclin A;Separation of Sister Chromatids	PSMB10	CDX1 FCRL2
APC-Cdc20 mediated degradation of Nek2A;APC/C:Cdc20 mediated degradation of Cyclin B;APC/C:Cdc20 mediated degradation of Securin;APC/C:Cdc20 mediated degradation of mitotic proteins;APC/C:Cdh1 mediated degradation of Cdc20 and other APC/C:Cdh1 targeted proteins in late mitosis/early G1;Antigen processing: Ubiquitination and Proteasome degradation;Autodegradation of Cdh1 by Cdh1:APC/C;CDK-mediated phosphorylation and removal of Cdc6;Cdc20:Phospho-APC/C mediated degradation of Cyclin A;Conversion from APC/C:Cdc20 to APC/C:Cdh1 in late anaphase;Inactivation of APC/C via direct inhibition of the APC/C complex;Phosphorylation of the APC/C;Regulation of APC/C activators between G1/S and early anaphase;Senescence-Associated Secretory Phenotype (SASP);Separation of Sister Chromatids	SLC25A2	FCRL2 NQO2
Clathrin-mediated endocytosis;Downstream TCR signaling;Invadopodia formation;PIPs transport between plasma and early endosome membranes;Regulation of actin dynamics for phagocytic cup formation;Role of LAT2/NTAL/LAB on calcium mobilization;Synthesis of PIPs at the plasma membrane	PIK3R1 RASGRF2	ATXN7L2
Dual incision in TC-NER;Formation of HIV elongation complex in the absence of HIV Tat;Formation of HIV-1 elongation complex containing HIV-1 Tat;Formation of RNA Pol II elongation complex;Formation of TC-NER Pre-Incision Complex;Gap-filling DNA repair synthesis and ligation in TC-NER;HIV Transcription Initiation;RNA Pol II CTD phosphorylation and interaction with CE;RNA Pol II CTD phosphorylation and interaction with CE during HIV infection;RNA Polymerase II HIV Promoter Escape;RNA Polymerase II Pre-transcription Events;RNA Polymerase II Promoter Escape;RNA Polymerase II Transcription Elongation;RNA Polymerase II Transcription Initiation;RNA Polymerase II Transcription Initiation And Promoter Clearance;RNA Polymerase II Transcription Pre-Initiation And Promoter Opening;TP53 Regulates Transcription of DNA Repair Genes;Tat-mediated elongation of the HIV-1 transcript;Transcription of the HIV genome;Transcription-Coupled Nucleotide Excision Repair (TC-NER);mRNA Capping	CCNH TNNT2	DAPK3

Table B (continued)

Pathway	Regs Conn	Regs NConn
Eukaryotic Translation Termination;Nonsense Mediated Decay (NMD) enhanced by the Exon Junction Complex (EJC);Nonsense Mediated Decay (NMD) independent of the Exon Junction Complex (EJC);Regulation of expression of SLITs and ROBOs	RPL13A<br>RPS23<br>RPS4X	
Formation of HIV elongation complex in the absence of HIV Tat;Formation of HIV-1 elongation complex containing HIV-1 Tat;Formation of RNA Pol II elongation complex;RNA Polymerase II Transcription Elongation;TP53 Regulates Transcription of DNA Repair Genes;Tat-mediated elongation of the HIV-1 transcript	CCNH<br>NTRK1<br>TNNT2	
Formation of the Early Elongation Complex;Formation of the HIV-1 Early Elongation Complex	CCNH<br>TNNT2	DAPK3
Formation of the ternary complex, and subsequently, the 43S complex;GTP hydrolysis and joining of the 60S ribosomal subunit;L13a-mediated translational silencing of Ceruloplasmin expression;Ribosomal scanning and start codon recognition;Translation initiation complex formation	BCAN<br>RPS23<br>RPS4X	
Gap-filling DNA repair synthesis and ligation in GG-NER;Gap-filling DNA repair synthesis and ligation in TC-NER;HDR through Homologous Recombination (HRR);PCNA-Dependent Long Patch Base Excision Repair;Processing of DNA double-strand break ends;Termination of translesion DNA synthesis	IL6R	HOXD9<br>TMOD4
Gap-filling DNA repair synthesis and ligation in GG-NER;Gap-filling DNA repair synthesis and ligation in TC-NER;HDR through Homologous Recombination (HRR);PCNA-Dependent Long Patch Base Excision Repair;Processing of DNA double-strand break ends;Termination of translesion DNA synthesis;Translesion Synthesis by POLH;Translesion synthesis by POLI;Translesion synthesis by POLK;Translesion synthesis by REV1	IL6R<br>TMOD4	HOXD9
glutaryl-CoA degradation;ketogenesis;ketolysis;mevalonate pathway I;superpathway of cholesterol biosynthesis;superpathway of geranylgeranyldiphosphate biosynthesis I (via mevalonate);tryptophan degradation III (eukaryotic)	ALDH3A1<br>MLYCD	TSPAN15
GTP hydrolysis and joining of the 60S ribosomal subunit	RPL13A<br>RPS23<br>RPS4X	
HATs acetylate histones	HIST2H2BE<br>PARP10	POGZ
HDACs deacetylate histones;NoRC negatively regulates rRNA expression	HIST2H2BE<br>SAP30L<br>TAF7	
HDMs demethylate histones	DERL1<br>MAN2A1<br>POLD3	
HDR through MMEJ (alt-NHEJ);Nonhomologous End-Joining (NHEJ);Processing of DNA double-strand break ends	RNF139<br>XRCC4	PAM
Mitochondrial translation elongation	MRPL54<br>MRPS28	DHX16

Table B (continued)

Pathway	Regs Conn	Regs NConn
Nonhomologous End-Joining (NHEJ);Processing of DNA double-strand break ends	IL6R<br>XRCC4	TMOD4
Nonsense Mediated Decay (NMD) enhanced by the Exon Junction Complex (EJC);Nonsense Mediated Decay (NMD) independent of the Exon Junction Complex (EJC);Regulation of expression of SLITs and ROBOs	RPL13A<br>RPS23<br>RPS4X	
PDGF signaling pathway	PIK3R1<br>PLCG2	IQGAP2
Peptide chain elongation	RPL13A<br>RPS23<br>RPS4X	
PKMTs methylate histone lysines;RUNX1 regulates genes involved in megakaryocyte differentiation and platelet function	KMT2E<br>RBBP5	DAAM2
Regulation of HSF1-mediated heat shock response		GUCA2B<br>LINC01587<br>SLC39A13
Resolution of Sister Chromatid Cohesion;Separation of Sister Chromatids	NIPAL4	SGCZ<br>TSPAN7
Selenocysteine synthesis	RPL13A<br>RPS23<br>RPS4X	
Signaling events regulated by Ret tyrosine kinase	PIK3R1<br>PTK2	COTL1
SUMOylation of DNA damage response and repair proteins	NSMCE2<br>PHC3<br>XRCC4	
TNFR2 non-canonical NF-kB pathway	POLD3<br>PSMB10<br>TNNT2	
Transcriptional regulation by small RNAs	AGO2<br>HIST2H2BE	GIMAP2
VEGFA-VEGFR2 Pathway	MEF2C<br>PIK3R1<br>PTK2	

Table B (continued)

Pathway	Regs Conn	Regs NConn
ABC-family proteins mediated transport;APC/C:Cdc20 mediated degradation of Securin;APC/C:Cdh1 mediated degradation of Cdc20 and other APC/C:Cdh1 targeted proteins in late mitosis/early G1;AUF1 (hnRNP D0) binds and destabilizes mRNA;Activation of NF-kappaB in B cells;Antigen processing: Ubiquitination and Proteasome degradation;Asymmetric localization of PCP proteins;Autodegradation of Cdh1 by Cdh1:APC/C;Autodegradation of the E3 ubiquitin ligase COP1;CDK-mediated phosphorylation and removal of Cdc6;CDT1 association with the CDC6:ORC:origin complex;Cdc20:Phospho-APC/C mediated degradation of Cyclin A;Cross-presentation of soluble exogenous antigens (endosomes);Dectin-1 mediated noncanonical NF-kB signaling;Defective CFTR causes cystic fibrosis;Degradation of AXIN;Degradation of DVL;Degradation of GLI1 by the proteasome;Degradation of GLI2 by the proteasome;Degradation of beta-catenin by the destruction complex;ER-Phagosome pathway;FBXL7 down-regulates AURKA during mitotic entry and in early mitosis;FCERI mediated NF-kB activation;G2/M Checkpoints;GLI3 is processed to GLI3R by the proteasome;Hedgehog 'on' state;Hedgehog ligand biogenesis;Hh mutants that don't undergo autocatalytic processing are degraded by ERAD;Interleukin-1 signaling;MAPK6/MAPK4 signaling;Neddylation;Orc1 removal from chromatin;Oxygen-dependent proline hydroxylation of Hypoxia-inducible Factor Alpha;RUNX1 regulates transcription of genes involved in differentiation of HSCs;Regulation of PTEN stability and activity;Regulation of RAS by GAPs;Regulation of RUNX2 expression and activity;Regulation of RUNX3 expression and activity;Regulation of activated PAK-2p34 by proteasome mediated degradation;Regulation of expression of SLITs and ROBOs;Regulation of ornithine decarboxylase (ODC);SCF(Skp2)-mediated degradation of p27/p21;SCF-beta-TrCP mediated degradation of Emi1;TNFR2 non-canonical NF-kB pathway;UCH proteinases;Ub-specific processing proteases;Ubiquitin Mediated Degradation of Phosphorylated Cdc25A;Ubiquitin-dependent degradation of Cyclin D1;Vif-mediated degradation of APOBEC3G;Vpu mediated degradation of CD4	PIK3R1<br>PSMB10	

Table B (continued)

Pathway	Regs Conn	Regs NConn
Acetylcholine regulates insulin secretion;Activated NTRK2 signals through PI3K;Antigen activates B Cell Receptor (BCR) leading to generation of second messengers;CD28 dependent PI3K/Akt signaling;CLEC7A (Dectin-1) signaling;Ca2+ pathway;Cargo recognition for clathrin-mediated endocytosis;Clathrin-mediated endocytosis;Constitutive Signaling by AKT1 E17K in Cancer;Constitutive Signaling by Aberrant PI3K in Cancer;Constitutive Signaling by EGFRvIII;Constitutive Signaling by Ligand-Responsive EGFR Cancer Variants;DAG and IP3 signaling;DAP12 signaling;DCC mediated attractive signaling;Downstream TCR signaling;Downstream signal transduction;EGFR downregulation;EPHB-mediated forward signaling;ER-Phagosome pathway;FCERI mediated Ca+2 mobilization;Fatty Acids bound to GPR40 (FFAR1) regulate insulin secretion;G alpha (q) signalling events;G beta:gamma signalling through PI3Kgamma;G beta:gamma signalling through PLC beta;GAB1 signalosome;GPVI-mediated activation cascade;GRB2:SOS provides linkage to MAPK signaling for Integrins;Generation of second messenger molecules;IRAK4 deficiency (TLR2/4);Integrin alphaIIb beta3 signaling;MET activates PI3K/AKT signaling;MyD88 deficiency (TLR2/4);MyD88:Mal cascade initiated on plasma membrane;Negative regulation of the PI3K/AKT network;Netrin-1 signaling;PI Metabolism;PI-3K cascade:FGFR1;PI-3K cascade:FGFR2;PI-3K cascade:FGFR3;PI-3K cascade:FGFR4;PI3K Cascade;PI3K events in ERBB2 signaling;PI3K events in ERBB4 signaling;PI3K/AKT Signaling;PI3K/AKT activation;PI5P, PP2A and IER3 Regulate PI3K/AKT Signaling;PLC beta mediated events;RHO GTPases Activate WASPs and WAVEs;RIPK1-mediated regulated necrosis;Recycling pathway of L1;Regulation of actin dynamics for phagocytic cup formation;Role of LAT2/NTAL/LAB on calcium mobilization;Role of phospholipids in phagocytosis;Role of second messengers in netrin-1 signaling;Signaling by FGFR1 in disease;Signaling by FGFR2 in disease;Signaling by FGFR3 fusions in cancer;Signaling by FGFR3 point mutants in cancer;Signaling by FGFR4 in disease;Signaling by SCF-KIT;Signaling by cytosolic FGFR1 fusion mutants;Syndecan interactions;Synthesis of IP3 and IP4 in the cytosol;Synthesis of PIPs at the plasma membrane;VEGFA-VEGFR2 Pathway;VEGFR2 mediated cell proliferation;WNT mediated activation of DVL;p130Cas linkage to MAPK signaling for integrins	PIP5K1A PTK2	

Table B (continued)

Pathway	Regs Conn	Regs NConn
Acetylcholine regulates insulin secretion;Activation of NF-kappaB in B cells;Activation of RAS in B cells;Antigen activates B Cell Receptor (BCR) leading to generation of second messengers;Arachidonate production from DAG;CLEC7A (Dectin-1) signaling;Ca2+ pathway;DAG and IP3 signaling;Downstream TCR signaling;EGFR Transactivation by Gastrin;Effects of PIP2 hydrolysis;Elevation of cytosolic Ca2+ levels;FCERI mediated Ca+2 mobilization;FCERI mediated NF-kB activation;Fatty Acids bound to GPR40 (FFAR1) regulate insulin secretion;G alpha (q) signalling events;G beta:gamma signalling through PLC beta;GPVI-mediated activation cascade;Generation of second messenger molecules;PLC beta mediated events;Rap1 signalling;Response to elevated platelet cytosolic Ca2+;Role of phospholipids in phagocytosis;Role of second messengers in netrin-1 signaling;Sphingolipid de novo biosynthesis;Syndecan interactions;Synthesis of IP3 and IP4 in the cytosol;VEGFR2 mediated cell proliferation	PLCG2 TERF2IP	
Acetylcholine regulates insulin secretion;Antigen activates B Cell Receptor (BCR) leading to generation of second messengers;CLEC7A (Dectin-1) signaling;Ca2+ pathway;Clathrin-mediated endocytosis;DAG and IP3 signaling;FCERI mediated Ca+2 mobilization;Fatty Acids bound to GPR40 (FFAR1) regulate insulin secretion;G beta:gamma signalling through PLC beta;GPVI-mediated activation cascade;Generation of second messenger molecules;PI5P, PP2A and IER3 Regulate PI3K/AKT Signaling;PLC beta mediated events;Role of phospholipids in phagocytosis;Role of second messengers in netrin-1 signaling;Synthesis of IP3 and IP4 in the cytosol;Synthesis of PIPs at the plasma membrane;VEGFR2 mediated cell proliferation;WNT mediated activation of DVL	PIP5K1A PLCG2	

Table B (continued)

Pathway	Regs Conn	Regs NConn
Activated NTRK2 signals through PI3K;Activation of PKB;Antigen activates B Cell Receptor (BCR) leading to generation of second messengers;CD28 dependent PI3K/Akt signaling;CD28 dependent Vav1 pathway;Clathrin-mediated endocytosis;Constitutive Signaling by Aberrant PI3K in Cancer;Constitutive Signaling by EGFRvIII;Constitutive Signaling by Ligand-Responsive EGFR Cancer Variants;DAP12 signaling;Downstream TCR signaling;Downstream signal transduction;FCERI mediated Ca+2 mobilization;FCERI mediated MAPK activation;G beta:gamma signalling through PI3Kgamma;GAB1 signalosome;GPVI-mediated activation cascade;Inhibition of TSC complex formation by PKB;Invadopodia formation;MET activates PI3K/AKT signaling;Negative regulation of the PI3K/AKT network;PDE3B signalling;PI Metabolism;PI-3K cascade:FGFR1;PI-3K cascade:FGFR2;PI-3K cascade:FGFR3;PI-3K cascade:FGFR4;PI3K Cascade;PI3K events in ERBB2 signaling;PI3K events in ERBB4 signaling;PI3K/AKT Signaling;PI3K/AKT activation;PI5P, PP2A and IER3 Regulate PI3K/AKT Signaling;PIPs transport between plasma and early endosome membranes;PTEN Loss of Function in Cancer;Phospholipase C-mediated cascade: FGFR1;Phospholipase C-mediated cascade; FGFR2;Phospholipase C-mediated cascade; FGFR3;Phospholipase C-mediated cascade; FGFR4;RHO GTPases Activate WASPs and WAVEs;RHO GTPases activate PKNs;RIPK1-mediated regulated necrosis;Regulation of TP53 Degradation;Regulation of actin dynamics for phagocytic cup formation;Role of LAT2/NTAL/LAB on calcium mobilization;Role of phospholipids in phagocytosis;Signaling by FGFR1 in disease;Signaling by FGFR2 in disease;Signaling by FGFR3 fusions in cancer;Signaling by FGFR3 point mutants in cancer;Signaling by FGFR4 in disease;Signaling by SCF-KIT;Signaling by cytosolic FGFR1 fusion mutants;Synthesis of PIPs at the plasma membrane;VEGFA-VEGFR2 Pathway;VEGFR2 mediated vascular permeability	PIK3R1 PIP5K1A	

Table B (continued)

Pathway	Regs Conn	Regs NConn
Activated NTRK2 signals through PI3K;Antigen activates B Cell Receptor (BCR) leading to generation of second messengers;CD28 dependent PI3K/Akt signaling;Clathrin-mediated endocytosis;Constitutive Signaling by Aberrant PI3K in Cancer;Constitutive Signaling by EGFRvIII;Constitutive Signaling by Ligand-Responsive EGFR Cancer Variants;DAP12 signaling;Downstream TCR signaling;Downstream signal transduction;G beta:gamma signalling through PI3Kgamma;GAB1 signalosome;GPVI-mediated activation cascade;MET activates PI3K/AKT signaling;PI-3K cascade:FGFR1;PI-3K cascade:FGFR2;PI-3K cascade:FGFR3;PI-3K cascade:FGFR4;PI3K Cascade;PI3K events in ERBB2 signaling;PI3K events in ERBB4 signaling;PI3K/AKT Signaling;PI3K/AKT activation;PI5P, PP2A and IER3 Regulate PI3K/AKT Signaling;Role of LAT2/NTAL/LAB on calcium mobilization;Role of phospholipids in phagocytosis;Signaling by FGFR1 in disease;Signaling by FGFR2 in disease;Signaling by FGFR3 fusions in cancer;Signaling by FGFR3 point mutants in cancer;Signaling by FGFR4 in disease;Signaling by SCF-KIT;Signaling by cytosolic FGFR1 fusion mutants;Synthesis of PIPs at the plasma membrane;VEGFA-VEGFR2 Pathway;WNT mediated activation of DVL	PIK3R1 PIP5K1A	
Activated PKN1 stimulates transcription of AR (androgen receptor) regulated genes KLK2 and KLK3;Activation of anterior HOX genes in hindbrain development during early embryogenesis;Activation of rRNA Expression by ERCC6 (CSB) and EHMT2 (G9a);B-WICH complex positively regulates rRNA expression;Condensation of Prophase Chromosomes;DNA Damage/Telomere Stress Induced Senescence;DNA methylation;Deposition of new CENPA-containing nucleosomes at the centromere;Estrogen-dependent gene expression;Formation of the beta-catenin:TCF transactivating complex;G2/M DNA damage checkpoint;HDACs deacetylate histones;Meiotic recombination;Meiotic synapsis;NoRC negatively regulates rRNA expression;Nonhomologous End-Joining (NHEJ);Oxidative Stress Induced Senescence;PRC2 methylates histones and DNA;Packaging Of Telomere Ends;Processing of DNA double-strand break ends;RNA Polymerase I Chain Elongation;RNA Polymerase I Promoter Opening;RUNX1 regulates genes involved in megakaryocyte differentiation and platelet function;RUNX1 regulates transcription of genes involved in differentiation of HSCs;Recruitment and ATM-mediated phosphorylation of repair and signaling proteins at DNA double strand breaks;SIRT1 negatively regulates rRNA expression;Senescence-Associated Secretory Phenotype (SASP);Transcriptional regulation by small RNAs	HIST2H2BE NIPAL4	

Table B (continued)

Pathway	Regs Conn	Regs NConn
Activated PKN1 stimulates transcription of AR (androgen receptor) regulated genes KLK2 and KLK3;Activation of anterior HOX genes in hindbrain development during early embryogenesis;Activation of rRNA Expression by ERCC6 (CSB) and EHMT2 (G9a);B-WICH complex positively regulates rRNA expression;Condensation of Prophase Chromosomes;DNA Damage/Telomere Stress Induced Senescence;DNA methylation;Deposition of new CENPA-containing nucleosomes at the centromere;Estrogen-dependent gene expression;Formation of the beta-catenin:TCF transactivating complex;HDACs deacetylate histones;Meiotic recombination;Meiotic synapsis;NoRC negatively regulates rRNA expression;Oxidative Stress Induced Senescence;PRC2 methylates histones and DNA;Packaging Of Telomere Ends;RNA Polymerase I Chain Elongation;RNA Polymerase I Promoter Opening;RUNX1 regulates genes involved in megakaryocyte differentiation and platelet function;RUNX1 regulates transcription of genes involved in differentiation of HSCs;SIRT1 negatively regulates rRNA expression;Senescence-Associated Secretory Phenotype (SASP);Transcriptional regulation by small RNAs	HIST2H2BE RAB3A	
Activated PKN1 stimulates transcription of AR (androgen receptor) regulated genes KLK2 and KLK3;Activation of anterior HOX genes in hindbrain development during early embryogenesis;Activation of rRNA Expression by ERCC6 (CSB) and EHMT2 (G9a);B-WICH complex positively regulates rRNA expression;Condensation of Prophase Chromosomes;DNA Damage/Telomere Stress Induced Senescence;DNA methylation;Deposition of new CENPA-containing nucleosomes at the centromere;Estrogen-dependent gene expression;Formation of the beta-catenin:TCF transactivating complex;Meiotic recombination;Meiotic synapsis;NoRC negatively regulates rRNA expression;Oxidative Stress Induced Senescence;PRC2 methylates histones and DNA;Packaging Of Telomere Ends;RNA Polymerase I Chain Elongation;RNA Polymerase I Promoter Opening;RUNX1 regulates genes involved in megakaryocyte differentiation and platelet function;RUNX1 regulates transcription of genes involved in differentiation of HSCs;SIRT1 negatively regulates rRNA expression;Senescence-Associated Secretory Phenotype (SASP);Transcriptional regulation by small RNAs	HIST2H2BE	ZNF446

Table B (continued)

Pathway	Regs Conn	Regs NConn
Activated PKN1 stimulates transcription of AR (androgen receptor) regulated genes KLK2 and KLK3;Activation of anterior HOX genes in hindbrain development during early embryogenesis;Activation of rRNA Expression by ERCC6 (CSB) and EHMT2 (G9a);B-WICH complex positively regulates rRNA expression;Condensation of Prophase Chromosomes;DNA methylation;Estrogen-dependent gene expression;Formation of the beta-catenin:TCF transactivating complex;Meiotic recombination;NoRC negatively regulates rRNA expression;Oxidative Stress Induced Senescence;PRC2 methylates histones and DNA;RNA Polymerase I Chain Elongation;RNA Polymerase I Promoter Opening;RUNX1 regulates genes involved in megakaryocyte differentiation and platelet function;RUNX1 regulates transcription of genes involved in differentiation of HSCs;SIRT1 negatively regulates rRNA expression;Senescence-Associated Secretory Phenotype (SASP);Transcriptional regulation by small RNAs	HIST2H2BE	ZNF446
Activation of anterior HOX genes in hindbrain development during early embryogenesis;Deposition of new CENPA-containing nucleosomes at the centromere;PRC2 methylates histones and DNA	HIST2H2BE	LYL1
Activation of anterior HOX genes in hindbrain development during early embryogenesis;Estrogen-dependent gene expression	HIST2H2BE	POGZ
Activation of anterior HOX genes in hindbrain development during early embryogenesis;PRC2 methylates histones and DNA	HIST2H2BE	LYL1
Activation of ATR in response to replication stress;Activation of the pre-replicative complex;Assembly of the pre-replicative complex;Orc1 removal from chromatin		CXCL14<br>S100A14
Activation of E2F1 target genes at G1/S	DHFR	CXCL14
Activation of E2F1 target genes at G1/S;Cyclin E associated events during G1/S transition;G0 and Early G1;Polo-like kinase mediated events;Transcription of E2F targets under negative control by DREAM complex		LYL1<br>SEC62
Activation of rRNA Expression by ERCC6 (CSB) and EHMT2 (G9a);HDACs deacetylate histones;Oxidative Stress Induced Senescence;SIRT1 negatively regulates rRNA expression	HIST2H2BE	LYL1
Activation of rRNA Expression by ERCC6 (CSB) and EHMT2 (G9a);HDACs deacetylate histones;SIRT1 negatively regulates rRNA expression	HIST2H2BE<br>PARP10	
Activation of the pre-replicative complex		CXCL14<br>S100A14
adenine and adenosine salvage III;adenosine nucleotides degradation II;guanine and guanosine salvage I;guanosine nucleotides degradation III;purine nucleotides degradation II (aerobic);purine ribonucleosides degradation to ribose-1-phosphate;urate biosynthesis/inosine 5'-phosphate degradation;xanthine and xanthosine salvage	PCK1<br>PFKFB1	
Angiogenesis	PLCG2<br>PTK2	
Angiogenesis;PDGF signaling pathway	PIK3R1<br>PLCG2	

Table B (continued)

Pathway	Regs Conn	Regs NConn
Angiogenesis;VEGF signaling pathway	PLCG2<br>PTK2	
Antigen activates B Cell Receptor (BCR) leading to generation of second messengers;CD22 mediated BCR regulation	ARHGEF4	ATXN7L2
Arf1 pathway;BCR signaling pathway;CXCR4-mediated signaling events;Class I PI3K signaling events;E-cadherin signaling in keratinocytes;EPO signaling pathway;Endothelins;ErbB1 downstream signaling;FGF signaling pathway;Fc-epsilon receptor I signaling in mast cells;IL2 signaling events mediated by PI3K;IL8- and CXCR1-mediated signaling events;IL8- and CXCR2-mediated signaling events;LPA receptor mediated events;LPA4-mediated signaling events;Nephrin/Neph1 signaling in the kidney podocyte;Netrin-mediated signaling events;Nongenotropic Androgen signaling;PAR1-mediated thrombin signaling events;PDGFR-alpha signaling pathway;PDGFR-beta signaling pathway;Plasma membrane estrogen receptor signaling;Regulation of Ras family activation;Role of Calcineurin-dependent NFAT signaling in lymphocytes;Signaling events mediated by Hepatocyte Growth Factor Receptor (c-Met);Signaling events mediated by VEGFR1 and VEGFR2;TCR signaling in nave CD4+ T cells;TCR signaling in nave CD8+ T cells;Thromboxane A2 receptor signaling;Trk receptor signaling mediated by PI3K and PLC-gamma;VEGFR1 specific signals	PLCG2<br>SELPLG	
ATR signaling pathway	YWHAZ	RNF139
AURKA Activation by TPX2;Amplification of signal from unattached kinetochores via a MAD2 inhibitory signal;Anchoring of the basal body to the plasma membrane;Loss of Nlp from mitotic centrosomes;Loss of proteins required for interphase microtubule organizationfrom the centrosome;Mitotic Prometaphase;RHO GTPases Activate Formins;Recruitment of NuMA to mitotic centrosomes;Recruitment of mitotic centrosome proteins and complexes;Regulation of PLK1 Activity at G2/M Transition;Resolution of Sister Chromatid Cohesion;Separation of Sister Chromatids		MRPL54<br>SOX14
Aurora B signaling	RASA1	ATG10
B cell activation	PLCG2	ATXN7L2
BCR signaling pathway	PIK3R1<br>PLCG2	
BCR signaling pathway;Class I PI3K signaling events	PIK3R1<br>PLCG2	

Table B (continued)

Pathway	Regs Conn	Regs NConn
BCR signaling pathway;CXCR3-mediated signaling events;CXCR4-mediated signaling events;Calcium signaling in the CD4+ TCR pathway;Class I PI3K signaling events;Downstream signaling in nave CD8+ T cells;E-cadherin signaling in keratinocytes;Effects of Botulinum toxin;ErbB1 downstream signaling;ErbB2/ErbB3 signaling events;FGF signaling pathway;Fc-epsilon receptor I signaling in mast cells;IFN-gamma pathway;IL8- and CXCR1-mediated signaling events;IL8- and CXCR2-mediated signaling events;LPA receptor mediated events;Nephrin/Neph1 signaling in the kidney podocyte;Netrin-mediated signaling events;Noncanonical Wnt signaling pathway;Nongenotropic Androgen signaling;PAR1-mediated thrombin signaling events;PAR4-mediated thrombin signaling events;PDGFR-alpha signaling pathway;PDGFR-beta signaling pathway;Plasma membrane estrogen receptor signaling;Role of Calcineurin-dependent NFAT signaling in lymphocytes;Signaling events mediated by VEGFR1 and VEGFR2;TCR signaling in nave CD4+ T cells;TCR signaling in nave CD8+ T cells;Thromboxane A2 receptor signaling;Trk receptor signaling mediated by PI3K and PLC-gamma;VEGFR1 specific signals;Visual signal transduction: Cones;Visual signal transduction: Rods	ARHGEF4 PTK2	
Budding and maturation of HIV virion;Endosomal Sorting Complex Required For Transport (ESCRT)		ARRDC2 CDX1
Cleavage of Growing Transcript in the Termination Region;mRNA 3'-end processing		PROCR ZC3H3
Cleavage of Growing Transcript in the Termination Region;Transport of Mature mRNA derived from an Intron-Containing Transcript;U12 Dependent Splicing;mRNA 3'-end processing;pre-mRNA splicing		NPIPB15 SMOC2
coenzyme A biosynthesis	ALDH3A1	ANP32B
Complex I biogenesis		ALDH3A1 MRPL54
Complex I biogenesis;Respiratory electron transport		ALDH3A1 MRPL54
Condensation of Prophase Chromosomes;Recruitment and ATM-mediated phosphorylation of repair and signaling proteins at DNA double strand breaks	HIST2H2BE NIPAL4	
COPI-dependent Golgi-to-ER retrograde traffic	KDELR2	WDR49
COPI-independent Golgi-to-ER retrograde traffic;COPI-mediated anterograde transport;HSP90 chaperone cycle for SHRs;MHC class II antigen presentation	DCTN4	GIMAP2
CXCR4-mediated signaling events	PIK3R1 PTK2	
CXCR4-mediated signaling events;IL12 signaling mediated by STAT4;IL12-mediated signaling events;TCR signaling in nave CD4+ T cells		ATXN7L2 HSBP1
CXCR4-mediated signaling events;TCR signaling in nave CD4+ T cells		ATXN7L2 HSBP1
Cyclin A/B1/B2 associated events during G2/M transition	CCNH	HAMP
Cyclin E associated events during G1/S transition	CCNH MYC	

Table B (continued)

Pathway	Regs Conn	Regs NConn
DAP12 signaling	PIK3R1<br>PLCG2	
Degradation of AXIN	BCAN<br>PSMB10	
Degradation of beta-catenin by the destruction complex	POLD3<br>PSMB10	
Degradation of DVL	DVL3<br>PSMB10	
Degradation of DVL;Hedgehog 'on' state	BCAN<br>PSMB10	
DNA Damage/Telomere Stress Induced Senescence;G2/M DNA damage checkpoint;HDR through Homologous Recombination (HRR);HDR through MMEJ (alt-NHEJ);HDR through Single Strand Annealing (SSA);Homologous DNA Pairing and Strand Exchange;Meiotic recombination;Nonhomologous End-Joining (NHEJ);Presynaptic phase of homologous DNA pairing and strand exchange;Processing of DNA double-strand break ends;Recruitment and ATM-mediated phosphorylation of repair and signaling proteins at DNA double strand breaks;Regulation of TP53 Activity through Phosphorylation;Resolution of D-loop Structures through Holliday Junction Intermediates;Resolution of D-loop Structures through Synthesis-Dependent Strand Annealing (SDSA);Sensing of DNA Double Strand Breaks	RNF139	PAM
DNA Damage/Telomere Stress Induced Senescence;Meiotic synapsis;Packaging Of Telomere Ends	HIST2H2BE<br>SSB	
Downstream signaling in nave CD8+ T cells;IL2-mediated signaling events;SHP2 signaling	PIK3R1<br>PLCG2	
Downstream TCR signaling;Generation of second messenger molecules;Interferon gamma signaling;MHC class II antigen presentation;PD-1 signaling;Phosphorylation of CD3 and TCR zeta chains;Translocation of ZAP-70 to Immunological synapse	HSBP1	POGZ
Dual Incision in GG-NER;Dual incision in TC-NER;Gap-filling DNA repair synthesis and ligation in TC-NER	POLK	TNNT2

Table B (continued)

Pathway	Regs Conn	Regs NConn
Dual incision in TC-NER;Formation of HIV elongation complex in the absence of HIV Tat;Formation of HIV-1 elongation complex containing HIV-1 Tat;Formation of Incision Complex in GG-NER;Formation of RNA Pol II elongation complex;Formation of TC-NER Pre-Incision Complex;Formation of the Early Elongation Complex;Formation of the HIV-1 Early Elongation Complex;Gap-filling DNA repair synthesis and ligation in TC-NER;HIV Transcription Initiation;NoRC negatively regulates rRNA expression;RNA Pol II CTD phosphorylation and interaction with CE;RNA Pol II CTD phosphorylation and interaction with CE during HIV infection;RNA Polymerase I Chain Elongation;RNA Polymerase I Promoter Escape;RNA Polymerase I Transcription Initiation;RNA Polymerase I Transcription Termination;RNA Polymerase II HIV Promoter Escape;RNA Polymerase II Pre-transcription Events;RNA Polymerase II Promoter Escape;RNA Polymerase II Transcription Elongation;RNA Polymerase II Transcription Initiation;RNA Polymerase II Transcription Initiation And Promoter Clearance;RNA Polymerase II Transcription Pre-Initiation And Promoter Opening;TP53 Regulates Transcription of DNA Repair Genes;Tat-mediated elongation of the HIV-1 transcript;Transcription of the HIV genome;Transcription-Coupled Nucleotide Excision Repair (TC-NER);mRNA Capping	CCNH	DAPK3
Dual incision in TC-NER;Formation of HIV elongation complex in the absence of HIV Tat;Formation of HIV-1 elongation complex containing HIV-1 Tat;Formation of RNA Pol II elongation complex;Formation of TC-NER Pre-Incision Complex;Gap-filling DNA repair synthesis and ligation in TC-NER;HIV Transcription Initiation;NoRC negatively regulates rRNA expression;RNA Pol II CTD phosphorylation and interaction with CE;RNA Pol II CTD phosphorylation and interaction with CE during HIV infection;RNA Polymerase I Chain Elongation;RNA Polymerase I Promoter Escape;RNA Polymerase I Transcription Initiation;RNA Polymerase I Transcription Termination;RNA Polymerase II HIV Promoter Escape;RNA Polymerase II Pre-transcription Events;RNA Polymerase II Promoter Escape;RNA Polymerase II Transcription Elongation;RNA Polymerase II Transcription Initiation;RNA Polymerase II Transcription Initiation And Promoter Clearance;RNA Polymerase II Transcription Pre-Initiation And Promoter Opening;TP53 Regulates Transcription of DNA Repair Genes;Tat-mediated elongation of the HIV-1 transcript;Transcription of the HIV genome;Transcription-Coupled Nucleotide Excision Repair (TC-NER);mRNA Capping	CCNH	DAPK3
Dual incision in TC-NER;Formation of Incision Complex in GG-NER;Formation of TC-NER Pre-Incision Complex;Gap-filling DNA repair synthesis and ligation in TC-NER;Transcription-Coupled Nucleotide Excision Repair (TC-NER)	CCNH	DAPK3
Dual incision in TC-NER;Formation of TC-NER Pre-Incision Complex;Gap-filling DNA repair synthesis and ligation in TC-NER;Neddylation;Transcription-Coupled Nucleotide Excision Repair (TC-NER)	BCAN	C14orf1
E3 ubiquitin ligases ubiquitinate target proteins	DERL1	HOXD9

Table B (continued)

Pathway	Regs Conn	Regs NConn
E-cadherin signaling in keratinocytes	PIK3R1<br>PIP5K1A	
EGF receptor signaling pathway	PEBP4<br>PLCG2	
EGF receptor signaling pathway;FGF signaling pathway	PLCG2	POGZ
EPHA2 forward signaling	PIK3R1<br>PTK2	
Eukaryotic Translation Termination;Formation of a pool of free 40S subunits;GTP hydrolysis and joining of the 60S ribosomal subunit;L13a-mediated translational silencing of Ceruloplasmin expression;Nonsense Mediated Decay (NMD) enhanced by the Exon Junction Complex (EJC);Nonsense Mediated Decay (NMD) independent of the Exon Junction Complex (EJC);Peptide chain elongation;Regulation of expression of SLITs and ROBOs;SRP-dependent cotranslational protein targeting to membrane;Selenocysteine synthesis;Viral mRNA Translation	RPS23<br>RPS4X	
Fanconi Anemia Pathway;Gap-filling DNA repair synthesis and ligation in GG-NER;Gap-filling DNA repair synthesis and ligation in TC-NER;HDR through Homologous Recombination (HRR);HDR through MMEJ (alt-NHEJ);Processing of DNA double-strand break ends;Translesion Synthesis by POLH;Translesion synthesis by POLI;Translesion synthesis by POLK;Translesion synthesis by REV1	RNF139	PAM
Fanconi Anemia Pathway;HDR through MMEJ (alt-NHEJ);Processing of DNA double-strand break ends	RNF139	PAM
fatty acid activation;fatty acid alpha-oxidation II;fatty acid beta-oxidation I;glutaryl-CoA degradation;ketogenesis;ketolysis;mevalonate pathway I;stearate biosynthesis I (animals);superpathway of cholesterol biosynthesis;superpathway of geranylgeranyldiphosphate biosynthesis I (via mevalonate);tryptophan degradation III (eukaryotic)	ALDH3A1	TSPAN15
fatty acid beta-oxidation I;glutaryl-CoA degradation;ketogenesis;ketolysis;mevalonate pathway I;superpathway of cholesterol biosynthesis;superpathway of geranylgeranyldiphosphate biosynthesis I (via mevalonate);tryptophan degradation III (eukaryotic)	ALDH3A1<br>MLYCD	
fatty acid biosynthesis initiation II	MLYCD	TERF2IP
FBXL7 down-regulates AURKA during mitotic entry and in early mitosis	PSMB10	PMP22
Fc-epsilon receptor I signaling in mast cells	PIK3R1<br>PTK2	
FGF signaling pathway	PLCG2	ARHGEF4
Formation of a pool of free 40S subunits	RPL13A<br>RPS23	
Formation of Incision Complex in GG-NER	CCNH	KRTAP15-1
Formation of the beta-catenin:TCF transactivating complex;Ub-specific processing proteases	HIST2H2BE	POGZ
G2/M DNA damage checkpoint;Recruitment and ATM-mediated phosphorylation of repair and signaling proteins at DNA double strand breaks	HIST2H2BE<br>TMOD4	

Table B (continued)

Pathway	Regs Conn	Regs NConn
G alpha (s) signalling events		ETV7 MNS1
Gap-filling DNA repair synthesis and ligation in GG-NER;Gap-filling DNA repair synthesis and ligation in TC-NER	CCNH POLK	
Gap-filling DNA repair synthesis and ligation in GG-NER;Gap-filling DNA repair synthesis and ligation in TC-NER;HDR through Homologous Recombination (HRR);HDR through MMEJ (alt-NHEJ);PCNA-Dependent Long Patch Base Excision Repair;Processing of DNA double-strand break ends;Termination of translesion DNA synthesis	RNF139	PAM
Gap-filling DNA repair synthesis and ligation in GG-NER;Gap-filling DNA repair synthesis and ligation in TC-NER;HDR through Homologous Recombination (HRR);HDR through MMEJ (alt-NHEJ);PCNA-Dependent Long Patch Base Excision Repair;Processing of DNA double-strand break ends;Termination of translesion DNA synthesis;Translesion Synthesis by POLH;Translesion synthesis by POLI;Translesion synthesis by POLK;Translesion synthesis by REV1	RNF139	PAM
Gap-filling DNA repair synthesis and ligation in TC-NER	CCNH POLK	
G beta:gamma signalling through PI3Kgamma;Interleukin receptor SHC signaling;Interleukin-3, 5 and GM-CSF signaling	PIK3R1	MCMDC2
gluconeogenesis I;glycolysis I	PCK1 PFKFB1	
glycogen degradation II;glycogen degradation III	PCK1 PFKFB1	
GMCSF-mediated signaling events	GAB2 PIK3R1	
GTP hydrolysis and joining of the 60S ribosomal subunit;L13a-mediated translational silencing of Ceruloplasmin expression;Nonsense Mediated Decay (NMD) enhanced by the Exon Junction Complex (EJC);Nonsense Mediated Decay (NMD) independent of the Exon Junction Complex (EJC);Regulation of expression of SLITs and ROBOs;Ribosomal scanning and start codon recognition;Translation initiation complex formation	RPS23 RPS4X	
HDR through Homologous Recombination (HRR);Homologous DNA Pairing and Strand Exchange;Presynaptic phase of homologous DNA pairing and strand exchange;Resolution of D-loop Structures through Holliday Junction Intermediates;Resolution of D-loop Structures through Synthesis-Dependent Strand Annealing (SDSA)	RAD51D	SULT4A1
HDR through MMEJ (alt-NHEJ)	RNF139	PAM
HDR through Single Strand Annealing (SSA);Meiotic recombination;Presynaptic phase of homologous DNA pairing and strand exchange;Processing of DNA double-strand break ends;Regulation of TP53 Activity through Phosphorylation	TMOD4	HOXD9
Heterotrimeric G-protein signaling pathway-Gi alpha and Gs alpha mediated pathway;Muscarinic acetylcholine receptor 2 and 4 signaling pathway		EDN3 MNS1

Table B (continued)

Pathway	Regs Conn	Regs NConn
Inflammation mediated by chemokine and cytokine signaling pathway;Interleukin signaling pathway;PDGF signaling pathway;VEGF signaling pathway	PIK3R1	DCP2
ketogenesis;mevalonate pathway I;superpathway of cholesterol biosynthesis;superpathway of geranylgeranyldiphosphate biosynthesis I (via mevalonate)	ALDH3A1 MLYCD	
L13a-mediated translational silencing of Ceruloplasmin expression;Nonsense Mediated Decay (NMD) enhanced by the Exon Junction Complex (EJC);Nonsense Mediated Decay (NMD) independent of the Exon Junction Complex (EJC);Regulation of expression of SLITs and ROBOs;Translation initiation complex formation	RPS23 RPS4X	
L13a-mediated translational silencing of Ceruloplasmin expression;Translation initiation complex formation	RPS23 RPS4X	
Mitochondrial protein import	PAM16	SLC39A13
Mitochondrial translation termination	MRPL54 MRPS28	
Muscarinic acetylcholine receptor 2 and 4 signaling pathway		EDN3 MNS1
Nonhomologous End-Joining (NHEJ)	HIST2H2BE XRCC4	
Nonsense Mediated Decay (NMD) enhanced by the Exon Junction Complex (EJC);Nonsense Mediated Decay (NMD) independent of the Exon Junction Complex (EJC)	RPS23 RPS4X	
NoRC negatively regulates rRNA expression;RNA Polymerase I Chain Elongation;RNA Polymerase I Promoter Escape;RNA Polymerase I Transcription Initiation;RNA Polymerase I Transcription Termination	CCNH SYT11	
Osteopontin-mediated events	PIK3R1 PIP5K1A	
PD-1 signaling	ARHGEF4	MNS1
PI5P, PP2A and IER3 Regulate PI3K/AKT Signaling;Synthesis of PIPs at the plasma membrane	PIP5K1A	GALE
Prefoldin mediated transfer of substrate to CCT/TriC	AGO2	FAM212B
Regulation of expression of SLITs and ROBOs	ISL1 PSMB10	
Regulation of ornithine decarboxylase (ODC)	AZIN1 PSMB10	
Regulation of signaling by CBL	CX3CL1 PIK3R1	
RHO GTPases Activate WASPs and WAVEs;Regulation of actin dynamics for phagocytic cup formation	PTK2	RFTN1
RMTs methylate histone arginines	NR3C2 RASA1	
RNA Polymerase III Abortive And Retractive Initiation;RNA Polymerase III Transcription Initiation From Type 3 Promoter	BDP1 SNAPC2	
Role of phospholipids in phagocytosis	PIK3R1 PLCG2	
Role of second messengers in netrin-1 signaling	TERF2IP	INTS8

Table B (continued)

Pathway	Regs Conn	Regs NConn
RUNX1 regulates genes involved in megakaryocyte differentiation and platelet function;RUNX1 regulates transcription of genes involved in differentiation of HSCs	HIST2H2BE	POGZ
RUNX1 regulates transcription of genes involved in differentiation of HSCs	CCNH<br>HIST2H2BE	
S-adenosyl-L-methionine biosynthesis	CES2<br>POLD3	
salvage pathways of pyrimidine ribonucleotides	PCK1<br>PFKFB1	
Signaling events mediated by Hepatocyte Growth Factor Receptor (c-Met)	ARHGEF4<br>PTK2	
S-methyl-5'-thioadenosine degradation II	PCK1<br>PFKFB1	
Termination of translesion DNA synthesis	POLK	MYO1D
Transcriptional Regulation by E2F6	PHC3	CXCL14
Transport of Mature mRNA Derived from an Intronless Transcript		GUCA2B<br>ZCCHC9
UDP-N-acetyl-D-galactosamine biosynthesis II;UDP-N-acetyl-D-glucosamine biosynthesis II	ALDH3A1<br>MLYCD	
2-oxobutanoate degradation I	KCNK7	
3-phosphoinositide degradation;gluconeogenesis I;glycolysis I	PCK1	
3-phosphoinositide degradation;salvage pathways of pyrimidine ribonucleotides	PCK1	
5HT2 type receptor mediated signaling pathway;Angiogenesis;Angiotensin_II-stimulated_signaling_through_G_proteins_and_beta-arrestin;B cell activation;EGF receptor signaling pathway;FGF signaling pathway;Histamine H1 receptor mediated signaling pathway;Metabotropic glutamate receptor group I pathway;Muscarinic acetylcholine receptor 1 and 3 signaling pathway;Oxytocin receptor mediated signaling pathway;T cell activation;Thyrotropin-releasing hormone receptor signaling pathway	PLCG2	
5HT2 type receptor mediated signaling pathway;Angiogenesis;EGF receptor signaling pathway;FGF signaling pathway;Histamine H1 receptor mediated signaling pathway;Inflammation mediated by chemokine and cytokine signaling pathway;Oxytocin receptor mediated signaling pathway;PDGF signaling pathway;VEGF signaling pathway;untitled	PLCG2	
5HT2 type receptor mediated signaling pathway;Angiogenesis;EGF receptor signaling pathway;FGF signaling pathway;Histamine H1 receptor mediated signaling pathway;Oxytocin receptor mediated signaling pathway	PLCG2	
a6b1 and a6b4 Integrin signaling	PMP22	

Table B (continued)

Pathway	Regs Conn	Regs NConn
ABC-family proteins mediated transport;APC/C:Cdc20 mediated degradation of Securin;APC/C:Cdh1 mediated degradation of Cdc20 and other APC/C:Cdh1 targeted proteins in late mitosis/early G1;AUF1 (hnRNP D0) binds and destabilizes mRNA;Activation of NF-kappaB in B cells;Antigen processing: Ubiquitination and Proteasome degradation;Asymmetric localization of PCP proteins;Autodegradation of the E3 ubiquitin ligase COP1;CDK-mediated phosphorylation and removal of Cdc6;CDT1 association with the CDC6:ORC:origin complex;Cdc20:Phospho-APC/C mediated degradation of Cyclin A;Cross-presentation of soluble exogenous antigens (endosomes);Dectin-1 mediated noncanonical NF-kB signaling;Defective CFTR causes cystic fibrosis;Degradation of AXIN;Degradation of DVL;Degradation of GLI1 by the proteasome;Degradation of GLI2 by the proteasome;Degradation of beta-catenin by the destruction complex;ER-Phagosome pathway;FBXL7 down-regulates AURKA during mitotic entry and in early mitosis;FCERI mediated NF-kB activation;G2/M Checkpoints;GLI3 is processed to GLI3R by the proteasome;Hedgehog 'on' state;Hedgehog ligand biogenesis;Hh mutants that don't undergo autocatalytic processing are degraded by ERAD;Interleukin-1 signaling;MAPK6/MAPK4 signaling;Neddylation;Orc1 removal from chromatin;Oxygen-dependent proline hydroxylation of Hypoxia-inducible Factor Alpha;RUNX1 regulates transcription of genes involved in differentiation of HSCs;Regulation of PTEN stability and activity;Regulation of RAS by GAPs;Regulation of RUNX2 expression and activity;Regulation of activated PAK-2p34 by proteasome mediated degradation;Regulation of expression of SLITs and ROBOs;Regulation of ornithine decarboxylase (ODC);SCF-beta-TrCP mediated degradation of Emi1;TNFR2 non-canonical NF-kB pathway;UCH proteinases;Ub-specific processing proteases;Ubiquitin Mediated Degradation of Phosphorylated Cdc25A;Ubiquitin-dependent degradation of Cyclin D1;Vif-mediated degradation of APOBEC3G;Vpu mediated degradation of CD4	PSMB10	
ABC-family proteins mediated transport;APC/C:Cdh1 mediated degradation of Cdc20 and other APC/C:Cdh1 targeted proteins in late mitosis/early G1;Autodegradation of Cdh1 by Cdh1:APC/C;Autodegradation of the E3 ubiquitin ligase COP1;CDK-mediated phosphorylation and removal of Cdc6;CDT1 association with the CDC6:ORC:origin complex;Cdc20:Phospho-APC/C mediated degradation of Cyclin A;Defective CFTR causes cystic fibrosis;Degradation of beta-catenin by the destruction complex;Interleukin-1 signaling;Oxygen-dependent proline hydroxylation of Hypoxia-inducible Factor Alpha;SCF(Skp2)-mediated degradation of p27/p21;SCF-beta-TrCP mediated degradation of Emi1;Ubiquitin Mediated Degradation of Phosphorylated Cdc25A;Ubiquitin-dependent degradation of Cyclin D1;Vif-mediated degradation of APOBEC3G;Vpu mediated degradation of CD4	PSMB10	

Table B (continued)

Pathway	Regs Conn	Regs NConn
ABC-family proteins mediated transport;ATP sensitive Potassium channels;Activation of G protein gated Potassium channels;Ca activated K+ channels;Classical Kir channels;Defective ABCC8 can cause hypoglycemias and hyperglycemias;HCN channels;Inhibition of voltage gated Ca2+ channels via Gbeta/gamma subunits;Ion homeostasis;Ion transport by P-type ATPases;Phase 1 - inactivation of fast Na+ channels;Phase 2 - plateau phase;Phase 3 - rapid repolarisation;Phase 4 - resting membrane potential;Potassium transport channels;Sperm Motility And Taxes;TWIK related potassium channel (TREK);TWIK-related alkaline pH activated K+ channel (TALK);TWIK-related spinal cord K+ channel (TRESK);TWIK-releated acid-sensitive K+ channel (TASK);Tandem of pore domain in a weak inwardly rectifying K+ channels (TWIK);Tandem pore domain halothane-inhibited K+ channel (THIK);The NLRP3 inflammasome;Voltage gated Potassium channels	PGK2	
ABC-family proteins mediated transport;Defective CFTR causes cystic fibrosis	DERL1	
Abortive elongation of HIV-1 transcript in the absence of Tat;Formation of HIV elongation complex in the absence of HIV Tat;Formation of HIV-1 elongation complex containing HIV-1 Tat;Formation of RNA Pol II elongation complex;Formation of the Early Elongation Complex;Formation of the HIV-1 Early Elongation Complex;HIV elongation arrest and recovery;Pausing and recovery of HIV elongation;Pausing and recovery of Tat-mediated HIV elongation;RNA Polymerase II Pre-transcription Events;RNA Polymerase II Transcription Elongation;TP53 Regulates Transcription of DNA Repair Genes;Tat-mediated HIV elongation arrest and recovery;Tat-mediated elongation of the HIV-1 transcript	NTRK1	
acetate conversion to acetyl-CoA;ethanol degradation II;ethanol degradation IV;oxidative ethanol degradation III	PIK3R1	

Table B (continued)

Pathway	Regs Conn	Regs NConn
Acetylcholine Neurotransmitter Release Cycle;Activation of AMPA receptors;Activation of Na-permeable kainate receptors;Activation of the phototransduction cascade;Amine compound SLC transporters;Amino acid and oligopeptide SLC transporters;Amino acid transport across the plasma membrane;Bicarbonate transporters;Bile salt and organic anion SLC transporters;Biotin transport and metabolism;Cation-coupled Chloride cotransporters;Cellular hexose transport;Clearance of dopamine;Clearance of seratonin;Creatine metabolism;Defective SLC24A5 causes oculocutaneous albinism 6 (OCA6);Defective SLC4A4 causes renal tubular acidosis, proximal, with ocular abnormalities and mental retardation (pRTA-OA);Defective SLC9A6 causes X-linked, syndromic mental retardation,, Christianson type (MRXSCH);Defective SLC9A9 causes autism 16 (AUTS16);Elevation of cytosolic Ca2+ levels;Glutamate Neurotransmitter Release Cycle;Highly sodium permeable acetylcholine nicotinic receptors;Import of palmitoyl-CoA into the mitochondrial matrix;Inositol transporters;Intestinal hexose absorption;Ion homeostasis;Ion transport by P-type ATPases;Mitochondrial calcium ion transport;Multifunctional anion exchangers;Na+/Cl-dependent neurotransmitter transporters;Neurotransmitter receptors and postsynaptic signal transmission;Nicotinamide salvaging;Organic anion transporters;Organic cation transport;Phase 0 - rapid depolarisation;Recycling of bile acids and salts;Reduction of cytosolic Ca++ levels;Reuptake of GABA;Sodium-coupled phosphate cotransporters;Sodium-coupled sulphate, di- and tri-carboxylate transporters;Sodium/Calcium exchangers;Sodium/Proton exchangers;Stimuli-sensing channels;Surfactant metabolism;Synthesis of PC;TRP channels;Thyroxine biosynthesis;Transport of bile salts and organic acids, metal ions and amine compounds;Transport of inorganic cations/anions and amino acids/oligopeptides;Transport of nucleosides and free purine and pyrimidine bases across the plasma membrane;Transport of vitamins, nucleosides, and related molecules;Type II Na+/Pi cotransporters;Unblocking of NMDA receptor, glutamate binding and activation;Vitamin B5 (pantothenate) metabolism;Vitamin C (ascorbate) metabolism		HTN1

Table B (continued)

Pathway	Regs Conn	Regs NConn
Acetylcholine regulates insulin secretion;Activated NTRK2 signals through PI3K;Activation of PKB;Antigen activates B Cell Receptor (BCR) leading to generation of second messengers;CD28 dependent PI3K/Akt signaling;CD28 dependent Vav1 pathway;CLEC7A (Dectin-1) signaling;Ca2+ pathway;Cargo recognition for clathrin-mediated endocytosis;Clathrin-mediated endocytosis;Constitutive Signaling by AKT1 E17K in Cancer;Constitutive Signaling by Aberrant PI3K in Cancer;Constitutive Signaling by EGFRvIII;Constitutive Signaling by Ligand-Responsive EGFR Cancer Variants;DAG and IP3 signaling;DAP12 signaling;DCC mediated attractive signaling;Downstream TCR signaling;Downstream signal transduction;EGFR downregulation;EPHB-mediated forward signaling;ER-Phagosome pathway;FCERI mediated Ca+2 mobilization;FCERI mediated MAPK activation;Fatty Acids bound to GPR40 (FFAR1) regulate insulin secretion;G alpha (q) signalling events;G beta:gamma signalling through PI3Kgamma;G beta:gamma signalling through PLC beta;GAB1 signalosome;GPVI-mediated activation cascade;GRB2:SOS provides linkage to MAPK signaling for Integrins;Generation of second messenger molecules;IRAK4 deficiency (TLR2/4);Inhibition of TSC complex formation by PKB;Integrin alphaIIb beta3 signaling;Invadopodia formation;MET activates PI3K/AKT signaling;MyD88 deficiency (TLR2/4);MyD88:Mal cascade initiated on plasma membrane;Negative regulation of the PI3K/AKT network;Netrin-1 signaling;PDE3B signalling;PI Metabolism;PI-3K cascade:FGFR1;PI-3K cascade:FGFR2;PI-3K cascade:FGFR3;PI-3K cascade:FGFR4;PI3K Cascade;PI3K events in ERBB2 signaling;PI3K events in ERBB4 signaling;PI3K/AKT Signaling;PI3K/AKT activation;PI5P, PP2A and IER3 Regulate PI3K/AKT Signaling;PIPs transport between plasma and early endosome membranes;PLC beta mediated events;PTEN Loss of Function in Cancer;Phospholipase C-mediated cascade: FGFR1;Phospholipase C-mediated cascade; FGFR2;Phospholipase C-mediated cascade; FGFR3;Phospholipase C-mediated cascade; FGFR4;RHO GTPases Activate WASPs and WAVEs;RHO GTPases activate PKNs;RIPK1-mediated regulated necrosis;Recycling pathway of L1;Regulation of TP53 Degradation;Regulation of actin dynamics for phagocytic cup formation;Role of LAT2/NTAL/LAB on calcium mobilization;Role of phospholipids in phagocytosis;Role of second messengers in netrin-1 signaling;Signaling by FGFR1 in disease;Signaling by FGFR2 in disease;Signaling by FGFR3 fusions in cancer;Signaling by FGFR3 point mutants in cancer;Signaling by FGFR4 in disease;Signaling by SCF-KIT;Signaling by cytosolic FGFR1 fusion mutants;Syndecan interactions;Synthesis of IP3 and IP4 in the cytosol;Synthesis of PIPs at the plasma membrane;VEGFA-VEGFR2 Pathway;VEGFR2 mediated cell proliferation;VEGFR2 mediated vascular permeability;WNT mediated activation of DVL;p130Cas linkage to MAPK signaling for integrins	PIP5K1A	

Table B (continued)

Pathway	Regs Conn	Regs NConn
Acetylcholine regulates insulin secretion;Activated NTRK2 signals through PI3K;Antigen activates B Cell Receptor (BCR) leading to generation of second messengers;CD28 dependent PI3K/Akt signaling;CLEC7A (Dectin-1) signaling;Ca2+ pathway;Cargo recognition for clathrin-mediated endocytosis;Clathrin-mediated endocytosis;Constitutive Signaling by AKT1 E17K in Cancer;Constitutive Signaling by Aberrant PI3K in Cancer;Constitutive Signaling by EGFRvIII;Constitutive Signaling by Ligand-Responsive EGFR Cancer Variants;DAG and IP3 signaling;DAP12 signaling;DCC mediated attractive signaling;Downstream TCR signaling;Downstream signal transduction;EGFR downregulation;EPHB-mediated forward signaling;ER-Phagosome pathway;FCERI mediated Ca+2 mobilization;Fatty Acids bound to GPR40 (FFAR1) regulate insulin secretion;G alpha (q) signalling events;G beta:gamma signalling through PI3Kgamma;G beta:gamma signalling through PLC beta;GAB1 signalosome;GPVI-mediated activation cascade;GRB2:SOS provides linkage to MAPK signaling for Integrins;Generation of second messenger molecules;IRAK4 deficiency (TLR2/4);Integrin alphaIIb beta3 signaling;Invadopodia formation;MET activates PI3K/AKT signaling;MyD88 deficiency (TLR2/4);MyD88:Mal cascade initiated on plasma membrane;Negative regulation of the PI3K/AKT network;Netrin-1 signaling;PI Metabolism;PI-3K cascade:FGFR1;PI-3K cascade:FGFR2;PI-3K cascade:FGFR3;PI-3K cascade:FGFR4;PI3K Cascade;PI3K events in ERBB2 signaling;PI3K events in ERBB4 signaling;PI3K/AKT Signaling;PI3K/AKT activation;PI5P, PP2A and IER3 Regulate PI3K/AKT Signaling;PIPs transport between plasma and early endosome membranes;PLC beta mediated events;RHO GTPases Activate WASPs and WAVEs;RIPK1-mediated regulated necrosis;Recycling pathway of L1;Regulation of actin dynamics for phagocytic cup formation;Role of LAT2/NTAL/LAB on calcium mobilization;Role of phospholipids in phagocytosis;Role of second messengers in netrin-1 signaling;Signaling by FGFR1 in disease;Signaling by FGFR2 in disease;Signaling by FGFR3 fusions in cancer;Signaling by FGFR3 point mutants in cancer;Signaling by FGFR4 in disease;Signaling by SCF-KIT;Signaling by cytosolic FGFR1 fusion mutants;Syndecan interactions;Synthesis of IP3 and IP4 in the cytosol;Synthesis of PIPs at the plasma membrane;VEGFA-VEGFR2 Pathway;VEGFR2 mediated cell proliferation;WNT mediated activation of DVL;p130Cas linkage to MAPK signaling for integrins	PIP5K1A	

Table B (continued)

Pathway	Regs Conn	Regs NConn
Acetylcholine regulates insulin secretion;Antigen activates B Cell Receptor (BCR) leading to generation of second messengers;CLEC7A (Dectin-1) induces NFAT activation;CLEC7A (Dectin-1) signaling;Ca2+ pathway;DAG and IP3 signaling;Effects of PIP2 hydrolysis;FCERI mediated Ca+2 mobilization;Fatty Acids bound to GPR40 (FFAR1) regulate insulin secretion;G alpha (q) signalling events;G beta:gamma signalling through PLC beta;GPVI-mediated activation cascade;Generation of second messenger molecules;IP3 and IP4 transport between cytosol and nucleus;IPs transport between ER lumen and cytosol;Ion homeostasis;PLC beta mediated events;Regulation of insulin secretion;Role of phospholipids in phagocytosis;Role of second messengers in netrin-1 signaling;Synthesis of IP2, IP, and Ins in the cytosol;Synthesis of IP3 and IP4 in the cytosol;VEGFR2 mediated cell proliferation	PLCG2	
Acetylcholine regulates insulin secretion;Antigen activates B Cell Receptor (BCR) leading to generation of second messengers;CLEC7A (Dectin-1) signaling;Ca2+ pathway;DAG and IP3 signaling;FCERI mediated Ca+2 mobilization;Fatty Acids bound to GPR40 (FFAR1) regulate insulin secretion;G beta:gamma signalling through PLC beta;GPVI-mediated activation cascade;Generation of second messenger molecules;PI5P, PP2A and IER3 Regulate PI3K/AKT Signaling;PLC beta mediated events;Role of phospholipids in phagocytosis;Role of second messengers in netrin-1 signaling;Synthesis of IP3 and IP4 in the cytosol;Synthesis of PIPs at the plasma membrane;VEGFR2 mediated cell proliferation	PLCG2	
Acetylcholine regulates insulin secretion;CLEC7A (Dectin-1) signaling;Clathrin-mediated endocytosis;DAG and IP3 signaling;FCERI mediated Ca+2 mobilization;Fatty Acids bound to GPR40 (FFAR1) regulate insulin secretion;Generation of second messenger molecules;PI5P, PP2A and IER3 Regulate PI3K/AKT Signaling;PLC beta mediated events;Role of phospholipids in phagocytosis;Role of second messengers in netrin-1 signaling;Synthesis of IP3 and IP4 in the cytosol;Synthesis of PIPs at the plasma membrane;VEGFR2 mediated cell proliferation;WNT mediated activation of DVL	PIP5K1A	
Activated NOTCH1 Transmits Signal to the Nucleus		FAM83E
Activated NOTCH1 Transmits Signal to the Nucleus;Constitutive Signaling by NOTCH1 HD Domain Mutants;Constitutive Signaling by NOTCH1 HD+PEST Domain Mutants;Constitutive Signaling by NOTCH1 PEST Domain Mutants;NOTCH2 Activation and Transmission of Signal to the Nucleus;NOTCH3 Activation and Transmission of Signal to the Nucleus;Receptor-ligand binding initiates the second proteolytic cleavage of Notch receptor;Signaling by NOTCH4		ZBTB43

Table B (continued)

Pathway	Regs Conn	Regs NConn
Activated NTRK2 signals through FRS2 and FRS3;Activated NTRK2 signals through RAS;CD209 (DC-SIGN) signaling;Constitutive Signaling by EGFRvIII;Constitutive Signaling by Ligand-Responsive EGFR Cancer Variants;DAP12 signaling;Downstream signal transduction;EGFR Transactivation by Gastrin;FCERI mediated MAPK activation;FRS-mediated FGFR1 signaling;FRS-mediated FGFR2 signaling;FRS-mediated FGFR3 signaling;FRS-mediated FGFR4 signaling;GRB2 events in EGFR signaling;GRB2 events in ERBB2 signaling;Insulin receptor signalling cascade;MET activates RAS signaling;NCAM signaling for neurite out-growth;RAF/MAP kinase cascade;SHC-mediated cascade:FGFR1;SHC-mediated cascade:FGFR2;SHC-mediated cascade:FGFR3;SHC-mediated cascade:FGFR4;SHC-related events triggered by IGF1R;SHC1 events in EGFR signaling;SHC1 events in ERBB2 signaling;SHC1 events in ERBB4 signaling;SOS-mediated signalling;Signaling by FGFR1 in disease;Signaling by FGFR2 in disease;Signaling by FGFR3 fusions in cancer;Signaling by FGFR3 point mutants in cancer;Signaling by FGFR4 in disease;Signaling by SCF-KIT;Signalling to RAS;Tie2 Signaling	PTK2	
Activated NTRK2 signals through PI3K	PIK3R1	
Activated NTRK2 signals through PI3K;Antigen activates B Cell Receptor (BCR) leading to generation of second messengers;CD28 dependent PI3K/Akt signaling;CLEC7A (Dectin-1) signaling;Ca2+ pathway;Clathrin-mediated endocytosis;Constitutive Signaling by Aberrant PI3K in Cancer;Constitutive Signaling by EGFRvIII;Constitutive Signaling by Ligand-Responsive EGFR Cancer Variants;DAG and IP3 signaling;DAP12 signaling;Downstream TCR signaling;Downstream signal transduction;FCERI mediated Ca+2 mobilization;G beta:gamma signalling through PI3Kgamma;G beta:gamma signalling through PLC beta;GAB1 signalosome;GPVI-mediated activation cascade;Generation of second messenger molecules;MET activates PI3K/AKT signaling;PI-3K cascade:FGFR1;PI-3K cascade:FGFR2;PI-3K cascade:FGFR3;PI-3K cascade:FGFR4;PI3K Cascade;PI3K events in ERBB2 signaling;PI3K events in ERBB4 signaling;PI3K/AKT Signaling;PI3K/AKT activation;PI5P, PP2A and IER3 Regulate PI3K/AKT Signaling;PLC beta mediated events;Role of LAT2/NTAL/LAB on calcium mobilization;Role of phospholipids in phagocytosis;Role of second messengers in netrin-1 signaling;Signaling by FGFR1 in disease;Signaling by FGFR2 in disease;Signaling by FGFR3 fusions in cancer;Signaling by FGFR3 point mutants in cancer;Signaling by FGFR4 in disease;Signaling by SCF-KIT;Signaling by cytosolic FGFR1 fusion mutants;Synthesis of PIPs at the plasma membrane;VEGFA-VEGFR2 Pathway;VEGFR2 mediated cell proliferation;WNT mediated activation of DVL	PIP5K1A	

Table B (continued)

Pathway	Regs Conn	Regs NConn
Activated NTRK2 signals through PI3K;Antigen activates B Cell Receptor (BCR) leading to generation of second messengers;CD28 dependent PI3K/Akt signaling;Constitutive Signaling by Aberrant PI3K in Cancer;Constitutive Signaling by EGFRvIII;Constitutive Signaling by Ligand-Responsive EGFR Cancer Variants;DAP12 signaling;Downstream TCR signaling;Downstream signal transduction;G beta:gamma signalling through PI3Kgamma;GAB1 signalosome;GPVI-mediated activation cascade;MET activates PI3K/AKT signaling;PI-3K cascade:FGFR1;PI-3K cascade:FGFR2;PI-3K cascade:FGFR3;PI-3K cascade:FGFR4;PI3K Cascade;PI3K events in ERBB2 signaling;PI3K events in ERBB4 signaling;PI3K/AKT Signaling;PI3K/AKT activation;PI5P, PP2A and IER3 Regulate PI3K/AKT Signaling;Role of LAT2/NTAL/LAB on calcium mobilization;Role of phospholipids in phagocytosis;Signaling by FGFR1 in disease;Signaling by FGFR2 in disease;Signaling by FGFR3 fusions in cancer;Signaling by FGFR3 point mutants in cancer;Signaling by FGFR4 in disease;Signaling by SCF-KIT;Signaling by cytosolic FGFR1 fusion mutants;Synthesis of PIPs at the plasma membrane;VEGFA-VEGFR2 Pathway	PIK3R1	
Activated PKN1 stimulates transcription of AR (androgen receptor) regulated genes KLK2 and KLK3	HIST2H2BE	
Activated PKN1 stimulates transcription of AR (androgen receptor) regulated genes KLK2 and KLK3;Activation of anterior HOX genes in hindbrain development during early embryogenesis;Activation of rRNA Expression by ERCC6 (CSB) and EHMT2 (G9a);B-WICH complex positively regulates rRNA expression;Condensation of Prophase Chromosomes;DNA Damage/Telomere Stress Induced Senescence;DNA methylation;Deposition of new CENPA-containing nucleosomes at the centromere;Estrogen-dependent gene expression;Formation of the beta-catenin:TCF transactivating complex;G2/M DNA damage checkpoint;Meiotic recombination;Meiotic synapsis;NoRC negatively regulates rRNA expression;Nonhomologous End-Joining (NHEJ);Oxidative Stress Induced Senescence;PRC2 methylates histones and DNA;Packaging Of Telomere Ends;Processing of DNA double-strand break ends;RNA Polymerase I Chain Elongation;RNA Polymerase I Promoter Opening;RUNX1 regulates genes involved in megakaryocyte differentiation and platelet function;RUNX1 regulates transcription of genes involved in differentiation of HSCs;Recruitment and ATM-mediated phosphorylation of repair and signaling proteins at DNA double strand breaks;SIRT1 negatively regulates rRNA expression;Senescence-Associated Secretory Phenotype (SASP);Transcriptional regulation by small RNAs	HIST2H2BE	
Activation of anterior HOX genes in hindbrain development during early embryogenesis;Estrogen-dependent gene expression;RUNX1 regulates genes involved in megakaryocyte differentiation and platelet function	HIST2H2BE	
Activation of anterior HOX genes in hindbrain development during early embryogenesis;Nonhomologous End-Joining (NHEJ)	HIST2H2BE	

Table B (continued)

Pathway	Regs Conn	Regs NConn
Activation of anterior HOX genes in hindbrain development during early embryogenesis;Transcriptional regulation by small RNAs	HIST2H2BE	
Activation of ATR in response to replication stress	RNF139	
Activation of ATR in response to replication stress;Activation of the pre-replicative complex		S100A14
Activation of ATR in response to replication stress;Activation of the pre-replicative complex;Assembly of the ORC complex at the origin of replication;Assembly of the pre-replicative complex;CDC6 association with the ORC:origin complex;CDT1 association with the CDC6:ORC:origin complex;E2F-enabled inhibition of pre-replication complex formation;Orc1 removal from chromatin		S100A14
Activation of ATR in response to replication stress;Activation of the pre-replicative complex;Assembly of the pre-replicative complex;Orc1 removal from chromatin;Switching of origins to a post-replicative state;Unwinding of DNA		CXCL14
Activation of ATR in response to replication stress;Fanconi Anemia Pathway;HDR through Single Strand Annealing (SSA);Presynaptic phase of homologous DNA pairing and strand exchange;Processing of DNA double-strand break ends;Regulation of TP53 Activity through Phosphorylation	RNF139	
Activation of E2F1 target genes at G1/S;Cyclin E associated events during G1/S transition;G0 and Early G1;Transcription of E2F targets under negative control by DREAM complex	SEC62	
Activation of NF-kappaB in B cells	PSMB10	
Activation of NF-kappaB in B cells;CLEC7A (Dectin-1) signaling;Downstream TCR signaling;FCERI mediated NF-kB activation;Interleukin-1 signaling	PSMB10	
Activation of NIMA Kinases NEK9, NEK6, NEK7	CCNH	
Activation of RAC1		GRHL2
Activation of rRNA Expression by ERCC6 (CSB) and EHMT2 (G9a);B-WICH complex positively regulates rRNA expression;SIRT1 negatively regulates rRNA expression	HIST2H2BE	
Activation of rRNA Expression by ERCC6 (CSB) and EHMT2 (G9a);HDACs deacetylate histones;NoRC negatively regulates rRNA expression;SIRT1 negatively regulates rRNA expression	HIST2H2BE	
Activation of rRNA Expression by ERCC6 (CSB) and EHMT2 (G9a);HDACs deacetylate histones;Oxidative Stress Induced Senescence;PKMTs methylate histone lysines;PRC2 methylates histones and DNA;SIRT1 negatively regulates rRNA expression		LYL1
Activation of rRNA Expression by ERCC6 (CSB) and EHMT2 (G9a);Oxidative Stress Induced Senescence;SIRT1 negatively regulates rRNA expression		LYL1
Activation of rRNA Expression by ERCC6 (CSB) and EHMT2 (G9a);RNA Polymerase I Chain Elongation	HIST2H2BE	
Activation of rRNA Expression by ERCC6 (CSB) and EHMT2 (G9a);SIRT1 negatively regulates rRNA expression	HIST2H2BE	

Table B (continued)

Pathway	Regs Conn	Regs NConn
Activation of rRNA Expression by ERCC6 (CSB) and EHMT2 (G9a);SIRT1 negatively regulates rRNA expression;Senescence-Associated Secretory Phenotype (SASP)	HIST2H2BE	
Activation of the mRNA upon binding of the cap-binding complex and eIFs, and subsequent binding to 43S;Deadenylation of mRNA;GTP hydrolysis and joining of the 60S ribosomal subunit;ISG15 antiviral mechanism;L13a-mediated translational silencing of Ceruloplasmin expression;Ribosomal scanning and start codon recognition;Translation initiation complex formation	IDH1	
Activation of the phototransduction cascade;Ion homeostasis;Ion transport by P-type ATPases;Sodium/Calcium exchangers		HTN1
acyl-CoA hydrolysis	ALDH3A1	
acyl-CoA hydrolysis;stearate biosynthesis I (animals)	ALDH3A1	
adenosine nucleotides degradation II;purine nucleotides de novo biosynthesis II;purine nucleotides degradation II (aerobic)		KCNK7
Adrenaline,noradrenaline inhibits insulin secretion		ETV7
ALK1 signaling events;Angiopoietin receptor Tie2-mediated signaling;Arf6 downstream pathway;CDC42 signaling events;CXCR3-mediated signaling events;Cellular roles of Anthrax toxin;EPHB forward signaling;Endothelins;ErbB1 downstream signaling;ErbB4 signaling events;FGF signaling pathway;GMCSF-mediated signaling events;IFN-gamma pathway;Integrins in angiogenesis;Netrin-mediated signaling events;Neurotrophic factor-mediated Trk receptor signaling;Nongenotropic Androgen signaling;Osteopontin-mediated events;PDGFR-beta signaling pathway;Regulation of Telomerase;S1P1 pathway;S1P2 pathway;S1P3 pathway;S1P4 pathway;Signaling events mediated by PRL;Signaling events mediated by VEGFR1 and VEGFR2;Signaling events regulated by Ret tyrosine kinase;Syndecan-1-mediated signaling events;Syndecan-2-mediated signaling events;TRAIL signaling pathway;Trk receptor signaling mediated by the MAPK pathway;VEGFR1 specific signals;VEGFR3 signaling in lymphatic endothelium;mTOR signaling pathway	TNFSF10	
ALK1 signaling events;Glypican 1 network;TGF-beta receptor signaling		PEBP4
Alpha9 beta1 integrin signaling events;Angiopoietin receptor Tie2-mediated signaling;Cellular roles of Anthrax toxin;Glypican 1 network;Plasma membrane estrogen receptor signaling;SHP2 signaling;Signaling events mediated by VEGFR1 and VEGFR2;Thromboxane A2 receptor signaling;VEGFR1 specific signals		HOXD9
Alpha-synuclein signaling		ADGRB1
Amino acid transport across the plasma membrane;Carboxyterminal post-translational modifications of tubulin	IQGAP2	
Amino acid transport across the plasma membrane;Cytosolic tRNA aminoacylation	IQGAP2	
Amino acid transport across the plasma membrane;mTOR signalling	IQGAP2	

Table B (continued)

Pathway	Regs Conn	Regs NConn
Amplification of signal from unattached kinetochores via a MAD2 inhibitory signal;Anchoring of the basal body to the plasma membrane;Mitotic Prometaphase;RHO GTPases Activate Formins;Resolution of Sister Chromatid Cohesion;Separation of Sister Chromatids	LSM11	
Amplification of signal from unattached kinetochores via a MAD2 inhibitory signal;Mitotic Prometaphase;Nonsense Mediated Decay (NMD) enhanced by the Exon Junction Complex (EJC);RHO GTPases Activate Formins;Resolution of Sister Chromatid Cohesion;Separation of Sister Chromatids	RPL13A	
Angiogenesis;EGF receptor signaling pathway	PLCG2	
Angiogenesis;Inflammation mediated by chemokine and cytokine signaling pathway;Interleukin signaling pathway;PDGF signaling pathway;Ras Pathway;VEGF signaling pathway	PIK3R1	
Angiogenesis;Ras Pathway	PIK3R1	
Angiotensin_II-stimulated_signaling_through_G_proteins_and_beta-arrestin		ETV7
Angiotensin_II-stimulated_signaling_through_G_proteins_and_beta-arrestin;B cell activation;Metabotropic glutamate receptor group I pathway;Muscarinic acetylcholine receptor 1 and 3 signaling pathway;T cell activation;Thyrotropin-releasing hormone receptor signaling pathway	PLCG2	
Angiotensin_II-stimulated_signaling_through_G_proteins_and_beta-arrestin;Endogenous_cannabinoid_signaling;GABA-B_receptor_II_signaling;Heterotrimeric G-protein signaling pathway-Gi alpha and Gs alpha mediated pathway;Heterotrimeric G-protein signaling pathway-Gq alpha and Go alpha mediated pathway;Heterotrimeric G-protein signaling pathway-rod outer segment phototransduction;PNAT;Wnt signaling pathway		ETV7
Angiotensin_II-stimulated_signaling_through_G_proteins_and_beta-arrestin;Endogenous_cannabinoid_signaling;GABA-B_receptor_II_signaling;Heterotrimeric G-protein signaling pathway-Gq alpha and Go alpha mediated pathway;Heterotrimeric G-protein signaling pathway-rod outer segment phototransduction;PNAT;Wnt signaling pathway		ETV7
Angiotensin_II-stimulated_signaling_through_G_proteins_and_beta-arrestin;Endogenous_cannabinoid_signaling;GABA-B_receptor_II_signaling;PNAT;Wnt signaling pathway		ETV7
Antigen activates B Cell Receptor (BCR) leading to generation of second messengers;Ca2+ pathway;Effects of PIP2 hydrolysis;G beta:gamma signalling through PLC beta;GPVI-mediated activation cascade	PLCG2	
Antigen activates B Cell Receptor (BCR) leading to generation of second messengers;Ca2+ pathway;G beta:gamma signalling through PLC beta;GPVI-mediated activation cascade;Synthesis of IP2, IP, and Ins in the cytosol	GALE	

Table B (continued)

Pathway	Regs Conn	Regs NConn
Antigen activates B Cell Receptor (BCR) leading to generation of second messengers;CLEC7A (Dectin-1) signaling;Ca2+ pathway;Clathrin-mediated endocytosis;DAG and IP3 signaling;FCERI mediated Ca+2 mobilization;G beta:gamma signalling through PLC beta;GPVI-mediated activation cascade;Generation of second messenger molecules;PI5P, PP2A and IER3 Regulate PI3K/AKT Signaling;PLC beta mediated events;Role of phospholipids in phagocytosis;Role of second messengers in netrin-1 signaling;Synthesis of PIPs at the plasma membrane;VEGFR2 mediated cell proliferation;WNT mediated activation of DVL	PIP5K1A	
Antigen activates B Cell Receptor (BCR) leading to generation of second messengers;Constitutive Signaling by Aberrant PI3K in Cancer;PI5P, PP2A and IER3 Regulate PI3K/AKT Signaling	PIK3R1	
Antigen activates B Cell Receptor (BCR) leading to generation of second messengers;DAP12 signaling;FCERI mediated Ca+2 mobilization	PLCG2	
Antigen activates B Cell Receptor (BCR) leading to generation of second messengers;DAP12 signaling;FCERI mediated Ca+2 mobilization;FCERI mediated MAPK activation	PLCG2	
Antigen activates B Cell Receptor (BCR) leading to generation of second messengers;FCERI mediated Ca+2 mobilization;FCERI mediated MAPK activation	PLCG2	
Antigen activates B Cell Receptor (BCR) leading to generation of second messengers;Role of phospholipids in phagocytosis	PLCG2	
Antigen Presentation: Folding, assembly and peptide loading of class I MHC;Antigen processing: Ubiquitination and Proteasome degradation;ER-Phagosome pathway	PSMB10	
Antigen processing: Ubiquitination and Proteasome degradation;Cross-presentation of soluble exogenous antigens (endosomes)	PSMB10	
Antigen processing: Ubiquitination and Proteasome degradation;Cross-presentation of soluble exogenous antigens (endosomes);ER-Phagosome pathway;Methionine salvage pathway	PSMB10	
Antigen processing: Ubiquitination and Proteasome degradation;ER-Phagosome pathway	PSMB10	
Antigen processing: Ubiquitination and Proteasome degradation;FBXL7 down-regulates AURKA during mitotic entry and in early mitosis;Neddylation		PMP22
Antigen processing: Ubiquitination and Proteasome degradation;Interconversion of nucleotide di- and triphosphates	ATG7	
Antigen processing: Ubiquitination and Proteasome degradation;Neddylation	BCAN	
Antigen processing: Ubiquitination and Proteasome degradation;Neddylation;Oxygen-dependent proline hydroxylation of Hypoxia-inducible Factor Alpha	VHL	
Antigen processing: Ubiquitination and Proteasome degradation;Purine catabolism	ATG7	
Antigen processing: Ubiquitination and Proteasome degradation;Purine salvage	ATG7	

Table B (continued)

Pathway	Regs Conn	Regs NConn
APC/C:Cdc20 mediated degradation of Securin;Cdc20:Phospho-APC/C mediated degradation of Cyclin A;SCF-beta-TrCP mediated degradation of Emi1;Separation of Sister Chromatids	PSMB10	
APC/C:Cdh1 mediated degradation of Cdc20 and other APC/C:Cdh1 targeted proteins in late mitosis/early G1;SCF-beta-TrCP mediated degradation of Emi1	PSMB10	
APC-Cdc20 mediated degradation of Nek2A;APC/C:Cdc20 mediated degradation of Cyclin B;APC/C:Cdc20 mediated degradation of Securin;APC/C:Cdc20 mediated degradation of mitotic proteins;APC/C:Cdh1 mediated degradation of Cdc20 and other APC/C:Cdh1 targeted proteins in late mitosis/early G1;Autodegradation of Cdh1 by Cdh1:APC/C;CDK-mediated phosphorylation and removal of Cdc6;Cdc20:Phospho-APC/C mediated degradation of Cyclin A;Conversion from APC/C:Cdc20 to APC/C:Cdh1 in late anaphase;Inactivation of APC/C via direct inhibition of the APC/C complex;Phosphorylation of the APC/C;Regulation of APC/C activators between G1/S and early anaphase;Senescence-Associated Secretory Phenotype (SASP);Separation of Sister Chromatids		CDX1
APEX1-Independent Resolution of AP Sites via the Single Nucleotide Replacement Pathway;Processing of DNA double-strand break ends		SLC39A13
Arf1 pathway;BCR signaling pathway;CDC42 signaling events;CXCR4-mediated signaling events;Class I PI3K signaling events;E-cadherin signaling in keratinocytes;EPHB forward signaling;EPO signaling pathway;Endothelins;ErbB1 downstream signaling;FGF signaling pathway;Fc-epsilon receptor I signaling in mast cells;IL2 signaling events mediated by PI3K;IL8- and CXCR1-mediated signaling events;IL8- and CXCR2-mediated signaling events;LPA receptor mediated events;LPA4-mediated signaling events;Nephrin/Neph1 signaling in the kidney podocyte;Netrin-mediated signaling events;Nongenotropic Androgen signaling;PAR1-mediated thrombin signaling events;PDGFR-alpha signaling pathway;PDGFR-beta signaling pathway;Plasma membrane estrogen receptor signaling;Posttranslational regulation of adherens junction stability and dissassembly;Regulation of Ras family activation;Role of Calcineurin-dependent NFAT signaling in lymphocytes;Signaling events mediated by Hepatocyte Growth Factor Receptor (c-Met);Signaling events mediated by VEGFR1 and VEGFR2;TCR signaling in nave CD4+ T cells;TCR signaling in nave CD8+ T cells;Thromboxane A2 receptor signaling;Trk receptor signaling mediated by PI3K and PLC-gamma;VEGFR1 specific signals	PLCG2	

Table B (continued)

Pathway	Regs Conn	Regs NConn
Arf1 pathway;BCR signaling pathway;CXCR4-mediated signaling events;Class I PI3K signaling events;E-cadherin signaling in keratinocytes;EPO signaling pathway;Endothelins;ErbB1 downstream signaling;FGF signaling pathway;Fc-epsilon receptor I signaling in mast cells;IL2 signaling events mediated by PI3K;IL2-mediated signaling events;IL8- and CXCR1-mediated signaling events;IL8- and CXCR2-mediated signaling events;LPA receptor mediated events;LPA4-mediated signaling events;Nephrin/Neph1 signaling in the kidney podocyte;Netrin-mediated signaling events;Nongenotropic Androgen signaling;PAR1-mediated thrombin signaling events;PDGFR-alpha signaling pathway;PDGFR-beta signaling pathway;Plasma membrane estrogen receptor signaling;Regulation of Ras family activation;Role of Calcineurin-dependent NFAT signaling in lymphocytes;Signaling events mediated by Hepatocyte Growth Factor Receptor (c-Met);Signaling events mediated by VEGFR1 and VEGFR2;TCR signaling in nave CD4+ T cells;TCR signaling in nave CD8+ T cells;Thromboxane A2 receptor signaling;Trk receptor signaling mediated by PI3K and PLC-gamma;VEGFR1 specific signals	PLCG2	
Arf1 pathway;BCR signaling pathway;CXCR4-mediated signaling events;Class I PI3K signaling events;E-cadherin signaling in keratinocytes;EPO signaling pathway;Endothelins;ErbB1 downstream signaling;FGF signaling pathway;Fc-epsilon receptor I signaling in mast cells;IL2 signaling events mediated by PI3K;IL8- and CXCR1-mediated signaling events;IL8- and CXCR2-mediated signaling events;LPA receptor mediated events;LPA4-mediated signaling events;Nephrin/Neph1 signaling in the kidney podocyte;Netrin-mediated signaling events;Nongenotropic Androgen signaling;PAR1-mediated thrombin signaling events;PDGFR-alpha signaling pathway;PDGFR-beta signaling pathway;Plasma membrane estrogen receptor signaling;Plexin-D1 Signaling;Regulation of Ras family activation;Role of Calcineurin-dependent NFAT signaling in lymphocytes;Signaling events mediated by Hepatocyte Growth Factor Receptor (c-Met);Signaling events mediated by VEGFR1 and VEGFR2;TCR signaling in nave CD4+ T cells;TCR signaling in nave CD8+ T cells;Thromboxane A2 receptor signaling;Trk receptor signaling mediated by PI3K and PLC-gamma;VEGFR1 specific signals	PLCG2	

Table B (continued)

Pathway	Regs Conn	Regs NConn
Arf1 pathway;BCR signaling pathway;CXCR4-mediated signaling events;Class I PI3K signaling events;Endothelins;FGF signaling pathway;IL8- and CXCR1-mediated signaling events;IL8- and CXCR2-mediated signaling events;LPA receptor mediated events;Nephrin/Neph1 signaling in the kidney podocyte;Netrin-mediated signaling events;Nongenotropic Androgen signaling;Osteopontin-mediated events;PDGFR-alpha signaling pathway;PDGFR-beta signaling pathway;Plasma membrane estrogen receptor signaling;RhoA signaling pathway;Signaling events mediated by Hepatocyte Growth Factor Receptor (c-Met);Signaling events mediated by VEGFR1 and VEGFR2;TCR signaling in nave CD4+ T cells;TCR signaling in nave CD8+ T cells;Thromboxane A2 receptor signaling;Trk receptor signaling mediated by PI3K and PLC-gamma;VEGFR1 specific signals	PLCG2	
Arf6 signaling events;BCR signaling pathway;CDC42 signaling events;CXCR3-mediated signaling events;CXCR4-mediated signaling events;Class I PI3K signaling events;Class I PI3K signaling events mediated by Akt;E-cadherin signaling in the nascent adherens junction;EPHA2 forward signaling;ErbB1 downstream signaling;ErbB2/ErbB3 signaling events;FAS (CD95) signaling pathway;FGF signaling pathway;GMCSF-mediated signaling events;IFN-gamma pathway;IL2 signaling events mediated by PI3K;IL4-mediated signaling events;IL8- and CXCR1-mediated signaling events;IL8- and CXCR2-mediated signaling events;Insulin Pathway;Nephrin/Neph1 signaling in the kidney podocyte;Osteopontin-mediated events;PDGFR-beta signaling pathway;Plasma membrane estrogen receptor signaling;RAC1 signaling pathway;Regulation of Ras family activation;Signaling events mediated by Hepatocyte Growth Factor Receptor (c-Met);Signaling events mediated by VEGFR1 and VEGFR2;TCR signaling in nave CD4+ T cells;TCR signaling in nave CD8+ T cells;Trk receptor signaling mediated by PI3K and PLC-gamma;VEGFR1 specific signals	PIK3R1	

Table B (continued)

Pathway	Regs Conn	Regs NConn
Arf6 signaling events;BCR signaling pathway;CXCR3-mediated signaling events;CXCR4-mediated signaling events;Class I PI3K signaling events;Class I PI3K signaling events mediated by Akt;E-cadherin signaling in the nascent adherens junction;EPHA2 forward signaling;ErbB1 downstream signaling;ErbB2/ErbB3 signaling events;FAS (CD95) signaling pathway;FGF signaling pathway;GMCSF-mediated signaling events;IFN-gamma pathway;IGF1 pathway;IL2 signaling events mediated by PI3K;IL4-mediated signaling events;IL8- and CXCR1-mediated signaling events;IL8- and CXCR2-mediated signaling events;Insulin Pathway;LPA receptor mediated events;Nephrin/Neph1 signaling in the kidney podocyte;Osteopontin-mediated events;PDGFR-beta signaling pathway;Plasma membrane estrogen receptor signaling;RAC1 signaling pathway;Regulation of Ras family activation;Signaling events mediated by Hepatocyte Growth Factor Receptor (c-Met);Signaling events mediated by Stem cell factor receptor (c-Kit);Signaling events mediated by VEGFR1 and VEGFR2;TCR signaling in nave CD4+ T cells;TCR signaling in nave CD8+ T cells;Trk receptor signaling mediated by PI3K and PLC-gamma;VEGFR1 specific signals	PIK3R1	
Arf6 signaling events;BCR signaling pathway;CXCR3-mediated signaling events;CXCR4-mediated signaling events;Class I PI3K signaling events;Class I PI3K signaling events mediated by Akt;E-cadherin signaling in the nascent adherens junction;EPHA2 forward signaling;ErbB1 downstream signaling;ErbB2/ErbB3 signaling events;FAS (CD95) signaling pathway;FGF signaling pathway;GMCSF-mediated signaling events;IFN-gamma pathway;IL2 signaling events mediated by PI3K;IL4-mediated signaling events;IL8- and CXCR1-mediated signaling events;IL8- and CXCR2-mediated signaling events;Insulin Pathway;Nephrin/Neph1 signaling in the kidney podocyte;Osteopontin-mediated events;PDGFR-beta signaling pathway;Plasma membrane estrogen receptor signaling;Plexin-D1 Signaling;RAC1 signaling pathway;Regulation of Ras family activation;Signaling events mediated by Hepatocyte Growth Factor Receptor (c-Met);Signaling events mediated by VEGFR1 and VEGFR2;Stabilization and expansion of the E-cadherin adherens junction;TCR signaling in nave CD4+ T cells;TCR signaling in nave CD8+ T cells;Trk receptor signaling mediated by PI3K and PLC-gamma;VEGFR1 specific signals	PIK3R1	

Table B (continued)

Pathway	Regs Conn	Regs NConn
Arf6 trafficking events;BCR signaling pathway;CXCR3-mediated signaling events;CXCR4-mediated signaling events;Canonical Wnt signaling pathway;Class I PI3K signaling events;E-cadherin signaling in the nascent adherens junction;EPHA2 forward signaling;ErbB1 downstream signaling;ErbB2/ErbB3 signaling events;FAS (CD95) signaling pathway;IFN-gamma pathway;IL2 signaling events mediated by PI3K;IL4-mediated signaling events;IL8- and CXCR1-mediated signaling events;IL8- and CXCR2-mediated signaling events;Insulin Pathway;LPA receptor mediated events;Nephrin/Neph1 signaling in the kidney podocyte;PDGFR-beta signaling pathway;Plasma membrane estrogen receptor signaling;Plexin-D1 Signaling;RhoA signaling pathway;Signaling events mediated by Hepatocyte Growth Factor Receptor (c-Met);Signaling events mediated by Stem cell factor receptor (c-Kit);Signaling events mediated by VEGFR1 and VEGFR2;TCR signaling in nave CD4+ T cells;TCR signaling in nave CD8+ T cells;Trk receptor signaling mediated by PI3K and PLC-gamma;VEGFR1 specific signals	PIK3R1	
Arf6 trafficking events;CXCR4-mediated signaling events;Canonical Wnt signaling pathway;Class I PI3K signaling events;IL8- and CXCR1-mediated signaling events;IL8- and CXCR2-mediated signaling events;Nephrin/Neph1 signaling in the kidney podocyte;Netrin-mediated signaling events;Nongenotropic Androgen signaling;PDGFR-beta signaling pathway;Plasma membrane estrogen receptor signaling;Plexin-D1 Signaling;RhoA signaling pathway;Signaling events mediated by VEGFR1 and VEGFR2;TCR signaling in nave CD4+ T cells;TCR signaling in nave CD8+ T cells;Trk receptor signaling mediated by PI3K and PLC-gamma;VEGFR1 specific signals	PLCG2	
arginine biosynthesis IV;citrulline degradation;proline biosynthesis II (from arginine);urea cycle	PCK1	
Association of TriC/CCT with target proteins during biosynthesis	BCAN	
Asymmetric localization of PCP proteins	PSMB10	
ATF4 activates genes;Butyrate Response Factor 1 (BRF1) binds and destabilizes mRNA;KSRP (KHSRP) binds and destabilizes mRNA;Major pathway of rRNA processing in the nucleolus and cytosol;Tristetraprolin (TTP, ZFP36) binds and destabilizes mRNA;mRNA decay by 3' to 5' exoribonuclease		FAM212B
ATM pathway	XRCC4	
ATM pathway;BARD1 signaling events;Fanconi anemia pathway;Regulation of Telomerase		PAM
ATR signaling pathway;Fanconi anemia pathway		RNF139
Attenuation phase	PARP10	
AUF1 (hnRNP D0) binds and destabilizes mRNA	PSMB10	
Aurora A signaling	RASA1	
Aurora A signaling;Aurora B signaling	RASA1	
Axon guidance mediated by netrin	PLCG2	
BARD1 signaling events;Fanconi anemia pathway	UBE2T	

Table B (continued)

Pathway	Regs Conn	Regs NConn
BCR signaling pathway;CXCR3-mediated signaling events;CXCR4-mediated signaling events;Class I PI3K signaling events;E-cadherin signaling in the nascent adherens junction;EPHA2 forward signaling;ErbB1 downstream signaling;ErbB2/ErbB3 signaling events;FAS (CD95) signaling pathway;FGF signaling pathway;GMCSF-mediated signaling events;IFN-gamma pathway;IL2 signaling events mediated by PI3K;IL4-mediated signaling events;IL8- and CXCR1-mediated signaling events;IL8- and CXCR2-mediated signaling events;Insulin Pathway;Nephrin/Neph1 signaling in the kidney podocyte;Osteopontin-mediated events;PDGFR-beta signaling pathway;Plasma membrane estrogen receptor signaling;Signaling events mediated by Hepatocyte Growth Factor Receptor (c-Met);Signaling events mediated by VEGFR1 and VEGFR2;TCR signaling in nave CD4+ T cells;TCR signaling in nave CD8+ T cells;Trk receptor signaling mediated by PI3K and PLC-gamma;VEGFR1 specific signals	PIK3R1	
BCR signaling pathway;CXCR3-mediated signaling events;CXCR4-mediated signaling events;Class I PI3K signaling events;E-cadherin signaling in the nascent adherens junction;EPHA2 forward signaling;ErbB1 downstream signaling;ErbB2/ErbB3 signaling events;FAS (CD95) signaling pathway;GMCSF-mediated signaling events;IFN-gamma pathway;IL2 signaling events mediated by PI3K;IL4-mediated signaling events;IL8- and CXCR1-mediated signaling events;IL8- and CXCR2-mediated signaling events;Insulin Pathway;Nephrin/Neph1 signaling in the kidney podocyte;PDGFR-beta signaling pathway;Plasma membrane estrogen receptor signaling;Signaling events mediated by Hepatocyte Growth Factor Receptor (c-Met);Signaling events mediated by VEGFR1 and VEGFR2;TCR signaling in nave CD4+ T cells;TCR signaling in nave CD8+ T cells;Trk receptor signaling mediated by PI3K and PLC-gamma;VEGFR1 specific signals		MCMDC2
BCR signaling pathway;CXCR3-mediated signaling events;CXCR4-mediated signaling events;Class I PI3K signaling events;E-cadherin signaling in the nascent adherens junction;EPHA2 forward signaling;ErbB1 downstream signaling;ErbB2/ErbB3 signaling events;FAS (CD95) signaling pathway;IFN-gamma pathway;IL2 signaling events mediated by PI3K;IL4-mediated signaling events;IL8- and CXCR1-mediated signaling events;IL8- and CXCR2-mediated signaling events;Insulin Pathway;LPA receptor mediated events;Nephrin/Neph1 signaling in the kidney podocyte;Netrin-mediated signaling events;PDGFR-beta signaling pathway;Plasma membrane estrogen receptor signaling;Signaling events mediated by Hepatocyte Growth Factor Receptor (c-Met);Signaling events mediated by Stem cell factor receptor (c-Kit);Signaling events mediated by VEGFR1 and VEGFR2;TCR signaling in nave CD4+ T cells;TCR signaling in nave CD8+ T cells;Trk receptor signaling mediated by PI3K and PLC-gamma;VEGFR1 specific signals	PIK3R1	
BCR signaling pathway;EPO signaling pathway	ARHGEF4	
Beta1 integrin cell surface interactions		ZNF488
Beta3 adrenergic receptor signaling pathway		MNS1

Table B (continued)

Pathway	Regs Conn	Regs NConn
Beta3 adrenergic receptor signaling pathway;Heterotrimeric G-protein signaling pathway-Gi alpha and Gs alpha mediated pathway		MNS1
Beta-catenin phosphorylation cascade		NDFIP1
Bicarbonate transporters;Erythrocytes take up carbon dioxide and release oxygen;Erythrocytes take up oxygen and release carbon dioxide;Multifunctional anion exchangers;Reversible hydration of carbon dioxide		FBLN1
Binding of TCF/LEF:CTNNB1 to target gene promoters;Constitutive Signaling by NOTCH1 HD+PEST Domain Mutants;Constitutive Signaling by NOTCH1 PEST Domain Mutants;Estrogen-dependent gene expression;NOTCH1 Intracellular Domain Regulates Transcription;RUNX3 regulates WNT signaling;SMAD2/SMAD3:SMAD4 heterotrimer regulates transcription	HIST2H2BE	
Budding and maturation of HIV virion		CDX1
Budding and maturation of HIV virion;Membrane binding and targetting of GAG proteins		RASSF5
B-WICH complex positively regulates rRNA expression;HATs acetylate histones	HIST2H2BE	
B-WICH complex positively regulates rRNA expression;HATs acetylate histones;RUNX1 regulates genes involved in megakaryocyte differentiation and platelet function		ANKRD46
B-WICH complex positively regulates rRNA expression;Metalloprotease DUBs		ANKRD46
B-WICH complex positively regulates rRNA expression;NoRC negatively regulates rRNA expression;Recruitment and ATM-mediated phosphorylation of repair and signaling proteins at DNA double strand breaks	HIST2H2BE	
B-WICH complex positively regulates rRNA expression;Recruitment and ATM-mediated phosphorylation of repair and signaling proteins at DNA double strand breaks	HIST2H2BE	
B-WICH complex positively regulates rRNA expression;Ub-specific processing proteases	HIST2H2BE	
calcium transport I	CACNB3	
Carboxyterminal post-translational modifications of tubulin;Glutathione synthesis and recycling		APOA1BP
Carboxyterminal post-translational modifications of tubulin;Post-chaperonin tubulin folding pathway		NUP210L
Cargo recognition for clathrin-mediated endocytosis;Clathrin-mediated endocytosis	RASGRF2	
Cargo recognition for clathrin-mediated endocytosis;Clathrin-mediated endocytosis;EPH-ephrin mediated repulsion of cells	RASGRF2	
Cargo recognition for clathrin-mediated endocytosis;Clathrin-mediated endocytosis;EPH-ephrin mediated repulsion of cells;Formation of annular gap junctions;Gap junction degradation	RASGRF2	
Cargo recognition for clathrin-mediated endocytosis;Clathrin-mediated endocytosis;Nef Mediated CD4 Down-regulation		ATXN7L2
Cargo recognition for clathrin-mediated endocytosis;Clathrin-mediated endocytosis;WNT5A-dependent internalization of FZD4		MYO1D

Table B (continued)

Pathway	Regs Conn	Regs NConn
Cation-coupled Chloride cotransporters;Ion homeostasis;Ion transport by P-type ATPases		LINC00051
Cation-coupled Chloride cotransporters;Mitochondrial calcium ion transport		HTN1
Cation-coupled Chloride cotransporters;Neurotransmitter receptors and postsynaptic signal transmission		LINC00051
Cation-coupled Chloride cotransporters;Phase 0 - rapid depolarisation;Stimuli-sensing channels;TRP channels		HTN1
CD28 dependent PI3K/Akt signaling;Constitutive Signaling by Aberrant PI3K in Cancer;PI5P, PP2A and IER3 Regulate PI3K/AKT Signaling	PIK3R1	
CD28 dependent Vav1 pathway		ABCC12
CD28 dependent Vav1 pathway;DAP12 signaling;EPH-ephrin mediated repulsion of cells;EPHB-mediated forward signaling;FCERI mediated MAPK activation;SEMA3A-Plexin repulsion signaling by inhibiting Integrin adhesion;Signal transduction by L1;Signaling by SCF-KIT	PLCG2	
CD28 dependent Vav1 pathway;DAP12 signaling;EPH-ephrin mediated repulsion of cells;EPHB-mediated forward signaling;SEMA3A-Plexin repulsion signaling by inhibiting Integrin adhesion;Signal transduction by L1;Signaling by SCF-KIT	PLCG2	
Cdc20:Phospho-APC/C mediated degradation of Cyclin A	PSMB10	
CDC42 signaling events;EPHB forward signaling;Fc-epsilon receptor I signaling in mast cells;Nongenotropic Androgen signaling;Posttranslational regulation of adherens junction stability and dissassembly;Regulation of Ras family activation	RASGRF2	
CDP-diacylglycerol biosynthesis I;phosphatidylglycerol biosynthesis II (non-plastidic);triacylglycerol biosynthesis	ALDH3A1	
CDT1 association with the CDC6:ORC:origin complex		VPS8
Cellular hexose transport;Ion homeostasis;Ion transport by P-type ATPases		PART1
Cellular hexose transport;Phase 0 - rapid depolarisation;Stimuli-sensing channels;TRP channels		PART1
ceramide biosynthesis	ALDH3A1	
Class I PI3K signaling events	PLCG2	
Class I PI3K signaling events;Neurotrophic factor-mediated Trk receptor signaling;Trk receptor signaling mediated by PI3K and PLC-gamma	PIK3R1	
Clathrin-mediated endocytosis;Downstream TCR signaling;Invadopodia formation;PIPs transport between plasma and early endosome membranes;Regulation of actin dynamics for phagocytic cup formation;Regulation of signaling by CBL;Role of LAT2/NTAL/LAB on calcium mobilization;Synthesis of PIPs at the plasma membrane	PIK3R1	
Clathrin-mediated endocytosis;PI5P, PP2A and IER3 Regulate PI3K/AKT Signaling;Synthesis of PIPs at the plasma membrane;WNT mediated activation of DVL	PIP5K1A	
Clathrin-mediated endocytosis;Synthesis of PIPs at the plasma membrane	PIK3R1	

Table B (continued)

Pathway	Regs Conn	Regs NConn
Cleavage of Growing Transcript in the Termination Region;Nonsense Mediated Decay (NMD) enhanced by the Exon Junction Complex (EJC);Regulation of expression of SLITs and ROBOs;Transport of Mature mRNA derived from an Intron-Containing Transcript;mRNA 3'-end processing;pre-mRNA splicing	PROCR	
Cleavage of Growing Transcript in the Termination Region;Processing of Intronless Pre-mRNAs;mRNA 3'-end processing;pre-mRNA splicing		ZNF446
Cleavage of Growing Transcript in the Termination Region;Processing of Intronless Pre-mRNAs;mRNA 3'-end processing;pre-mRNA splicing;tRNA processing in the nucleus		ZNF446
Cleavage of Growing Transcript in the Termination Region;Processing of Intronless Pre-mRNAs;Transport of Mature mRNA Derived from an Intronless Transcript;mRNA 3'-end processing;pre-mRNA splicing		ICAM3
CLEC7A (Dectin-1) signaling;DAG and IP3 signaling;Downstream TCR signaling;FCERI mediated Ca+2 mobilization;FCERI mediated NF-kB activation;Generation of second messenger molecules;PLC beta mediated events;Role of phospholipids in phagocytosis;Role of second messengers in netrin-1 signaling;Sphingolipid de novo biosynthesis;VEGFR2 mediated cell proliferation	PLCG2	
CMP-N-acetylneuraminate biosynthesis I (eukaryotes)	ST3GAL2	
Cohesin Loading onto Chromatin;Establishment of Sister Chromatid Cohesion;Resolution of Sister Chromatid Cohesion;Separation of Sister Chromatids	NIPAL4	
Condensation of Prometaphase Chromosomes	ATG10	
Condensation of Prometaphase Chromosomes;Condensation of Prophase Chromosomes		WDR41
Constitutive Signaling by Aberrant PI3K in Cancer;Costimulation by the CD28 family;PI5P, PP2A and IER3 Regulate PI3K/AKT Signaling	PIK3R1	
Constitutive Signaling by Aberrant PI3K in Cancer;Downstream TCR signaling;PI5P, PP2A and IER3 Regulate PI3K/AKT Signaling	PIK3R1	
Constitutive Signaling by Aberrant PI3K in Cancer;PI3K events in ERBB2 signaling;PI3K events in ERBB4 signaling;PI5P, PP2A and IER3 Regulate PI3K/AKT Signaling	PIK3R1	
Constitutive Signaling by Aberrant PI3K in Cancer;PI5P, PP2A and IER3 Regulate PI3K/AKT Signaling	PIK3R1	
Constitutive Signaling by Aberrant PI3K in Cancer;PI5P, PP2A and IER3 Regulate PI3K/AKT Signaling;Signaling by SCF-KIT	PIK3R1	
Constitutive Signaling by EGFRvIII;Constitutive Signaling by Ligand-Responsive EGFR Cancer Variants	PIK3R1	
Constitutive Signaling by NOTCH1 HD Domain Mutants		ZBTB43
COPI-dependent Golgi-to-ER retrograde traffic;COPI-mediated anterograde transport		ADGRB1
COPI-independent Golgi-to-ER retrograde traffic	DCTN4	
COPI-mediated anterograde transport;COPII-mediated vesicle transport		MEF2C
COPI-mediated anterograde transport;Intra-Golgi traffic		MEF2C
Costimulation by the CD28 family	PIK3R1	
Cristae formation		SLC39A13

Table B (continued)

Pathway	Regs Conn	Regs NConn
Cristae formation;Formation of ATP by chemiosmotic coupling	ATP5G3	
CTLA4 inhibitory signaling		KLHL24
CXCR3-mediated signaling events;CXCR4-mediated signaling events;ErbB2/ErbB3 signaling events;ErbB4 signaling events;LPA receptor mediated events;Nephrin/Neph1 signaling in the kidney podocyte;PDGFR-beta signaling pathway	PIK3R1	
CXCR3-mediated signaling events;ErbB2/ErbB3 signaling events;ErbB4 signaling events;Nephrin/Neph1 signaling in the kidney podocyte	PIK3R1	
CXCR4-mediated signaling events;Class I PI3K signaling events;IL8- and CXCR1-mediated signaling events;IL8- and CXCR2-mediated signaling events;Nephrin/Neph1 signaling in the kidney podocyte;Netrin-mediated signaling events;Nongenotropic Androgen signaling;PDGFR-beta signaling pathway;Plasma membrane estrogen receptor signaling;Signaling events mediated by VEGFR1 and VEGFR2;TCR signaling in nave CD4+ T cells;TCR signaling in nave CD8+ T cells;Trk receptor signaling mediated by PI3K and PLC-gamma;VEGFR1 specific signals	PLCG2	
CXCR4-mediated signaling events;Downstream signaling in nave CD8+ T cells;IL12 signaling mediated by STAT4;IL12-mediated signaling events;TCR signaling in nave CD4+ T cells;TCR signaling in nave CD8+ T cells	ARHGEF4	
CXCR4-mediated signaling events;Nongenotropic Androgen signaling	PIK3R1	
CXCR4-mediated signaling events;PDGFR-beta signaling pathway	PIK3R1	
Cyclin A/B1/B2 associated events during G2/M transition;Cyclin A:Cdk2-associated events at S phase entry	CCNH	

Table B (continued)

Pathway	Regs Conn	Regs NConn
Cyclin A/B1/B2 associated events during G2/M transition;Cyclin A:Cdk2-associated events at S phase entry;Cyclin D associated events in G1;Cyclin E associated events during G1/S transition;Dual incision in TC-NER;Formation of HIV elongation complex in the absence of HIV Tat;Formation of HIV-1 elongation complex containing HIV-1 Tat;Formation of Incision Complex in GG-NER;Formation of RNA Pol II elongation complex;Formation of TC-NER Pre-Incision Complex;Formation of the Early Elongation Complex;Formation of the HIV-1 Early Elongation Complex;Gap-filling DNA repair synthesis and ligation in TC-NER;HIV Transcription Initiation;NoRC negatively regulates rRNA expression;RNA Pol II CTD phosphorylation and interaction with CE;RNA Pol II CTD phosphorylation and interaction with CE during HIV infection;RNA Polymerase I Chain Elongation;RNA Polymerase I Promoter Escape;RNA Polymerase I Transcription Initiation;RNA Polymerase I Transcription Termination;RNA Polymerase II HIV Promoter Escape;RNA Polymerase II Pre-transcription Events;RNA Polymerase II Promoter Escape;RNA Polymerase II Transcription Elongation;RNA Polymerase II Transcription Initiation;RNA Polymerase II Transcription Initiation And Promoter Clearance;RNA Polymerase II Transcription Pre-Initiation And Promoter Opening;RUNX1 regulates transcription of genes involved in differentiation of HSCs;TP53 Regulates Transcription of DNA Repair Genes;Tat-mediated elongation of the HIV-1 transcript;Transcription of the HIV genome;Transcription-Coupled Nucleotide Excision Repair (TC-NER);mRNA Capping		DAPK3
Cyclin A/B1/B2 associated events during G2/M transition;Cyclin D associated events in G1;Initiation of Nuclear Envelope Reformation;Nonsense Mediated Decay (NMD) enhanced by the Exon Junction Complex (EJC)	RPL13A	
Cyclin A:Cdk2-associated events at S phase entry;Cyclin E associated events during G1/S transition	CCNH	
Cyclin A:Cdk2-associated events at S phase entry;Cyclin E associated events during G1/S transition;Polo-like kinase mediated events;Transcription of E2F targets under negative control by DREAM complex	MYC	
Cyclin D associated events in G1	CCNH	
Cytosolic tRNA aminoacylation;Glutathione synthesis and recycling		APOA1BP
DAP12 signaling;FCERI mediated Ca+2 mobilization	PLCG2	
DAP12 signaling;FCERI mediated Ca+2 mobilization;FCERI mediated MAPK activation	PLCG2	
DAP12 signaling;Regulation of signaling by CBL;Role of phospholipids in phagocytosis	PIK3R1	
DCC mediated attractive signaling	PTK2	
DCC mediated attractive signaling;Factors involved in megakaryocyte development and platelet production	PDE8B	
DCC mediated attractive signaling;Netrin mediated repulsion signals	PTK2	
DCC mediated attractive signaling;VEGFA-VEGFR2 Pathway	PTK2	
Deactivation of the beta-catenin transactivating complex;Formation of the beta-catenin:TCF transactivating complex	WWOX	

Pathway	Regs Conn	Regs NConn
Deadenylation of mRNA	IDH1	
Dectin-1 mediated noncanonical NF-kB signaling;NIK–¿noncanonical NF-kB signaling	PSMB10	
Degradation of beta-catenin by the destruction complex;FBXL7 down-regulates AURKA during mitotic entry and in early mitosis;Regulation of RUNX2 expression and activity;SCF(Skp2)-mediated degradation of p27/p21;SCF-beta-TrCP mediated degradation of Emi1	PSMB10	
Degradation of beta-catenin by the destruction complex;FBXL7 down-regulates AURKA during mitotic entry and in early mitosis;Regulation of RUNX2 expression and activity;SCF(Skp2)-mediated degradation of p27/p21;SCF-beta-TrCP mediated degradation of Emi1;Vpu mediated degradation of CD4	PSMB10	
Degradation of beta-catenin by the destruction complex;Regulation of RUNX2 expression and activity	PSMB10	
Degradation of beta-catenin by the destruction complex;SCF-beta-TrCP mediated degradation of Emi1;Vpu mediated degradation of CD4	PSMB10	
Degradation of DVL;Degradation of beta-catenin by the destruction complex;FBXL7 down-regulates AURKA during mitotic entry and in early mitosis;Hedgehog 'on' state;Oxygen-dependent proline hydroxylation of Hypoxia-inducible Factor Alpha;Regulation of RUNX2 expression and activity;Regulation of expression of SLITs and ROBOs;Vif-mediated degradation of APOBEC3G	PSMB10	
Degradation of GLI1 by the proteasome;Degradation of GLI2 by the proteasome;GLI3 is processed to GLI3R by the proteasome	PSMB10	
Degradation of GLI1 by the proteasome;Hedgehog 'on' state	PSMB10	
Direct p53 effectors;E2F transcription factor network;Regulation of retinoblastoma protein		CXCL14
Disassembly of the destruction complex and recruitment of AXIN to the membrane	DVL3	
DNA Damage Recognition in GG-NER		POGZ
DNA Damage Recognition in GG-NER;Formation of Incision Complex in GG-NER		KRTAP15-1
DNA methylation	HIST2H2BE	
DNA methylation;PRC2 methylates histones and DNA	HIST2H2BE	
Downstream signaling in nave CD8+ T cells;IL12-mediated signaling events;TCR signaling in nave CD8+ T cells	ARHGEF4	
Downstream TCR signaling;Generation of second messenger molecules;Immunoregulatory interactions between a Lymphoid and a non-Lymphoid cell;PD-1 signaling;Phosphorylation of CD3 and TCR zeta chains;Translocation of ZAP-70 to Immunological synapse	ARHGEF4	
Downstream TCR signaling;Negative regulation of the PI3K/AKT network;Synthesis of PIPs at the plasma membrane	PIP5K1A	
Dual Incision in GG-NER	POLK	
Dual Incision in GG-NER;Dual incision in TC-NER	POLK	

Table B (continued)

Pathway	Regs Conn	Regs NConn
Dual Incision in GG-NER;Dual incision in TC-NER;Formation of HIV elongation complex in the absence of HIV Tat;Formation of HIV-1 elongation complex containing HIV-1 Tat;Formation of Incision Complex in GG-NER;Formation of RNA Pol II elongation complex;Formation of TC-NER Pre-Incision Complex;Formation of the Early Elongation Complex;Formation of the HIV-1 Early Elongation Complex;Gap-filling DNA repair synthesis and ligation in TC-NER;HIV Transcription Initiation;NoRC negatively regulates rRNA expression;RNA Pol II CTD phosphorylation and interaction with CE;RNA Pol II CTD phosphorylation and interaction with CE during HIV infection;RNA Polymerase I Chain Elongation;RNA Polymerase I Promoter Escape;RNA Polymerase I Transcription Initiation;RNA Polymerase I Transcription Termination;RNA Polymerase II HIV Promoter Escape;RNA Polymerase II Pre-transcription Events;RNA Polymerase II Promoter Escape;RNA Polymerase II Transcription Elongation;RNA Polymerase II Transcription Initiation;RNA Polymerase II Transcription Initiation And Promoter Clearance;RNA Polymerase II Transcription Pre-Initiation And Promoter Opening;TP53 Regulates Transcription of DNA Repair Genes;Tat-mediated elongation of the HIV-1 transcript;Transcription of the HIV genome;Transcription-Coupled Nucleotide Excision Repair (TC-NER);mRNA Capping		TNNT2
Dual Incision in GG-NER;Dual incision in TC-NER;Formation of Incision Complex in GG-NER;Formation of TC-NER Pre-Incision Complex;Gap-filling DNA repair synthesis and ligation in TC-NER;Transcription-Coupled Nucleotide Excision Repair (TC-NER)	TNNT2	
Dual incision in TC-NER	CCNH	
Dual incision in TC-NER;Formation of HIV elongation complex in the absence of HIV Tat;Formation of HIV-1 elongation complex containing HIV-1 Tat;Formation of RNA Pol II elongation complex;Formation of TC-NER Pre-Incision Complex;Gap-filling DNA repair synthesis and ligation in TC-NER;RNA Polymerase II Transcription Elongation;TP53 Regulates Transcription of DNA Repair Genes;Tat-mediated elongation of the HIV-1 transcript;Transcription-Coupled Nucleotide Excision Repair (TC-NER)	CCNH	
Dual incision in TC-NER;Formation of TC-NER Pre-Incision Complex	CCNH	
E2F transcription factor network;Regulation of retinoblastoma protein		CXCL14
E-cadherin signaling in keratinocytes;N-cadherin signaling events	PIK3R1	
Endogenous_cannabinoid_signaling;GABA-B_receptor_II_signaling;Heterotrimeric G-protein signaling pathway-Gi alpha and Gs alpha mediated pathway;Inflammation mediated by chemokine and cytokine signaling pathway;PNAT		ETV7
Energy dependent regulation of mTOR by LKB1-AMPK	IDH1	
EPHB-mediated forward signaling	PTK2	
EPHB-mediated forward signaling;VEGFA-VEGFR2 Pathway	PTK2	
EPO signaling pathway	PIK3R1	
EPO signaling pathway;Fc-epsilon receptor I signaling in mast cells	PIP5K1A	
ErbB1 downstream signaling	PIK3R1	
ErbB2/ErbB3 signaling events	PIK3R1	

Table B (continued)

Pathway	Regs Conn	Regs NConn
Erythrocytes take up carbon dioxide and release oxygen;Erythrocytes take up oxygen and release carbon dioxide;Reversible hydration of carbon dioxide;Stimuli-sensing channels		MNS1
Establishment of Sister Chromatid Cohesion	NIPAL4	
Estrogen-dependent gene expression;Formation of the beta-catenin:TCF transactivating complex	HIST2H2BE	
Estrogen-dependent gene expression;RUNX3 regulates WNT signaling	HIST2H2BE	
Eukaryotic Translation Termination;Formation of a pool of free 40S subunits;Formation of the ternary complex, and subsequently, the 43S complex;GTP hydrolysis and joining of the 60S ribosomal subunit;L13a-mediated translational silencing of Ceruloplasmin expression;Major pathway of rRNA processing in the nucleolus and cytosol;Nonsense Mediated Decay (NMD) enhanced by the Exon Junction Complex (EJC);Nonsense Mediated Decay (NMD) independent of the Exon Junction Complex (EJC);Peptide chain elongation;Regulation of expression of SLITs and ROBOs;Ribosomal scanning and start codon recognition;SRP-dependent cotranslational protein targeting to membrane;Selenocysteine synthesis;Translation initiation complex formation;Viral mRNA Translation;rRNA modification in the nucleus and cytosol	PRKACA	
Factors involved in megakaryocyte development and platelet production	PRKACA	
Fanconi Anemia Pathway;Resolution of D-loop Structures through Holliday Junction Intermediates		CDX1
FAS (CD95) signaling pathway;TRAIL signaling pathway	PIK3R1	
fatty acid activation;fatty acid alpha-oxidation II;fatty acid beta-oxidation I;fatty acid biosynthesis initiation II;stearate biosynthesis I (animals)		TERF2IP
fatty acid activation;fatty acid alpha-oxidation II;fatty acid beta-oxidation I;ketogenesis;mevalonate pathway I;stearate biosynthesis I (animals);superpathway of cholesterol biosynthesis;superpathway of geranylgeranyldiphosphate biosynthesis I (via mevalonate)	ALDH3A1	
fatty acid activation;fatty acid alpha-oxidation II;fatty acid beta-oxidation I;mevalonate pathway I;stearate biosynthesis I (animals);superpathway of cholesterol biosynthesis;superpathway of geranylgeranyldiphosphate biosynthesis I (via mevalonate)	ALDH3A1	
fatty acid activation;fatty acid alpha-oxidation II;fatty acid beta-oxidation I;oleate biosynthesis II (animals);stearate biosynthesis I (animals)	ALDH3A1	
fatty acid activation;fatty acid alpha-oxidation II;fatty acid beta-oxidation I;stearate biosynthesis I (animals)	GNPDA1	
FCERI mediated Ca+2 mobilization	PLCG2	
FCERI mediated MAPK activation	PLCG2	
Formation of HIV-1 elongation complex containing HIV-1 Tat	NTRK1	

Table B (continued)

Pathway	Regs Conn	Regs NConn
Formation of HIV elongation complex in the absence of HIV Tat;Formation of HIV-1 elongation complex containing HIV-1 Tat;Formation of RNA Pol II elongation complex;HIV elongation arrest and recovery;Pausing and recovery of HIV elongation;Pausing and recovery of Tat-mediated HIV elongation;RNA Polymerase II Pre-transcription Events;RNA Polymerase II Transcription Elongation;TP53 Regulates Transcription of DNA Repair Genes;Tat-mediated HIV elongation arrest and recovery;Tat-mediated elongation of the HIV-1 transcript	ELL	
Formation of HIV elongation complex in the absence of HIV Tat;Formation of HIV-1 elongation complex containing HIV-1 Tat;Formation of RNA Pol II elongation complex;HIV Transcription Initiation;RNA Pol II CTD phosphorylation and interaction with CE;RNA Pol II CTD phosphorylation and interaction with CE during HIV infection;RNA Polymerase II HIV Promoter Escape;RNA Polymerase II Pre-transcription Events;RNA Polymerase II Promoter Escape;RNA Polymerase II Transcription Elongation;RNA Polymerase II Transcription Initiation;RNA Polymerase II Transcription Initiation And Promoter Clearance;RNA Polymerase II Transcription Pre-Initiation And Promoter Opening;TP53 Regulates Transcription of DNA Repair Genes;Tat-mediated elongation of the HIV-1 transcript;Transcription of the HIV genome;mRNA Capping	CCNH	
Formation of HIV elongation complex in the absence of HIV Tat;Formation of HIV-1 elongation complex containing HIV-1 Tat;Formation of RNA Pol II elongation complex;RNA Pol II CTD phosphorylation and interaction with CE;RNA Pol II CTD phosphorylation and interaction with CE during HIV infection;RNA Polymerase II Transcription Elongation;TP53 Regulates Transcription of DNA Repair Genes;Tat-mediated elongation of the HIV-1 transcript;mRNA Capping	CCNH	
Formation of HIV elongation complex in the absence of HIV Tat;Formation of RNA Pol II elongation complex;HIV elongation arrest and recovery;Pausing and recovery of HIV elongation;RNA Polymerase II Pre-transcription Events;RNA Polymerase II Transcription Elongation;RNA polymerase II transcribes snRNA genes;TP53 Regulates Transcription of DNA Repair Genes	ELL	
Formation of HIV elongation complex in the absence of HIV Tat;Formation of RNA Pol II elongation complex;RNA Polymerase II Transcription Elongation;TP53 Regulates Transcription of DNA Repair Genes	CCNH	
Formation of RNA Pol II elongation complex;RNA Polymerase II Pre-transcription Events;RNA Polymerase II Transcription Elongation	ELL	
Formation of RNA Pol II elongation complex;RNA Polymerase II Transcription Elongation	CCNH	
Formation of TC-NER Pre-Incision Complex	CCNH	
Formation of TC-NER Pre-Incision Complex;Formation of the Early Elongation Complex;Formation of the HIV-1 Early Elongation Complex;RNA Pol II CTD phosphorylation and interaction with CE;TP53 Regulates Transcription of DNA Repair Genes	CCNH	

Table B (continued)

Pathway	Regs Conn	Regs NConn
Formation of the beta-catenin:TCF transactivating complex;HATs acetylate histones		POGZ
Formation of the beta-catenin:TCF transactivating complex;HATs acetylate histones;Ub-specific processing proteases		POGZ
Formation of the beta-catenin:TCF transactivating complex;PKMTs methylate histone lysines;RUNX1 regulates genes involved in megakaryocyte differentiation and platelet function	RBBP5	
Formation of tubulin folding intermediates by CCT/TriC;Prefoldin mediated transfer of substrate to CCT/TriC	BCAN	
FOXA2 and FOXA3 transcription factor networks		RABGAP1
FOXM1 transcription factor network	MYC	
G2/M Checkpoints;The role of GTSE1 in G2/M progression after G2 checkpoint	PSMB10	
G2/M DNA damage checkpoint;Meiotic recombination;Meiotic synapsis;Nonhomologous End-Joining (NHEJ);Processing of DNA double-strand break ends;Recruitment and ATM-mediated phosphorylation of repair and signaling proteins at DNA double strand breaks	HIST2H2BE	
G2/M DNA damage checkpoint;Meiotic recombination;Nonhomologous End-Joining (NHEJ);Processing of DNA double-strand break ends;Recruitment and ATM-mediated phosphorylation of repair and signaling proteins at DNA double strand breaks	HIST2H2BE	
G2/M DNA damage checkpoint;Metalloprotease DUBs;Nonhomologous End-Joining (NHEJ);Processing of DNA double-strand break ends;Recruitment and ATM-mediated phosphorylation of repair and signaling proteins at DNA double strand breaks	GCG	
G alpha (12/13) signalling events;Rho GTPase cycle;Sema4D induced cell migration and growth-cone collapse	RASGRF2	
G alpha (q) signalling events		MNS1
G alpha (q) signalling events;G alpha (s) signalling events;Vasopressin regulates renal water homeostasis via Aquaporins	NPY4R	
G alpha (s) signalling events;Vasopressin regulates renal water homeostasis via Aquaporins		MNS1
gamma-glutamyl cycle		APOA1BP
gamma-glutamyl cycle;glycogen degradation II;glycogen degradation III		APOA1BP
G beta:gamma signalling through PI3Kgamma	PIK3R1	
G beta:gamma signalling through PI3Kgamma;Interleukin receptor SHC signaling	PIK3R1	
G beta:gamma signalling through PI3Kgamma;Interleukin receptor SHC signaling;Interleukin-7 signaling	PIK3R1	
G beta:gamma signalling through PI3Kgamma;Interleukin receptor SHC signaling;RET signaling;Role of LAT2/NTAL/LAB on calcium mobilization	PIK3R1	
GDP-mannose biosynthesis;colanic acid building blocks biosynthesis	MAN2A1	
GLI3 is processed to GLI3R by the proteasome	PSMB10	
Glucuronidation		SH2D3C
Glycolysis;PP2A-mediated dephosphorylation of key metabolic factors		S100A14
GMCSF-mediated signaling events;IL3-mediated signaling events	PIK3R1	
Growth hormone receptor signaling		MCMDC2

Table B (continued)

Pathway	Regs Conn	Regs NConn
HATs acetylate histones;Ub-specific processing proteases		POGZ
HDR through Homologous Recombination (HRR);Homologous DNA Pairing and Strand Exchange;Resolution of D-loop Structures through Holliday Junction Intermediates;Resolution of D-loop Structures through Synthesis-Dependent Strand Annealing (SDSA)	RAD51D	
Hedgehog 'on' state	PSMB10	
heme biosynthesis II;tetrapyrrole biosynthesis II	ALDH3A1	
Heterotrimeric G-protein signaling pathway-Gi alpha and Gs alpha mediated pathway		HHLA1
Heterotrimeric G-protein signaling pathway-Gq alpha and Go alpha mediated pathway		ETV7
HIV Transcription Initiation;NoRC negatively regulates rRNA expression;RNA Polymerase I Chain Elongation;RNA Polymerase I Promoter Escape;RNA Polymerase I Transcription Initiation;RNA Polymerase I Transcription Termination;RNA Polymerase II HIV Promoter Escape;RNA Polymerase II Pre-transcription Events;RNA Polymerase II Promoter Escape;RNA Polymerase II Transcription Initiation;RNA Polymerase II Transcription Initiation And Promoter Clearance;RNA Polymerase II Transcription Pre-Initiation And Promoter Opening;Transcription of the HIV genome	CCNH	
HSP90 chaperone cycle for SHRs	MYC	
Hypoxia response via HIF activation;T cell activation	PIK3R1	
Hypoxic and oxygen homeostasis regulation of HIF-1-alpha	VHL	
IFN-gamma pathway	PIK3R1	
IGF1 pathway	PTK2	
IL2-mediated signaling events	PIK3R1	
IL2-mediated signaling events;SHP2 signaling	PIK3R1	
IL2 signaling events mediated by PI3K	PIK3R1	
IL2 signaling events mediated by PI3K;IL2 signaling events mediated by STAT5;IL2-mediated signaling events;SHP2 signaling	PIK3R1	
IL2 signaling events mediated by STAT5	PIK3R1	
IL6-mediated signaling events	PIK3R1	
Inflammation mediated by chemokine and cytokine signaling pathway		TNFSF10
Inhibition of voltage gated Ca2+ channels via Gbeta/gamma subunits		MNS1
Integrin alphaIIb beta3 signaling	PTK2	
Interleukin-7 signaling	PIK3R1	
Interleukin receptor SHC signaling	ARHGEF4	
Internalization of ErbB1;Signaling events mediated by focal adhesion kinase	PTK2	
Internalization of ErbB1;Signaling events mediated by TCPTP	PIK3R1	
Intra-Golgi traffic	MAN2A1	
Ion homeostasis;Ion transport by P-type ATPases;Reduction of cytosolic Ca++ levels;Sodium/Calcium exchangers		HTN1
Ion homeostasis;Regulation of insulin secretion;Stimuli-sensing channels		PEBP4
IRF3-mediated induction of type I IFN	IFI16	
JAK/STAT signaling pathway		MCMDC2
Ketone body catabolism;Pyruvate metabolism;Synthesis of Ketone Bodies		TSPAN15

Table B (continued)

Pathway	Regs Conn	Regs NConn
LPA receptor mediated events	PIK3R1	
MASTL Facilitates Mitotic Progression	ENSA	
Meiotic recombination	HIST2H2BE	
Meiotic synapsis	HIST2H2BE	
MET activates PTK2 signaling	PTK2	
Metalloprotease DUBs;RUNX1 regulates genes involved in megakaryocyte differentiation and platelet function		ANKRD46
mevalonate pathway I;superpathway of cholesterol biosynthesis;superpathway of geranylgeranyldiphosphate biosynthesis I (via mevalonate)	ALDH3A1	
mitochondrial L-carnitine shuttle pathway	ALDH3A1	
Mitochondrial translation initiation	MRPS28	
Mitotic Prometaphase;RHO GTPases Activate Formins	PAM16	
NAD biosynthesis III	PARP10	
N-cadherin signaling events	PIK3R1	
Neddylation;Oxygen-dependent proline hydroxylation of Hypoxia-inducible Factor Alpha	VHL	
Negative regulation of MAPK pathway		RPGR
NEP/NS2 Interacts with the Cellular Export Machinery;Transcriptional regulation by small RNAs;tRNA processing in the nucleus		BCAN
Neurotrophic factor-mediated Trk receptor signaling	PIK3R1	
Nongenotropic Androgen signaling	PIK3R1	
Nongenotropic Androgen signaling;Plasma membrane estrogen receptor signaling	PIK3R1	
NoRC negatively regulates rRNA expression;RNA Polymerase I Promoter Opening	HIST2H2BE	
Nuclear Pore Complex (NPC) Disassembly		HOXD9
Nuclear Pore Complex (NPC) Disassembly;Resolution of Sister Chromatid Cohesion		TSPAN7
oleate biosynthesis II (animals);stearate biosynthesis I (animals)	ALDH3A1	
Olfactory Signaling Pathway		LINC00051
Ovarian tumor domain proteases	VCPIP1	
Oxidative stress response		TMOD4
Oxygen-dependent proline hydroxylation of Hypoxia-inducible Factor Alpha	PSMB10	
Oxygen-dependent proline hydroxylation of Hypoxia-inducible Factor Alpha;Regulation of expression of SLITs and ROBOs	PSMB10	
p53 pathway		SPINK4
p53 pathway by glucose deprivation		HOXD9
p73 transcription factor network	WWOX	
PDGFR-beta signaling pathway	PIK3R1	
Phosphorylation of CD3 and TCR zeta chains	ARHGEF4	
PI3K/AKT activation	PIK3R1	
PI3 kinase pathway		MNS1
PKMTs methylate histone lysines;RUNX1 regulates genes involved in megakaryocyte differentiation and platelet function;RUNX1 regulates transcription of genes involved in differentiation of HSCs		POGZ
Plasma membrane estrogen receptor signaling	PIK3R1	

Table B (continued)

Pathway	Regs Conn	Regs NConn
PLK1 signaling events	ECT2	
PMID: 15646034;7646062	GNPDA1	
PMID: 9122178;9822713	GNPDA1	
PMID: 9187108;9256433	PTK2	
Polo-like kinase mediated events	SEC62	
PRC2 methylates histones and DNA	HIST2H2BE	
Pre-NOTCH Transcription and Translation		PRKCI
Processing of Capped Intron-Containing Pre-mRNA		FEV
Processing of Capped Intron-Containing Pre-mRNA;pre-mRNA splicing		YTHDC1
Processing of DNA double-strand break ends	HIST2H2BE	
Processing of DNA double-strand break ends;Recruitment and ATM-mediated phosphorylation of repair and signaling proteins at DNA double strand breaks	HIST2H2BE	
purine nucleotides de novo biosynthesis II		KCNK7
putrescine degradation III;spermine and spermidine degradation I	MLYCD	
Regulation of gene expression in beta cells		RABGAP1
Regulation of insulin secretion;Zinc efflux and compartmentalization by the SLC30 family;Zinc influx into cells by the SLC39 gene family	CACNB3	
Regulation of nuclear SMAD2/3 signaling;Regulation of retinoblastoma protein		CXCL14
Regulation of RAC1 activity	ARHGAP1	
Regulation of RAS by GAPs	PSMB10	
Regulation of Ras family activation	RASGRF2	
Regulation of retinoblastoma protein	MEF2C	
Regulation of RUNX2 expression and activity	PSMB10	
Regulation of signaling by CBL;Signaling by SCF-KIT	PIK3R1	
Regulation of TP53 Activity through Phosphorylation	TAF7	
Resolution of D-loop Structures through Synthesis-Dependent Strand Annealing (SDSA)	RAD51D	
Respiratory electron transport	CAMK2A	
Retinoic acid receptors-mediated signaling	CCNH	
Retinoid cycle disease events;The canonical retinoid cycle in rods (twilight vision)		LST1
Retrograde transport at the Trans-Golgi-Network	RHOBTB3	
RET signaling	PIK3R1	
Rev-mediated nuclear export of HIV RNA		BCAN
RHO GTPases Activate Formins	PAM16	
RNA Pol II CTD phosphorylation and interaction with CE;RNA Pol II CTD phosphorylation and interaction with CE during HIV infection;mRNA Capping	CCNH	
RNA Polymerase III Abortive And Retractive Initiation;RNA Polymerase III Transcription Initiation From Type 1 Promoter	BDP1	
RNA Polymerase III Abortive And Retractive Initiation;RNA Polymerase III Transcription Initiation From Type 1 Promoter;RNA Polymerase III Transcription Initiation From Type 2 Promoter	BDP1	
RNA Polymerase III Abortive And Retractive Initiation;RNA Polymerase III Transcription Initiation From Type 3 Promoter;RNA polymerase II transcribes snRNA genes	SNAPC2	

Table B (continued)

Pathway	Regs Conn	Regs NConn
RNA Polymerase I Promoter Escape;RNA Polymerase I Transcription Initiation	CCNH	
RNA Polymerase I Promoter Opening	HIST2H2BE	
RNA Polymerase I Transcription Termination	CCNH	
Role of LAT2/NTAL/LAB on calcium mobilization	PIK3R1	
SCF(Skp2)-mediated degradation of p27/p21	PSMB10	
Senescence-Associated Secretory Phenotype (SASP)	HIST2H2BE	
Signaling by BRAF and RAF fusions	TRAK1	
Signaling by cytosolic FGFR1 fusion mutants	PIK3R1	
Signaling by FGFR1 in disease	PIK3R1	
Signaling by SCF-KIT		MCMDC2
Signaling events mediated by focal adhesion kinase	PTK2	
Signaling events mediated by Stem cell factor receptor (c-Kit)	PIK3R1	
SIRT1 negatively regulates rRNA expression	HIST2H2BE	
SMAD2/SMAD3:SMAD4 heterotrimer regulates transcription	CDK8	
Smooth Muscle Contraction		PEBP4
sphingosine and sphingosine-1-phosphate metabolism	CES2	
stearate biosynthesis I (animals)	ALDH3A1	
sulfate activation for sulfonation	CHST12	
SUMOylation of chromatin organization proteins	PHC3	
SUMOylation of DNA replication proteins		HOXD9
SUMOylation of RNA binding proteins	PHC3	
Synthesis of PIPs at the Golgi membrane	VAC14	
TCR signaling in nave CD4+ T cells;TCR signaling in nave CD8+ T cells	ARHGEF4	
tetrahydrobiopterin biosynthesis I;tetrahydrobiopterin biosynthesis II	PTS	
TFAP2A acts as a transcriptional repressor during retinoic acid induced cell differentiation	NDRG1	
TFAP2A acts as a transcriptional repressor during retinoic acid induced cell differentiation;Transcription of E2F targets under negative control by p107 (RBL1) and p130 (RBL2) in complex with HDAC1	NPM1	
TFAP2 (AP-2) family regulates transcription of cell cycle factors		PTPRE
TFAP2 (AP-2) family regulates transcription of growth factors and their receptors	NDRG1	
TGF-beta signaling pathway		POGZ
TNF receptor superfamily (TNFSF) members mediating non-canonical NF-kB pathway;TNFR2 non-canonical NF-kB pathway	TNNT2	
Transcription-Coupled Nucleotide Excision Repair (TC-NER)	CCNH	
triacylglycerol biosynthesis	ALDH3A1	
Trk receptor signaling mediated by PI3K and PLC-gamma	PIK3R1	
tRNA charging	NQO2	
tRNA processing in the nucleus	POP1	
U12 Dependent Splicing	SF3B4	
U12 Dependent Splicing;pre-mRNA splicing;snRNP Assembly		FIGF
Ubiquitin proteasome pathway		ZCCHC9
UCH proteinases	PSMB10	
UCH proteinases;Ub-specific processing proteases	PSMB10	
Unwinding of DNA		SOX14

Table B (continued)

Pathway	Regs Conn	Regs NConn
Validated targets of C-MYC transcriptional activation		GSTP1
Vif-mediated degradation of APOBEC3G	PSMB10	
Vpu mediated degradation of CD4	PSMB10	

Table C. Most disrupted pathways. (N = Total number of datasets in which pathway potentially disrupted).

Pathway	Regulators	N
Downstream TCR signaling	ABCC12 ARHGEF4 ATXN7L2 HSBP1 IGSF9 MEF2C MNS1 PBXIP1 PIK3R1 PIP5K1A PLCG2 POGZ PSMB10 PTK2 RASGRF2 TERF2IP VHL	33
Generation of second messenger molecules	ARHGEF4 ATXN7L2 HSBP1 PIP5K1A PLCG2 POGZ PTK2 TERF2IP VHL	33
Antigen activates B Cell Receptor (BCR) leading to generation of second messengers	ABCC12 ARHGEF4 ATXN7L2 ETV7 GALE IGSF9 INTS8 MCMDC2 MEF2C MNS1 PBXIP1 PEBP4 PIK3R1 PIP5K1A PLCG2 PTK2 RASGRF2 TERF2IP	31
Formation of the beta-catenin:TCF transactivating complex	BCL9 CPEB4 HIST2H2BE HOXD9 LYL1 MYC NIPAL4 POGZ PYGO2 RAB3A RBBP5 WWOX ZNF446	31
RUNX1 regulates genes involved in megakaryocyte differentiation and platelet function	ANKRD46 DAAM2 HIST2H2BE HOXD9 KMT2E LYL1 NIPAL4 POGZ RAB3A RBBP5 ZNF446	31
PD-1 signaling	ARHGEF4 ATXN7L2 HSBP1 MNS1 POGZ VHL	31
Phosphorylation of CD3 and TCR zeta chains | Translocation of ZAP-70 to Immunological synapse	ARHGEF4 ATXN7L2 HSBP1 POGZ VHL	31
FCERI mediated Ca+2 mobilization | Role of phospholipids in phagocytosis	ABCC12 ETV7 IGSF9 MCMDC2 MEF2C MNS1 PBXIP1 PEBP4 PIK3R1 PIP5K1A PLCG2 PTK2 RASGRF2 TERF2IP	30
SUMOylation of DNA damage response and repair proteins	ASNSD1 GUCA2B HOXD9 LINC01587 NR3C2 NSMCE2 PHC3 SLC30A5 SSB TNNT2 TSPAN7 XRCC4	30
Ca2+ pathway	ETV7 GALE INTS8 MCMDC2 PEBP4 PIP5K1A PLCG2 PTK2 TERF2IP	30
DAG and IP3 signaling | PLC beta mediated events | VEGFR2 mediated cell proliferation	ETV7 MCMDC2 PEBP4 PIP5K1A PLCG2 PTK2 TERF2IP	30
Major pathway of rRNA processing in the nucleolus and cytosol	AGO2 BCAN CDX1 CFHR4 DENND1C DUOX1 FAM212B FCRL5 GKN1 IDH1 KCNK4 KDELR1 KLK6 MYC NIP7 PDE4C PRKACA PROCR RIPK3 RPL13A RPS23 RPS4X SEC24D SF3B4 STARD4 TLE2 ZCCHC9	29

Table C (continued)

Pathway	Regulators	N
Transcriptional regulation by small RNAs	AGO2 ASNSD1 BCAN GIMAP2 GUCA2B HIST2H2BE HOXD9 LINC01587 LYL1 NIPAL4 NR3C2 RAB3A SLC30A5 SSB TNNT2 TSPAN7 ZNF446	29
snRNP Assembly	ASNSD1 CFHR4 FIGF GCG GEMIN7 GEMIN8 GUCA2B HOXD9 LINC01587 NR3C2 POGZ SLC30A5 SSB TNNT2 TSPAN7	29
GPVI-mediated activation cascade	ABCC12 GALE IGSF9 INTS8 MEF2C MNS1 PBXIP1 PIK3R1 PIP5K1A PLCG2 PTK2 RAS-GRF2 TERF2IP	29
Estrogen-dependent gene expression	AGO2 CHD1 HIST2H2BE IQ-GAP2 LYL1 MYC NIPAL4 PIEZO1 POGZ RAB3A ZNF446	29
FGF signaling pathway	ARHGEF4 COTL1 GAB2 PIK3R1 PLCG2 POGZ PTK2 SELPLG TN-FSF10	29
G alpha (q) signalling events	INTS8 MNS1 NPY4R PIP5K1A PLCG2 PTK2 TERF2IP	29
Activation of anterior HOX genes in hindbrain development during early embryogenesis	DAAM2 HIST2H2BE LYL1 NCOA6 NIPAL4 PARP10 PAX6 PLCG2 POGZ RAB3A RBBP5 VHL ZNF446	28
PKMTs methylate histone lysines	ASH1L DAAM2 DQX1 KMT2E LYL1 MECOM PARP10 POGZ RBBP5	28
G beta:gamma signalling through PLC beta	GALE INTS8 PIP5K1A PLCG2 PTK2 TERF2IP	28
MHC class II antigen presentation	DCTN4 GIMAP2 HSBP1 POGZ RASGRF2 RILP	28
Acetylcholine regulates insulin secretion | Fatty Acids bound to GPR40 (FFAR1) regulate insulin secretion | Role of second messengers in netrin-1 signaling | Syndecan interactions | Synthesis of IP3 and IP4 in the cytosol	INTS8 PIP5K1A PLCG2 PTK2 TERF2IP	28
CLEC7A (Dectin-1) signaling	PIP5K1A PLCG2 PSMB10 PTK2 TERF2IP	28
Transport of Mature mRNA derived from an Intron-Containing Transcript	ASNSD1 BCAN GUCA2B HOXD9 LINC01587 MRPL54 NPIPB15 NR3C2 PROCR SLC30A5 SMOC2 SSB TNNT2 TSPAN7 ZC3H3	27
Regulation of actin dynamics for phagocytic cup formation	ABCC12 ATXN7L2 IGSF9 MEF2C MNS1 PBXIP1 PIK3R1 PIP5K1A PLCG2 PTK2 RASGRF2 RFTN1	27

Table C (continued)

Pathway	Regulators	N
Synthesis of PIPs at the plasma membrane	ABCC12 ATXN7L2 GALE IGSF9 MEF2C MNS1 PBXIP1 PIK3R1 PIP5K1A PLCG2 PTK2 RASGRF2	27
G beta:gamma signalling through PI3Kgamma | Signaling by SCF-KIT	ABCC12 IGSF9 MCMDC2 MEF2C MNS1 PBXIP1 PIK3R1 PIP5K1A PLCG2 PTK2 RASGRF2	27
Role of LAT2/NTAL/LAB on calcium mobilization	ABCC12 ATXN7L2 IGSF9 MEF2C MNS1 PBXIP1 PIK3R1 PIP5K1A PLCG2 PTK2 RASGRF2	27
TCR signaling in nave CD4+ T cells	ARHGEF4 ATXN7L2 COTL1 GAB2 HSBP1 KCNA2 MCMDC2 PIK3R1 PLCG2 PTK2 SELPLG	27
CXCR4-mediated signaling events	ARHGEF4 ATXN7L2 COTL1 GAB2 HSBP1 MCMDC2 PIK3R1 PLCG2 PTK2 SELPLG	27
Clathrin-mediated endocytosis	ATXN7L2 MYO1D PFKFB1 PIK3R1 PIP5K1A PLCG2 PTK2 RASGRF2	27
Interferon gamma signaling	HSBP1 POGZ	27
Antigen processing: Ubiquitination and Proteasome degradation	ATG7 BCAN FCRL2 LRSAM1 MRPL54 NPIPB15 NQO2 PIK3R1 PMP22 PSMB10 SLC25A2 VHL ZCCHC9	26
tRNA processing in the nucleus	ASNSD1 BCAN GUCA2B HOXD9 LINC01587 NR3C2 POP1 SLC30A5 SSB TNNT2 TSPAN7 ZNF446	26
PI5P, PP2A and IER3 Regulate PI3K/AKT Signaling	ABCC12 GALE IGSF9 MEF2C MNS1 PBXIP1 PIK3R1 PIP5K1A PLCG2 PTK2 RASGRF2	26
RHO GTPases Activate WASPs and WAVEs	ABCC12 IGSF9 MEF2C MNS1 PBXIP1 PIK3R1 PIP5K1A PLCG2 PTK2 RASGRF2 RFTN1	26

Table C (continued)

Pathway	Regulators	N
Activated NTRK2 signals through PI3K | CD28 dependent PI3K/Akt signaling | Constitutive Signaling by Aberrant PI3K in Cancer | Constitutive Signaling by EGFRvIII | Constitutive Signaling by Ligand-Responsive EGFR Cancer Variants | DAP12 signaling | Downstream signal transduction | FCERI mediated MAPK activation | GAB1 signalosome | MET activates PI3K/AKT signaling | Negative regulation of the PI3K/AKT network | PI3K/AKT activation | PI3K/AKT Signaling | PI3K Cascade | PI-3K cascade:FGFR1 | PI-3K cascade:FGFR2 | PI-3K cascade:FGFR3 | PI-3K cascade:FGFR4 | PI3K events in ERBB2 signaling | PI3K events in ERBB4 signaling | PI Metabolism | RIPK1-mediated regulated necrosis | Signaling by cytosolic FGFR1 fusion mutants | Signaling by FGFR1 in disease | Signaling by FGFR2 in disease | Signaling by FGFR3 fusions in cancer | Signaling by FGFR3 point mutants in cancer | Signaling by FGFR4 in disease | VEGFA-VEGFR2 Pathway	ABCC12 IGSF9 MEF2C MNS1 PBXIP1 PIK3R1 PIP5K1A PLCG2 PTK2 RASGRF2	26
SUMOylation of chromatin organization proteins | SUMOylation of RNA binding proteins	ASNSD1 GUCA2B HOXD9 LINC01587 NR3C2 PHC3 SLC30A5 SSB TNNT2 TSPAN7	26
Ub-specific processing proteases	ADRB2 AR HIST2H2BE MYC PIK3R1 POGZ PSMB10 USP13	26
Cargo recognition for clathrin-mediated endocytosis	ATXN7L2 MYO1D PIP5K1A PTK2 RASGRF2	26
EGFR Transactivation by Gastrin	INTS8 PLCG2 PTK2 TERF2IP	26
DCC mediated attractive signaling	PDE8B PIP5K1A PTK2	26
pre-mRNA splicing	ASNSD1 DHX16 EMILIN1 FIGF GUCA2B ICAM3 IDH1 KLHL24 MRPL54 NIPAL4 NPIPB15 PCDHB9 PROCR PURA SF3B4 SMOC2 TCERG1 TMEM259 TNNT2 YTHDC1 ZNF446	25
Nonsense Mediated Decay (NMD) enhanced by the Exon Junction Complex (EJC)	DENND1C DUOX1 FCRL5 IDH1 KDELR1 KLK6 PAX6 PHC3 PRKACA PROCR RPL13A RPS23 RPS4X STARD4 TLE2 ZCCHC9	25
Transport of Mature mRNA Derived from an Intronless Transcript	ASNSD1 GUCA2B HOXD9 ICAM3 LINC01587 NR3C2 SLC30A5 SSB TNNT2 TSPAN7 ZCCHC9	25
RUNX1 regulates transcription of genes involved in differentiation of HSCs	CCNH DAPK3 HIST2H2BE LYL1 NIPAL4 PIK3R1 POGZ PSMB10 RAB3A ZNF446	25

Table C (continued)

Pathway	Regulators	N
Fc-epsilon receptor I signaling in mast cells	ARHGEF4 PIK3R1 PIP5K1A PLCG2 PTK2 RASGRF2 SELPLG	25
Integrin signalling pathway	CX3CL1 DOCK1 FPR2 PIK3R1 PTK2 PTPRE ZEB1	25
E-cadherin signaling in keratinocytes	ARHGEF4 PIK3R1 PIP5K1A PLCG2 PTK2 SELPLG	25
ER-Phagosome pathway	PIK3R1 PIP5K1A PSMB10 PTK2	25
HATs acetylate histones	ANKRD46 HIST2H2BE PARP10 POGZ	25
WNT mediated activation of DVL	PIK3R1 PIP5K1A PLCG2 PTK2	25
EPHB-mediated forward signaling	PIP5K1A PLCG2 PTK2	25
Constitutive Signaling by AKT1 E17K in Cancer | EGFR downregulation | GRB2:SOS provides linkage to MAPK signaling for Integrins | Integrin alphaIIb beta3 signaling | IRAK4 deficiency (TLR2/4) | MyD88 deficiency (TLR2/4) | MyD88:Mal cascade initiated on plasma membrane | Netrin-1 signaling | p130Cas linkage to MAPK signaling for integrins | Recycling pathway of L1	PIP5K1A PTK2	25
Separation of Sister Chromatids	ASNSD1 AURKC CDX1 FCRL2 GUCA2B KCNMB3 LINC01587 LSM11 MRPL54 NAALADL2 NIPAL4 NQO2 PSMB10 RPL13A SGCZ SLC25A2 SLC27A3 SOX14 SSB TBCK TSPAN7 USP13	24
Processing of DNA double-strand break ends	C14orf1 CCNH DAPK3 DCTN4 GALE GCG HIST2H2BE HOXD9 IL6R KLK11 LYL1 MCTP1 MRPL54 NIPAL4 PAM RNF139 SLC39A13 TMOD4 TNNT2 UBE2T XRCC4	24
GTP hydrolysis and joining of the 60S ribosomal subunit	BCAN DENND1C DUOX1 FCRL5 GKN1 IDH1 KDELR1 KLK6 MRPL54 MUC5B PRKACA RPL13A RPS23 RPS4X STARD4 TLE2 VPS8 ZCCHC9	24
Formation of a pool of free 40S subunits	DENND1C DUOX1 FCRL5 GKN1 IDH1 KDELR1 KLK6 MRPL54 MUC5B PRKACA RPL13A RPS23 RPS4X STARD4 TLE2 VPS8 ZCCHC9	24
L13a-mediated translational silencing of Ceruloplasmin expression	BCAN DENND1C DUOX1 GKN1 IDH1 KDELR1 KLK6 MRPL54 MUC5B PRKACA RPL13A RPS23 RPS4X STARD4 TLE2 VPS8 ZCCHC9	24

Table C (continued)

Pathway	Regulators	N
Regulation of expression of SLITs and ROBOs	DENND1C DUOX1 FCRL5 IDH1 ISL1 KDELR1 KLK6 PIK3R1 PRKACA PROCR PSMB10 RPL13A RPS23 RPS4X STARD4 TLE2 ZCCHC9	24
NEP/NS2 Interacts with the Cellular Export Machinery | Rev-mediated nuclear export of HIV RNA	ASNSD1 BCAN GUCA2B HOXD9 LINC01587 NR3C2 SLC30A5 SSB TNNT2 TSPAN7	24
Regulation of HSF1-mediated heat shock response	ASNSD1 GUCA2B HOXD9 LINC01587 NR3C2 SLC30A5 SLC39A13 SSB TNNT2 TSPAN7	24
Signaling events mediated by VEGFR1 and VEGFR2 | VEGFR1 specific signals	ARHGEF4 COTL1 GAB2 HOXD9 MCMDC2 PIK3R1 PLCG2 PTK2 SELPLG TNFSF10	24
ErbB1 downstream signaling | PDGFR-beta signaling pathway	ARHGEF4 COTL1 GAB2 MCMDC2 PIK3R1 PLCG2 PTK2 SELPLG TNFSF10	24
NS1 Mediated Effects on Host Pathways | Nuclear import of Rev protein | Nuclear Pore Complex (NPC) Disassembly | Regulation of Glucokinase by Glucokinase Regulatory Protein | SUMOylation of DNA replication proteins | Transport of Ribonucleoproteins into the Host Nucleus | Transport of the SLBP Dependant Mature mRNA | Transport of the SLBP independent Mature mRNA | Viral Messenger RNA Synthesis | Vpr-mediated nuclear import of PICs	ASNSD1 GUCA2B HOXD9 LINC01587 NR3C2 SLC30A5 SSB TNNT2 TSPAN7	24
Plasma membrane estrogen receptor signaling	ARHGEF4 COTL1 GAB2 HOXD9 MCMDC2 PIK3R1 PLCG2 PTK2 SELPLG	24
Activation of NF-kappaB in B cells	ETV7 INTS8 MCMDC2 PEBP4 PIK3R1 PLCG2 PSMB10 TERF2IP	24
BCR signaling pathway | Class I PI3K signaling events | IL8- and CXCR1-mediated signaling events | IL8- and CXCR2-mediated signaling events | Nephrin/Neph1 signaling in the kidney podocyte | Signaling events mediated by Hepatocyte Growth Factor Receptor (c-Met) | TCR signaling in nave CD8+ T cells | Trk receptor signaling mediated by PI3K and PLC-gamma	ARHGEF4 COTL1 GAB2 MCMDC2 PIK3R1 PLCG2 PTK2 SELPLG	24
Cleavage of Growing Transcript in the Termination Region | mRNA 3'-end processing	BCAN ICAM3 MRPL54 NPIPB15 PROCR SMOC2 ZC3H3 ZNF446	24
Neddylation	BCAN C14orf1 KLHL3 PIK3R1 PMP22 PROCR PSMB10 VHL	24
CXCR3-mediated signaling events | IFN-gamma pathway	ARHGEF4 COTL1 GAB2 MCMDC2 PIK3R1 PTK2 TNFSF10	24

Table C (continued)

Pathway	Regulators	N
EPHA2 forward signaling | ErbB2/ErbB3 signaling events	ARHGEF4 COTL1 GAB2 MCMDC2 PIK3R1 PTK2	24
ABC-family proteins mediated transport	DERL1 PGK2 PIK3R1 PSMB10	24
VEGF signaling pathway	DCP2 PIK3R1 PLCG2 PTK2	24
EGF receptor signaling pathway	PEBP4 PLCG2 POGZ	24
Regulation of ornithine decarboxylase (ODC)	AZIN1 PIK3R1 PSMB10	24
Mitotic Prometaphase | RHO GTPases Activate Formins	ASNSD1 AURKC GUCA2B KCNMB3 LINC01587 LSM11 MRPL54 NAALADL2 PAM16 RPL13A SGCZ SLC27A3 SOX14 SSB TBCK TSPAN7 USP13	23
Resolution of Sister Chromatid Cohesion	ASNSD1 AURKC GUCA2B KCNMB3 LINC01587 LSM11 MRPL54 NAALADL2 NIPAL4 RPL13A SGCZ SLC27A3 SOX14 SSB TBCK TSPAN7 USP13	23
SRP-dependent cotranslational protein targeting to membrane	CLDND2 DENND1C DUOX1 FCRL5 IDH1 KCNN3 KDELR1 KLK6 PRKACA RPL13A RPRM RPS23 RPS4X SSB STARD4 TLE2 ZCCHC9	23
Amplification of signal from unattached kinetochores via a MAD2 inhibitory signal	ASNSD1 AURKC GUCA2B KCNMB3 LINC01587 LSM11 MRPL54 NAALADL2 RPL13A SGCZ SLC27A3 SOX14 SSB TBCK TSPAN7 USP13	23
VEGFR2 mediated vascular permeability	ABCC12 ETV7 IGSF9 MCMDC2 MEF2C MNS1 PBXIP1 PEBP4 PIK3R1 PIP5K1A PLCG2 RASGRF2	23
Fanconi Anemia Pathway	CDX1 DCTN4 GALE IL6R KLK11 PAM RNF139 UBE2T	23
Presynaptic phase of homologous DNA pairing and strand exchange	HOXD9 IL6R PAM RAD51D RNF139 SULT4A1 TMOD4 UBE2T	23
Condensation of Prophase Chromosomes	HIST2H2BE LYL1 NIPAL4 POGZ RAB3A WDR41 ZNF446	23
Elevation of cytosolic Ca2+ levels	ETV7 HTN1 INTS8 MCMDC2 PEBP4 PLCG2 TERF2IP	23
Nongenotropic Androgen signaling	ARHGEF4 PIK3R1 PLCG2 PTK2 RASGRF2 SELPLG TNFSF10	23
Effects of PIP2 hydrolysis | Rap1 signalling | Response to elevated platelet cytosolic Ca2+	ETV7 INTS8 MCMDC2 PEBP4 PLCG2 TERF2IP	23
Mitochondrial translation elongation	ADAMTSL4 DHX16 FIGF MRPL54 MRPS28 SLC39A13	23
Netrin-mediated signaling events	ARHGEF4 PIK3R1 PLCG2 PTK2 SELPLG TNFSF10	23
RNA polymerase II transcribes snRNA genes	ELL ELL2 ELL3 PITX1 POGZ SNAPC2	23

Table C (continued)

Pathway	Regulators	N
LPA receptor mediated events	ARHGEF4 PIK3R1 PLCG2 PTK2 SELPLG	23
Thromboxane A2 receptor signaling	ARHGEF4 HOXD9 PLCG2 PTK2 SELPLG	23
Downstream signaling in nave CD8+ T cells	ARHGEF4 PIK3R1 PLCG2 PTK2	23
E2F transcription factor network	CES2 CXCL14 DHFR MYC	23
PAR1-mediated thrombin signaling events | PDGFR-alpha signaling pathway | Role of Calcineurin-dependent NFAT signaling in lymphocytes	ARHGEF4 PLCG2 PTK2 SELPLG	23
Signaling events regulated by Ret tyrosine kinase	COTL1 PIK3R1 PTK2 TNFSF10	23
Angiogenesis	PIK3R1 PLCG2 PTK2	23
Defective CFTR causes cystic fibrosis	DERL1 PIK3R1 PSMB10	23
HDMs demethylate histones	DERL1 MAN2A1 POLD3	23
Eukaryotic Translation Termination | Nonsense Mediated Decay (NMD) independent of the Exon Junction Complex (EJC) | Peptide chain elongation | Selenocysteine synthesis | Viral mRNA Translation	DENND1C DUOX1 FCRL5 IDH1 KDELR1 KLK6 PRKACA RPL13A RPS23 RPS4X STARD4 TLE2 ZCCHC9	22
rRNA modification in the nucleus and cytosol	AGO2 BCAN CDX1 CFHR4 GKN1 KCNK4 PRKACA SEC24D SF3B4	22
Recruitment and ATM-mediated phosphorylation of repair and signaling proteins at DNA double strand breaks	GCG HIST2H2BE MAN2A1 MRPL54 NIPAL4 PAM RNF139 TMOD4	22
B-WICH complex positively regulates rRNA expression	ANKRD46 HIST2H2BE LYL1 NIPAL4 RAB3A SYT11 ZNF446	22
FCERI mediated NF-kB activation	ETV7 MCMDC2 PEBP4 PIK3R1 PLCG2 PSMB10 TERF2IP	22
Osteopontin-mediated events	ARHGEF4 COTL1 GAB2 PIK3R1 PIP5K1A PLCG2 TNFSF10	22
Regulation of TP53 Activity through Phosphorylation	HOXD9 IL6R PAM RNF139 TAF7 TMOD4 UBE2T	22
HDR through Single Strand Annealing (SSA)	HOXD9 IL6R PAM RNF139 TMOD4 UBE2T	22
Fanconi anemia pathway	COL4A5 HOXD9 PAM RNF139 UBE2T	22
Mitochondrial translation initiation | Mitochondrial translation termination	ADAMTSL4 FIGF MRPL54 MRPS28 SLC39A13	22
Alpha4 beta1 integrin signaling events	CX3CL1 JAML PTK2	22
Prefoldin mediated transfer of substrate to CCT/TriC	AGO2 BCAN FAM212B	22
IGF1 pathway | Internalization of ErbB1	PIK3R1 PTK2	22
Gap-filling DNA repair synthesis and ligation in TC-NER	BCAN C14orf1 CCNH DAPK3 HOXD9 IL6R LYL1 MCTP1 PAM POLK RNF139 TMOD4 TNNT2	21
Nonhomologous End-Joining (NHEJ)	GCG HIST2H2BE IL6R MRPL54 NIPAL4 PAM RNF139 TMOD4 XRCC4	21

Table C (continued)

Pathway	Regulators	N
HDR through Homologous Recombination (HRR)	HOXD9 IL6R PAM POLK RAD51D RNF139 SULT4A1 TMOD4	21
HDR through MMEJ (alt-NHEJ)	CCNH HOXD9 IL6R MCTP1 PAM RNF139 TMOD4 XRCC4	21
Gap-filling DNA repair synthesis and ligation in GG-NER	CCNH HOXD9 IL6R PAM POLK RNF139 TMOD4	21
Termination of translesion DNA synthesis	HOXD9 IL6R MYO1D PAM POLK RNF139 TMOD4	21
Activation of RAS in B cells | Arachidonate production from DAG	INTS8 PLCG2 TERF2IP	21
IL12-mediated signaling events | IL12 signaling mediated by STAT4	ARHGEF4 ATXN7L2 HSBP1	21
ATR signaling pathway	RNF139 YWHAZ	21
Calcium signaling in the CD4+ TCR pathway | Effects of Botulinum toxin | Noncanonical Wnt signaling pathway | PAR4-mediated thrombin signaling events | Visual signal transduction: Cones | Visual signal transduction: Rods	ARHGEF4 PTK2	21
E3 ubiquitin ligases ubiquitinate target proteins	DERL1 HOXD9	21
9256433 | Activated NTRK2 signals through FRS2 and FRS3 | Activated NTRK2 signals through RAS | CD209 (DC-SIGN) signaling | FRS-mediated FGFR1 signaling | FRS-mediated FGFR2 signaling | FRS-mediated FGFR3 signaling | FRS-mediated FGFR4 signaling | GRB2 events in EGFR signaling | GRB2 events in ERBB2 signaling | Insulin receptor signalling cascade | MET activates PTK2 signaling | MET activates RAS signaling | NCAM signaling for neurite out-growth | Netrin mediated repulsion signals | P|: 9187108| RAF/MAP kinase cascade | SHC1 events in EGFR signaling | SHC1 events in ERBB2 signaling | SHC1 events in ERBB4 signaling | SHC-mediated cascade:FGFR1 | SHC-mediated cascade:FGFR2 | SHC-mediated cascade:FGFR3 | SHC-mediated cascade:FGFR4 | SHC-related events triggered by IGF1R | Signaling events mediated by focal adhesion kinase | Signalling to RAS | SOS-mediated signalling | Tie2 Signaling	PTK2	21
Formation of the ternary complex, and subsequently, the 43S complex | Ribosomal scanning and start codon recognition | Translation initiation complex formation	BCAN DENND1C GKN1 IDH1 KDELR1 MRPL54 MUC5B PRKACA RPS23 RPS4X TLE2 VPS8	20

Table C (continued)

Pathway	Regulators	N
Activation of PKB | CD28 dependent Vav1 pathway | FGFR2 | FGFR3 | FGFR4 | Inhibition of TSC complex formation by PKB | PDE3B signalling | Phospholipase C-mediated cascade | Phospholipase C-mediated cascade: FGFR1 | PTEN Loss of Function in Cancer | Regulation of TP53 Degradation | RHO GTPases activate PKNs	ABCC12 IGSF9 MEF2C MNS1 PBXIP1 PIK3R1 PIP5K1A PLCG2 RASGRF2	20
Meiotic recombination	HIST2H2BE HOXD9 LYL1 NIPAL4 PAM RAB3A RNF139 TMOD4 ZNF446	20
Resolution of D-loop Structures through Holliday Junction Intermediates	CDX1 HOXD9 IL6R PAM RAD51D RNF139 SULT4A1 TMOD4	20
DNA Damage/Telomere Stress Induced Senescence	HIST2H2BE NIPAL4 PAM RAB3A RNF139 SSB ZNF446	20
G2/M DNA damage checkpoint	GCG HIST2H2BE MRPL54 NIPAL4 PAM RNF139 TMOD4	20
Homologous DNA Pairing and Strand Exchange | Resolution of D-loop Structures through Synthesis-Dependent Strand Annealing (SDSA)	HOXD9 IL6R PAM RAD51D RNF139 SULT4A1 TMOD4	20
IL2 signaling events mediated by PI3K	ARHGEF4 COTL1 GAB2 MCMDC2 PIK3R1 PLCG2 SELPLG	20
EPO signaling pathway	ARHGEF4 PIK3R1 PIP5K1A PLCG2 SELPLG	20
fatty acid beta-oxidation I	ALDH3A1 GNPDA1 MLYCD TERF2IP TSPAN15	20
DNA Damage Recognition in GG-NER	KRTAP15-1 POGZ	20
TGF-beta signaling pathway	POGZ	20
HDACs deacetylate histones	HDAC10 HIST2H2BE HOXD9 LYL1 NIPAL4 PARP10 RAB3A SAP30L SOX14 TAF7 TBL1XR1 WFIKKN1	19
Regulation of Ras family activation	ARHGEF4 COTL1 GAB2 PIK3R1 PLCG2 RASGRF2 SELPLG	19
GMCSF-mediated signaling events	ARHGEF4 COTL1 GAB2 MCMDC2 PIK3R1 TNFSF10	19
Rho GTPase cycle	ARHGAP30 ARHGEF4 LRRIQ4 MYO1D NSMCE2 RASGRF2	19
E-cadherin signaling in the nascent adherens junction | FAS (CD95) signaling pathway | IL4-mediated signaling events | Insulin Pathway	ARHGEF4 COTL1 GAB2 MCMDC2 PIK3R1	19
PCNA-Dependent Long Patch Base Excision Repair | Translesion Synthesis by POLH | Translesion synthesis by POLI | Translesion synthesis by POLK | Translesion synthesis by REV1	HOXD9 IL6R PAM RNF139 TMOD4	19
U12 Dependent Splicing	FIGF NPIPB15 SF3B4 SMOC2 TCERG1	19

Table C (continued)

Pathway	Regulators	N
fatty acid biosynthesis initiation II	MLYCD TERF2IP	19
S-adenosyl-L-methionine biosynthesis	CES2 POLD3	19
NoRC negatively regulates rRNA expression	CCNH DAPK3 HIST2H2BE HOXD9 LYL1 NIPAL4 RAB3A SAP30L SYT11 TAF7 TNNT2 ZNF446	18
Deposition of new CENPA-containing nucleosomes at the centromere	HIST2H2BE KCNMB3 LYL1 NIPAL4 RAB3A S100A14 SRPX TBCK USP13 ZNF446	18
RNA Polymerase II Pre-transcription Events	ATG10 CCNH DAPK3 ELL FBLN1 NTRK1 PARP10 SLC25A37 TAF7 TNNT2	18
Regulation of insulin secretion	CACNB3 ETV7 MCMDC2 PEBP4 PLCG2	18
Arf6 signaling events | Class I PI3K signaling events mediated by Akt | RAC1 signaling pathway	ARHGEF4 COTL1 GAB2 PIK3R1	18
Invadopodia formation | PIPs transport between plasma and early endosome membranes	ATXN7L2 PIK3R1 PIP5K1A RASGRF2	18
PDGF signaling pathway	DCP2 IQGAP2 PIK3R1 PLCG2	18
TNFR2 non-canonical NF-kB pathway	PIK3R1 POLD3 PSMB10 TNNT2	18
Degradation of beta-catenin by the destruction complex	PIK3R1 POLD3 PSMB10	18
Interleukin receptor SHC signaling	ARHGEF4 MCMDC2 PIK3R1	18
Oxygen-dependent proline hydroxylation of Hypoxia-inducible Factor Alpha	PIK3R1 PSMB10 VHL	18
Sensing of DNA Double Strand Breaks	PAM RNF139	18
Anchoring of the basal body to the plasma membrane	ADH1C ATG10 CERK KRTAP15-1 LSM11 MRPL54 PDE8B SOX14 TMEM37	17
COPI-mediated anterograde transport	ADGRB1 DCTN4 GIMAP2 KDELR2 MEF2C	17
Inflammation mediated by chemokine and cytokine signaling pathway	DCP2 ETV7 PIK3R1 PLCG2 TNFSF10	17
CDT1 association with the CDC6:ORC:origin complex	PIK3R1 PSMB10 S100A14 VPS8	17
fatty acid activation | fatty acid alpha-oxidation II | stearate biosynthesis I (animals)	ALDH3A1 GNPDA1 TERF2IP TSPAN15	17
FBXL7 down-regulates AURKA during mitotic entry and in early mitosis	PIK3R1 PMP22 PSMB10	17
BARD1 signaling events	PAM UBE2T	17
Metalloprotease DUBs	ANKRD46 GCG	17
Sphingolipid de novo biosynthesis	PLCG2 TERF2IP	17
FOXA2 and FOXA3 transcription factor networks | Regulation of gene expression in beta cells	RABGAP1	17
Signaling by BRAF and RAF fusions	TRAK1	17

Table C (continued)

Pathway	Regulators	N
HIV Transcription Initiation | RNA Polymerase II HIV Promoter Escape | RNA Polymerase II Promoter Escape | RNA Polymerase II Transcription Initiation | RNA Polymerase II Transcription Initiation And Promoter Clearance | RNA Polymerase II Transcription Pre-Initiation And Promoter Opening | Transcription of the HIV genome	ATG10 CCNH DAPK3 FBLN1 PARP10 SLC25A37 TAF7 TNNT2	16
Ion homeostasis	ETV7 HTN1 LINC00051 MCMDC2 PART1 PEBP4 PGK2 PLCG2	16
TP53 Regulates Transcription of DNA Repair Genes	CCNH DAPK3 ELL NTRK1 PROCR RAD51D S100A14 TNNT2	16
Oxidative Stress Induced Senescence	ASNSD1 HIST2H2BE LYL1 NIPAL4 PHC3 RAB3A ZNF446	16
APC/C:Cdc20 mediated degradation of Securin | APC/C:Cdh1 mediated degradation of Cdc20 and other APC/C:Cdh1 targeted proteins in late mitosis/early G1 | Autodegradation of Cdh1 by Cdh1:APC/C | Cdc20:Phospho-APC/C mediated degradation of Cyclin A | CDK-mediated phosphorylation and removal of Cdc6	CDX1 FCRL2 NQO2 PIK3R1 PSMB10 SLC25A2	16
Cyclin E associated events during G1/S transition	CCNH DAPK3 LYL1 MYC SEC62	16
CLEC7A (Dectin-1) induces NFAT activation	ETV7 MCMDC2 PEBP4 PLCG2	16
Degradation of DVL	BCAN DVL3 PIK3R1 PSMB10	16
Carboxyterminal post-translational modifications of tubulin	APOA1BP IQGAP2 NUP210L	16
glutaryl-CoA degradation | ketogenesis | ketolysis | mevalonate pathway I | superpathway of cholesterol biosynthesis | superpathway of geranylgeranyldiphosphate biosynthesis I (via mevalonate) | tryptophan degradation III (eukaryotic)	ALDH3A1 MLYCD TSPAN15	16
HSP90 chaperone cycle for SHRs	DCTN4 GIMAP2 MYC	16
Interleukin-3, 5 and GM-CSF signaling	MCMDC2 PIK3R1	16
Regulation of Telomerase	PAM TNFSF10	16
Transcriptional Regulation by E2F6	CXCL14 PHC3	16
Deactivation of the beta-catenin transactivating complex | p73 transcription factor network	WWOX	16
Interconversion of nucleotide di- and triphosphates | Purine catabolism | Purine salvage	ATG7	16
Ovarian tumor domain proteases	VCPIP1	16
RNA Polymerase I Chain Elongation	CCNH DAPK3 HIST2H2BE LYL1 NIPAL4 RAB3A SYT11 TNNT2 ZNF446	15
CDC42 signaling events	PIK3R1 PLCG2 RASGRF2 TNFSF10	15
Orc1 removal from chromatin	CXCL14 PIK3R1 PSMB10 S100A14	15

Table C (continued)

Pathway	Regulators	N
Activation of ATR in response to replication stress	CXCL14 RNF139 S100A14	15
Degradation of AXIN | Hedgehog 'on' state	BCAN PIK3R1 PSMB10	15
EPHB forward signaling	PLCG2 RASGRF2 TNFSF10	15
SHP2 signaling	HOXD9 PIK3R1 PLCG2	15
Activated NOTCH1 Transmits Signal to the Nucleus	FAM83E ZBTB43	15
ATM pathway	PAM XRCC4	15
Condensation of Prometaphase Chromosomes	ATG10 WDR41	15
Cytosolic tRNA aminoacylation	APOA1BP IQGAP2	15
Intra-Golgi traffic	MAN2A1 MEF2C	15
TFAP2A acts as a transcriptional repressor during retinoic acid induced cell differentiation	NDRG1 NPM1	15
tetrahydrobiopterin biosynthesis I | tetrahydrobiopterin biosynthesis II	PTS	15
Dual incision in TC-NER	BCAN C14orf1 CCNH DAPK3 LYL1 MCTP1 POLK TNNT2	14
Formation of HIV-1 elongation complex containing HIV-1 Tat | Formation of HIV elongation complex in the absence of HIV Tat | Formation of RNA Pol II elongation complex | RNA Polymerase II Transcription Elongation | Tat-mediated elongation of the HIV-1 transcript	CCNH DAPK3 ELL NTRK1 TNNT2	14
Endothelins	PLCG2 SELPLG TNFSF10	14
Polo-like kinase mediated events | Transcription of E2F targets under negative control by DREAM complex	LYL1 MYC SEC62	14

Table C (continued)

Pathway	Regulators	N
Asymmetric localization of PCP proteins | AUF1 (hnRNP D0) binds and destabilizes mRNA | Autodegradation of the E3 ubiquitin ligase COP1 | Cross-presentation of soluble exogenous antigens (endosomes) | Dectin-1 mediated noncanonical NF-kB signaling | Degradation of GLI1 by the proteasome | Degradation of GLI2 by the proteasome | G2/M Checkpoints | GLI3 is processed to GLI3R by the proteasome | Hedgehog ligand biogenesis | Hh mutants that don't undergo autocatalytic processing are degraded by ERAD | Interleukin-1 signaling | MAPK6/MAPK4 signaling | Regulation of activated PAK-2p34 by proteasome mediated degradation | Regulation of PTEN stability and activity | Regulation of RAS by GAPs | Regulation of RUNX2 expression and activity | Regulation of RUNX3 expression and activity | SCF-beta-TrCP mediated degradation of Emi1 | SCF(Skp2)-mediated degradation of p27/p21 | Ubiquitin-dependent degradation of Cyclin D1 | Ubiquitin Mediated Degradation of Phosphorylated Cdc25A | UCH proteinases | Vif-mediated degradation of APOBEC3G | Vpu mediated degradation of CD4	PIK3R1 PSMB10	14
Aurora B signaling	ATG10 RASA1	14
Constitutive Signaling by NOTCH1 HD+PEST Domain Mutants | Constitutive Signaling by NOTCH1 PEST Domain Mutants	HIST2H2BE ZBTB43	14
COPI-independent Golgi-to-ER retrograde traffic	DCTN4 GIMAP2	14
Regulation of signaling by CBL	CX3CL1 PIK3R1	14
RMTs methylate histone arginines	NR3C2 RASA1	14
SMAD2/SMAD3:SMAD4 heterotrimer regulates transcription	CDK8 HIST2H2BE	14
UDP-N-acetyl-D-galactosamine biosynthesis II | UDP-N-acetyl-D-glucosamine biosynthesis II	ALDH3A1 MLYCD	14
Beta-catenin phosphorylation cascade	NDFIP1	14
Constitutive Signaling by NOTCH1 HD Domain Mutants | NOTCH2 Activation and Transmission of Signal to the Nucleus | NOTCH3 Activation and Transmission of Signal to the Nucleus | Receptor-ligand binding initiates the second proteolytic cleavage of Notch receptor | Signaling by NOTCH4	ZBTB43	14
putrescine degradation III | spermine and sperdegradation I	MLYCD	14
sphingosine and sphingosine-1-phosphate metabolism	CES2	14

Table C (continued)

Pathway	Regulators	N
Ion transport by P-type ATPases	ETV7 HTN1 LINC00051 MCMDC2 PART1 PEBP4 PGK2	13
Stimuli-sensing channels	ETV7 HTN1 MCMDC2 MNS1 PART1 PEBP4	13
Activation of E2F1 target genes at G1/S	CXCL14 DHFR LYL1 SEC62	13
RNA Polymerase I Promoter Escape | RNA Polymerase I Transcription Initiation | RNA Polymerase I Transcription Termination	CCNH DAPK3 SYT11 TNNT2	13
Arf6 trafficking events | Canonical Wnt signaling pathway | IL2-mediated signaling events | Plexin-D1 Signaling | RhoA signaling pathway | T cell activation	PIK3R1 PLCG2	13
Interleukin signaling pathway	DCP2 PIK3R1	13
Activation of RAC1	GRHL2	13
Aurora A signaling	RASA1	13
colanic acid building blocks biosynthesis | GDP-mannose biosynthesis	MAN2A1	13
Disassembly of the destruction complex and recruitment of AXIN to the membrane	DVL3	13
Meiotic synapsis | Packaging Of Telomere Ends	HIST2H2BE NIPAL4 RAB3A SSB ZNF446	12
Neurotransmitter receptors and postsynaptic signal transmission	ETV7 HTN1 LINC00051 MCMDC2 PEBP4	12
Phase 0 - rapid depolarisation | TRP channels	ETV7 HTN1 MCMDC2 PART1 PEBP4	12
Cyclin A/B1/B2 associated events during G2/M transition	CCNH DAPK3 HAMP RPL13A	12
Cyclin A:Cdk2-associated events at S phase entry	CCNH DAPK3 MYC	12
Cyclin D associated events in G1	CCNH DAPK3 RPL13A	12
ALK1 signaling events	PEBP4 TNFSF10	12
Amino acid transport across the plasma membrane	HTN1 IQGAP2	12
Arf1 pathway | LPA4-mediated signaling events	PLCG2 SELPLG	12
B cell activation	ATXN7L2 PLCG2	12
coenzyme A biosynthesis	ALDH3A1 ANP32B	12
EPH-ephrin mediated repulsion of cells | Post-translational regulation of adherens junction stability and dissassembly	PLCG2 RASGRF2	12
ErbB4 signaling events | Neurotrophic factor-mediated Trk receptor signaling | TRAIL signaling pathway	PIK3R1 TNFSF10	12
RNA Polymerase III Abortive And Retractive Initiation | RNA Polymerase III Transcription Initiation From Type 3 Promoter	BDP1 SNAPC2	12
PLK1 signaling events	ECT2	12
TFAP2 (AP-2) family regulates transcription of growth factors and their receptors	NDRG1	12

Table C (continued)

Pathway	Regulators	N
Activation of rRNA Expression by ERCC6 (CSB) and EHMT2 (G9a) | SIRT1 negatively regulates rRNA expression	HIST2H2BE LYL1 NIPAL4 PARP10 RAB3A ZNF446	11
AURKA Activation by TPX2 | Loss of Nlp from mitotic centrosomes | Loss of proteins required for interphase microtubule organizationfrom the centrosome | Recruitment of mitotic centrosome proteins and complexes | Recruitment of NuMA to mitotic centrosomes | Regulation of PLK1 Activity at G2/M Transition	ADH1C ATG10 KRTAP15-1 MRPL54 SOX14	11
Activation of AMPA receptors | Activation of the phototransduction cascade | Glutamate Neurotransmitter Release Cycle | Mitochondrial calcium ion transport | Reduction of cytosolic Ca++ levels | Sodium/Calcium exchangers | Unblocking of NMDA receptor, glutamate binding and activation	ETV7 HTN1 MCMDC2 PEBP4	11
Phase 2 - plateau phase | Sperm Motility And Taxes	ETV7 MCMDC2 PEBP4 PGK2	11

Table C (continued)

Pathway	Regulators	N
Activation of CaMK IV | Activation of Ca-permeable Kainate Receptor | Amyloid fiber formation | Calcineurin activates NFAT | Calmodulin induced events | CaMK IV-mediated phosphorylation of CREB | CaM pathway | Cam-PDE 1 activation | Chromatin modifying enzymes | CREB phosphorylation through the activation of Adenylate Cyclase | CREB phosphorylation through the activation of CaMKII | DARPP-32 events | Defective SLC24A1 causes congenital stationary night blindness 1D (CSNB1D) | Defective SLC24A4 causes hypomineralized amelogenesis imperfecta (AI) | Degradation of the extracellular matrix | Deregulated CDK5 triggers multiple neurodegenerative pathways in Alzheimer's disease models | eNOS activation | Glycerophospholipid biosynthesis | Glycogen breakdown (glycogenolysis) | Glycosphingolipid metabolism | Highly calcium permeable nicotinic acetylcholine receptors | Highly calcium permeable postsynaptic nicotinic acetylcholine receptors | Inactivation, recovery and regulation of the phototransduction cascade | phospho-PLA2 pathway | PKA activation | Presynaptic depolarization and calcium channel opening | Ras activation upon Ca2+ influx through NMDA receptor | RHO GTPases activate PAKs | Smooth Muscle Contraction | Striated Muscle Contraction | Synthesis of Leukotrienes (LT) and Eoxins (EX) | Tetrahydrobiopterin (BH4) synthesis, recycling, salvage and regulation | Translocation of GLUT4 to the plasma membrane | Uptake and function of anthrax toxins	ETV7 MCMDC2 PEBP4	11
COPI-dependent Golgi-to-ER retrograde traffic	ADGRB1 KDELR2 WDR49	11
glycogen degradation II | glycogen degradation III	APOA1BP PCK1 PFKFB1	11
Angiotensin_II-stimulated_signaling_through_G proteins_and_beta-arrestin	ETV7 PLCG2	11
Synthesis of IP2, IP, and Ins in the cytosol	GALE PLCG2	11

Table C (continued)

Pathway	Regulators	N
acetate conversion to acetyl-CoA | Costimulation by the CD28 family | ethanol degradation II | ethanol degradation IV | Hypoxia response via HIF activation | IL2 signaling events mediated by STAT5 | IL3-mediated signaling events | IL6-mediated signaling events | Interleukin-7 signaling | N-cadherin signaling events | oxidative ethanol degradation III | Ras Pathway | RET signaling | Signaling events mediated by Stem cell factor receptor (c-Kit) | Signaling events mediated by TCPTP | Stabilization and expansion of the E-cadherin adherens junction	PIK3R1	11
mTOR signalling	IQGAP2	11
Synthesis of PIPs at the Golgi membrane	VAC14	11
Ubiquitin proteasome pathway	ZCCHC9	11
Senescence-Associated Secretory Phenotype (SASP)	CDX1 FCRL2 HIST2H2BE LYL1 NIPAL4 NQO2 RAB3A SLC25A2 ZNF446	10
Formation of TC-NER Pre-Incision Complex | Transcription-Coupled Nucleotide Excision Repair (TC-NER)	BCAN C14orf1 CCNH DAPK3 LYL1 TNNT2	10
Angiopoietin receptor Tie2-mediated signaling | Cellular roles of Anthrax toxin	HOXD9 TNFSF10	10
CD22 mediated BCR regulation	ARHGEF4 ATXN7L2	10
Cristae formation	ATP5G3 SLC39A13	10
G0 and Early G1	LYL1 SEC62	10
5HT2 type receptor mediated signaling pathway | Axon guidance mediated by netrin | Histamine H1 receptor mediated signaling pathway | IP3 and IP4 transport between cytosol and nucleus | IPs transport between ER lumen and cytosol | Metabotropic glutamate receptor group I pathway | Muscarinic acetylcholine receptor 1 and 3 signaling pathway | Oxytocin receptor mediated signaling pathway | SEMA3A-Plexin repulsion signaling by inhibiting Integrin adhesion | Signal transduction by L1 | Thyrotropin-releasing hormone receptor signaling pathway | untitled	PLCG2	10
a6b1 and a6b4 Integrin signaling	PMP22	10
CTLA4 inhibitory signaling	KLHL24	10
RNA Polymerase III Transcription Initiation From Type 1 Promoter | RNA Polymerase III Transcription Initiation From Type 2 Promoter	BDP1	10
sulfate activation for sulfonation	CHST12	10
Formation of Incision Complex in GG-NER	CCNH DAPK3 KRTAP15-1 TNNT2	9
Formation of the Early Elongation Complex | Formation of the HIV-1 Early Elongation Complex	CCNH DAPK3 NTRK1 TNNT2	9

Table C (continued)

Pathway	Regulators	N
mRNA Capping | RNA Pol II CTD phosphorylation and interaction with CE | RNA Pol II CTD phosphorylation and interaction with CE during HIV infection	CCNH DAPK3 TNNT2	9
Dual Incision in GG-NER	POLK TNNT2	9
Factors involved in megakaryocyte development and platelet production	PDE8B PRKACA	9
HIV elongation arrest and recovery | Pausing and recovery of HIV elongation | Pausing and recovery of Tat-mediated HIV elongation | Tat-mediated HIV elongation arrest and recovery	ELL NTRK1	9
Processing of Capped Intron-Containing Pre-mRNA	FEV YTHDC1	9
Regulation of retinoblastoma protein	CXCL14 MEF2C	9
Activation of NIMA Kinases NEK9, NEK6, NEK7 | Retinoic acid receptors-mediated signaling	CCNH	9
Arf6 downstream pathway | Integrins in angiogenesis | mTOR signaling pathway | S1P1 pathway | S1P2 pathway | S1P3 pathway | S1P4 pathway | Signaling events mediated by PRL | Syndecan-1-mediated signaling events | Syndecan-2-mediated signaling events | Trk receptor signaling mediated by the MAPK pathway | VEGFR3 signaling in lymphatic endothelium	TNFSF10	9
gamma-glutamyl cycle | Glutathione synthesis and recycling	APOA1BP	9
Growth hormone receptor signaling | JAK/STAT signaling pathway	MCMDC2	9
MASTL Facilitates Mitotic Progression	ENSA	9
Pre-NOTCH Transcription and Translation	PRKCI	9
Transcription of E2F targets under negative control by p107 (RBL1) and p130 (RBL2) in complex with HDAC1	NPM1	9
APC/C:Cdc20 mediated degradation of Cyclin B | APC/C:Cdc20 mediated degradation of mitotic proteins | APC-Cdc20 mediated degradation of Nek2A | Conversion from APC/C:Cdc20 to APC/C:Cdh1 in late anaphase | Inactivation of APC/C via direct inhibition of the APC/C complex | Phosphorylation of the APC/C | Regulation of APC/C activators between G1/S and early anaphase	CDX1 FCRL2 NQO2 SLC25A2	8
Respiratory electron transport	ALDH3A1 CAMK2A MRPL54	8
Activation of the pre-replicative complex | Assembly of the pre-replicative complex	CXCL14 S100A14	8
Attenuation phase | NAD biosynthesis III	PARP10	8

Table C (continued)

Pathway	Regulators	N
CMP-N-acetylneuraminate biosynthesis I (eukaryotes)	ST3GAL2	8
FOXM1 transcription factor network	MYC	8
Hypoxic and oxygen homeostasis regulation of HIF-1-alpha	VHL	8
tRNA charging	NQO2	8
Activated PKN1 stimulates transcription of AR (androgen receptor) regulated genes KLK2 and KLK3 | DNA methylation | PRC2 methylates histones and DNA | RNA Polymerase I Promoter Opening	HIST2H2BE LYL1 NIPAL4 RAB3A ZNF446	7
adenosine nucleotides degradation II | purine nucleotides degradation II (aerobic)	KCNK7 PCK1 PFKFB1	7
Complex I biogenesis	ALDH3A1 MRPL54	7
Erythrocytes take up carbon dioxide and release oxygen | Erythrocytes take up oxygen and release carbon dioxide | Reversible hydration of carbon dioxide	FBLN1 MNS1	7
Unwinding of DNA	CXCL14 SOX14	7
calcium transport I | Zinc efflux and compartmentalization by the SLC30 family | Zinc influx into cells by the SLC39 gene family	CACNB3	7
Formation of ATP by chemiosmotic coupling	ATP5G3	7
Initiation of Nuclear Envelope Reformation	RPL13A	7
Regulation of RAC1 activity	ARHGAP1	7
Bicarbonate transporters | Multifunctional anion exchangers	FBLN1 HTN1	6
Processing of Intronless Pre-mRNAs	ICAM3 ZNF446	6
7646062 | 9822713 | P|: 15646034| P|: 9122178 |	GNPDA1	6
Antigen Presentation: Folding, assembly and peptide loading of class I MHC | Methionine salvage pathway | NIK–¿noncanonical NF-kB signaling | The role of GTSE1 in G2/M progression after G2 checkpoint	PSMB10	6
Direct p53 effectors | Regulation of nuclear SMAD2/3 signaling | Switching of origins to a post-replicative state	CXCL14	6
Immunoregulatory interactions between a Lymphoid and a non-Lymphoid cell	ARHGEF4	6
Budding and maturation of HIV virion	ARRDC2 CDX1 RASSF5	5
G alpha (s) signalling events	ETV7 MNS1 NPY4R	5
adenine and adenosine salvage III | gluconeogenesis I | glycolysis I | guanine and guanosine salvage I | guanosine nucleotides degradation III | purine ribonucleosides degradation to ribose-1-phosphate | salvage pathways of pyriribonucleotides | S-methyl-5'-thioadenosine degradation II | urate biosynthesis/inosine 5'-phosphate degradation | xanthine and xanthosine salvage	PCK1 PFKFB1	5

Table C (continued)

Pathway	Regulators	N
Glypican 1 network	HOXD9 PEBP4	5
Vasopressin regulates renal water homeostasis via Aquaporins	MNS1 NPY4R	5
COPII-mediated vesicle transport	MEF2C	5
Ketone body catabolism | Pyruvate metabolism | Synthesis of Ketone Bodies	TSPAN15	5
Nef Mediated CD4 Down-regulation	ATXN7L2	5
Retrograde transport at the Trans-Golgi-Network	RHOBTB3	5
WNT5A-dependent internalization of FZD4	MYO1D	5
Heterotrimeric G-protein signaling pathway-Gi alpha and Gs alpha mediated pathway	EDN3 ETV7 HHLA1 MNS1	4
Endosomal Sorting Complex Required For Transport (ESCRT)	ARRDC2 CDX1	4
Mitochondrial protein import	PAM16 SLC39A13	4
Muscarinic acetylcholine receptor 2 and 4 signaling pathway	EDN3 MNS1	4
2-oxobutanoate degradation I | purine nucleotides de novo biosynthesis II	KCNK7	4
Activation of the mRNA upon binding of the cap-binding complex and eIFs, and subsequent binding to 43S | Deadenylation of mRNA | Energy dependent regulation of mTOR by LKB1-AMPK | ISG15 antiviral mechanism	IDH1	4
APEX1-Independent Resolution of AP Sites via the Single Nucleotide Replacement Pathway	SLC39A13	4
Assembly of the ORC complex at the origin of replication | CDC6 association with the ORC:origin complex | E2F-enabled inhibition of pre-replication complex formation | Glycolysis | PP2A-mediated dephosphorylation of key metabolic factors	S100A14	4
Association of TriC/CCT with target proteins during biosynthesis | Formation of tubulin folding intermediates by CCT/TriC	BCAN	4
Formation of annular gap junctions | G alpha (12/13) signalling events | Gap junction degradation | Sema4D induced cell migration and growth-cone collapse	RASGRF2	4
Negative regulation of MAPK pathway	RPGR	4
TFAP2 (AP-2) family regulates transcription of cell cycle factors	PTPRE	4
TGF-beta receptor signaling	PEBP4	4
Cellular hexose transport	HTN1 PART1	3
Inhibition of voltage gated Ca2+ channels via Gbeta/gamma subunits	MNS1 PGK2	3
Alpha-synuclein signaling	ADGRB1	3
Beta1 integrin cell surface interactions	ZNF488	3

Table C (continued)

Pathway	Regulators	N
Beta3 adrenergic receptor signaling pathway | PI3 kinase pathway	MNS1	3
Oxidative stress response	TMOD4	3
p53 pathway	SPINK4	3
Post-chaperonin tubulin folding pathway	NUP210L	3
TNF receptor superfamily (TNFSF) members mediating non-canonical NF-kB pathway	TNNT2	3
Cation-coupled Chloride cotransporters	HTN1 LINC00051	2
Adrenaline,noradrenaline inhibits insulin secretion | Endogenous_cannabinoid_signaling | GABA-B_receptor_II_signaling | Heterotrimeric G-protein signaling pathway-Gq alpha and Go alpha mediated pathway | Heterotrimeric G-protein signaling pathway-rod outer segment phototransduction | PNAT | Wnt signaling pathway	ETV7	2
Alpha9 beta1 integrin signaling events | p53 pathway by glucose deprivation	HOXD9	2
Binding of TCF/LEF:CTNNB1 to target gene promoters | NOTCH1 Intracellular Domain Regulates Transcription | RUNX3 regulates WNT signaling	HIST2H2BE	2
Cohesin Loading onto Chromatin | Establishment of Sister Chromatid Cohesion	NIPAL4	2
Glucuronidation	SH2D3C	2
IRF3-mediated induction of type I IFN	IFI16	2
Membrane binding and targetting of GAG proteins	RASSF5	2
Validated targets of C-MYC transcriptional activation	GSTP1	2
3-phosphoinositide degradation | arginine biosynthesis IV | citrulline degradation | proline biosynthesis II (from arginine) | urea cycle	PCK1	1
Abortive elongation of HIV-1 transcript in the absence of Tat	NTRK1	1

Table C (continued)

Pathway	Regulators	N
Acetylcholine Neurotransmitter Release Cycle | Activation of Na-permeable kainate receptors | Amine compound SLC transporters | Amino acid and oligopeptide SLC transporters | Bile salt and organic anion SLC transporters | Biotin transport and metabolism | Clearance of dopamine | Clearance of seratonin | Creatine metabolism | Defective SLC24A5 causes oculocutaneous albinism 6 (OCA6) | Defective SLC4A4 causes renal tubular acidosis, proximal, with ocular abnormalities and mental retardation (pRTA-OA) | Defective SLC9A6 causes X-linked, syndromic mental retardation,, Christianson type (MRXSCH) | Defective SLC9A9 causes autism 16 (AUTS16) | Highly sodium permeable acetylcholine nicotinic receptors | Import of palmitoyl-CoA into the mitochondrial matrix | Inositol transporters | Intestinal hexose absorption | Na+/Cl- dependent neurotransmitter transporters | Nicotinasalvaging | Organic anion transporters | Organic cation transport | Recycling of bile acids and salts | Reuptake of GABA | Sodium-coupled phosphate cotransporters | Sodium-coupled sulphate, di- and tri-carboxylate transporters | Sodium/Proton exchangers | Surfactant metabolism | Synthesis of PC | Thyroxine biosynthesis | Transport of bile salts and organic acids, metal ions and amine compounds | Transport of inorganic cations/anions and amino acids/oligopeptides | Transport of nucleosides and free purine and pyribases across the plasma membrane | Transport of vitamins, nucleosides, and related molecules | Type II Na+/Pi cotransporters | Vitamin B5 (pantothenate) metabolism | Vitamin C (ascorbate) metabolism	HTN1	1

Table C (continued)

Pathway	Regulators	N
Activation of G protein gated Potassium channels | ATP sensitive Potassium channels | Ca activated K+ channels | Classical Kir channels | Defective ABCC8 can cause hypoglycemias and hyperglycemias | HCN channels | Phase 1 - inactivation of fast Na+ channels | Phase 3 - rapid repolarisation | Phase 4 - resting membrane potential | Potassium transport channels | Tandem of pore domain in a weak inwardly rectifying K+ channels (TWIK) | Tandem pore domain halothane-inhibited K+ channel (THIK) | The NLRP3 inflammasome | TWIK-related alkaline pH activated K+ channel (TALK) | TWIK related potassium channel (TREK) | TWIK-related spinal cord K+ channel (TRESK) | TWIK-releated acid-sensitive K+ channel (TASK) | Voltage gated Potassium channels	PGK2	1
acyl-CoA hydrolysis | CDP-diacylglycerol biosynthesis I | cerabiosynthesis | heme biosynthesis II | mitochondrial L-carnitine shuttle pathway | oleate biosynthesis II (animals) | phosphatidylglycerol biosynthesis II (non-plastidic) | tetrapyrrole biosynthesis II | triacylglycerol biosynthesis	ALDH3A1	1
ATF4 activates genes | Butyrate Response Factor 1 (BRF1) binds and destabilizes mRNA | KSRP (KHSRP) binds and destabilizes mRNA | mRNA decay by 3' to 5' exoribonuclease | Tristetraprolin (TTP, ZFP36) binds and destabilizes mRNA	FAM212B	1
Olfactory Signaling Pathway	LINC00051	1
Retinoid cycle disease events | The canonical retinoid cycle in rods (twilight vision)	LST1	1

Table D. Regulators, their number of targets, number of datasets with self aCGH/expression correlation and main pathway. Targs = Number of significant targets for which regulator is the best regulator p-value < 0.05, D'sets =Number of datsets in which the regulator has significant correlation between its aCGH profile and its own expression profile

Regulator	Targs	D'sets	Main pathway
AZIN1	2	22	Regulation of ornithine decarboxylase (ODC)
AGO2	7	21	Transcriptional regulation by small RNAs
DERL1	3	21	E3 ubiquitin ligases ubiquitinate target proteins
PTK2	3	21	PMID: 9187108;9256433
BCL9	1	21	Formation of the beta-catenin:TCF transactivating complex
POGZ	33	20	RNA polymerase II transcribes snRNA genes
MRPS28	4	19	Mitochondrial translation termination
YWHAZ	4	19	ATR signaling pathway
CHD1	2	18	Estrogen-dependent gene expression
ZC3H3	1	18	Cleavage of Growing Transcript in the Termination Region;Transport of Mature mRNA derived from an Intron-Containing Transcript;mRNA 3'-end processing
HSBP1	5	17	CXCR4-mediated signaling events;IL12 signaling mediated by STAT4;IL12-mediated signaling events;TCR signaling in nave CD4+ T cells
ANKRD46	4	17	RUNX1 regulates genes involved in megakaryocyte differentiation and platelet function
RABGAP1	1	17	Regulation of gene expression in beta cells
TRAK1	1	17	Signaling by BRAF and RAF fusions
TERF2IP	8	16	Acetylcholine regulates insulin secretion;Activation of NF-kappaB in B cells;Activation of RAS in B cells;Antigen activates B Cell Receptor (BCR) leading to generation of second messengers;Arachidonate production from DAG;Ca2+ pathway;EGFR Transactivation by Gastrin;Effects of PIP2 hydrolysis;Elevation of cytosolic Ca2+ levels;Fatty Acids bound to GPR40 (FFAR1) regulate insulin secretion;G alpha (q) signalling events;G beta:gamma signalling through PLC beta;GPVI-mediated activation cascade;Rap1 signalling;Response to elevated platelet cytosolic Ca2+;Syndecan interactions;Synthesis of IP3 and IP4 in the cytosol
RBBP5	2	16	Activation of anterior HOX genes in hindbrain development during early embryogenesis;RUNX1 regulates genes involved in megakaryocyte differentiation and platelet function
ATG7	1	16	Antigen processing: Ubiquitination and Proteasome degradation;Interconversion of nucleotide di- and triphosphates
NCOA6	1	16	Activation of anterior HOX genes in hindbrain development during early embryogenesis

Table D (continued)

Regulator	Targs	D'sets	Main pathway
SLC30A5	1	16	NEP/NS2 Interacts with the Cellular Export Machinery;NS1 Mediated Effects on Host Pathways;Nuclear Pore Complex (NPC) Disassembly;Nuclear import of Rev protein;Regulation of Glucokinase by Glucokinase Regulatory Protein;Regulation of HSF1-mediated heat shock response;Rev-mediated nuclear export of HIV RNA;SUMOylation of DNA damage response and repair proteins;SUMOylation of DNA replication proteins;SUMOylation of RNA binding proteins;SUMOylation of chromatin organization proteins;Transcriptional regulation by small RNAs;Transport of Mature mRNA Derived from an Intronless Transcript;Transport of Mature mRNA derived from an Intron-Containing Transcript;Transport of Ribonucleoproteins into the Host Nucleus;Transport of the SLBP Dependant Mature mRNA;Transport of the SLBP independent Mature mRNA;Viral Messenger RNA Synthesis;Vpr-mediated nuclear import of PICs;snRNP Assembly;tRNA processing in the nucleus
VCPIP1	1	16	Ovarian tumor domain proteases
WWOX	1	16	Formation of the beta-catenin:TCF transactivating complex
PTS	6	15	tetrahydrobiopterin biosynthesis I;tetrahydrobiopterin biosynthesis II
DOCK1	1	15	Integrin signalling pathway
INTS8	9	14	Acetylcholine regulates insulin secretion;Activation of NF-kappaB in B cells;Activation of RAS in B cells;Antigen activates B Cell Receptor (BCR) leading to generation of second messengers;Arachidonate production from DAG;Ca2+ pathway;EGFR Transactivation by Gastrin;Effects of PIP2 hydrolysis;Elevation of cytosolic Ca2+ levels;Fatty Acids bound to GPR40 (FFAR1) regulate insulin secretion;G alpha (q) signalling events;G beta:gamma signalling through PLC beta;GPVI-mediated activation cascade;Rap1 signalling;Response to elevated platelet cytosolic Ca2+;Syndecan interactions;Synthesis of IP3 and IP4 in the cytosol
ZBTB43	4	14	Constitutive Signaling by NOTCH1 HD Domain Mutants
CES2	3	14	E2F transcription factor network
MLYCD	3	14	UDP-N-acetyl-D-galactosamine biosynthesis II;UDP-N-acetyl-D-glucosamine biosynthesis II
CDK8	2	14	SMAD2/SMAD3:SMAD4 heterotrimer regulates transcription
NDFIP1	2	14	Beta-catenin phosphorylation cascade
DCTN4	1	14	COPI-mediated anterograde transport
POP1	1	14	tRNA processing in the nucleus
RNF139	6	13	Gap-filling DNA repair synthesis and ligation in TC-NER;HDR through MMEJ (alt-NHEJ);Processing of DNA double-strand break ends
WDR41	4	13	Condensation of Prophase Chromosomes
DVL3	1	13	Degradation of DVL
GRHL2	1	13	Activation of RAC1
MAN2A1	1	13	GDP-mannose biosynthesis;colanic acid building blocks biosynthesis
NSMCE2	1	13	SUMOylation of DNA damage response and repair proteins

Table D (continued)

Regulator	Targs	D'sets	Main pathway
RASA1	1	13	RMTs methylate histone arginines
TBL1XR1	1	13	HDACs deacetylate histones
TCERG1	1	13	pre-mRNA splicing
NDRG1	8	12	TFAP2 (AP-2) family regulates transcription of growth factors and their receptors
PIP5K1A	6	12	Acetylcholine regulates insulin secretion;Activated NTRK2 signals through PI3K;Antigen activates B Cell Receptor (BCR) leading to generation of second messengers;CD28 dependent PI3K/Akt signaling;CLEC7A (Dectin-1) signaling;Ca2+ pathway;Cargo recognition for clathrin-mediated endocytosis;Clathrin-mediated endocytosis;Constitutive Signaling by AKT1 E17K in Cancer;Constitutive Signaling by Aberrant PI3K in Cancer;Constitutive Signaling by EGFRvIII;Constitutive Signaling by Ligand-Responsive EGFR Cancer Variants;DAG and IP3 signaling;DAP12 signaling;DCC mediated attractive signaling;Downstream TCR signaling;Downstream signal transduction;EGFR downregulation;EPHB-mediated forward signaling;ER-Phagosome pathway;FCERI mediated Ca+2 mobilization;Fatty Acids bound to GPR40 (FFAR1) regulate insulin secretion;G alpha (q) signalling events;G beta:gamma signalling through PI3Kgamma;G beta:gamma signalling through PLC beta;GAB1 signalosome;GPVI-mediated activation cascade;GRB2:SOS provides linkage to MAPK signaling for Integrins;Generation of second messenger molecules;IRAK4 deficiency (TLR2/4);Integrin alphaIIb beta3 signaling;MET activates PI3K/AKT signaling;MyD88 deficiency (TLR2/4);MyD88:Mal cascade initiated on plasma membrane;Negative regulation of the PI3K/AKT network;Netrin-1 signaling;PI Metabolism;PI-3K cascade:FGFR1;PI-3K cascade:FGFR2;PI-3K cascade:FGFR3;PI-3K cascade:FGFR4;PI3K Cascade;PI3K events in ERBB2 signaling;PI3K events in ERBB4 signaling;PI3K/AKT Signaling;PI3K/AKT activation;PI5P, PP2A and IER3 Regulate PI3K/AKT Signaling;PLC beta mediated events;RHO GTPases Activate WASPs and WAVEs;RIPK1-mediated regulated necrosis;Recycling pathway of L1;Regulation of actin dynamics for phagocytic cup formation;Role of LAT2/NTAL/LAB on calcium mobilization;Role of phospholipids in phagocytosis;Role of second messengers in netrin-1 signaling;Signaling by FGFR1 in disease;Signaling by FGFR2 in disease;Signaling by FGFR3 fusions in cancer;Signaling by FGFR3 point mutants in cancer;Signaling by FGFR4 in disease;Signaling by SCF-KIT;Signaling by cytosolic FGFR1 fusion mutants;Syndecan interactions;Synthesis of IP3 and IP4 in the cytosol;Synthesis of PIPs at the plasma membrane;VEGFA-VEGFR2 Pathway;VEGFR2 mediated cell proliferation;WNT mediated activation of DVL;p130Cas linkage to MAPK signaling for integrins

Table D (continued)

Regulator	Targs	D'sets	Main pathway
USP13	4	12	Amplification of signal from unattached kinetochores via a MAD2 inhibitory signal;Mitotic Prometaphase;RHO GTPases Activate Formins;Resolution of Sister Chromatid Cohesion;Separation of Sister Chromatids
PHC3	3	12	Oxidative Stress Induced Senescence
ANP32B	2	12	coenzyme A biosynthesis
ASH1L	2	12	PKMTs methylate histone lysines
ECT2	1	12	PLK1 signaling events
ZCCHC9	26	11	Antigen processing: Ubiquitination and Proteasome degradation
IQGAP2	9	11	Estrogen-dependent gene expression
POLD3	7	11	TNFR2 non-canonical NF-kB pathway
PIK3R1	6	11	Activated NTRK2 signals through PI3K;Activation of PKB;Antigen activates B Cell Receptor (BCR) leading to generation of second messengers;CD28 dependent PI3K/Akt signaling;CD28 dependent Vav1 pathway;Constitutive Signaling by Aberrant PI3K in Cancer;Constitutive Signaling by EGFRvIII;Constitutive Signaling by Ligand-Responsive EGFR Cancer Variants;DAP12 signaling;Downstream TCR signaling;Downstream signal transduction;FCERI mediated Ca+2 mobilization;FCERI mediated MAPK activation;G beta:gamma signalling through PI3Kgamma;GAB1 signalosome;GPVI-mediated activation cascade;Inhibition of TSC complex formation by PKB;MET activates PI3K/AKT signaling;Negative regulation of the PI3K/AKT network;PDE3B signalling;PI Metabolism;PI-3K cascade:FGFR1;PI-3K cascade:FGFR2;PI-3K cascade:FGFR3;PI-3K cascade:FGFR4;PI3K Cascade;PI3K events in ERBB2 signaling;PI3K events in ERBB4 signaling;PI3K/AKT Signaling;PI3K/AKT activation;PI5P, PP2A and IER3 Regulate PI3K/AKT Signaling;PTEN Loss of Function in Cancer;Phospholipase C-mediated cascade: FGFR1;Phospholipase C-mediated cascade; FGFR2;Phospholipase C-mediated cascade; FGFR3;Phospholipase C-mediated cascade; FGFR4;RHO GTPases Activate WASPs and WAVEs;RHO GTPases activate PKNs;RIPK1-mediated regulated necrosis;Regulation of TP53 Degradation;Regulation of actin dynamics for phagocytic cup formation;Role of LAT2/NTAL/LAB on calcium mobilization;Role of phospholipids in phagocytosis;Signaling by FGFR1 in disease;Signaling by FGFR2 in disease;Signaling by FGFR3 fusions in cancer;Signaling by FGFR3 point mutants in cancer;Signaling by FGFR4 in disease;Signaling by SCF-KIT;Signaling by cytosolic FGFR1 fusion mutants;Synthesis of PIPs at the plasma membrane;VEGFA-VEGFR2 Pathway;VEGFR2 mediated vascular permeability
DCP2	3	11	Inflammation mediated by chemokine and cytokine signaling pathway;Interleukin signaling pathway;PDGF signaling pathway;VEGF signaling pathway
VAC14	2	11	Synthesis of PIPs at the Golgi membrane
KDELR2	1	11	COPI-dependent Golgi-to-ER retrograde traffic

Table D (continued)

Regulator	Targs	D'sets	Main pathway
PAM	1	11	Gap-filling DNA repair synthesis and ligation in TC-NER;HDR through MMEJ (alt-NHEJ);Processing of DNA double-strand break ends
PLCG2	10	10	FCERI mediated Ca+2 mobilization
KLHL24	9	10	CTLA4 inhibitory signaling
VPS8	6	10	Formation of a pool of free 40S subunits;Formation of the ternary complex, and subsequently, the 43S complex;GTP hydrolysis and joining of the 60S ribosomal subunit;L13a-mediated translational silencing of Ceruloplasmin expression;Ribosomal scanning and start codon recognition;Translation initiation complex formation
CERK	3	10	Anchoring of the basal body to the plasma membrane
BDP1	2	10	RNA Polymerase III Abortive And Retractive Initiation;RNA Polymerase III Transcription Initiation From Type 3 Promoter
PMP22	2	10	FBXL7 down-regulates AURKA during mitotic entry and in early mitosis
CHST12	1	10	sulfate activation for sulfonation

Table D (continued)

Regulator	Targs	D'sets	Main pathway
MCMDC2	10	9	Activation of AMPA receptors;Activation of Ca-permeable Kainate Receptor;Activation of CaMK IV;Activation of NF-kappaB in B cells;Activation of the phototransduction cascade;Amyloid fiber formation;Antigen activates B Cell Receptor (BCR) leading to generation of second messengers;CLEC7A (Dectin-1) induces NFAT activation;CREB phosphorylation through the activation of Adenylate Cyclase;CREB phosphorylation through the activation of CaMKII;Ca2+ pathway;CaM pathway;CaMK IV-mediated phosphorylation of CREB;Calcineurin activates NFAT;Calmodulin induced events;Cam-PDE 1 activation;Chromatin modifying enzymes;DAG and IP3 signaling;DARPP-32 events;Defective SLC24A1 causes congenital stationary night blindness 1D (CSNB1D);Defective SLC24A4 causes hypomineralized amelogenesis imperfecta (AI);Degradation of the extracellular matrix;Deregulated CDK5 triggers multiple neurodegenerative pathways in Alzheimer's disease models;Effects of PIP2 hydrolysis;Elevation of cytosolic Ca2+ levels;FCERI mediated Ca+2 mobilization;FCERI mediated NF-kB activation;Glutamate Neurotransmitter Release Cycle;Glycerophospholipid biosynthesis;Glycogen breakdown (glycogenolysis);Glycosphingolipid metabolism;Highly calcium permeable nicotinic acetylcholine receptors;Highly calcium permeable postsynaptic nicotinic acetylcholine receptors;Inactivation, recovery and regulation of the phototransduction cascade;Ion homeostasis;Ion transport by P-type ATPases;Mitochondrial calcium ion transport;Neurotransmitter receptors and postsynaptic signal transmission;PKA activation;PLC beta mediated events;Phase 0 - rapid depolarisation;Phase 2 - plateau phase;Presynaptic depolarization and calcium channel opening;RHO GTPases activate PAKs;Rap1 signalling;Ras activation upon Ca2+ influx through NMDA receptor;Reduction of cytosolic Ca++ levels;Regulation of insulin secretion;Response to elevated platelet cytosolic Ca2+;Role of phospholipids in phagocytosis;Smooth Muscle Contraction;Sodium/Calcium exchangers;Sperm Motility And Taxes;Stimuli-sensing channels;Striated Muscle Contraction;Synthesis of Leukotrienes (LT) and Eoxins (EX);TRP channels;Tetrahydrobiopterin (BH4) synthesis, recycling, salvage and regulation;Translocation of GLUT4 to the plasma membrane;Unblocking of NMDA receptor, glutamate binding and activation;Uptake and function of anthrax toxins;VEGFR2 mediated cell proliferation;VEGFR2 mediated vascular permeability;eNOS activation;phospho-PLA2 pathway
UBE2T	6	9	Fanconi Anemia Pathway
CPEB4	5	9	Formation of the beta-catenin:TCF transactivating complex
RPS4X	4	9	Nonsense Mediated Decay (NMD) enhanced by the Exon Junction Complex (EJC)
ENSA	3	9	MASTL Facilitates Mitotic Progression
APOA1BP	2	9	gamma-glutamyl cycle

Table D (continued)

Regulator	Targs	D'sets	Main pathway
SEC24D	2	9	Major pathway of rRNA processing in the nucleolus and cytosol;rRNA modification in the nucleus and cytosol
CCNH	1	9	RUNX1 regulates transcription of genes involved in differentiation of HSCs
ELL2	1	9	RNA polymerase II transcribes snRNA genes
ELL	1	9	RNA polymerase II transcribes snRNA genes
GAB2	1	9	Arf6 signaling events;BCR signaling pathway;CXCR3-mediated signaling events;CXCR4-mediated signaling events;Class I PI3K signaling events;Class I PI3K signaling events mediated by Akt;E-cadherin signaling in the nascent adherens junction;EPHA2 forward signaling;ErbB1 downstream signaling;ErbB2/ErbB3 signaling events;FAS (CD95) signaling pathway;FGF signaling pathway;GMCSF-mediated signaling events;IFN-gamma pathway;IL2 signaling events mediated by PI3K;IL4-mediated signaling events;IL8- and CXCR1-mediated signaling events;IL8- and CXCR2-mediated signaling events;Insulin Pathway;Nephrin/Neph1 signaling in the kidney podocyte;Osteopontin-mediated events;PDGFR-beta signaling pathway;Plasma membrane estrogen receptor signaling;RAC1 signaling pathway;Regulation of Ras family activation;Signaling events mediated by Hepatocyte Growth Factor Receptor (c-Met);Signaling events mediated by VEGFR1 and VEGFR2;TCR signaling in nave CD4+ T cells;TCR signaling in nave CD8+ T cells;Trk receptor signaling mediated by PI3K and PLC-gamma;VEGFR1 specific signals
KMT2E	1	9	RUNX1 regulates genes involved in megakaryocyte differentiation and platelet function
NPM1	1	9	TFAP2A acts as a transcriptional repressor during retinoic acid induced cell differentiation;Transcription of E2F targets under negative control by p107 (RBL1) and p130 (RBL2) in complex with HDAC1
PRKCI	1	9	Pre-NOTCH Transcription and Translation

Regulator	Targs	D'sets	Main pathway
TNFSF10	1	9	ALK1 signaling events;Angiopoietin receptor Tie2-mediated signaling;Arf6 downstream pathway;CDC42 signaling events;CXCR3-mediated signaling events;Cellular roles of Anthrax toxin;EPHB forward signaling;Endothelins;ErbB1 downstream signaling;ErbB4 signaling events;FGF signaling pathway;GMCSF-mediated signaling events;IFN-gamma pathway;Integrins in angiogenesis;Netrin-mediated signaling events;Neurotrophic factor-mediated Trk receptor signaling;Nongenotropic Androgen signaling;Osteopontin-mediated events;PDGFR-beta signaling pathway;Regulation of Telomerase;S1P1 pathway;S1P2 pathway;S1P3 pathway;S1P4 pathway;Signaling events mediated by PRL;Signaling events mediated by VEGFR1 and VEGFR2;Signaling events regulated by Ret tyrosine kinase;Syndecan-1-mediated signaling events;Syndecan-2-mediated signaling events;TRAIL signaling pathway;Trk receptor signaling mediated by the MAPK pathway;VEGFR1 specific signals;VEGFR3 signaling in lymphatic endothelium;mTOR signaling pathway
PARP10	21	8	Activation of rRNA Expression by ERCC6 (CSB) and EHMT2 (G9a);HDACs deacetylate histones;SIRT1 negatively regulates rRNA expression
SSB	10	8	SRP-dependent cotranslational protein targeting to membrane
CX3CL1	9	8	Integrin signalling pathway
NQO2	7	8	APC-Cdc20 mediated degradation of Nek2A;APC/C:Cdc20 mediated degradation of Cyclin B;APC/C:Cdc20 mediated degradation of Securin;APC/C:Cdc20 mediated degradation of mitotic proteins;APC/C:Cdh1 mediated degradation of Cdc20 and other APC/C:Cdh1 targeted proteins in late mitosis/early G1;Antigen processing: Ubiquitination and Proteasome degradation;Autodegradation of Cdh1 by Cdh1:APC/C;CDK-mediated phosphorylation and removal of Cdc6;Cdc20:Phospho-APC/C mediated degradation of Cyclin A;Conversion from APC/C:Cdc20 to APC/C:Cdh1 in late anaphase;Inactivation of APC/C via direct inhibition of the APC/C complex;Phosphorylation of the APC/C;Regulation of APC/C activators between G1/S and early anaphase;Senescence-Associated Secretory Phenotype (SASP);Separation of Sister Chromatids

Table D (continued)

Regulator	Targs	D'sets	Main pathway
IGSF9	6	8	Activated NTRK2 signals through PI3K;Activation of PKB;Antigen activates B Cell Receptor (BCR) leading to generation of second messengers;CD28 dependent PI3K/Akt signaling;CD28 dependent Vav1 pathway;Constitutive Signaling by Aberrant PI3K in Cancer;Constitutive Signaling by EGFRvIII;Constitutive Signaling by Ligand-Responsive EGFR Cancer Variants;DAP12 signaling;Downstream TCR signaling;Downstream signal transduction;FCERI mediated Ca+2 mobilization;FCERI mediated MAPK activation;G beta:gamma signalling through PI3Kgamma;GAB1 signalosome;GPVI-mediated activation cascade;Inhibition of TSC complex formation by PKB;MET activates PI3K/AKT signaling;Negative regulation of the PI3K/AKT network;PDE3B signalling;PI Metabolism;PI-3K cascade:FGFR1;PI-3K cascade:FGFR2;PI-3K cascade:FGFR3;PI-3K cascade:FGFR4;PI3K Cascade;PI3K events in ERBB2 signaling;PI3K events in ERBB4 signaling;PI3K/AKT Signaling;PI3K/AKT activation;PI5P, PP2A and IER3 Regulate PI3K/AKT Signaling;PTEN Loss of Function in Cancer;Phospholipase C-mediated cascade: FGFR1;Phospholipase C-mediated cascade; FGFR2;Phospholipase C-mediated cascade; FGFR3;Phospholipase C-mediated cascade; FGFR4;RHO GTPases Activate WASPs and WAVEs;RHO GTPases activate PKNs;RIPK1-mediated regulated necrosis;Regulation of TP53 Degradation;Regulation of actin dynamics for phagocytic cup formation;Role of LAT2/NTAL/LAB on calcium mobilization;Role of phospholipids in phagocytosis;Signaling by FGFR1 in disease;Signaling by FGFR2 in disease;Signaling by FGFR3 fusions in cancer;Signaling by FGFR3 point mutants in cancer;Signaling by FGFR4 in disease;Signaling by SCF-KIT;Signaling by cytosolic FGFR1 fusion mutants;Synthesis of PIPs at the plasma membrane;VEGFA-VEGFR2 Pathway;VEGFR2 mediated vascular permeability
VHL	4	8	Activation of anterior HOX genes in hindbrain development during early embryogenesis
MYC	3	8	Formation of the beta-catenin:TCF transactivating complex
XRCC4	3	8	Nonhomologous End-Joining (NHEJ)
ST3GAL2	2	8	CMP-N-acetylneuraminate biosynthesis I (eukaryotes)
KLHL3	1	8	Neddylation
NIP7	1	8	Major pathway of rRNA processing in the nucleolus and cytosol
SAP30L	1	8	NoRC negatively regulates rRNA expression
SEC62	1	8	Activation of E2F1 target genes at G1/S;Cyclin E associated events during G1/S transition;G0 and Early G1;Polo-like kinase mediated events;Transcription of E2F targets under negative control by DREAM complex

Table D (continued)

Regulator	Targs	D'sets	Main pathway
SLC25A37	1	8	HIV Transcription Initiation;RNA Polymerase II HIV Promoter Escape;RNA Polymerase II Pre-transcription Events;RNA Polymerase II Promoter Escape;RNA Polymerase II Transcription Initiation;RNA Polymerase II Transcription Initiation And Promoter Clearance;RNA Polymerase II Transcription Pre-Initiation And Promoter Opening;Transcription of the HIV genome
RPL13A	70	7	SRP-dependent cotranslational protein targeting to membrane
MRPL54	15	7	Antigen processing: Ubiquitination and Proteasome degradation
ASNSD1	10	7	pre-mRNA splicing
COTL1	10	7	Signaling events regulated by Ret tyrosine kinase
YTHDC1	7	7	pre-mRNA splicing
ATG10	6	7	AURKA Activation by TPX2;Anchoring of the basal body to the plasma membrane;Loss of Nlp from mitotic centrosomes;Loss of proteins required for interphase microtubule organization-from the centrosome;Recruitment of NuMA to mitotic centrosomes;Recruitment of mitotic centrosome proteins and complexes;Regulation of PLK1 Activity at G2/M Transition
PURA	5	7	pre-mRNA splicing
CACNB3	4	7	Regulation of insulin secretion;Zinc efflux and compartmentalization by the SLC30 family;Zinc influx into cells by the SLC39 gene family
ATP5G3	3	7	Cristae formation;Formation of ATP by chemiosmotic coupling
GEMIN8	3	7	snRNP Assembly
KCNMB3	3	7	Amplification of signal from unattached kinetochores via a MAD2 inhibitory signal;Mitotic Prometaphase;RHO GTPases Activate Formins;Resolution of Sister Chromatid Cohesion;Separation of Sister Chromatids
POLK	3	7	HDR through Homologous Recombination (HRR)
DHX16	2	7	Mitochondrial translation elongation

Table D (continued)

Regulator	Targs	D'sets	Main pathway
PBXIP1	2	7	Activated NTRK2 signals through PI3K;Activation of PKB;Antigen activates B Cell Receptor (BCR) leading to generation of second messengers;CD28 dependent PI3K/Akt signaling;CD28 dependent Vav1 pathway;Constitutive Signaling by Aberrant PI3K in Cancer;Constitutive Signaling by EGFRvIII;Constitutive Signaling by Ligand-Responsive EGFR Cancer Variants;DAP12 signaling;Downstream TCR signaling;Downstream signal transduction;FCERI mediated Ca+2 mobilization;FCERI mediated MAPK activation;G beta:gamma signalling through PI3Kgamma;GAB1 signalosome;GPVI-mediated activation cascade;Inhibition of TSC complex formation by PKB;MET activates PI3K/AKT signaling;Negative regulation of the PI3K/AKT network;PDE3B signalling;PI Metabolism;PI-3K cascade:FGFR1;PI-3K cascade:FGFR2;PI-3K cascade:FGFR3;PI-3K cascade:FGFR4;PI3K Cascade;PI3K events in ERBB2 signaling;PI3K events in ERBB4 signaling;PI3K/AKT Signaling;PI3K/AKT activation;PI5P, PP2A and IER3 Regulate PI3K/AKT Signaling;PTEN Loss of Function in Cancer;Phospholipase C-mediated cascade: FGFR1;Phospholipase C-mediated cascade; FGFR2;Phospholipase C-mediated cascade; FGFR3;Phospholipase C-mediated cascade; FGFR4;RHO GTPases Activate WASPs and WAVEs;RHO GTPases activate PKNs;RIPK1-mediated regulated necrosis;Regulation of TP53 Degradation;Regulation of actin dynamics for phagocytic cup formation;Role of LAT2/NTAL/LAB on calcium mobilization;Role of phospholipids in phagocytosis;Signaling by FGFR1 in disease;Signaling by FGFR2 in disease;Signaling by FGFR3 fusions in cancer;Signaling by FGFR3 point mutants in cancer;Signaling by FGFR4 in disease;Signaling by SCF-KIT;Signaling by cytosolic FGFR1 fusion mutants;Synthesis of PIPs at the plasma membrane;VEGFA-VEGFR2 Pathway;VEGFR2 mediated vascular permeability
PIEZO1	2	7	Estrogen-dependent gene expression
PYGO2	2	7	Formation of the beta-catenin:TCF transactivating complex
ARHGAP1	1	7	Regulation of RAC1 activity
AURKC	1	7	Amplification of signal from unattached kinetochores via a MAD2 inhibitory signal;Mitotic Prometaphase;RHO GTPases Activate Formins;Resolution of Sister Chromatid Cohesion;Separation of Sister Chromatids
LRSAM1	1	7	Antigen processing: Ubiquitination and Proteasome degradation
LSM11	1	7	Amplification of signal from unattached kinetochores via a MAD2 inhibitory signal;Mitotic Prometaphase;RHO GTPases Activate Formins;Resolution of Sister Chromatid Cohesion;Separation of Sister Chromatids
SF3B4	1	7	pre-mRNA splicing
CXCL14	17	6	E2F transcription factor network
IL6R	7	6	Gap-filling DNA repair synthesis and ligation in TC-NER;Processing of DNA double-strand break ends

Table D (continued)

Regulator	Targs	D'sets	Main pathway
ARHGEF4	6	6	Antigen activates B Cell Receptor (BCR) leading to generation of second messengers;CD22 mediated BCR regulation
PDE8B	5	6	Anchoring of the basal body to the plasma membrane
C14orf1	4	6	Dual incision in TC-NER;Formation of TC-NER Pre-Incision Complex;Gap-filling DNA repair synthesis and ligation in TC-NER;Transcription-Coupled Nucleotide Excision Repair (TC-NER)
GNPDA1	3	6	PMID: 9122178;9822713
NR3C2	2	6	NEP/NS2 Interacts with the Cellular Export Machinery;NS1 Mediated Effects on Host Pathways;Nuclear Pore Complex (NPC) Disassembly;Nuclear import of Rev protein;Regulation of Glucokinase by Glucokinase Regulatory Protein;Regulation of HSF1-mediated heat shock response;Rev-mediated nuclear export of HIV RNA;SUMOylation of DNA damage response and repair proteins;SUMOylation of DNA replication proteins;SUMOylation of RNA binding proteins;SUMOylation of chromatin organization proteins;Transcriptional regulation by small RNAs;Transport of Mature mRNA Derived from an Intronless Transcript;Transport of Mature mRNA derived from an Intron-Containing Transcript;Transport of Ribonucleoproteins into the Host Nucleus;Transport of the SLBP Dependant Mature mRNA;Transport of the SLBP independent Mature mRNA;Viral Messenger RNA Synthesis;Vpr-mediated nuclear import of PICs;snRNP Assembly;tRNA processing in the nucleus
PRKACA	2	6	Eukaryotic Translation Termination;Formation of a pool of free 40S subunits;GTP hydrolysis and joining of the 60S ribosomal subunit;Nonsense Mediated Decay (NMD) enhanced by the Exon Junction Complex (EJC);Nonsense Mediated Decay (NMD) independent of the Exon Junction Complex (EJC);Peptide chain elongation;Regulation of expression of SLITs and ROBOs;SRP-dependent cotranslational protein targeting to membrane;Selenocysteine synthesis;Viral mRNA Translation
DHFR	1	6	Activation of E2F1 target genes at G1/S
PSMB10	1	6	Cdc20:Phospho-APC/C mediated degradation of Cyclin A
MYO1D	23	5	Termination of translesion DNA synthesis
ATXN7L2	11	5	Antigen activates B Cell Receptor (BCR) leading to generation of second messengers;CD22 mediated BCR regulation
FIGF	9	5	Mitochondrial translation elongation;Mitochondrial translation initiation;Mitochondrial translation termination

Table D (continued)

Regulator	Targs	D'sets	Main pathway
MEF2C	9	5	Activated NTRK2 signals through PI3K;Activation of PKB;Antigen activates B Cell Receptor (BCR) leading to generation of second messengers;CD28 dependent PI3K/Akt signaling;CD28 dependent Vav1 pathway;Constitutive Signaling by Aberrant PI3K in Cancer;Constitutive Signaling by EGFRvIII;Constitutive Signaling by Ligand-Responsive EGFR Cancer Variants;DAP12 signaling;Downstream TCR signaling;Downstream signal transduction;FCERI mediated Ca+2 mobilization;FCERI mediated MAPK activation;G beta:gamma signalling through PI3Kgamma;GAB1 signalosome;GPVI-mediated activation cascade;Inhibition of TSC complex formation by PKB;MET activates PI3K/AKT signaling;Negative regulation of the PI3K/AKT network;PDE3B signalling;PI Metabolism;PI-3K cascade:FGFR1;PI-3K cascade:FGFR2;PI-3K cascade:FGFR3;PI-3K cascade:FGFR4;PI3K Cascade;PI3K events in ERBB2 signaling;PI3K events in ERBB4 signaling;PI3K/AKT Signaling;PI3K/AKT activation;PI5P, PP2A and IER3 Regulate PI3K/AKT Signaling;PTEN Loss of Function in Cancer;Phospholipase C-mediated cascade: FGFR1;Phospholipase C-mediated cascade; FGFR2;Phospholipase C-mediated cascade; FGFR3;Phospholipase C-mediated cascade; FGFR4;RHO GTPases Activate WASPs and WAVEs;RHO GTPases activate PKNs;RIPK1-mediated regulated necrosis;Regulation of TP53 Degradation;Regulation of actin dynamics for phagocytic cup formation;Role of LAT2/NTAL/LAB on calcium mobilization;Role of phospholipids in phagocytosis;Signaling by FGFR1 in disease;Signaling by FGFR2 in disease;Signaling by FGFR3 fusions in cancer;Signaling by FGFR3 point mutants in cancer;Signaling by FGFR4 in disease;Signaling by SCF-KIT;Signaling by cytosolic FGFR1 fusion mutants;Synthesis of PIPs at the plasma membrane;VEGFA-VEGFR2 Pathway;VEGFR2 mediated vascular permeability
RHOBTB3	8	5	Retrograde transport at the Trans-Golgi-Network
SYT11	8	5	B-WICH complex positively regulates rRNA expression
MCTP1	5	5	Dual incision in TC-NER;Gap-filling DNA repair synthesis and ligation in TC-NER
GEMIN7	3	5	snRNP Assembly
RAD51D	3	5	HDR through Homologous Recombination (HRR)
FBLN1	2	5	HIV Transcription Initiation;RNA Polymerase II HIV Promoter Escape;RNA Polymerase II Pre-transcription Events;RNA Polymerase II Promoter Escape;RNA Polymerase II Transcription Initiation;RNA Polymerase II Transcription Initiation And Promoter Clearance;RNA Polymerase II Transcription Pre-Initiation And Promoter Opening;Transcription of the HIV genome
TBCK	2	5	Amplification of signal from unattached kinetochores via a MAD2 inhibitory signal;Mitotic Prometaphase;RHO GTPases Activate Formins;Resolution of Sister Chromatid Cohesion;Separation of Sister Chromatids

Table D (continued)

Regulator	Targs	D'sets	Main pathway
TSPAN15	2	5	glutaryl-CoA degradation;ketogenesis;ketolysis;mevalonate pathway I;superpathway of cholesterol biosynthesis;superpathway of geranylgeranyldiphosphate biosynthesis I (via mevalonate);tryptophan degradation III (eukaryotic)
ISL1	1	5	Regulation of expression of SLITs and ROBOs
PROCR	131	4	pre-mRNA splicing
BCAN	33	4	Neddylation
IDH1	30	4	GTP hydrolysis and joining of the 60S ribosomal subunit;L13a-mediated translational silencing of Ceruloplasmin expression;Ribosomal scanning and start codon recognition;Translation initiation complex formation
RASGRF2	27	4	Clathrin-mediated endocytosis
KCNK7	12	4	2-oxobutanoate degradation I
SMOC2	11	4	pre-mRNA splicing
SLC39A13	10	4	Mitochondrial translation elongation;Mitochondrial translation initiation;Mitochondrial translation termination

Table D (continued)

Regulator	Targs	D'sets	Main pathway
PEBP4	8	4	Activation of AMPA receptors;Activation of Ca-permeable Kainate Receptor;Activation of CaMK IV;Activation of NF-kappaB in B cells;Activation of the phototransduction cascade;Amyloid fiber formation;Antigen activates B Cell Receptor (BCR) leading to generation of second messengers;CLEC7A (Dectin-1) induces NFAT activation;CREB phosphorylation through the activation of Adenylate Cyclase;CREB phosphorylation through the activation of CaMKII;Ca2+ pathway;CaM pathway;CaMK IV-mediated phosphorylation of CREB;Calcineurin activates NFAT;Calmodulin induced events;Cam-PDE 1 activation;Chromatin modifying enzymes;DAG and IP3 signaling;DARPP-32 events;Defective SLC24A1 causes congenital stationary night blindness 1D (CSNB1D);Defective SLC24A4 causes hypomineralized amelogenesis imperfecta (AI);Degradation of the extracellular matrix;Deregulated CDK5 triggers multiple neurodegenerative pathways in Alzheimer's disease models;Effects of PIP2 hydrolysis;Elevation of cytosolic Ca2+ levels;FCERI mediated Ca+2 mobilization;FCERI mediated NF-kB activation;Glutamate Neurotransmitter Release Cycle;Glycerophospholipid biosynthesis;Glycogen breakdown (glycogenolysis);Glycosphingolipid metabolism;Highly calcium permeable nicotinic acetylcholine receptors;Highly calcium permeable postsynaptic nicotinic acetylcholine receptors;Inactivation, recovery and regulation of the phototransduction cascade;Ion homeostasis;Ion transport by P-type ATPases;Mitochondrial calcium ion transport;Neurotransmitter receptors and postsynaptic signal transmission;PKA activation;PLC beta mediated events;Phase 0 - rapid depolarisation;Phase 2 - plateau phase;Presynaptic depolarization and calcium channel opening;RHO GTPases activate PAKs;Rap1 signalling;Ras activation upon Ca2+ influx through NMDA receptor;Reduction of cytosolic Ca++ levels;Regulation of insulin secretion;Response to elevated platelet cytosolic Ca2+;Role of phospholipids in phagocytosis;Smooth Muscle Contraction;Sodium/Calcium exchangers;Sperm Motility And Taxes;Stimuli-sensing channels;Striated Muscle Contraction;Synthesis of Leukotrienes (LT) and Eoxins (EX);TRP channels;Tetrahydrobiopterin (BH4) synthesis, recycling, salvage and regulation;Translocation of GLUT4 to the plasma membrane;Unblocking of NMDA receptor, glutamate binding and activation;Uptake and function of anthrax toxins;VEGFR2 mediated cell proliferation;VEGFR2 mediated vascular permeability;eNOS activation;phospho-PLA2 pathway
PTPRE	7	4	Integrin signalling pathway
S100A14	6	4	Deposition of new CENPA-containing nucleosomes at the centromere
SLC27A3	6	4	Amplification of signal from unattached kinetochores via a MAD2 inhibitory signal;Mitotic Prometaphase;RHO GTPases Activate Formins;Resolution of Sister Chromatid Cohesion;Separation of Sister Chromatids

Table D (continued)

Regulator	Targs	D'sets	Main pathway
DUOX1	5	4	Eukaryotic Translation Termination;Formation of a pool of free 40S subunits;GTP hydrolysis and joining of the 60S ribosomal subunit;L13a-mediated translational silencing of Ceruloplasmin expression;Major pathway of rRNA processing in the nucleolus and cytosol;Nonsense Mediated Decay (NMD) enhanced by the Exon Junction Complex (EJC);Nonsense Mediated Decay (NMD) independent of the Exon Junction Complex (EJC);Peptide chain elongation;Regulation of expression of SLITs and ROBOs;SRP-dependent cotranslational protein targeting to membrane;Selenocysteine synthesis;Viral mRNA Translation
EMILIN1	5	4	pre-mRNA splicing
PAX6	5	4	Nonsense Mediated Decay (NMD) enhanced by the Exon Junction Complex (EJC)
ARHGAP30	4	4	Rho GTPase cycle
ICAM3	4	4	Cleavage of Growing Transcript in the Termination Region;Processing of Intronless Pre-mRNAs;Transport of Mature mRNA Derived from an Intronless Transcript;mRNA 3'-end processing;pre-mRNA splicing
DAAM2	3	4	PKMTs methylate histone lysines;RUNX1 regulates genes involved in megakaryocyte differentiation and platelet function
SGCZ	3	4	Amplification of signal from unattached kinetochores via a MAD2 inhibitory signal;Mitotic Prometaphase;RHO GTPases Activate Formins;Resolution of Sister Chromatid Cohesion;Separation of Sister Chromatids
SRPX	3	4	Deposition of new CENPA-containing nucleosomes at the centromere
TMEM37	3	4	Anchoring of the basal body to the plasma membrane
RPGR	2	4	Negative regulation of MAPK pathway
RPS23	2	4	Nonsense Mediated Decay (NMD) enhanced by the Exon Junction Complex (EJC)
TMEM259	2	4	pre-mRNA splicing
ADRB2	1	4	Ub-specific processing proteases
KDELR1	1	4	Eukaryotic Translation Termination;Formation of a pool of free 40S subunits;GTP hydrolysis and joining of the 60S ribosomal subunit;Nonsense Mediated Decay (NMD) enhanced by the Exon Junction Complex (EJC);Nonsense Mediated Decay (NMD) independent of the Exon Junction Complex (EJC);Peptide chain elongation;Regulation of expression of SLITs and ROBOs;SRP-dependent cotranslational protein targeting to membrane;Selenocysteine synthesis;Viral mRNA Translation
PFKFB1	1	4	gluconeogenesis I;glycolysis I
RILP	1	4	MHC class II antigen presentation
SNAPC2	1	4	RNA polymerase II transcribes snRNA genes
TAF7	1	4	HDACs deacetylate histones;NoRC negatively regulates rRNA expression
MNS1	41	3	G alpha (s) signalling events;Vasopressin regulates renal water homeostasis via Aquaporins

Table D (continued)

Regulator	Targs	D'sets	Main pathway
TMOD4	15	3	G2/M DNA damage checkpoint;Recruitment and ATM-mediated phosphorylation of repair and signaling proteins at DNA double strand breaks
TNNT2	12	3	TNFR2 non-canonical NF-kB pathway
LYL1	9	3	Activation of anterior HOX genes in hindbrain development during early embryogenesis;Deposition of new CENPA-containing nucleosomes at the centromere;PRC2 methylates histones and DNA
RIPK3	9	3	Major pathway of rRNA processing in the nucleolus and cytosol
ADGRB1	8	3	COPI-dependent Golgi-to-ER retrograde traffic;COPI-mediated anterograde transport
RFTN1	8	3	RHO GTPases Activate WASPs and WAVEs;Regulation of actin dynamics for phagocytic cup formation
TSPAN7	8	3	Amplification of signal from unattached kinetochores via a MAD2 inhibitory signal;Mitotic Prometaphase;RHO GTPases Activate Formins;Resolution of Sister Chromatid Cohesion;Separation of Sister Chromatids
DAPK3	7	3	Formation of the Early Elongation Complex;Formation of the HIV-1 Early Elongation Complex
MECOM	7	3	PKMTs methylate histone lysines
NPY4R	7	3	G alpha (q) signalling events;G alpha (s) signalling events;Vasopressin regulates renal water homeostasis via Aquaporins
KCNN3	6	3	SRP-dependent cotranslational protein targeting to membrane
PART1	6	3	Cellular hexose transport;Phase 0 - rapid depolarisation;Stimuli-sensing channels;TRP channels
ZNF446	6	3	pre-mRNA splicing
AR	5	3	Ub-specific processing proteases
NUP210L	5	3	Carboxyterminal post-translational modifications of tubulin;Post-chaperonin tubulin folding pathway
STARD4	4	3	Eukaryotic Translation Termination;Formation of a pool of free 40S subunits;GTP hydrolysis and joining of the 60S ribosomal subunit;L13a-mediated translational silencing of Ceruloplasmin expression;Major pathway of rRNA processing in the nucleolus and cytosol;Nonsense Mediated Decay (NMD) enhanced by the Exon Junction Complex (EJC);Nonsense Mediated Decay (NMD) independent of the Exon Junction Complex (EJC);Peptide chain elongation;Regulation of expression of SLITs and ROBOs;SRP-dependent cotranslational protein targeting to membrane;Selenocysteine synthesis;Viral mRNA Translation
SULT4A1	4	3	HDR through Homologous Recombination (HRR);Homologous DNA Pairing and Strand Exchange;Presynaptic phase of homologous DNA pairing and strand exchange;Resolution of D-loop Structures through Holliday Junction Intermediates;Resolution of D-loop Structures through Synthesis-Dependent Strand Annealing (SDSA)

Table D (continued)

Regulator	Targs	D'sets	Main pathway
TLE2	4	3	Eukaryotic Translation Termination;Formation of a pool of free 40S subunits;Formation of the ternary complex, and subsequently, the 43S complex;GTP hydrolysis and joining of the 60S ribosomal subunit;L13a-mediated translational silencing of Ceruloplasmin expression;Major pathway of rRNA processing in the nucleolus and cytosol;Nonsense Mediated Decay (NMD) enhanced by the Exon Junction Complex (EJC);Nonsense Mediated Decay (NMD) independent of the Exon Junction Complex (EJC);Peptide chain elongation;Regulation of expression of SLITs and ROBOs;Ribosomal scanning and start codon recognition;SRP-dependent cotranslational protein targeting to membrane;Selenocysteine synthesis;Translation initiation complex formation;Viral mRNA Translation
PITX1	3	3	RNA polymerase II transcribes snRNA genes
SPINK4	3	3	p53 pathway
ZNF488	3	3	Beta1 integrin cell surface interactions
ABCC12	1	3	Activated NTRK2 signals through PI3K;Activation of PKB;Antigen activates B Cell Receptor (BCR) leading to generation of second messengers;CD28 dependent PI3K/Akt signaling;CD28 dependent Vav1 pathway;Constitutive Signaling by Aberrant PI3K in Cancer;Constitutive Signaling by EGFRvIII;Constitutive Signaling by Ligand-Responsive EGFR Cancer Variants;DAP12 signaling;Downstream TCR signaling;Downstream signal transduction;FCERI mediated Ca+2 mobilization;FCERI mediated MAPK activation;G beta:gamma signalling through PI3Kgamma;GAB1 signalosome;GPVI-mediated activation cascade;Inhibition of TSC complex formation by PKB;MET activates PI3K/AKT signaling;Negative regulation of the PI3K/AKT network;PDE3B signalling;PI Metabolism;PI-3K cascade:FGFR1;PI-3K cascade:FGFR2;PI-3K cascade:FGFR3;PI-3K cascade:FGFR4;PI3K Cascade;PI3K events in ERBB2 signaling;PI3K events in ERBB4 signaling;PI3K/AKT Signaling;PI3K/AKT activation;PI5P, PP2A and IER3 Regulate PI3K/AKT Signaling;PTEN Loss of Function in Cancer;Phospholipase C-mediated cascade: FGFR1;Phospholipase C-mediated cascade; FGFR2;Phospholipase C-mediated cascade; FGFR3;Phospholipase C-mediated cascade; FGFR4;RHO GTPases Activate WASPs and WAVEs;RHO GTPases activate PKNs;RIPK1-mediated regulated necrosis;Regulation of TP53 Degradation;Regulation of actin dynamics for phagocytic cup formation;Role of LAT2/NTAL/LAB on calcium mobilization;Role of phospholipids in phagocytosis;Signaling by FGFR1 in disease;Signaling by FGFR2 in disease;Signaling by FGFR3 fusions in cancer;Signaling by FGFR3 point mutants in cancer;Signaling by FGFR4 in disease;Signaling by SCF-KIT;Signaling by cytosolic FGFR1 fusion mutants;Synthesis of PIPs at the plasma membrane;VEGFA-VEGFR2 Pathway;VEGFR2 mediated vascular permeability

Table D (continued)

Regulator	Targs	D'sets	Main pathway
SELPLG	1	3	Arf1 pathway;BCR signaling pathway;CXCR4-mediated signaling events;Class I PI3K signaling events;E-cadherin signaling in keratinocytes;EPO signaling pathway;Endothelins;ErbB1 downstream signaling;FGF signaling pathway;Fc-epsilon receptor I signaling in mast cells;IL2 signaling events mediated by PI3K;IL8- and CXCR1-mediated signaling events;IL8- and CXCR2-mediated signaling events;LPA receptor mediated events;LPA4-mediated signaling events;Nephrin/Neph1 signaling in the kidney podocyte;Netrin-mediated signaling events;Nongenotropic Androgen signaling;PAR1-mediated thrombin signaling events;PDGFR-alpha signaling pathway;PDGFR-beta signaling pathway;Plasma membrane estrogen receptor signaling;Regulation of Ras family activation;Role of Calcineurin-dependent NFAT signaling in lymphocytes;Signaling events mediated by Hepatocyte Growth Factor Receptor (c-Met);Signaling events mediated by VEGFR1 and VEGFR2;TCR signaling in nave CD4+ T cells;TCR signaling in nave CD8+ T cells;Thromboxane A2 receptor signaling;Trk receptor signaling mediated by PI3K and PLC-gamma;VEGFR1 specific signals
HOXD9	54	2	Gap-filling DNA repair synthesis and ligation in TC-NER;Processing of DNA double-strand break ends

Table D (continued)

Regulator	Targs	D'sets	Main pathway
ETV7	32	2	Activation of AMPA receptors;Activation of Ca-permeable Kainate Receptor;Activation of CaMK IV;Activation of NF-kappaB in B cells;Activation of the phototransduction cascade;Amyloid fiber formation;Antigen activates B Cell Receptor (BCR) leading to generation of second messengers;CLEC7A (Dectin-1) induces NFAT activation;CREB phosphorylation through the activation of Adenylate Cyclase;CREB phosphorylation through the activation of CaMKII;Ca2+ pathway;CaM pathway;CaMK IV-mediated phosphorylation of CREB;Calcineurin activates NFAT;Calmodulin induced events;Cam-PDE 1 activation;Chromatin modifying enzymes;DAG and IP3 signaling;DARPP-32 events;Defective SLC24A1 causes congenital stationary night blindness 1D (CSNB1D);Defective SLC24A4 causes hypomineralized amelogenesis imperfecta (AI);Degradation of the extracellular matrix;Deregulated CDK5 triggers multiple neurodegenerative pathways in Alzheimer's disease models;Effects of PIP2 hydrolysis;Elevation of cytosolic Ca2+ levels;FCERI mediated Ca+2 mobilization;FCERI mediated NF-kB activation;Glutamate Neurotransmitter Release Cycle;Glycerophospholipid biosynthesis;Glycogen breakdown (glycogenolysis);Glycosphingolipid metabolism;Highly calcium permeable nicotinic acetylcholine receptors;Highly calcium permeable postsynaptic nicotinic acetylcholine receptors;Inactivation, recovery and regulation of the phototransduction cascade;Ion homeostasis;Ion transport by P-type ATPases;Mitochondrial calcium ion transport;Neurotransmitter receptors and postsynaptic signal transmission;PKA activation;PLC beta mediated events;Phase 0 - rapid depolarisation;Phase 2 - plateau phase;Presynaptic depolarization and calcium channel opening;RHO GTPases activate PAKs;Rap1 signalling;Ras activation upon Ca2+ influx through NMDA receptor;Reduction of cytosolic Ca++ levels;Regulation of insulin secretion;Response to elevated platelet cytosolic Ca2+;Role of phospholipids in phagocytosis;Smooth Muscle Contraction;Sodium/Calcium exchangers;Sperm Motility And Taxes;Stimuli-sensing channels;Striated Muscle Contraction;Synthesis of Leukotrienes (LT) and Eoxins (EX);TRP channels;Tetrahydrobiopterin (BH4) synthesis, recycling, salvage and regulation;Translocation of GLUT4 to the plasma membrane;Unblocking of NMDA receptor, glutamate binding and activation;Uptake and function of anthrax toxins;VEGFR2 mediated cell proliferation;VEGFR2 mediated vascular permeability;eNOS activation;phospho-PLA2 pathway
CDX1	29	2	Major pathway of rRNA processing in the nucleolus and cytosol;rRNA modification in the nucleus and cytosol

Table D (continued)

Regulator	Targs	D'sets	Main pathway
KLK6	25	2	Eukaryotic Translation Termination;Formation of a pool of free 40S subunits;GTP hydrolysis and joining of the 60S ribosomal subunit;L13a-mediated translational silencing of Ceruloplasmin expression;Major pathway of rRNA processing in the nucleolus and cytosol;Nonsense Mediated Decay (NMD) enhanced by the Exon Junction Complex (EJC);Nonsense Mediated Decay (NMD) independent of the Exon Junction Complex (EJC);Peptide chain elongation;Regulation of expression of SLITs and ROBOs;SRP-dependent cotranslational protein targeting to membrane;Selenocysteine synthesis;Viral mRNA Translation
GUCA2B	21	2	Amplification of signal from unattached kinetochores via a MAD2 inhibitory signal;Mitotic Prometaphase;RHO GTPases Activate Formins;Resolution of Sister Chromatid Cohesion;Separation of Sister Chromatids
NIPAL4	20	2	pre-mRNA splicing
SOX14	20	2	Amplification of signal from unattached kinetochores via a MAD2 inhibitory signal;Mitotic Prometaphase;RHO GTPases Activate Formins;Resolution of Sister Chromatid Cohesion;Separation of Sister Chromatids
RASSF5	18	2	Budding and maturation of HIV virion;Membrane binding and targetting of GAG proteins
ADAMTSL4	16	2	Mitochondrial translation elongation;Mitochondrial translation initiation;Mitochondrial translation termination
GIMAP2	10	2	Transcriptional regulation by small RNAs
CFHR4	7	2	Major pathway of rRNA processing in the nucleolus and cytosol;rRNA modification in the nucleus and cytosol
HHLA1	7	2	Heterotrimeric G-protein signaling pathway-Gi alpha and Gs alpha mediated pathway
NAALADL2	6	2	Amplification of signal from unattached kinetochores via a MAD2 inhibitory signal;Mitotic Prometaphase;RHO GTPases Activate Formins;Resolution of Sister Chromatid Cohesion;Separation of Sister Chromatids
PDE4C	6	2	Major pathway of rRNA processing in the nucleolus and cytosol
EDN3	5	2	Heterotrimeric G-protein signaling pathway-Gi alpha and Gs alpha mediated pathway;Muscarinic acetylcholine receptor 2 and 4 signaling pathway
KCNA2	5	2	TCR signaling in nave CD4+ T cells
WDR49	5	2	COPI-dependent Golgi-to-ER retrograde traffic
DENND1C	4	2	Eukaryotic Translation Termination;Formation of a pool of free 40S subunits;GTP hydrolysis and joining of the 60S ribosomal subunit;Nonsense Mediated Decay (NMD) enhanced by the Exon Junction Complex (EJC);Nonsense Mediated Decay (NMD) independent of the Exon Junction Complex (EJC);Peptide chain elongation;Regulation of expression of SLITs and ROBOs;SRP-dependent cotranslational protein targeting to membrane;Selenocysteine synthesis;Viral mRNA Translation
FAM83E	4	2	Activated NOTCH1 Transmits Signal to the Nucleus
FEV	4	2	Processing of Capped Intron-Containing Pre-mRNA
GSTP1	4	2	Validated targets of C-MYC transcriptional activation

Table D (continued)

Regulator	Targs	D'sets	Main pathway
LRRIQ4	4	2	Rho GTPase cycle
RAB3A	4	2	Activation of anterior HOX genes in hindbrain development during early embryogenesis
GALE	3	2	Antigen activates B Cell Receptor (BCR) leading to generation of second messengers;Ca2+ pathway;G beta:gamma signalling through PLC beta;GPVI-mediated activation cascade;Synthesis of IP2, IP, and Ins in the cytosol
IFI16	3	2	IRF3-mediated induction of type I IFN
ARRDC2	2	2	Budding and maturation of HIV virion;Endosomal Sorting Complex Required For Transport (ESCRT)
COL4A5	2	2	Fanconi anemia pathway
HIST2H2BE	2	2	Transcriptional regulation by small RNAs
KLK11	2	2	Fanconi Anemia Pathway
CAMK2A	1	2	Respiratory electron transport
FCRL5	1	2	Eukaryotic Translation Termination;Formation of a pool of free 40S subunits;GTP hydrolysis and joining of the 60S ribosomal subunit;Nonsense Mediated Decay (NMD) enhanced by the Exon Junction Complex (EJC);Nonsense Mediated Decay (NMD) independent of the Exon Junction Complex (EJC);Peptide chain elongation;Regulation of expression of SLITs and ROBOs;SRP-dependent cotranslational protein targeting to membrane;Selenocysteine synthesis;Viral mRNA Translation
JAML	1	2	Alpha4 beta1 integrin signaling events
SH2D3C	1	2	Glucuronidation
LINC00051	106	1	Cation-coupled Chloride cotransporters;Neurotransmitter receptors and postsynaptic signal transmission
GCG	81	1	snRNP Assembly
MUC5B	46	1	Formation of a pool of free 40S subunits;Formation of the ternary complex, and subsequently, the 43S complex;GTP hydrolysis and joining of the 60S ribosomal subunit;L13a-mediated translational silencing of Ceruloplasmin expression;Ribosomal scanning and start codon recognition;Translation initiation complex formation
GKN1	43	1	Major pathway of rRNA processing in the nucleolus and cytosol;rRNA modification in the nucleus and cytosol
HTN1	27	1	Cation-coupled Chloride cotransporters;Phase 0 - rapid depolarisation;Stimuli-sensing channels;TRP channels
NPIPB15	14	1	pre-mRNA splicing
ZEB1	14	1	Integrin signalling pathway

Table D (continued)

Regulator	Targs	D'sets	Main pathway
PGK2	13	1	ABC-family proteins mediated transport;ATP sensitive Potassium channels;Activation of G protein gated Potassium channels;Ca activated K+ channels;Classical Kir channels;Defective ABCC8 can cause hypoglycemias and hyperglycemias;HCN channels;Inhibition of voltage gated Ca2+ channels via Gbeta/gamma subunits;Ion homeostasis;Ion transport by P-type ATPases;Phase 1 - inactivation of fast Na+ channels;Phase 2 - plateau phase;Phase 3 - rapid repolarisation;Phase 4 - resting membrane potential;Potassium transport channels;Sperm Motility And Taxes;TWIK related potassium channel (TREK);TWIK-related alkaline pH activated K+ channel (TALK);TWIK-related spinal cord K+ channel (TRESK);TWIK-releated acid-sensitive K+ channel (TASK);Tandem of pore domain in a weak inwardly rectifying K+ channels (TWIK);Tandem pore domain halothane-inhibited K+ channel (THIK);The NLRP3 inflammasome;Voltage gated Potassium channels
DQX1	8	1	PKMTs methylate histone lysines
NTRK1	8	1	Abortive elongation of HIV-1 transcript in the absence of Tat;Formation of HIV elongation complex in the absence of HIV Tat;Formation of HIV-1 elongation complex containing HIV-1 Tat;Formation of RNA Pol II elongation complex;Formation of the Early Elongation Complex;Formation of the HIV-1 Early Elongation Complex;HIV elongation arrest and recovery;Pausing and recovery of HIV elongation;Pausing and recovery of Tat-mediated HIV elongation;RNA Polymerase II Pre-transcription Events;RNA Polymerase II Transcription Elongation;TP53 Regulates Transcription of DNA Repair Genes;Tat-mediated HIV elongation arrest and recovery;Tat-mediated elongation of the HIV-1 transcript
CLDND2	6	1	SRP-dependent cotranslational protein targeting to membrane
FPR2	6	1	Integrin signalling pathway
PAM16	6	1	Mitotic Prometaphase;RHO GTPases Activate Formins
PCDHB9	6	1	pre-mRNA splicing
FAM212B	5	1	Major pathway of rRNA processing in the nucleolus and cytosol
KRTAP15-1	5	1	AURKA Activation by TPX2;Anchoring of the basal body to the plasma membrane;Loss of Nlp from mitotic centrosomes;Loss of proteins required for interphase microtubule organization-from the centrosome;Recruitment of NuMA to mitotic centrosomes;Recruitment of mitotic centrosome proteins and complexes;Regulation of PLK1 Activity at G2/M Transition
WFIKKN1	5	1	HDACs deacetylate histones
ADH1C	4	1	AURKA Activation by TPX2;Anchoring of the basal body to the plasma membrane;Loss of Nlp from mitotic centrosomes;Loss of proteins required for interphase microtubule organization-from the centrosome;Recruitment of NuMA to mitotic centrosomes;Recruitment of mitotic centrosome proteins and complexes;Regulation of PLK1 Activity at G2/M Transition
KCNK4	4	1	Major pathway of rRNA processing in the nucleolus and cytosol;rRNA modification in the nucleus and cytosol

Table D (continued)

Regulator	Targs	D'sets	Main pathway
SLC25A2	4	1	APC-Cdc20 mediated degradation of Nek2A;APC/C:Cdc20 mediated degradation of Cyclin B;APC/C:Cdc20 mediated degradation of Securin;APC/C:Cdc20 mediated degradation of mitotic proteins;APC/C:Cdh1 mediated degradation of Cdc20 and other APC/C:Cdh1 targeted proteins in late mitosis/early G1;Antigen processing: Ubiquitination and Proteasome degradation;Autodegradation of Cdh1 by Cdh1:APC/C;CDK-mediated phosphorylation and removal of Cdc6;Cdc20:Phospho-APC/C mediated degradation of Cyclin A;Conversion from APC/C:Cdc20 to APC/C:Cdh1 in late anaphase;Inactivation of APC/C via direct inhibition of the APC/C complex;Phosphorylation of the APC/C;Regulation of APC/C activators between G1/S and early anaphase;Senescence-Associated Secretory Phenotype (SASP);Separation of Sister Chromatids
HDAC10	3	1	HDACs deacetylate histones
RPRM	3	1	SRP-dependent cotranslational protein targeting to membrane
ALDH3A1	2	1	Complex I biogenesis;Respiratory electron transport
ELL3	2	1	RNA polymerase II transcribes snRNA genes
HAMP	2	1	Cyclin A/B1/B2 associated events during G2/M transition
LST1	2	1	Retinoid cycle disease events;The canonical retinoid cycle in rods (twilight vision)
FCRL2	1	1	APC/C:Cdc20 mediated degradation of Securin;APC/C:Cdh1 mediated degradation of Cdc20 and other APC/C:Cdh1 targeted proteins in late mitosis/early G1;Autodegradation of Cdh1 by Cdh1:APC/C;Cdc20:Phospho-APC/C mediated degradation of Cyclin A;Separation of Sister Chromatids
LINC01587	1	1	Amplification of signal from unattached kinetochores via a MAD2 inhibitory signal;Mitotic Prometaphase;RHO GTPases Activate Formins;Resolution of Sister Chromatid Cohesion;Separation of Sister Chromatids
PCK1	1	1	adenine and adenosine salvage III;adenosine nucleotides degradation II;guanine and guanosine salvage I;guanosine nucleotides degradation III;purine nucleotides degradation II (aerobic);purine ribonucleosides degradation to ribose-1-phosphate;urate biosynthesis/inosine 5'-phosphate degradation;xanthine and xanthosine salvage
