# Supplementary material for: A meta-analysis of multiple matched aCGH/expression cancer datasets reveals regulatory relationships and pathway enrichment of potential oncogenes
Source: PLoS One. 2019 Jul 23;14(7):e0213221. doi: 10.1371/journal.pone.0213221 (PMC6650054; doi:10.1371/journal.pone.0213221)
Supplement: S5 File — (PDF) [file pone.0213221.s005.pdf]

S5 File: Supplementary Results - Network analysis

...

"A meta-analysis of multiple matched aCGH/expression cancer datasets reveals regulatory relationships and pathway enrichment of potential oncogenes"

...

Richard Newton & Lorenz Wernisch

June 11, 2019

1 Supplementary Results

1.1 Network analysis

1.1.1 Pathway Example 4: PKMTs methylate histone lysines

Protein lysine methyltransferases (PKMTs) are enzymes that modify histones, the main protein component of chromatin, as part of the epigenetic control of gene transcription. Disruption of protein methyltransferases activity has been linked with a number of types of cancer [[1]].

Nine regulators have target lists enriched with this pathway: *POGZ* (20), *RBBP5* (16), *ASH1L* (12), *KMT2E* (9), *PARP10* (8), *DAAM2* (4), *MECOM* (3), *LYL1* (3) and *DQX1* (1).

POGZ (Pogo Transposable Element Derived With ZNF Domain) is not known to be part of this pathway and has no known connection to its members in Reactome. POGZ does however have an HP1-binding motif [[2]] and HP1α binds histone H3 di- or tri-methylated at position lysine 9 (H3K9me2/3) performing an essential role in the formation of higher order chromatin structures and implicated in the epigenetic control mechanisms involved in aberrant cell proliferation and metastasis [[3, 4]].

RBBP5 (RB Binding Protein 5), *ASH1L (ASH1 Like Histone Lysine Methyltransferase)* and *KMT2E (Lysine Methyltransferase 2E)* are annotated with this pathway in Reactome.

PARP10 (Poly(ADP-Ribose) Polymerase Family Member 10) has self-correlation in 8 datasets and has 21 significant best targets, 4 of which are associated by the LEAN analysis with this pathway, namely *CREBBP*, *CDKAL1*, *GATAD2B*, *NSD1*. Of the other 17 significant best targets, 8 are not annotated in Reactome whilst the remaining targets have some path to members of the pathway. *PARP10* is not annotated with this pathway but does have a path to members of the pathway in Reactome. *PARP10* codes an enzyme involved in the control of cell proliferation, is overexpressed in many human tumours and is involved in tumour metastasis suppression via negative regulation of Aurora A activity [[5, 6]].

1.1.2 Pathway Example 5: Fanconi Anemia Pathway

The Fanconi anemia (FA) pathway is a DNA damage repair (DDR) pathway with additional cytoprotective functions that may be independent of DDR, such as selective autophagy. The pathway comprises a complex that recognizes DNA interstrand crosslinks, a multisubunit ubiquitin ligase and downstream repair proteins including nucleases and homologous recombination enzymes [[7]]. FA itself is not a common disease, but disruption of FA pathway genes are found in many types of cancer [[8, 9]].

Eight regulators in Metamatched have the FA pathway enriched in their target lists, *DCTN4* (14), *RNF139* (13), *PAM* (11), *UBE2T* (9), *IL6R* (6), *CDX1* (2), *GALE* (2) and *KLK11* (2). Figure 6 shows a simplified pathway diagram derived by amalgamating the results of the LEAN analysis for these eight regulators.

DCTN4 (Dynactin Subunit 4) has significant self-correlation in 14 datasets and one significant best target, *FANCM*, which is part of the FA core complex. There are paths in Reactome from *DCTN4* to members of the pathway. If the less stringent condition for a regulator to be the best regulator is applied then a second significant best target, *DBF4*, features in the pathway.

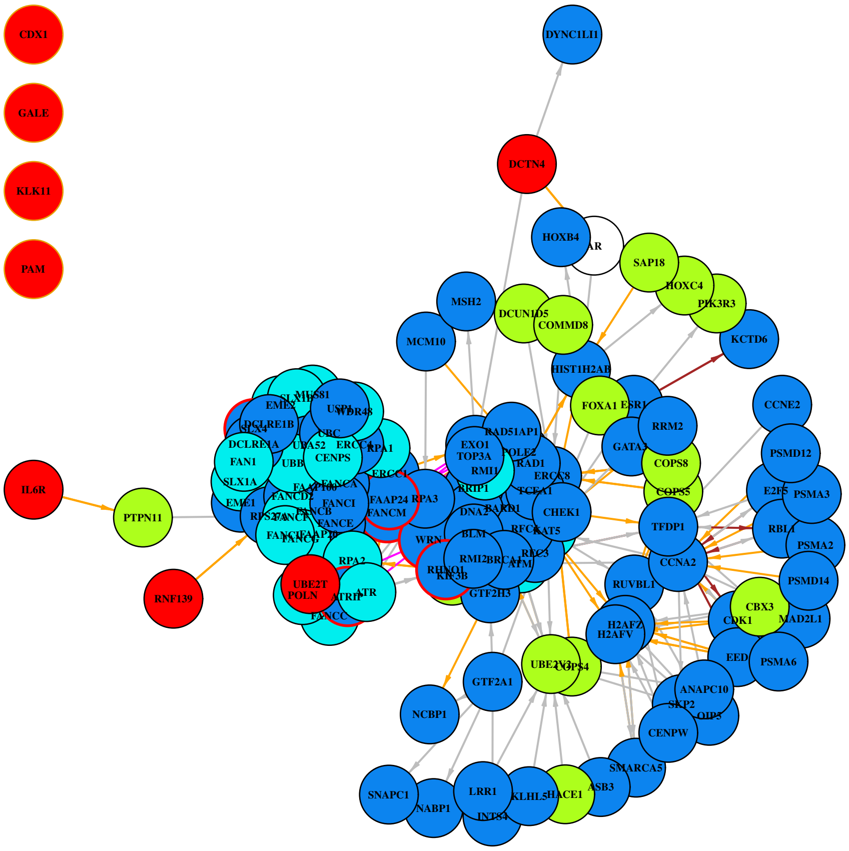

Figure S1: Fanconi Anemia pathway: diagram fusing the results of the LEAN analysis. Simplified diagram of the Fanconi Anemia pathway fusing the results of the LEAN analysis for DCTN4, RNF139, PAM, UBE2T, IL6R, CDX1, GALE and KLK11.
Nodes:
Green = significant target, Red border = regulator is best regulator for target
Dark Blue = enriched centre also significant target,
Light Blue = enriched centre but not significant target, White = target not significant,
Red = regulator.
Edges:
grey = ‘in-complex-with’, purple = ‘catalysis-precedes’, ‘used-to-produce’
orange = ‘controls-state-change-of’, ‘controls-phosphorylation-of’
brown = ‘controls-expression-of’, ‘cntrls-production-of’, consumption-cntrlled-by’

RNF139 (Ring Finger Protein 139) has self-correlation in 13 datasets and has 6 significant best targets. Disruption of *RNF139* is known to be associated with a number of cancers due to its function as a negative regulator of cell proliferation [[10, 11, 12]]. It has two significant best targets in the pathway, *MRE11A* and *ATRIP*. Whilst not annotated as belonging to this pathway in Reactome it is connected to the pathway via gene *RPS27A*. Of its four other significant best targets two do have a path to members of the pathway, *DDO* and *TEK*, whilst two are not annotated in Reactome, *BOLA1* and *DDX12P*. If the condition for a regulator to be the best regulator for a target is made less stringent then a further 5 significant best targets of *RNF139* occur in this pathway.

PAM (Peptidylglycine Alpha-Amidating Monooxygenase) has self-correlation in 11 datasets and has 1 significant target, like *RNF139* this target is *MRE11A*, but in a repressive rather than activating role. If the less stringent condition is applied then a further significant

best target is included, *RMI2*. *PAM* is not known to be connected to this pathway. It has been shown to play a role in the development of antiandrogen drug resistance in prostate cancer through activating autocrine growth, and many lung cancer cell lines excrete *PAM* into their culture media [[13]].

UBE2T (Ubiquitin Conjugating Enzyme E2 T) is annotated with the Fanconi Anemia pathway in Reactome and Pid. It has six significant best targets, one of which, *RHNO1*, is directly connected to members of this pathway.

References

[1] Copeland RA. Protein methyltransferase inhibitors as precision cancer therapeutics: a decade of discovery. Philosophical Transactions of the Royal Society of London B: Biological Sciences. 2018;373(1748). doi:10.1098/rstb.2017.0080.

[2] Nozawa RS, Nagao K, Masuda HT, Iwasaki O, Hirota T, Nozaki N, et al. Human POGZ modulates dissociation of HP1 from mitotic chromosome arms through Aurora B activation. Nature Cell Biology. 2010;12:719. doi:10.1038/ncb2075.

[3] Vad-Nielsen J, Nielsen AL. Beyond the histone tale: HP1α deregulation in breast cancer epigenetics. Cancer Biology & Therapy. 2015;16(2):189–200. doi:10.1080/15384047.2014.1001277.

[4] Choi J, , J-S L. Interplay between Epigenetics and Genetics in Cancer. Genomics Inform. 2013;11(4):164–173. doi:10.5808/GI.2013.11.4.164.

[5] Schleicher EM, Galvan AM, Imamura-Kawasawa Y, Moldovan GL, Nicolae CM. PARP10 promotes cellular proliferation and tumorigenesis by alleviating replication stress. Nucleic Acids Research. 2018; p. gky658. doi:10.1093/nar/gky658.

[6] Zhao Y, Hu X, Wei L, Song D, Wang J, You L, et al. PARP10 suppresses tumor metastasis through regulation of Aurora A activity. Oncogene. 2018;37(22):2921 – 2935. doi:10.1038/s41388-018-0168-5.

[7] Sumpter R, Levine B. Emerging functions of the Fanconi anemia pathway at a glance. Journal of Cell Science. 2017;130(16):2657–2662. doi:10.1242/jcs.204909.

[8] Esteban-Jurado C, Franch-Expsito S, Muoz J, Ocaa T, Carballal S, Lpez-Cern M, et al. The Fanconi anemia DNA damage repair pathway in the spotlight for germline predisposition to colorectal cancer. European Journal Of Human Genetics. 2016;24:1501. doi:10.1038/ejhg.2016.44.

[9] Stoepker C, Ameziane N, van der Lelij P, Kooi IE, Oostra AB, Rooimans MA, et al. Defects in the Fanconi Anemia Pathway and Chromatid Cohesion in Head and Neck Cancer. Cancer Research. 2015;75(17):3543–3553. doi:10.1158/0008-5472.CAN-15-0528.

[10] Brauweiler A, Lorick KL, Lee JP, Tsai YC, Chan D, Weissman AM, et al. RING-dependent tumor suppression and G2/M arrest induced by the TRC8 hereditary kidney cancer gene. 101038/sjonc1210017. 2007;26:22632271. doi:10.1038/sj.onc.1210017.

[11] Wang XW, Wei W, Wang WQ, Zhao XY, Guo H, Fang DC. RING Finger Proteins Are Involved in the Progression of Barrett Esophagus to Esophageal Adenocarcinoma: A Preliminary Study. Gut and Liver. 2014;8(5):497–484. doi:10.5009/gnl13133.

[12] Beckner M, Pollack I, Nordberg M, Hamilton R. Glioblastomas with copy number gains in EGFR and RNF139 show increased expressions of carbonic anhydrase genes transformed by ENO1. BBA Clinical. 2016;5:1 – 15. doi:https://doi.org/10.1016/j.bbacli.2015.11.001.

[13] Trendel JA, Ellis N, Sarver JG, Klis WA, Dhananjeyan M, Bykowski CA, et al. Catalytically Active Peptidylglycine α-Amidating Monooxygenase in the Media of Androgen-Independent Prostate Cancer Cell Lines. Journal of Biomolecular Screening. 2008;13(8):804–809. doi:10.1177/1087057108321976.
